# Single-carbon-atom transfer to *para*-quinone methides from TMSCF_2_Br†

**DOI:** 10.1039/d5sc03234b

**Published:** 2025-06-04

**Authors:** Ruikang Sun, Pei Zhang, Yong Yan, Jie Zhu, Qirui Chen, Chi Yang, Aijun Lin, Xuanyi Li, Shang Gao, Hequan Yao

**Affiliations:** a State Key Laboratory of Natural Medicines (SKLNM), Department of Medicinal Chemistry, School of Pharmacy, China Pharmaceutical University Nanjing 210009 P. R. China gaoshang1990@cpu.edu.cn hyao@cpu.edu.cn; b Anhui Provincial Joint Key Laboratory for Innovative Drug Research and Industry Integration, School of Chemistry and Materials Engineering, Fuyang Normal University Fuyang 236037 P. R. China

## Abstract

Single-carbon-atom transfer reactions offer a powerful strategy for constructing complex molecular architectures by sequential assembly of substituents around the atomic carbon core. However, the limited availability of atomic carbon sources has significantly hindered progress in this field. Herein, we demonstrate a single-carbon atom transfer reaction utilizing commercially available TMSCF_2_Br as an atomic carbon equivalent. Through a cascade of 1,6-addition and TBAF-catalyzed intramolecular cyclization with *para*-quinone methides (*p*-QMs), *gem*-difluorinated spiro[2.5]octa-4,7-dien-6-ones were efficiently formed. These spirocyclic intermediates exhibit remarkable electrophilicity, enabling stereoselective capture of diverse nucleophiles to access fluorinated alkenes with excellent stereocontrol. The resulting fluoroalkenes serve as versatile platforms for constructing tetrasubstituted alkenes *via* nucleophilic vinylic substitution (S_N_V), achieving excellent stereoselectivities. In the presence of a 1,3-bisnucleophile, for example a C2-substituted acetoacetate ester, cyclic 2-methylene-2,3-dihydrofuran was generated *via* a sequential S_N_V reaction with excellent stereoselectivities. Moreover, a computational study and a control experiment provide insight into the mechanism of the reaction.

## Abstract 2

A single-carbon-atom transfer reaction to *para*-quinone methides with commercially available TMSCF_2_Br as an atomic carbon equivalent was developed.

## Graphical abstract

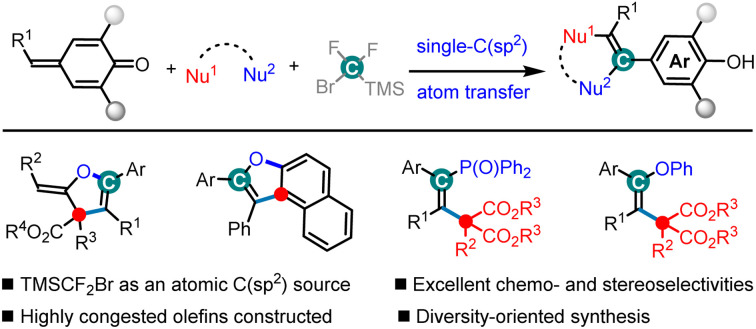

## Introduction

**Scheme 1:**
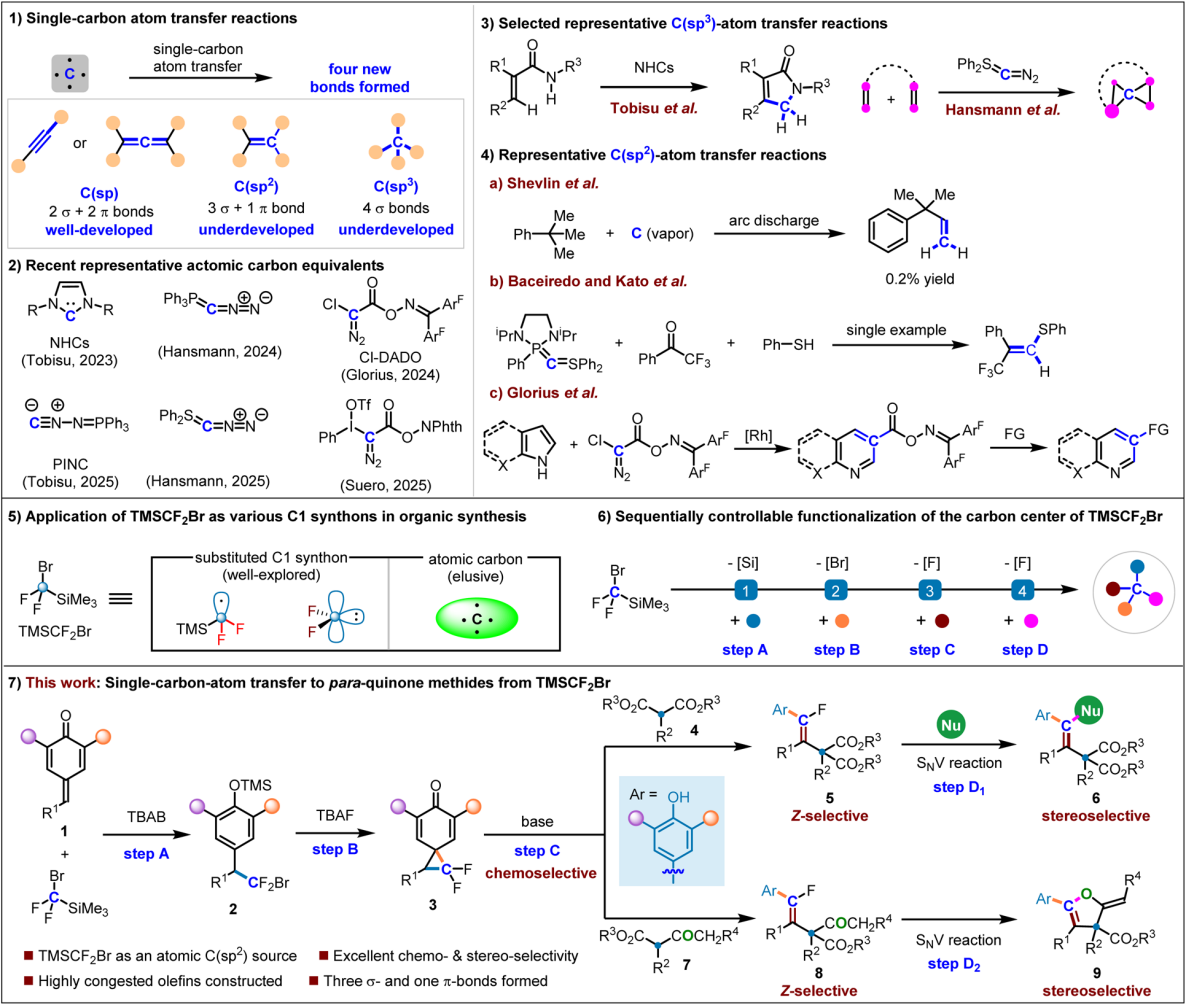
The chemistry of single-carbon atom transfer reaction.

Molecular skeleton editing has emerged as a transformative strategy for diversifying molecular complexity in synthetic chemistry.^1^ While advancements in late-stage functionalization have enabled precise modifications of (hetero)aromatic backbones,^2^ the incorporation of a single carbon atom into molecular frameworks with simultaneous formation of four bonds provides a fascinating platform to enhance molecular complexity beyond aromatic compounds (Scheme 1(1)).^3–10^ Wherein, single C(sp)-atom transfer reactions have been well-developed to construct alkynes and allenes, including several textbook reactions.^4^ In contrast, single C(sp^2^)- and C(sp^3^)-atom transfer reactions are still largely underdeveloped due to the limited types of atomic carbon sources. With the development of novel atomic carbon sources from the Tobisu,^5^ Hansmann,^6^ Glorius^7^ and Suero^8^ groups, especially the carbene precursors, significant breakthrough has been made over the past few years (Scheme 1(2)). For example, using N-heterocyclic carbene (NHC) and diazosulfur ylide (Ph_2_S=C=N_2_) reagents as single C(sp^3^)-atomic sources, γ-lactams and highly strained carbon spiro-centers were constructed by the Tobisu and Hansmann groups, respectively (Scheme 1(3)).^4,5^ Shevlin and colleagues made seminal contributions to the development of C(sp^2^)-atom transfer reactions with arc-discharge-generated carbon atoms and *tert*-butylbenzene systems.^9^ However, the synthetic utility of this method remains limited due to its remarkably low efficiency (Scheme 1(4a)). In 2010, Baceiredo and Kato reported another example with mixed *P*,*S*-bis(ylide) as a carbon atom source, allowing the creation of vinyl phenyl sulfide in quantitative yield *via* sequential elimination of phosphine oxide and Ph_2_S (Scheme 1(4b)).^10^ Very recently, Glorius's group developed a new reagent, chloro-diazoacetyl diarylmethanone oxime (Cl-DADO), enabling access to C3-functionalized quinolines *via* stepwise Rh-catalyzed carbyne insertion/functionalization of the oxime ester (Scheme 1(4c)).^7^ Despite these advances, further exploration of novel single-carbon-atom transfer reaction remains highly desirable yet challenging.

Commercially available TMSCF_2_Br presents an attractive yet underexploited reagent, offering triple functionality as a TMSCF_2_ radical donor, a difluorocarbene precursor and an atomic carbon equivalent (Scheme 1(5)).^11^ While the efficacy of the first two variants has been empirically validated,^12^ its implementation potential in single-carbon-atom transfer reactions remains elusive.^13^ Concurrently, *para*-quinone methides (*p*-QMs) have gained prominence as versatile synthons due to their inherent aromaticity-driven reactivity, particularly in 1,6-addition cascades.^14^ We envisioned that merging the *p*-QMs' reactivity with TMSCF_2_Br's latent single atomic carbon-donating capability would provide unprecedented opportunities for advancing single-carbon-atom transfer reactions. Several challenges are apparent for this transformation: (1) functionalization of TMSCF_2_Br in a sequentially controllable manner is essential to generate single atomic carbon doped products in synthetically useful yields (Scheme 1(6)). (2) The regioselectivity of the ring-opening of 1,1-difluoro-spiro[2.5]octa-4,7-dien-6-one 3 is not clear for different nucleophiles (Scheme 1(7)). (3) It is challenging to construct the highly congested tetra-substituted olefins 5, 6 and 8. (4) The control of the stereoselectivities for the formation of fully substituted olefin 6 and the chemoselectivities for the intramolecular S_N_V reaction of intermediate 8 to generate 9 is another challenge.^15^ With our continuing interest in the construction of stereodefined alkenes and the chemistry of *p*-QMs,^16^ we herein report a skeleton editing of *p*-QMs *via* a single-carbon atom transfer reaction, enabling the stereoselective construction of sterically hindered tetra-substituted fluoroalkenes and 2-methylene-2,3-dihydrofurans. The fluoroalkenes could serve as a platform to undergo diversity-oriented synthesis, giving sterically hindered tetra-substituted alkenes *via* a formal S_N_V reaction with good retention of the olefinic configuration. The *exo*-alkene motif of 2-methylene-2,3-dihydrofurans could undergo a series of late-stage transformations to enrich the structural diversity of the products. Of note is that the TMSCF_2_Br works as an atomic sp^2^-hybridized carbon.

## Results and discussion

To probe our hypothesis, we initiated our studies with *para*-quinone methide 1a and diethyl 2-methylmalonate 4a as the model substrates to construct fluoroalkene 5aa. Substrate 1a was first treated with TBAB in the presence of TMSCF_2_Br in toluene at 80 °C for 18 h,^17^ followed by the addition of TBAF, 4a and ^*t*^BuOK in THF. The tetra-substituted fluoroalkene 5aa was isolated in 42% yield with >20 : 1 *Z*-selectivity (Table 1, entry 1). Screening of bases indicated that the base had a great impact on the yield (entries 2–5). While the yield of 5aa could be further promoted to 75% in the presence of NaH, only trace product was detected when PhCO_2_Na was used; no product was detected using K_2_CO_3_ or KHCO_3_. Subsequent solvent optimization (entries 6–10) failed to improve outcomes. The yield of 5aa could be further improved to 87% after reducing the loading of TBAF to 0.4 equivalent (entry 11). Further decreasing the equivalent of TBAF did not improve the yield of 5aa (entry 12).

**Table 1:** Optimization of reaction conditions (steps A–C)^a^^,^^b^^,^^c^

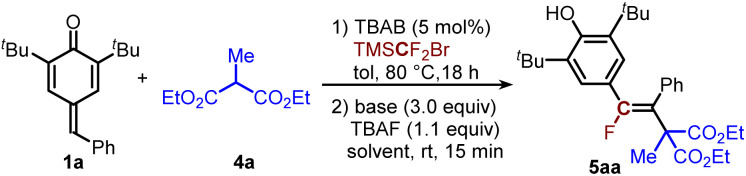				
Entry	Base	Solvent	Yield (%)	*Z*/*E*
1	^*t*^BuOK	THF	42	>20 : 1
2	K_2_CO_3_	THF	ND	—
3	KHCO_3_	THF	ND	—
4	PhCO_2_Na	THF	Trace	—
5	NaH	THF	75	>20 : 1
6	NaH	MTBE	Trace	—
7	NaH	DMF	38	>20 : 1
8	NaH	MeCN	36	>20 : 1
9	NaH	Toluene	33	>20 : 1
10	NaH	DCM	ND	—
11^d^	NaH	THF	87	>20 : 1
12^e^	NaH	THF	68	>20 : 1

a Reaction conditions: 1a (0.5 mmol, 1.0 equiv.), TMSCF_2_Br (2.0 equiv.), TBAB (5 mol%) and toluene (1.0 mL) in a sealed tube, 80 °C, 18 h, then 4a (1.0 mmol, 2.0 equiv.), TBAF (1.1 equiv.) and base (1.1 equiv.) in solvent (2.0 mL) was added. The reaction mixture was stirred at rt for 15 minutes.

b Isolated yields.

c The *Z*/*E* ratio was determined by ^1^H NMR.

d 0.4 equiv. of TBAF was used.

e 0.2 equiv. of TBAF was used.

With the optimized conditions for the steps A-C established, we next evaluated the substrate scope of *p*-QMs and substituted malonates. As shown in Table 2a, *p*-QMs containing various electron-withdrawing or electron-donating groups at the *para*-, *meta*- or *ortho*-position of the phenyl ring are suitable substrates for this transformation, producing 5ba–5ia in 51–82% yields. Substrates bearing multiple substituents on the phenyl ring were also amenable to this reaction, and products 5ja and 5ka were isolated in 69% and 71% yields, respectively. When the *tert*-butyl groups were replaced with isopropyl groups, 5la was obtained in 76% yield. When unsymmetrical *para*-quinone methide 1m was used, the corresponding product 5ma was isolated in 80% yield. Diethyl 2-methylmalonate containing a phenyl or allyl group at the C2-position is also a suitable substrates for this transformation, delivering 5ab and 5ac in 52% yields. When dimethyl 2-methylmalonate was used, the product 5ad could be obtained in 70% yield. All transformations proceeded with exceptional stereocontrol (>20 : 1 *Z*-selectivity), underscoring the robustness of this platform for constructing congested alkenes. To complete the C(sp^2^)-atomic transfer reaction, 5aa was further subjected to intermolecular functionalization with various nucleophiles, aiming to enrich the complexity and diversity of tetra-substituted alkenes (Table 2b, step D). Generally, in the presence of ^*t*^BuOK, various *O*-, *N*-, *P*-, and *C*-nucleophiles could participate in intermolecular nucleophilic vinylic substitution (S_N_V) reactions^18^ under mild conditions, giving 6a–6e in 70–86% yields with 13–20:1 *Z*-selectivity and 6f in 66% yield with > 20 : 1 *E*-selectivity. In addition, as a proof of concept, this reaction was run in one pot, and the corresponding product 6b was isolated in 60% yield with > 20 : 1 *Z*-selectivity (Table 2c).

**Table 2:** Single-carbon-atom transfer to *p-*QMs from TMSCF_2_Br with two different nucleophiles^a^^,^^b^^,^^c^

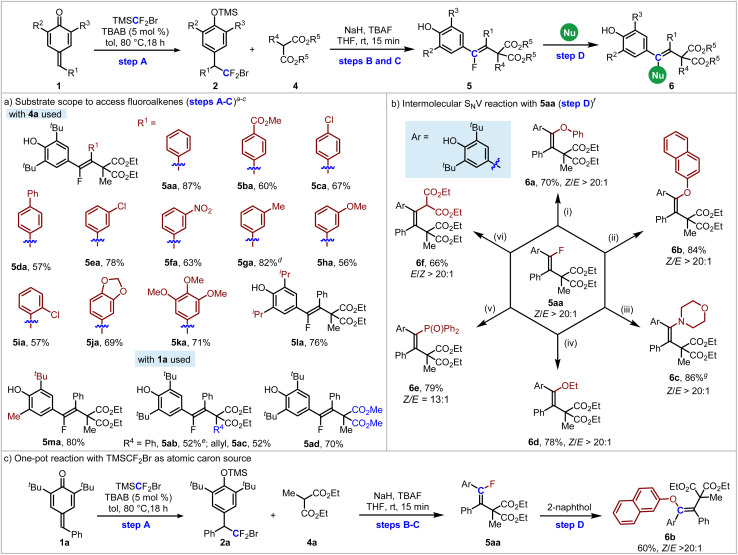

a 1 (0.5 mmol, 1.0 equiv.), TMSCF_2_Br (1.0 mmol, 2.0 equiv.), TBAB (5 mol%) and toluene (1.0 mL) in a sealed tube, 80 °C, 18 h, and then 4 (1.0 mmol, 2.0 equiv.), TBAF (0.4 equiv.) and NaH (3.0 equiv.) were added. The reaction mixture was stirred at rt for 15 minutes.

b Isolated yields.

c The *Z*/*E* ratio was determined by ^1^H NMR.

d The reaction time for steps B and C was further prolonged to 30 min.

e NaH (5.0 equiv.) was used.

f Nucleophiles (0.4 mmol, 2.0 equiv.), 5aa (0.2 mmol, 1.0 equiv.) and ^*t*^Bu (4.0 equiv.) in DCM were stirred at rt to 60 °C for 12 h.

g K_3_PO_4_ (0.4 mmol, 2.0 equiv.) and DMF were used.

**Scheme 2:**
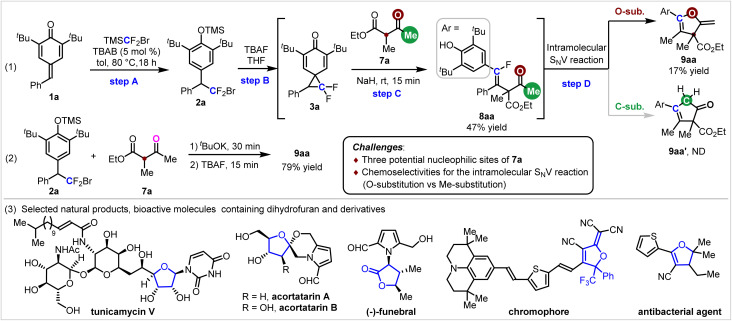
Single-carbon-atom transfer to *p*-QMs from TMSCF_2_Br with bisnucleophile 7a.

Encouraged by the above-mentioned single-carbon-atom transfer to *p*-QMs from TMSCF_2_Br with two different nucleophiles *via* stepwise functionalization, we next turned our attention to bisnucleophiles to examine the ability of this single-carbon-atom transfer reaction to construct cyclic compounds. First, ethyl 2-methyl-3-oxobutanoate 7a was tested. It's noteworthy that 7a contains three potential nucleophilic sites, and the chemoselectivity for the intramolecular S_N_V reaction of 8aa is another challenge. Under our previous standard conditions, the intramolecular O-substituted S_N_V product 9aa was successfully obtained in 17% yield, accompanied by the formation of 8aa in 47% yield, while the intramolecular C-substituted S_N_V product 9aa′ was not detected (Scheme 2(1)). At this stage, initiating condition screening with compound 2a as the precursor presented an enhanced efficiency profile for subsequent development. To our delight, the yield of 8aa could be further improved to 79% with ^*t*^BuOK as the base (Scheme 2(2), see the ESI† for details of the condition screening).

Dihydrofuran skeletons and derivatives are important skeletons widely found in natural products and bioactive molecules (Scheme 2(3)).^19^ For example, Tunicamycin V is an N-glycosylation inhibitor, and has been used as a pharmacological inducer of endoplasmic reticulum (ER) stress.^19*f*^ Acortatarins A and B are two spirocyclic alkaloids that significantly inhibit reactive oxygen species production in high-glucose-stimulated mesangial cells.^19*g*^ In this context, we further explored the substrate scope of *p*-QMs for the construction of 2-methylene-2,3-dihydrofurans *via* single-carbon-atom transfer reactions. As shown in Table 3, substrates 2 containing various electron-donating groups and electron-withdrawing groups at the *ortho*-, *meta*- and *para*-positions are all compatible with this reaction, delivering products 9ab–9am in 46–82% yields. When the R^1^ group was a heterocycle such as indole and thiophene, the corresponding products 9an and 9ao were obtained in moderate yields. Cyclopropyl (R^1^) substituted *p*-QM could also deliver 9ap in 42% yield accompanied by some unknown byproducts detected. Other alkyl groups such as cyclohexyl, phenylethyl could not give the corresponding products (not shown in Table 3). The unsymmetric *p*-QM derived adduct 2q generated 9aq smoothly in 64% yield. When the reaction was run on a 4 mmol scale, the product 9aa could still be isolated in 75% yield (1.34 g) without loss of efficiency.

**Table 3:** Substrate scope of *p-*QMs with bisnucleophile 7a^a^^,^^b^

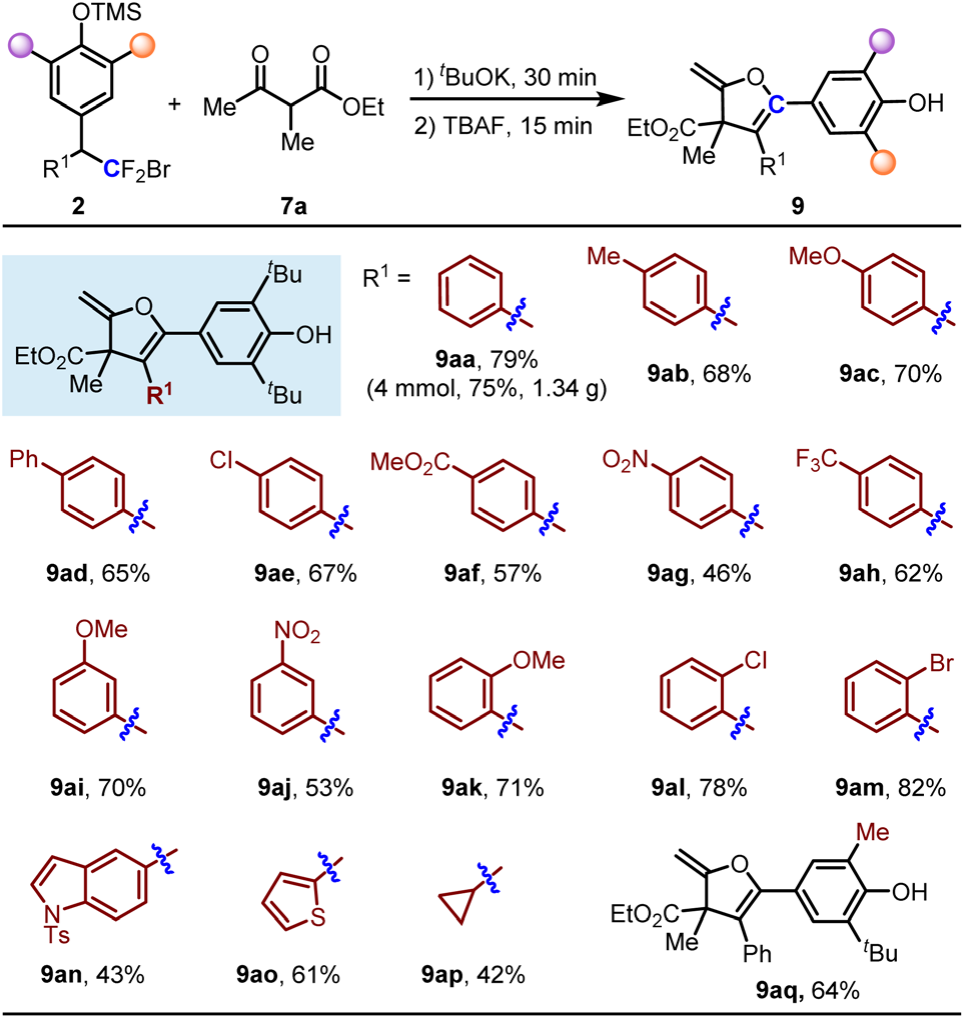

a Reaction conditions: 2 (0.2 mmol, 1.0 equiv.), 7a (0.4 mmol, 2.0 equiv.), TBAF (0.08 mmol, 0.4 equiv.) and ^*t*^BuOK (0.8 mmol, 4.0 equiv.) in DCM at 60 °C.

b Isolated yield.

Next, the scope of substituted acetoacetic esters was examined. As shown in Table 4, acetoacetic esters containing various alkyl groups at the C2-position were all compatible with this reaction, giving products 9ba–9da in 45–83% yields. Notably, the generation of some uncertain byproducts led to a moderate yield for product 9ca while the conversion of 2a was complete. Switching the R^3^ group from an ethyl group to a benzyl group could still deliver product 9ea in 70% yield. Intriguingly, introducing an alkyl or aryl group at the α-position of the ketone (R^1^ = Me or Ph) could also afford the cyclic products 9fa and 9ga in moderate yields with excellent *Z*-selectivities. Ethyl 2-oxocyclohexane-1-carboxylate 7h could also deliver the corresponding product 7ha in 42% yield. Next, electron-rich phenolic substrates 7i–7k were tested, generating 9ia–9ka in 44–67% yields. Again, a one pot reaction was performed to probe the efficacy of this single-carbon-atom transfer reaction, giving 9aa in 56% yield and 9ka in 64% yield.

**Table 4:** Substrate scope of bisnucleophile with 2a^a^^,^^b^

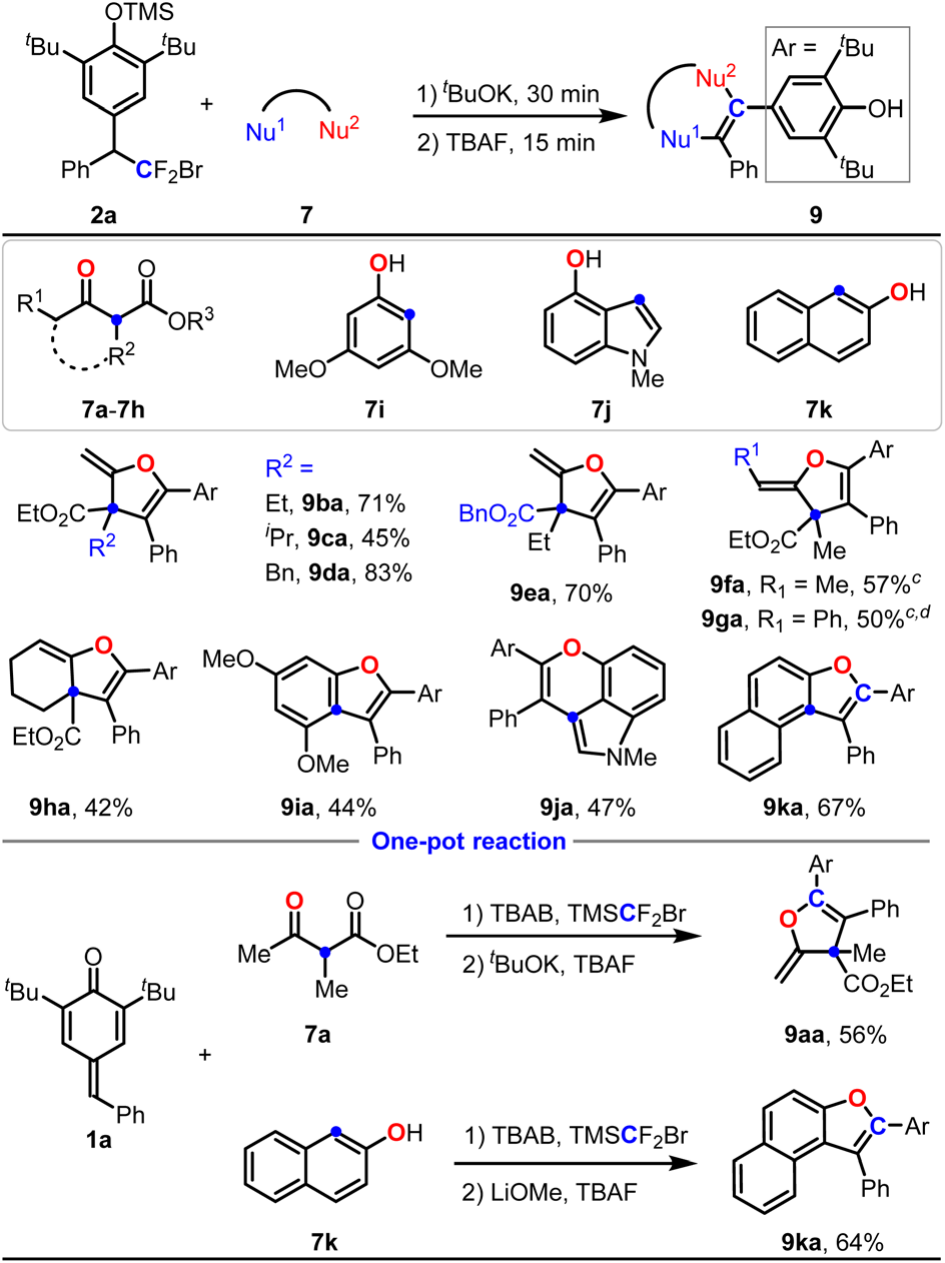

a Reaction conditions: 2a (0.2 mmol, 1.0 equiv.), 7 (0.4 mmol, 2.0 equiv.), TBAF (0.08 mmol, 0.4 equiv.) and ^*t*^BuOK (0.8 mmol, 4.0 equiv.) in DCM at 60 °C.

b Isolated yield.

c The *Z*/*E* ratio (>20 : 1) was determined by ^1^H NMR.

d Room temperature.

**Scheme 3:**
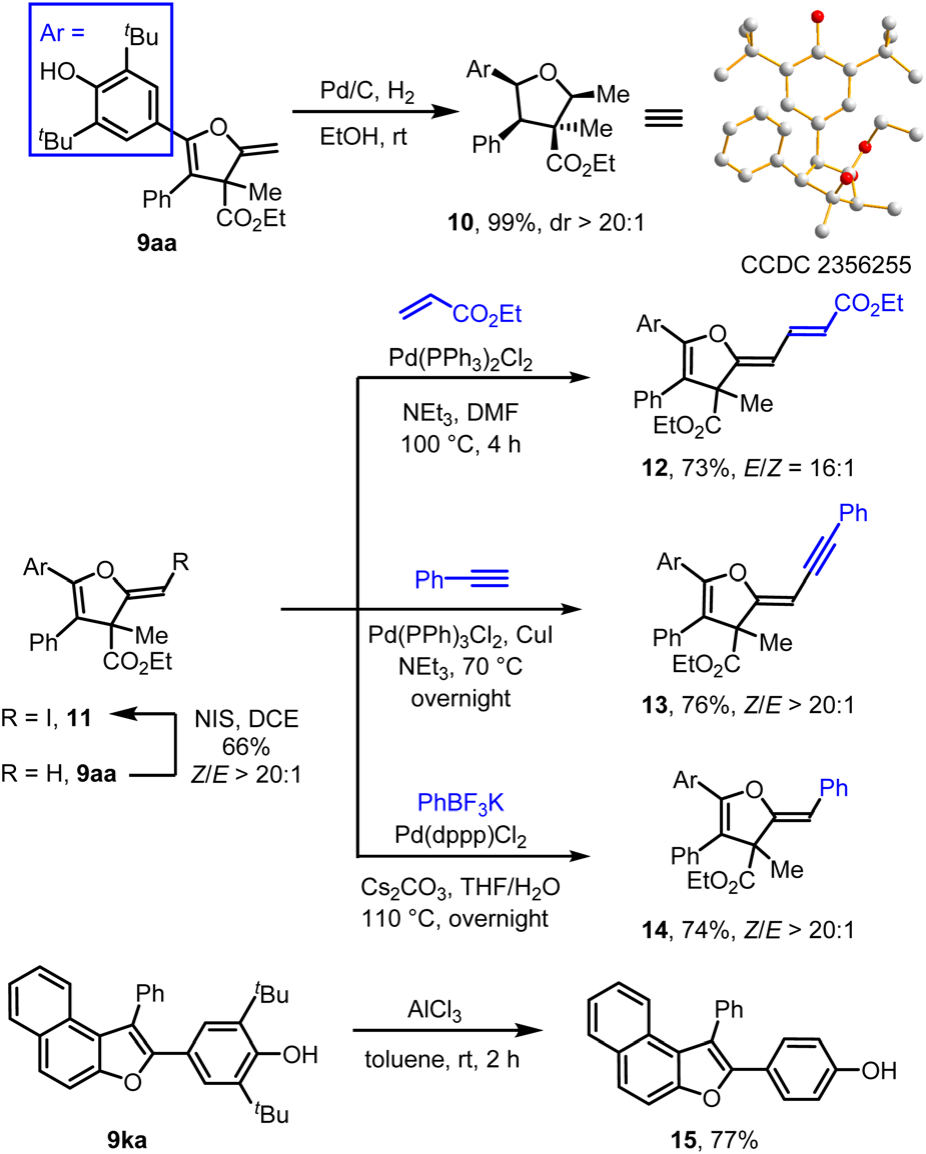
Transformations of the products.

To demonstrate the synthetic utility of this single-carbon-atom transfer reaction, a series of transformations of the products were conducted. As shown in Scheme 3, Pd/C-catalyzed hydrogenation of 2-methylene-2,3-dihydrofuran 9aa proceeded smoothly, and the product 10 containing four contiguous chiral centres was isolated in 99% yield with > 20 : 1 dr. The relative configuration of 10 was determined by X-ray analysis. In the presence of *N*-iodosuccinimide (NIS), the terminal alkene 9aa was iodinated to afford 11 in 66% yield with > 20 : 1 *Z*-selectivity. The vinyl iodide motif of compound 11 provided a handle for further functionalization, enriching the structural diversity of 2-methylene-2,3-dihydrofurans. For example, vinyl iodide could participate to a series of palladium-catalyzed stereoretentive cross-coupling reactions, including Heck, Sonogashira and Suzuki reaction, generating products 12–14 in 73–76% yields. The *tert*-butyl groups of 9ka were removed *via* AlCl_3_-catalyzed retro-Friedel–Crafts alkylation, yielding product 15 in 77% yield.

**Scheme 4:**
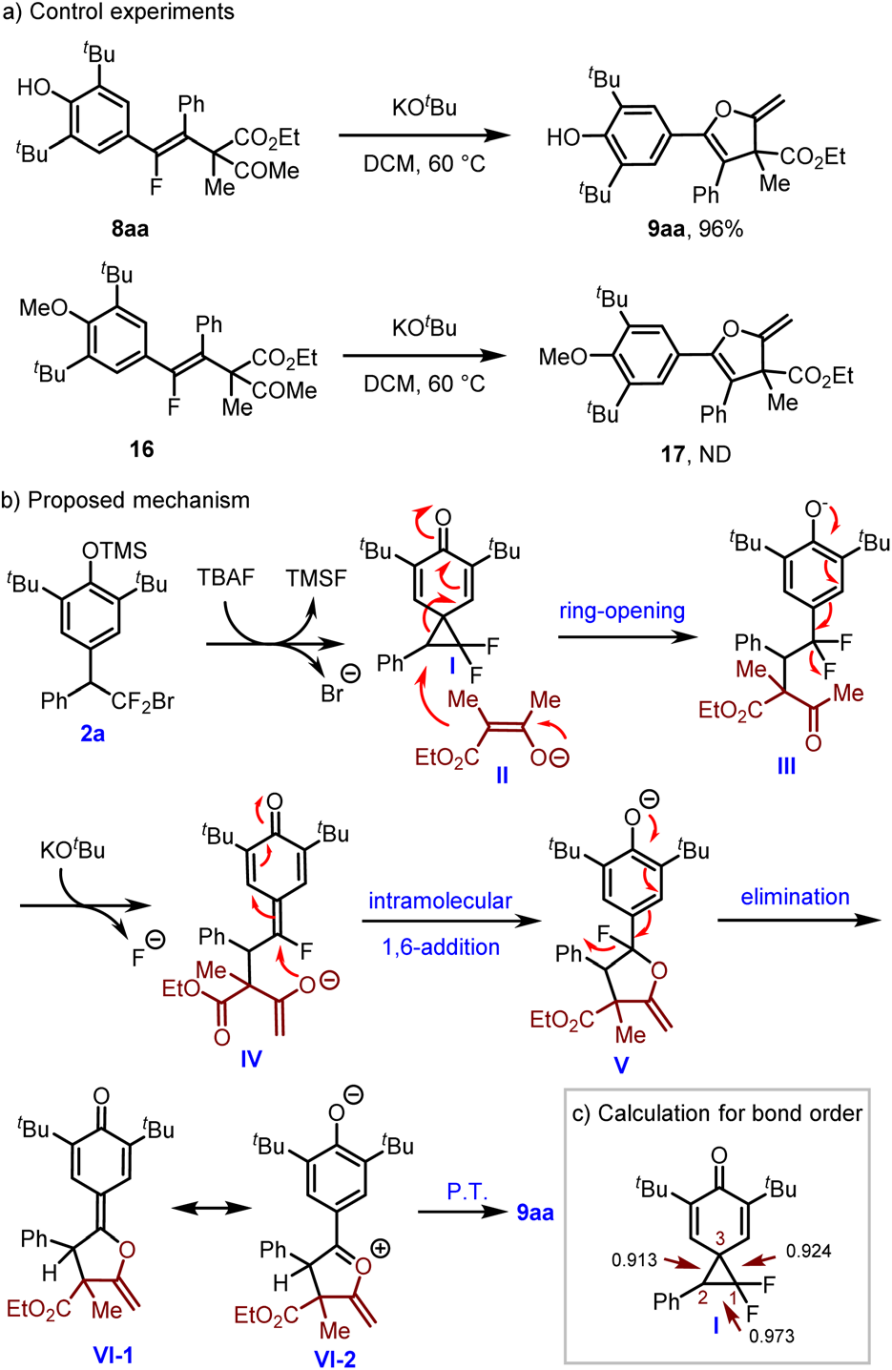
Control experiment and the proposed mechanism.

To gain insight into the reaction mechanism, a control experiment was conducted. As shown in Scheme 4(a), vinyl fluoride 8aa could be converted to 9aa smoothly in 96% yield in the presence of KO^*t*^Bu, while no cyclic product 17 could be detected when compound 16 was used. Collectively, these results suggest that 8aa serves as the intermediate for the cyclic process and the hydroxyl group is essential for the intramolecular S_N_V reaction. Combined with previous studies,^16*g*,17^ a plausible mechanism for the formation of 2-methylene-2,3-dihydrofuran 9aa was proposed as shown in Scheme 4(b). In the presence of TBAF, 2a could be transformed to spiro INT-I*via* sequential desilylation/cyclization. Then the enolate II generated from 7a and basic KO^*t*^Bu underwent regioselective nucleophilic substitution with INT-I to give INT-III. Dearomatization of INT-III*via* the elimination of fluoride and base promoted enolization generated INT-IV, which then underwent intramolecular 1,6-addition to yield INT-V. Phenolic anion or enol ether assisted elimination of the fluoride anion from INT-V would give INT-VI-1(2), and then proton transfer (P.T.) would produce the product 9aa. To probe the origin of the regioselectivity for the ring-opening step of INT-I, a computational study was conducted. As shown in Scheme 4(c), the computational study of INT-I showed that the C_2_–C_3_ bond possesses the smallest bond order in the cyclopropane unit.^20^ In other words, the C_2_–C_3_ bond is the weakest bond in the cyclopropane unit.

## Conclusions

In summary, we have developed a single-carbon atom transfer reaction of the alkene motifs of *p*-QMs. In the presence of a nucleophile such as malonic acid ester, tetra-substituted fluoroalkenes were constructed with excellent stereoselectivities. The fluoroalkenes could further react with another nucleophile *via* formal S_N_V reaction to generate highly congested tetra-substituted olefins with good to excellent stereoselectivities. When 1,3-bisnucleophiles were used, cyclic products such as 2-methylene-2,3-dihydrofurans were obtained with excellent chemo- and stereo-selectivities. Sequentially controllable functionalization of TMSCF_2_Br enables it to act as an atomic carbon equivalent. More importantly, this reaction features with mild conditions, a broad substrate scope and the ability to enable diversity-directed synthesis. Further applications of TMSCF_2_Br as an atomic carbon source are currently ongoing in our laboratory.

## Author contributions

H. Y. and S. G. directed the project and revised the manuscript. R. S. and P. Z. performed the experiments, and analysed and interpreted the results; they contributed equally to this work. Y. Y., J. Z., Q. C. and C. Y. were involved in the preparation of the substrates. X. L. performed the computational studies. C. Y. and A. L. revised the manuscript.

## Conflicts of interest

There are no conflicts to declare.

## Supplementary Material

SC-OLF-D5SC03234B-s001

SC-OLF-D5SC03234B-s002

## Data Availability

The data supporting this article have been included as part of the ESI,† including detailed experimental procedures and characterization data for new compounds. Crystallographic data have been deposited with the CCDC with deposition numbers 2356255 (10).
