# Supplementary material for: Single-carbon-atom transfer to para-quinone methides from TMSCF2Br
Source: Chem Sci. 2025 Jun 4;16(27):12324–31. doi: 10.1039/d5sc03234b (PMC12152741; doi:10.1039/d5sc03234b)
Supplement: SC-016-D5SC03234B-s001 [file SC-016-D5SC03234B-s001.pdf]

Supplementary Information (SI) for Chemical Science.
This journal is © The Royal Society of Chemistry 2025

Supporting Information

Single-Carbon-Atom Transfer to *para*-Quinone Methides from $TMSCF_2Br$

Ruikang Sun,[‡,a] Zhang Pei,[‡,a] Yong Yan,[a] Jie Zhu,[a] Qirui Chen,[a] Chi Yang,[b] Aijun Lin,[a] Xuanyi Li,[a] Shang Gao*,[a] and Hequan Yao*,[a]

[a]State Key Laboratory of Natural Medicines (SKLNM) and Department of Medicinal Chemistry, School of Pharmacy, China Pharmaceutical University, Nanjing 210009, P. R. China

[b]Anhui Provincial Joint Key Laboratory for Innovative Drug Research and Industry Integration, School of Chemistry and Materials Engineering, Fuyang Normal University, Fuyang 236037, P. R. China

*E-mail: gaoshang1990@cpu.edu.cn; hyao@cpu.edu.cn.

Content

1. General Information 2
2. General Procedure for the Synthesis of *p*-QMs Derivatives 3
3. General Procedure A for the Synthesis of Monofluoroalkenes **5** 5
4. Intermolecular S_NV Reaction with **5aa** 11
5. One-pot Reaction for the Synthesis of tetra-Substituted Alkene **6b** 15
6. Optimization of Reaction Parameters for the Synthesis of **9aa** 16
7. General Procedure B for the Synthesis of Product **9** 18
8. Gram-scale Reaction for the Synthesis of **9aa** 26
9. One-pot Reaction for the Synthesis of **9aa** and **9ka** 27
10. Transformations of the Products 29
11. Control Experiments 33
12. Calculation Studies 34
13. Crystal Structure of Product **10** 40
14. ^1H, ^{13}C and ^{19}F NMR Spectra of Title Compounds 41

1. General Information

Reagents and Solvents: PE refers to petroleum ether b. p. 60-90 °C, EA refers to ethyl acetate, and DCM refers to dichloromethane. All other starting materials and solvents were commercially available and were used without further purification unless otherwise stated.

Chromatography: Flash column chromatography was carried out using commercially available 200-300 mesh under pressure unless otherwise indicated. Gradient flash chromatography was conducted eluting with PE/EA or DCM/MeOH, they were listed as volume ratios.

Data collection: ^{1}H, ^{13}C and ^{19}F NMR spectra were collected on BRUKER AV-300 (300 MHz) spectrometer using $CDCl_3$ or DMSO-*d*$_6$ as solvent. Chemical shifts of ^{1}H NMR were recorded in parts per million (ppm, δ) relative to tetramethylsilane (δ = 0.00 ppm) with the solvent resonance as an internal standard ($CDCl_3$: δ = 7.26 ppm, DMSO-*d*$_6$: δ = 2.50 ppm). Data are reported as follows: chemical shift in ppm (δ), multiplicity (s = singlet, d = doublet, t = triplet, q = quartet, brs = broad singlet, m = multiplet), coupling constant (Hz), and integration. Chemical shifts of ^{13}C NMR were reported in ppm with the solvent as the internal standard ($CDCl_3$: δ = 77.16 ppm). High Resolution Mass measurement was performed on Agilent Q-TOF 65451 mass spectrometer with electron spray ionization (ESI) as the ion source. Melting point (m. p.) was measured on a microscopic melting point apparatus. X-ray diffraction analyses were carried out on a microcrystalline powder using a Rigaku Oxford Diffraction XtaLAB Synergy-S diffractometer using Mo radiation (λ = 0.71073 Å).

2. General Procedure for the Synthesis of *p*-QMs Derivatives

The *p*-QMs Derivatives **2a – 2q** were prepared according to the reported literature procedures.[1]

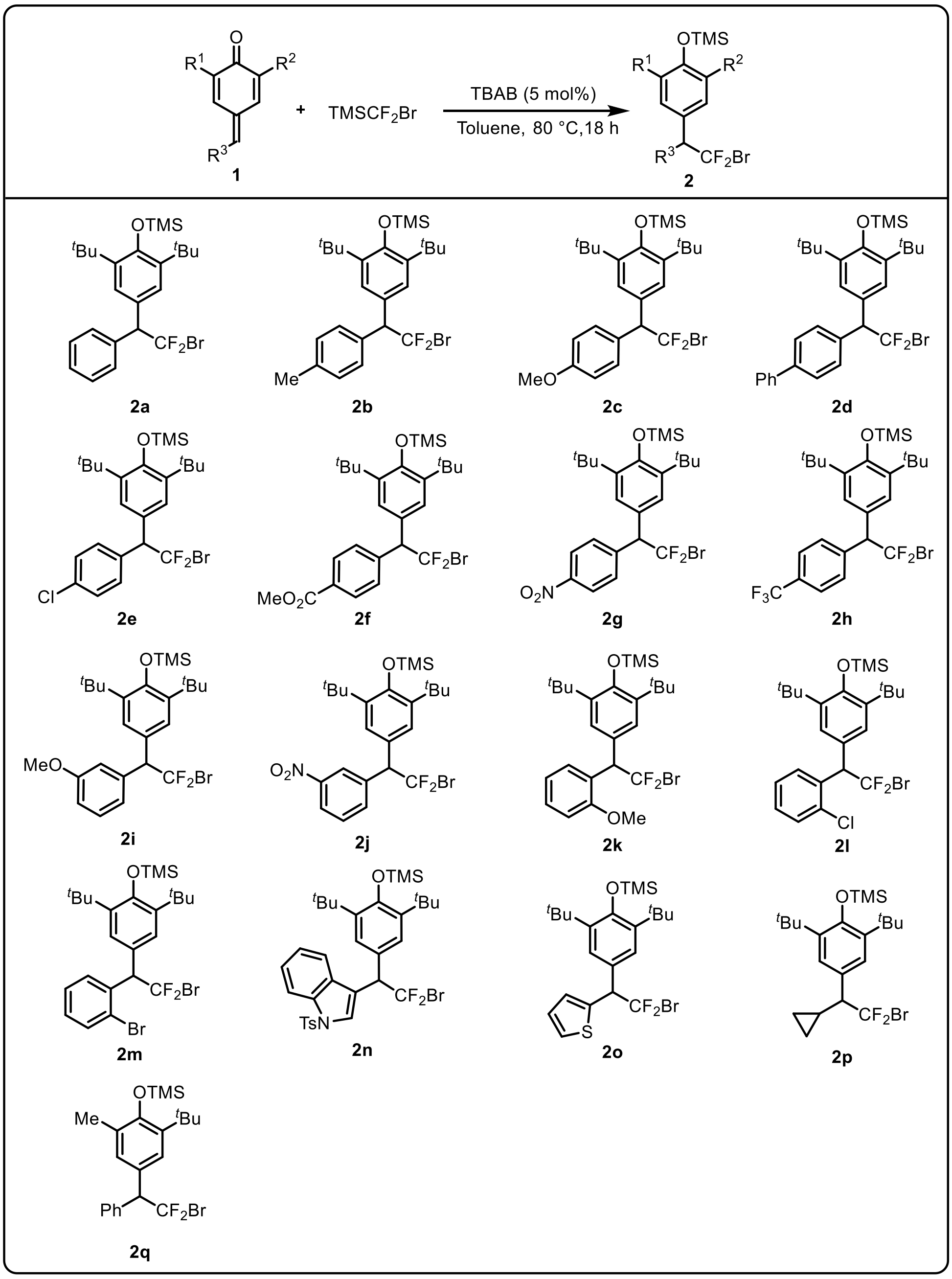

Figure S1. Synthesis of *p*-QMs Derivatives **2**

To an oven-dried 10 mL Schlenk tube equipped with a Teflon coated magnetic stir bar was added *p*-QMs (1.0 mmol, 1.0 equiv) and TBAB (16 mg, 0.05 mmol, 5 mol %). Then the Schlenk tube was evacuated and filled with argon for three times. After that, $TMSCF_2Br$ (406.2 mg, 2.0 mmol, 2.0 equiv) dissolved in toluene (2.0 mL) was added

[1] J. Zhu, M. Xu, B. Gong, A. Lin, S. Gao, *Org. Lett.* **2023**, *25*, 3271.

under argon atmosphere via a syringe. The reaction mixture was stirred at 80 °C in oil bath for 18 h. After completed consumption of starting material, the resulting mixture was then poured into ice water (5 mL), extracted with EA (3 × 5 mL). The organic layers were combined and dried over anhydrous $MgSO_4$. After removal of the solvent in vacuo, the crude material was purified by flash chromatography on silica gel (PE) to afford the desired product **2a** – **2q**.

3. General Procedure A for the Synthesis of Monofluoroalkenes 5

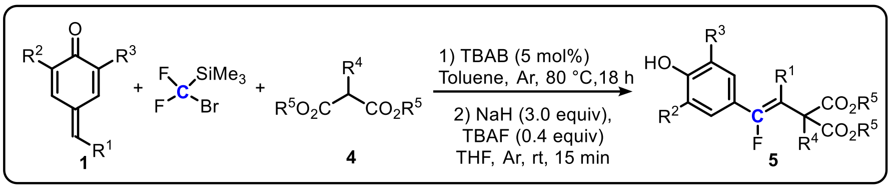

To an oven-dried 10-mL Schlenk tube equipped with a Teflon coated magnetic stir bar was added *p*-QMs **1** (0.5 mmol, 1.0 equiv) and TBAB (8.0 mg, 0.025 mmol, 5 mol %), then the Schlenk tube was evacuated and filled with argon for three times. After that, $TMSCF_2Br$ (203.1 mg, 1.0 mmol, 2.0 equiv) dissolved in toluene (1.0 mL) was added under argon atmosphere via a syringe. The reaction mixture was stirred at 80 °C in oil bath for 18 h to afford the toluene solution of crude intermediate.

To another oven-dried 10-mL Schlenk tube equipped with a Teflon coated magnetic stir bar was added 60% NaH (60.0 mg, 1.5 mmol, 3.0 equiv), then the Schlenk tube was evacuated and filled with argon for three times. After that, anhydrous THF (0.5 mL) and **4** (1.0 mmol, 2.0 equiv, dissolved in 0.5 mL THF) was added under argon atmosphere via a syringe. After stirred for 30 min at room temperature, the toluene solution of crude intermediate was added, and then TBAF (0.2 mmol, 0.4 equiv, 1 M solution in THF) was dropped slowly under argon atmosphere stirred for 15 min at room temperature. When the starting material was completely consumed, saturated solution of NH_4Cl (5 mL) was slowly added to quench the reaction. The reaction mixture was extracted with EA (3 × 10 mL). The organic layers were combined and dried over anhydrous $MgSO_4$. After removal of the solvent in vacuo, the residue was purified by flash chromatography on silica gel (PE/EA) to afford the corresponding product **5**.

diethyl (Z)-2-(2-(3,5-di-tert-butyl-4-hydroxyphenyl)-2-fluoro-1-phenylvinyl)-2-methylmalonate (5aa)

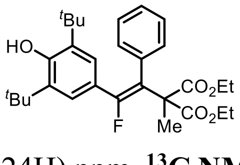

Prepared through general procedure A to give **5aa** in 187.0 mg, 75% yield, white solid, **m.p.** 120 – 122 °C, R_f = 0.45 (PE/EA = 10/1). **^{1}H NMR (300 MHz, $CDCl_3$)** δ 7.32 – 7.19 (m, 5H), 6.94 (s, 2H), 5.27 (s, 1H), 4.12 (q, J = 7.2 Hz, 4H), 1.65 (s, 3H), 1.22 – 1.17 (m, 24H) ppm. **^{13}C NMR (75 MHz, $CDCl_3$)** δ 171.2, 156.4 (d, ^1J = 252.4 Hz), 154.2, 136.6 (d, ^3J = 8.4 Hz), 135.0, 130.8 (d, ^4J = 3.1 Hz), 128.6, 127.6, 125.3 (d, ^3J = 7.6 Hz), 123.0 (d, ^2J = 28.2 Hz), 117.8 (d, ^2J = 19.5 Hz), 61.6, 58.8, 34.3, 30.1, 22.0 (d, ^4J = 3.1 Hz), 14.0 ppm. **^{19}F NMR (282 MHz, $CDCl_3$)** δ – 96.19 ppm. **HRMS (ESI)** *m*/*z* Calcd for $[C_{30}H_{39}FO_5 - H]^-$ 497.2709, found 497.2701.

diethyl (Z)-2-(2-(3,5-di-tert-butyl-4-hydroxyphenyl)-2-fluoro-1-(4-(methoxycarbonyl)phenyl)vinyl)-2-methylmalonate (5ba)

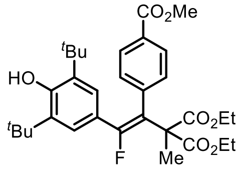

Prepared through general procedure A to give **5ba** in 167.0 mg, 60% yield, colorless oil, R_f = 0.2 (PE/EA = 10/1). **^{1}H NMR (300 MHz, $CDCl_3$)** δ 7.97 (d, J = 8.4 Hz, 2H), 7.30 (d, J = 8.3 Hz, 2H), 6.91 (s, 2H), 5.31 (s, 1H), 4.12 (q, J = 7.2 Hz, 4H), 3.91 (s, 3H), 1.67 (s, 3H), 1.22 – 1.18 (m, 24H) ppm. **^{13}C NMR (75 MHz, $CDCl_3$)** δ 170.5, 166.4, 156.5 (d, ^1J = 254.5 Hz), 154.1, 141.5 (d, ^3J = 8.5 Hz), 134.8, 130.6 (d, ^4J = 3.0 Hz), 129.4, 128.8, 125.0 (d, ^3J = 7.1 Hz), 122.2 (d, ^2J = 28.2 Hz), 116.7 (d, ^2J = 20.4 Hz), 61.4, 58.2, 51.9, 33.9, 29.6, 21.7 (d, ^4J = 2.6 Hz), 13.6 ppm. **^{19}F NMR (282 MHz, $CDCl_3$)** δ – 93.54 ppm. **HRMS (ESI)** *m/z* Calcd for $[C_{32}H_{41}FO_7 - H]^-$ 555.2764, found 555.2768.

diethyl (Z)-2-(1-(4-chlorophenyl)-2-(3,5-di-tert-butyl-4-hydroxyphenyl)-2-fluorovinyl)-2-methylmalonate (5ca)

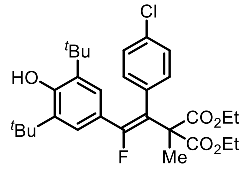

Prepared through general procedure A to give **5ca** in 177.9 mg, 67% yield, yellow solid, **m.p.** 78 – 80 °C, R_f = 0.35 (PE/EA = 10/1). **^{1}H NMR (300 MHz, $CDCl_3$)** δ 7.44 (d, J = 8.5 Hz, 2H), 7.32 (d, J = 8.4 Hz, 2H), 7.08 (s, 2H), 5.50 (s, 1H), 4.30 (q, J = 7.1 Hz, 4H), 1.83 (s, 3H), 1.41 (s, 18H), 1.37 (t, J = 7.1 Hz, 6H) ppm. **^{13}C NMR (75 MHz, $CDCl_3$)** δ 171.0, 156.8 (d, ^1J = 252.4 Hz), 154.4, 135.3, 135.2, 133.7, 132.3 (d, ^4J = 3.4 Hz), 128.8, 125.3 (d, ^3J = 7.3 Hz), 122.7 (d, ^2J = 28.0 Hz), 116.7 (d, ^2J = 20.0 Hz), 61.7, 58.6, 34.3, 30.0, 22.0 (d, ^4J = 3.3 Hz), 14.0 ppm. **^{19}F NMR (282 MHz, $CDCl_3$)** δ – 94.43 ppm. **HRMS (ESI)** *m/z* Calcd for $[C_{30}H_{38}ClFO_5 - H]^-$ 531.2319, found 531.2310.

diethyl (Z)-2-(1-([1,1'-biphenyl]-4-yl)-2-(3,5-di-tert-butyl-4-hydroxyphenyl)-2-fluorovinyl)-2-methylmalonate (5da)

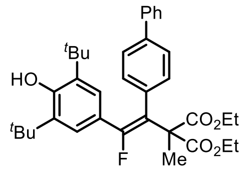

Prepared through general procedure A to give **5da** in 163.8 mg, 57% yield, white solid, **m.p.** 96 – 98 °C, R_f = 0.4 (PE/EA = 10/1). **^{1}H NMR (300 MHz, $CDCl_3$)** δ 7.18 – 7.14 (m, 4H), 7.09 – 7.04 (m, 2H), 7.00 – 6.91 (m, 3H), 6.62 (s, 2H), 4.92 (s, 1H), 3.78 (q, J = 7.1 Hz, 4H), 1.35 (s, 3H), 0.90 – 0.81 (m, 24H) ppm.**^{13}C NMR (75 MHz, $CDCl_3$)** δ 171.2, 156.4(d, ^1J = 251.3 Hz), 154.2, 141.0, 140.7, 135.7 (d, ^3J = 8.4 Hz), 135.1, 131.2 (d, ^4J = 2.9 Hz), 128.9, 127.4, 127.1, 125.2 (d, ^3J = 7.8 Hz), 123.0 (d, ^2J = 28.1 Hz), 117.4 (d, ^2J = 19.5 Hz), 61.7, 58.8, 34.3, 30.0, 22.1 (d, ^4J = 3.0 Hz), 14.0 ppm. **^{19}F NMR (282 MHz, $CDCl_3$)** δ – 96.35 ppm. **HRMS (ESI)** *m/z* Calcd for $[C_{36}H_{43}FO_5 - H]^-$ 573.3022, found 573.3029.

diethyl (Z)-2-(1-(3-chlorophenyl)-2-(3,5-di-tert-butyl-4-hydroxyphenyl)-2-fluorovinyl)-2-methylmalonate (5ea)

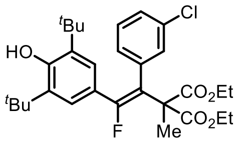

Prepared through general procedure A to give **5ea** in 207.9 mg, 78% yield, white solid, **m.p.** 101 – 103 °C, R_f = 0.45 (PE/EA = 10/1). **^{1}H NMR (300 MHz, CDCl$_3$)** δ 7.26 – 7.21 (m, 3H), 7.14 – 7.08 (m, 1H), 6.95 (s, 2H), 5.34 (s, 1H), 4.14 (q, J = 7.1 Hz, 4H), 1.67 (s, 3H), 1.29 – 1.19 (m, 24H) ppm. **^{13}C NMR (75 MHz, CDCl$_3$)** δ 170.9, 156.9 (d, ^1J = 252.4 Hz), 154.5, 138.6 (d, ^3J = 8.7 Hz), 135.2, 134.4, 130.9 (d, ^4J = 3.3 Hz), 130.0, 129.0 (d, ^4J = 3.1 Hz), 127.8, 125.3 (d, ^3J = 7.6 Hz), 122.5 (d, ^2J = 27.9 Hz), 116.6 (d, ^2J = 20.3 Hz), 61.8, 58.7, 34.3, 30.0, 22.0 (d, ^4J = 3.1 Hz), 14.0 ppm. **^{19}F NMR (282 MHz, CDCl$_3$)** δ – 94.52 ppm. **HRMS (ESI)** *m*/*z* Calcd for $[C_{30}H_{38}ClFO_5 - H]^-$ 531.2319, found 531.2311.

diethyl (Z)-2-(2-(3,5-di-tert-butyl-4-hydroxyphenyl)-2-fluoro-1-(3-nitrophenyl)vinyl)-2-methylmalonate (5fa)

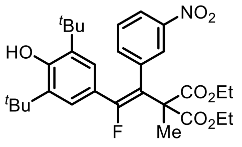

Prepared through general procedure A to give **5fa** in 171.6 mg, 63% yield, white solid, **m.p.** 108 – 110 °C, R_f = 0.3 (PE/EA = 10/1). **^{1}H NMR (300 MHz, CDCl$_3$)** δ 8.15 – 8.10 (m, 2H), 7.60 – 7.56 (m, 1H), 7.49 (t, J = 7.8 Hz, 1H), 6.89 (s, 2H), 5.36 (s, 1H), 4.15 (q, J = 7.2 Hz, 4H), 1.72 (s, 3H), 1.26 – 1.18 (s, 24H) ppm. **^{13}C NMR (75 MHz, CDCl$_3$)** δ 170.7, 158.0 (d, ^1J = 255.3 Hz), 154.7, 148.3, 138.9 (d, ^3J = 9.0 Hz), 137.3 (d, ^4J = 3.0 Hz), 135.5, 129.5, 126.0 (d, ^4J = 3.3 Hz), 125.6 (d, ^3J = 6.6 Hz), 122.5, 122.2 (d, ^2J = 27.8 Hz), 116.1 (d, ^2J = 21.2 Hz), 62.0, 58.6, 34.3, 30.0, 22.2 (d, ^4J = 3.6 Hz), 14.0 ppm. **^{19}F NMR (282 MHz, CDCl$_3$)** δ – 90.92 ppm. **HRMS (ESI)** *m*/*z* Calcd for $[C_{30}H_{38}FNO_7 - H]^-$ 542.2560, found 542.2572.

diethyl (Z)-2-(2-(3,5-di-tert-butyl-4-hydroxyphenyl)-2-fluoro-1-(m-tolyl)vinyl)-2-methylmalonate (5ga)

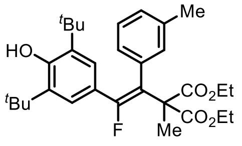

Prepared through general procedure A to give **5ga** in 130.3 mg, 51% yield, yellow solid, **m.p.** 110 – 112 °C, R_f = 0.4 (PE/EA = 10/1). **^{1}H NMR (300 MHz, CDCl$_3$)** δ 7.26 – 7.15 (m, 1H), 7.07 – 6.96 (m, 5H), 5.28 (s, 1H), 4.13 (q, J = 7.1 Hz, 4H), 2.29 (s, 3H), 1.63 (s, 3H), 1.28 – 1.18 (m, 24H) ppm. **^{13}C NMR (75 MHz, CDCl$_3$)** δ 171.3, 156.1 (d, ^1J = 250.4 Hz), 154.1, 138.1, 136.3 (d, ^3J = 8.3 Hz), 134.9, 131.3 (d, ^4J = 3.0 Hz), 128.6, 128.4, 127.7 (d, ^4J = 3.0 Hz), 125.1 (d, ^3J = 7.9 Hz), 123.0 (d, ^2J = 28.3 Hz), 117.7 (d, ^2J = 19.2 Hz), 61.6, 58.7, 34.2, 30.0, 22.0 (d, ^4J = 2.8 Hz), 21.3, 14.0 ppm. **^{19}F NMR (282 MHz, CDCl$_3$)** δ – 97.08 ppm. **HRMS (ESI)** *m*/*z* Calcd for $[C_{31}H_{41}FO_5 - H]^-$ 511.2865, found 511.2867.

diethyl (Z)-2-(2-(3,5-di-tert-butyl-4-hydroxyphenyl)-2-fluoro-1-(3-methoxyphenyl)vinyl)-2-methylmalonate (5ha)

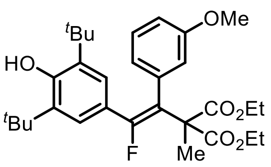

Prepared through general procedure A to give **5ha** in 148.6 mg, 56% yield, white solid, **m.p.** 88 – 90 °C, R_f = 0.4 (PE/EA = 10/1). **^{1}H NMR (300 MHz, CDCl$_3$)** δ 7.21 (t, *J* = 7.9 Hz, 1H), 6.99 (s, 2H), 6.82 – 6.74 (m, 3H), 5.30 (s, 1H), 4.15 (q, *J* = 7.1 Hz, 4H), 3.71 (s, 3H), 1.64 (s, 3H), 1.24 – 1.19 (m, 24H) ppm. **^{13}C NMR (75 MHz, CDCl$_3$)** δ 171.2, 159.8, 156.3 (d, ^1J = 251.0 Hz), 154.2, 137.8 (d, ^3J = 8.4 Hz), 135.0, 129.6, 125.1 (d, ^3J = 7.5 Hz), 123.1 (d, ^4J = 3.0 Hz), 123.0 (d, ^2J = 28.1 Hz), 117.5 (d, ^2J = 19.5 Hz), 116.3 (d, ^4J = 3.1 Hz), 113.4, 61.6, 58.6, 55.3, 34.2, 30.0, 22.0 (d, ^4J = 2.7 Hz), 13.9 ppm. **^{19}F NMR (282 MHz, CDCl$_3$)** δ – 96.35 ppm. **HRMS (ESI)** *m/z* Calcd for $[C_{31}H_{41}FO_6 – H]^-$ 527.2814, found 527.2819.

diethyl (E)-2-(1-(2-chlorophenyl)-2-(3,5-di-tert-butyl-4-hydroxyphenyl)-2-fluorovinyl)-2-methylmalonate (5ia)

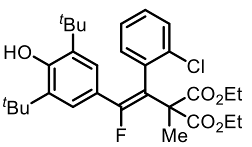

Prepared through general procedure A to give **5ia** in 151.9 mg, 57% yield, white solid, **m.p.** 96 – 98 °C, R_f = 0.35 (PE/EA = 10/1). **^{1}H NMR (300 MHz, CDCl$_3$)** δ 7.38 – 7.35 (m, 1H), 7.28 – 7.17 (m, 3H), 6.99 (s, 2H), 5.29 (s, 1H), 4.26 (q, *J* = 7.0 Hz, 2H), 4.05 – 3.97 (m, 2H), 1.66 (s, 3H), 1.30 (t, *J* = 7.1 Hz, 3H), 1.25 (s, 18H), 1.15 (t, *J* = 7.1 Hz, 3H) ppm. **^{13}C NMR (75 MHz, CDCl$_3$)** δ 171.2, 157.6 (d, ^1J = 255.0 Hz), 154.5, 136.1 (d, ^4J = 2.9 Hz), 136.0 (d, ^3J = 9.2 Hz), 135.2, 132.8 (d, ^4J = 3.1 Hz), 129.7, 129.2, 127.0, 124.7 (d, ^3J = 7.3 Hz), 122.9 (d, ^2J = 28.3 Hz), 114.8 (d, ^2J = 22.2 Hz), 61.7 (d, ^4J = 4.5 Hz), 58.6, 34.3, 30.1, 21.5, 14.1, 13.9 ppm. **^{19}F NMR (282 MHz, CDCl$_3$)** δ – 93.06 ppm. **HRMS (ESI)** *m/z* Calcd for $[C_{30}H_{38}ClFO_5 – H]^-$ 531.2319, found 531.2312.

diethyl (Z)-2-(1-(benzo[d][1,3]dioxol-5-yl)-2-(3,5-di-tert-butyl-4-hydroxyphenyl)-2-fluorovinyl)-2-methylmalonate (5ja)

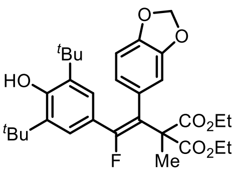

Prepared through general procedure A to give **5ja** in 188.6 mg, 69% yield, colorless oil, R_f = 0.25 (PE/EA = 10/1). **^{1}H NMR (300 MHz, CDCl$_3$)** δ 7.02 (s, 2H), 6.78 (d, *J* = 8.3 Hz, 1H), 6.72 – 6.70 (m, 2H), 5.93 (s, 2H), 5.34 (s, 1H), 4.18 (q, *J* = 7.1 Hz, 4H), 1.66 (s, 3H), 1.30 – 1.23 (m, 24H) ppm. **^{13}C NMR (75 MHz, CDCl$_3$)** δ 171.2, 156.5 (d, ^1J = 251.1 Hz), 154.2, 147.7, 147.1, 135.0, 130.0 (d, ^3J = 8.9 Hz), 125.1 (d, ^3J = 7.9 Hz), 124.3 (d, ^4J = 3.3 Hz), 123.0 (d, ^2J = 28.1 Hz), 117.1 (d, ^2J = 20.2 Hz), 111.4 (d, ^4J = 3.4 Hz), 108.6, 101.0, 61.6, 58.7, 34.3, 30.1, 22.0 (d, ^4J = 2.9 Hz), 14.0 ppm. **^{19}F NMR (282 MHz, CDCl$_3$)** δ – 96.04 ppm. **HRMS (ESI)** *m/z* Calcd for $[C_{31}H_{39}FO_7 – H]^-$ 541.2607, found 541.2615.

diethyl (Z)-2-(2-(3,5-di-tert-butyl-4-hydroxyphenyl)-2-fluoro-1-(2,3,4-trimethoxyphenyl)vinyl)-2-methylmalonate (5ka)

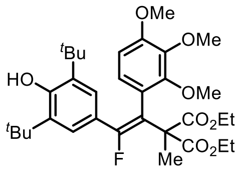

Prepared through general procedure A to give **5ka** in 209.1 mg, 71% yield, colorless oil, R_f = 0.2 (PE/EA = 5/1). **^{1}H NMR (300 MHz, CDCl$_3$)** δ 7.03 (s, 2H), 6.42 (s, 2H), 5.31 (s, 1H), 4.16 (q, J = 7.1 Hz, 4H), 3.82 (s, 3H), 3.74 (s, 6H), 1.67 (s, 3H), 1.27 (s, 18H), 1.22 (t, J = 7.2 Hz, 6H) ppm. **^{13}C NMR (75 MHz, CDCl$_3$)** δ 171.1, 156.4 (d, ^1J = 251.7 Hz), 154.2, 153.4, 137.5, 135.1, 132.1 (d, ^3J = 8.8 Hz), 125.0 (d, ^3J = 7.6 Hz), 123.0 (d, ^2J = 28.2 Hz), 117.6 (d, ^2J = 20.0 Hz), 107.9 (d, ^4J = 3.2 Hz), 61.6, 60.8, 58.8, 56.2, 34.3, 30.1, 22.0 (d, ^4J = 3.0 Hz), 14.0 ppm. **^{19}F NMR (282 MHz, CDCl$_3$)** δ – 96.50 ppm. **HRMS (ESI)** *m/z* Calcd for [$C_{33}H_{45}FO_8$ – H]$^-$ 587.3026, found 587.3019.

diethyl (Z)-2-(2-fluoro-2-(4-hydroxy-3,5-diisopropylphenyl)-1-phenylvinyl)-2-methylmalonate (5la)

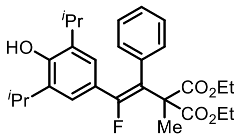

Prepared through general procedure A to give **5la** in 180.0 mg, 76% yield, colorless oil, R_f = 0.35 (PE/EA = 10/1). **^{1}H NMR (300 MHz, CDCl$_3$)** δ 7.29 – 7.19 (m, 5H), 6.78 (s, 2H), 5.08 (s, 1H), 4.12 (q, J = 7.1 Hz, 4H), 3.05 – 2.91 (m, 2H), 1.66 (s, 3H), 1.19 (t, J = 7.1 Hz, 6H), 1.00 (d, J = 6.9 Hz, 12H) ppm. **^{13}C NMR (75 MHz, CDCl$_3$)** δ 171.3, 156.3 (d, ^1J = 251.3 Hz), 150.4, 136.6 (d, ^3J = 8.5 Hz), 132.9, 130.9 (d, ^4J = 3.2 Hz), 128.6, 127.7, 124.1 (d, ^2J = 28.7 Hz), 123.7 (d, ^3J = 7.3 Hz), 117.9 (d, ^2J = 19.1 Hz), 61.7, 58.8, 26.9, 22.5, 22.0 (d, ^4J = 2.4 Hz), 14.0 ppm. **^{19}F NMR (282 MHz, CDCl$_3$)** δ – 96.24 ppm. **HRMS (ESI)** *m/z* Calcd for [$C_{28}H_{35}FO_5$ – H]$^-$ 469.2396, found 469.2389.

diethyl (Z)-2-(2-(3-(tert-butyl)-4-hydroxy-5-methylphenyl)-2-fluoro-1-phenylvinyl)-2-methylmalonate (5ma)

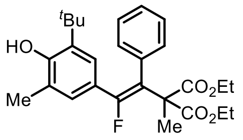

Prepared through general procedure A to give **5ma** in 182.0 mg, 80% yield, colorless oil, R_f = 0.25 (PE/EA = 10/1). **^{1}H NMR (300 MHz, CDCl$_3$)** δ 7.30 – 7.14 (m, 5H), 6.90 (s, 1H), 6.71 (s, 1H), 5.27 (s, 1H), 4.11 (q, J = 7.1 Hz, 4H), 2.09 (s, 3H), 1.65 (s, 3H), 1.18 (t, J = 7.1 Hz, 6H), 1.10 (s, 9H) ppm. **^{13}C NMR (75 MHz, CDCl$_3$)** δ 171.3, 156.2 (d, ^1J = 251.2 Hz), 153.2, 136.4 (d, ^3J = 8.2 Hz), 134.7, 130.7 (d, ^4J = 3.0 Hz), 128.5, 127.8 (d, ^3J = 7.3 Hz), 127.6, 126.1 (d, ^3J = 7.0 Hz), 123.3 (d, ^2J = 28.7 Hz), 122.6, 117.8 (d, ^2J = 19.1 Hz), 61.7, 58.7, 34.4, 29.3, 21.9 (d, ^4J = 3.0 Hz), 16.0, 13.9 ppm. **^{19}F NMR (282 MHz, CDCl$_3$)** δ – 95.38 ppm. **HRMS (ESI)** *m/z* Calcd for [$C_{27}H_{33}FO_5$ – H]$^-$ 455.2239, found 455.2227.

diethyl (Z)-2-(2-(3,5-di-tert-butyl-4-hydroxyphenyl)-2-fluoro-1-phenylvinyl)-2-phenylmalonate (5ab)

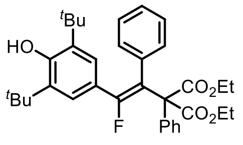

Prepared through general procedure A to give **5ab** in 145.8 mg, 52% yield, yellow solid, **m.p.** 132 – 134 °C, R_f = 0.4 (PE/EA = 10/1). **^{1}H NMR (300 MHz, CDCl$_3$)** δ 7.63 – 7.59 (m, 2H), 7.37 – 7.22 (m, 8H), 7.00 (s, 2H), 5.32 (s, 1H), 4.10 – 3.94 (m, 4H), 1.23 (s,

18H), 1.06 (t, J = 7.1 Hz, 6H) ppm. **^{13}C NMR (75 MHz, $CDCl_3$)** δ 169.8, 156.5 (d, ^{1}J = 251.2 Hz), 154.4, 137.1 (d, ^{3}J = 7.9 Hz), 136.5, 135.1, 130.9 (d, ^{4}J = 3.0 Hz), 129.3, 128.6, 127.7, 127.6, 127.4, 125.3 (d, ^{3}J = 7.9 Hz), 122.7 (d, ^{2}J = 27.7 Hz), 116.1 (d, ^{2}J = 19.7 Hz), 62.0, 34.3, 30.0, 13.8 ppm. **^{19}F NMR (282 MHz, $CDCl_3$)** δ – 90.19 ppm. **HRMS (ESI)** *m/z* Calcd for $[C_{35}H_{41}FO_5 - H]^-$ 559.2865, found 559.2870.

diethyl (Z)-2-allyl-2-(2-(3,5-di-tert-butyl-4-hydroxyphenyl)-2-fluoro-1-phenylvinyl) malonate (5ac)

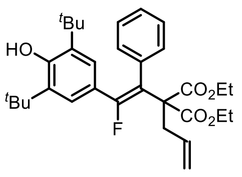

Prepared through general procedure A to give **5ac** in 134.0 mg, 52% yield, white solid, **m.p.** 114 – 116 °C, R_f = 0.45 (PE/EA = 10/1). **^{1}H NMR (300 MHz, $CDCl_3$)** δ 7.22 – 7.11 (m, 5H), 6.84 (s, 2H), 6.05 – 5.91 (m, 1H), 5.20 (s, 1H), 5.03 – 4.95 (m, 2H), 4.02 (q, J = 7.1 Hz, 4H), 2.71 (d, J = 7.2 Hz, 2H), 1.14 – 1.07 (m, 24H) ppm. **^{13}C NMR (75 MHz, $CDCl_3$)** δ 170.2, 156.5 (d, ^{1}J = 252.0 Hz), 154.2, 136.7 (d, ^{3}J = 8.3 Hz), 135.0, 134.6, 130.9 (d, ^{4}J = 2.9 Hz), 128.5, 127.5, 125.3 (d, ^{3}J = 7.3 Hz), 123.0 (d, ^{2}J = 28.2 Hz), 118.2, 116.4 (d, ^{2}J = 19.3 Hz), 62.5, 61.4, 39.9 (d, ^{4}J = 2.8 Hz), 34.2, 30.0, 13.9 ppm. **^{19}F NMR (282 MHz, $CDCl_3$)** δ – 94.24 ppm. **HRMS (ESI)** *m/z* Calcd for $[C_{32}H_{41}FO_5 - H]^-$ 523.2865, found 523.2858.

dimethyl (Z)-2-(2-(3,5-di-tert-butyl-4-hydroxyphenyl)-2-fluoro-1-phenylvinyl)-2-methylmalonate (5ad)

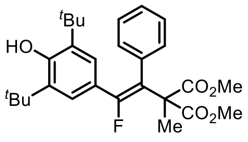

Prepared through general procedure A to give **5ad** in 164.7 mg, 70% yield, white solid, **m.p.** 140 – 142 °C, R_f = 0.45 (PE/EA = 10/1). **^{1}H NMR (300 MHz, $CDCl_3$)** δ 7.26 – 7.16 (m, 3H), 7.13 – 7.10 (m, 2H), 6.88 (s, 2H), 5.22 (s, 1H), 3.58 (s, 6H), 1.54 (s, 3H), 1.13 (s, 18H) ppm. **^{13}C NMR (75 MHz, $CDCl_3$)** δ 171.7 (d, ^{4}J = 1.0 Hz), 156.4 (d, ^{1}J = 250.3 Hz), 154.3, 136.3 (d, ^{3}J = 8.4 Hz), 135.0 (d, ^{4}J = 1.2 Hz), 130.7 (d, ^{4}J = 3.0 Hz), 128.7, 127.8, 125.2 (d, ^{3}J = 8.0 Hz), 122.7 (d, ^{2}J = 28.2 Hz), 117.3 (d, ^{2}J = 19.3 Hz), 58.6 (d, ^{3}J = 1.1 Hz), 52.8, 34.3, 30.0, 22.1 (d, ^{4}J = 2.6 Hz) ppm. **^{19}F NMR (282 MHz, $CDCl_3$)** δ – 97.51 ppm. **HRMS (ESI)** *m/z* Calcd for $[C_{28}H_{35}FO_5 - H]^-$ 469.2396, found 469.2388.

methyl (Z)-2-acetyl-4-(3,5-di-tert-butyl-4-hydroxyphenyl)-4-fluoro-2-methyl-3-phenylbut-3-enoate (8aa)

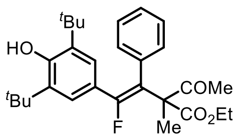

Prepared through general procedure A to give **8aa** in 110.1 mg, 47% yield, yellow solid, **m.p.** 120 – 122 °C, R_f = 0.45 (PE/EA = 10/1). **^{1}H NMR (300 MHz, $CDCl_3$)** δ 7.34 – 7.26 (m, 3H), 7.18 – 7.16 (m, 2H), 6.95 (s, 2H), 5.33 (s, 1H), 4.09 – 3.98 (m, 2H), 2.48 (s, 3H), 1.48 (s, 3H), 1.23 (s, 18H), 1.14 (t, J = 7.1 Hz, 3H) ppm. **^{13}C NMR (75 MHz, $CDCl_3$)** δ 205.0, 171.5, 156.4 (d, ^{1}J = 249.7 Hz), 154.5, 136.3 (d, ^{3}J = 8.9 Hz), 135.2, 130.5 (d, ^{4}J = 2.0 Hz), 128.9, 127.9, 125.2 (d, ^{3}J = 7.8 Hz), 122.5 (d, ^{2}J = 27.9 Hz), 118.1 (d, ^{2}J = 20.0 Hz), 64.0, 61.5, 34.3, 30.1, 27.2 (d, ^{4}J = 4.7 Hz), 20.9, 13.9 ppm. **^{19}F NMR (282 MHz, $CDCl_3$)** δ – 96.01 ppm. **HRMS (ESI)** *m/z* Calcd for $[C_{29}H_{37}FO_4 + H]^+$ 469.2749, found 469.2760.

4. Intermolecular S$_N$V Reaction with 5aa

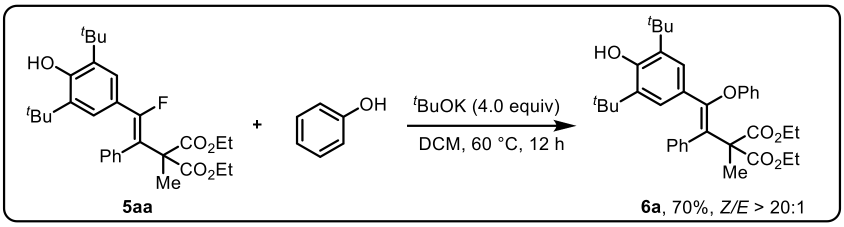

diethyl (Z)-2-(2-(3,5-di-tert-butyl-4-hydroxyphenyl)-2-phenoxy-1-phenylvinyl)-2-methylmalonate (6a) To an oven-dried 10 mL Schlenk tube equipped with a Teflon coated magnetic stir bar was added ^{t}BuOK (89.8 mg, 0.8 mmol, 4.0 equiv), **5aa** (99.7 mg, 0.2 mmol, 1.0 equiv) and phenol (37.6 mg, 0.4 mmol, 2.0 equiv) then the Schlenk tube was evacuated and filled with argon for three times. After that, anhydrous DCM (2 mL) was added under argon atmosphere via a syringe under argon atmosphere stirred for 12 h at 60 °C. After the starting material was completely consumed, ice water (5 mL) was slowly added to quench the reaction. The reaction mixture was extracted with DCM (3 × 10 mL). The organic layers were combined and dried over anhydrous $MgSO_4$. After removal of the solvent in vacuo, the residue was purified by flash chromatography on silica gel (PE/EA) to afford the corresponding product **6a** as a white solid (80.1 mg, 70%, *Z/E* > 20:1). **m.p.** 134 – 135 °C, R_f = 0.3 (PE/EA = 20/1). **^{1}H NMR (300 MHz, $CDCl_3$)** δ 7.22 – 7.08 (m, 7H), 6.86 – 6.78 (m, 5H), 5.01 (s, 1H), 4.10 – 3.90 (m, 4H), 1.61 (s, 3H), 1.13 – 1.09 (m, 24H) ppm. **^{13}C NMR (75 MHz, $CDCl_3$)** δ 171.7, 156.2, 153.0, 150.0, 138.6, 134.6, 131.2, 128.8, 128.1, 127.3, 126.8, 125.1, 125.0, 121.7, 118.2, 61.4, 59.0, 34.1, 30.2, 22.4, 14.0 ppm. **HRMS (ESI)** *m*/*z* Calcd for $[C_{36}H_{44}O_6 + H]^+$ 573.3211, found 573.3224.

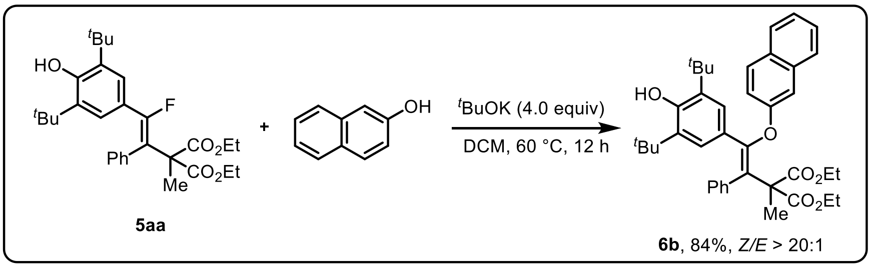

diethyl (Z)-2-(2-(3,5-di-tert-butyl-4-hydroxyphenyl)-2-(naphthalen-2-yloxy)-1-phenylvinyl)-2-methylmalonate (6b) To an oven-dried 10 mL Schlenk tube equipped with a Teflon coated magnetic stir bar was added ^{t}BuOK (89.8 mg, 0.8 mmol, 4.0 equiv), **5aa** (99.7 mg, 0.2 mmol, 1.0 equiv) and 2-hydroxynaphthalene (57.6 mg, 0.4 mmol, 2.0 equiv) then the Schlenk tube was evacuated and filled with argon for three times. After that, anhydrous DCM (2 mL) was added under argon atmosphere via a syringe under argon atmosphere stirred for 12 h at 60 °C. After the starting material was completely consumed, ice water (5 mL) was slowly added to quench the reaction. The reaction mixture was extracted with DCM (3 × 10 mL). The organic layers were combined and dried over anhydrous $MgSO_4$. After removal of the solvent in vacuo, the residue was purified by flash chromatography on silica gel (PE/EA) to afford the corresponding

product **6b** as a white solid (104.4 mg, 84% yield, *Z/E* > 20:1). **m.p.** 116 – 118 °C, R_f = 0.3 (PE/EA = 20/1). The regioselectivity of this reaction (C1 vs OH) was determined by the proton NMR with "Heavy Water Exchange NMR Experiment". **^{1}H NMR (300 MHz, $CDCl_3$)** δ 7.70 – 7.57 (m, 3H), 7.38 – 7.14 (m, 9H), 6.86 (s, 2H), 4.99 (s, 1H), 4.07 – 3.90 (m, 3H), 1.12 – 1.07 (m, 24H) ppm. **^{13}C NMR (75 MHz, $CDCl_3$)** δ 171.7, 154.0, 153.1, 150.2, 138.5, 134.6, 134.1, 131.2, 129.5, 128.85, 128.1, 127.7, 127.2, 127.0, 126.9, 126.1, 125.5, 125.1, 124.0, 119.6, 113.8, 61.5, 59.0, 34.1, 30.2, 22.5, 14.0 ppm. **HRMS (ESI)** *m/z* Calcd for $[C_{40}H_{46}O_6 + Na]^+$ 645.3187, found 645.3204.

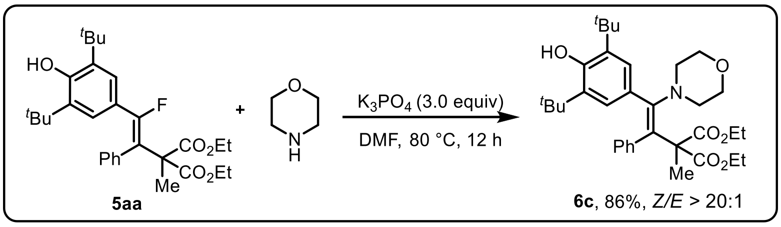

diethyl (Z)-2-(2-(3,5-di-tert-butyl-4-hydroxyphenyl)-2-morpholino-1-phenylvinyl)-2-methylmalonate (6c) To an oven-dried 10 mL Schlenk tube equipped with a Teflon coated magnetic stir bar was added K_3PO_4 (127.4 mg, 0.6 mmol, 3.0 equiv), **5aa** (99.7 mg, 0.2 mmol, 1.0 equiv) and morpholine (34.8 mg, 0.4 mmol, 2.0 equiv) then the Schlenk tube was evacuated and filled with argon for three times. After that, anhydrous DMF (2 mL) was added under argon atmosphere via a syringe under argon atmosphere stirred for 12 h at 80 °C. After the starting material was completely consumed, ice water (5 mL) was slowly added to quench the reaction. The reaction mixture was extracted with EA (3 × 10 mL). The organic layers were combined and dried over anhydrous $MgSO_4$. After removal of the solvent in vacuo, the residue was purified by flash chromatography on silica gel (PE/EA) to afford the corresponding product **6c** as yellow oil (97.6 mg, 86% yield), R_f = 0.4 (PE/EA = 5/1). **^{1}H NMR (300 MHz, $CDCl_3$)** δ 6.99 – 6.88 (m, 3H), 6.87 – 6.83 (m, 2H), 6.60 (s, 2H), 4.92 (s, 1H), 4.20 (q, *J* = 7.1 Hz, 4H), 3.69 (t, *J* = 4.6 Hz, 4H), 2.66 (t, *J* = 4.6 Hz, 4H), 1.49 (s, 3H), 1.29 – 1.24 (m, 24H) ppm. **^{13}C NMR (75 MHz, CDCl3)** δ 171.9, 152.0, 149.8, 138.8, 136.8, 134.1, 130.1, 127.3, 126.9, 125.9, 125.3, 66.5, 61.1, 58.6, 50.2, 33.9, 30.2, 23.8, 14.0 ppm. **HRMS (ESI)** *m/z* Calcd for $[C_{34}H_{47}NO_6 + H]^+$ 566.3477, found 566.3475.

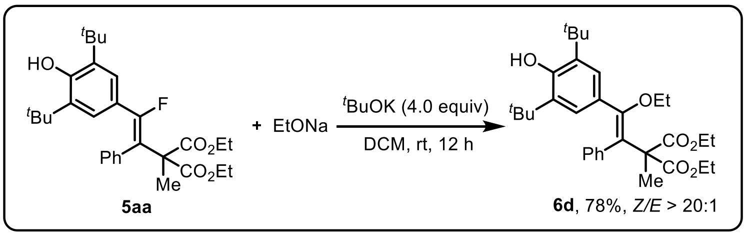

diethyl (Z)-2-(2-(3,5-di-tert-butyl-4-hydroxyphenyl)-2-ethoxy-1-phenylvinyl)-2-methylmalonate (6d) To an oven-dried 10 mL Schlenk tube equipped with a Teflon coated magnetic stir bar was added ^{t}BuOK (89.8 mg, 0.8 mmol, 4.0 equiv), **5aa** (99.7 mg, 0.2 mmol, 1.0 equiv) and EtONa (136.1 mg, 20 wt% in EtOH, 0.4 mmol, 2.0 equiv) then the Schlenk tube was evacuated and filled with argon for three times. After that,

anhydrous DCM (2 mL) was added under argon atmosphere via a syringe under argon atmosphere stirred for 12 h at rt. After the starting material was completely consumed, ice water (5 mL) was slowly added to quench the reaction. The reaction mixture was extracted with DCM (3 × 10 mL). The organic layers were combined and dried over anhydrous $MgSO_4$. After removal of the solvent in vacuo, the residue was purified by flash chromatography on silica gel (PE/EA) to afford the corresponding product **6d** as colorless oil (81.9 mg, 78% yield, *Z*/*E* > 20:1), R_f = 0.3 (PE/EA = 20/1). **^{1}H NMR (300 MHz, $CDCl_3$)** δ 7.33 – 7.27 (m, 2H), 7.25 – 7.20 (m, 5H), 5.25 (s, 1H), 4.29 – 4.10 (m, 2H), 3.94 – 3.81 (m, 4H), 1.42 (s, 3H), 1.32 – 1.19 (m, 27H) ppm. **^{13}C NMR (75 MHz, $CDCl_3$)** δ 172.7, 154.3, 149.0, 135.2, 134.9, 130.5, 128.6, 127.1, 124.7, 121.4, 121.1, 114.7, 64.1, 61.2, 59.3, 59.1, 34.3, 30.1, 17.3, 15.4, 15.3, 14.3 ppm. **HRMS (ESI)** *m/z* Calcd for $[C_{32}H_{44}O_6 + H]^+$ 525.3211, found 525.3217.

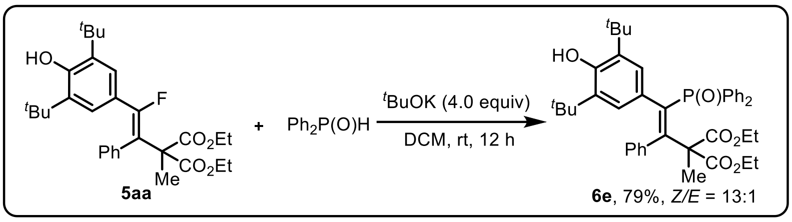

diethyl (Z)-2-(2-(3,5-di-tert-butyl-4-hydroxyphenyl)-2-((diphenylphosphoryl)oxy)-1-phenylvinyl)-2-methylmalonate (6e) To an oven-dried 10 mL Schlenk tube equipped with a Teflon coated magnetic stir bar was added ^{t}BuOK (89.8 mg, 0.8 mmol, 4.0 equiv), **5aa** (99.7 mg, 0.2 mmol, 1.0 equiv) and diphenylphosphine oxide (80.8 mg, 0.4 mmol, 2.0 equiv) then the Schlenk tube was evacuated and filled with argon for three times. After that, anhydrous DCM (2 mL) was added under argon atmosphere via a syringe under argon atmosphere stirred for 12 h at rt. After the starting material was completely consumed, ice water (5 mL) was slowly added to quench the reaction. The reaction mixture was extracted with DCM (3 × 10 mL). The organic layers were combined and dried over anhydrous $MgSO_4$. After removal of the solvent in vacuo, the residue was purified by flash chromatography on silica gel (DCM/MeOH) to afford the corresponding product **6e** as a white solid (107.0 mg, 79% yield, *Z*/*E* = 13:1). **m.p.** 198 – 200 °C, R_f = 0.3 (DCM/MeOH = 20/1). **^{1}H NMR (300 MHz, $CDCl_3$)** δ 7.48 – 7.41 (m, 4H), 7.20 – 7.04 (m, 10H), 6.99 – 6.90 (m, 3H), 5.02 (s, 1H), 3.90 (dq, *J* = 10.7, 7.1 Hz, 2H), 3.70 (dq, *J* = 10.7, 7.1 Hz, 2H)., 1.29 (s, 18H), 1.20 (s, 3H), 1.14 (t, *J* = 7.15 Hz, 6H) ppm. **^{13}C NMR (75 MHz, $CDCl_3$)** δ 171.0 (d, *J* = 1.3 Hz), 153.3 (d, *J* = 11.1 Hz), 152.8 (d, *J* = 2.6 Hz), 140.6 (d, *J* = 91.4 Hz), 136.9 (d, *J* = 8.1 Hz), 135.4, 134.8 (d, *J* = 1.9 Hz), 134.1, 133.1 (d, *J* = 9.1 Hz), 131.2 (d, *J* = 1.6 Hz), 131.0 (d, *J* = 8.7 Hz), 130.1 (d, *J* = 2.8 Hz), 127.6 (d, *J* = 5.0 Hz), 127.4 (d, *J* = 12.0 Hz), 126.5, 62.4 (d, *J* = 12.8 Hz), 61.5, 34.2, 30.1, 24.1, 13.9 ppm. **^{31}P NMR (121 MHz, $CDCl_3$)** δ 24.84 ppm. **HRMS (ESI)** *m/z* Calcd for $[C_{42}H_{49}O_6P + Na]^+$ 703.3159, found 703.3167.

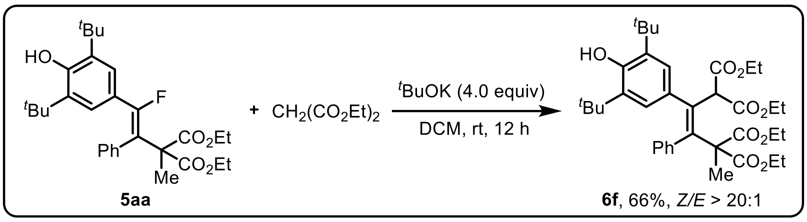

tetraethyl (E)-2-(3,5-di-tert-butyl-4-hydroxyphenyl)-3-phenylpent-2-ene-1,1,4,4-tetracarboxylate (6f) To an oven-dried 10 mL Schlenk tube equipped with a Teflon coated magnetic stir bar was added ^{t}BuOK (89.8 mg, 0.8 mmol, 4.0 equiv), **5aa** (99.7 mg, 0.2 mmol, 1.0 equiv) and dimethyl malonate (60.1 mg, 0.4 mmol, 2.0 equiv) then the Schlenk tube was evacuated and filled with argon for three times. After that, anhydrous DCM (2 mL) was added under argon atmosphere via a syringe under argon atmosphere stirred for 12 h at rt. After the starting material was completely consumed, ice water (5 mL) was slowly added to quench the reaction. The reaction mixture was extracted with DCM (3 × 10 mL). The organic layers were combined and dried over anhydrous $MgSO_4$. After removal of the solvent in vacuo, the residue was purified by flash chromatography on silica gel (PE/EA) to afford the corresponding product **6f** as a white solid (84.2 mg, 66% yield, *Z/E* > 20:1). **m.p.** 163 – 165 °C, R_f = 0.4 (PE/EA = 20/1). **^{1}H NMR (300 MHz, $CDCl_3$)** δ 7.35 – 7.26 (m, 7H), 5.15 (s, 1H), 4.21 (s, 1H), 3.93 – 3.82 (m, 6H), 3.77 – 3.69 (m, 2H), 1.42 (s, 18H), 1.25 (s, 3H), 1.13 (t, *J* = 7.1 Hz, 6H), 1.02 (t, *J* = 7.1 Hz, 6H) ppm. **^{13}C NMR (75 MHz, $CDCl_3$)** δ 171.3, 168.4, 153.2, 140.8, 140.2, 135.9, 134.4, 129.6, 128.4, 127.9, 127.5, 61.3, 61.1, 60.6, 59.9, 34.4, 30.4, 30.3, 24.2, 14.0 ppm. **HRMS (ESI)** *m/z* Calcd for $[C_{37}H_{50}O_9 + H]^+$ 639.3528, found 639.3548.

5. One-pot Reaction for the Synthesis of tetra-Substituted Alkene 6b

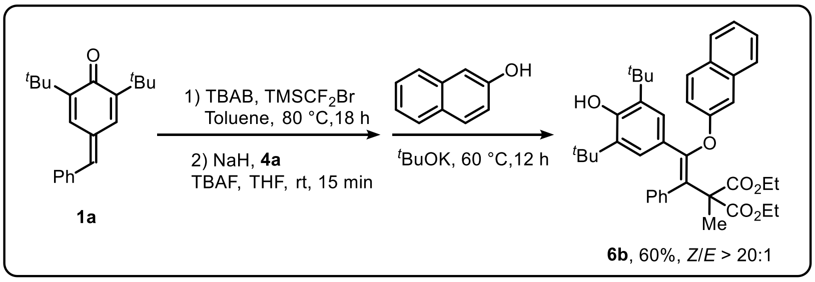

To an oven-dried 10-mL Schlenk tube equipped with a Teflon coated magnetic stir bar was added *p*-QM **1a** (147.2 mg, 0.5 mmol, 1.0 equiv) and TBAB (8.0 mg, 0.025 mmol, 5 mol%), then the Schlenk tube was evacuated and backfilled with argon for three times. After that, $TMSCF_2Br$ (203.1 mg, 1.0 mmol, 2.0 equiv) dissolved in toluene (1.0 mL) was added under argon atmosphere via a syringe. The reaction mixture was stirred at 80 °C (oil bath) for 18 h to afford the solution of intermediate **2a** in toluene.

To another oven-dried 10-mL Schlenk tube equipped with a Teflon coated magnetic stir bar was added NaH (60 mg, 1.5 mmol, 3.0 equiv), then the Schlenk tube was evacuated and backfilled with argon for three times. Compound **4a** (1.0 mmol, 2.0 equiv) dissolved in 1.0 mL anhydrous THF was added under argon atmosphere via a syringe, and the reaction mixture was stirred at room temperature for 30 min. Then the solution of crude **2a** in toluene was added, followed by the dropwise addition of TBAF (0.4 mmol, 0.4 equiv, 0.3 M solution in THF) under argon atmosphere. After stirring at room temperature for 15 min, ^{t}BuOK (224.4 mg, 2.0 mmol, 4.0 equiv) and 2-hydroxynaphthalene (144.2 mg, 1.0 mmol, 2.0 equiv) were directly added to the above-mentioned solution under argon atmosphere. After stirred at 60 °C for 12 h, saturated solution of NH_4Cl (5.0 mL) was added to quench the reaction. The reaction mixture was extracted with ethyl acetate (3 × 10 mL). The organic layers were combined and dried over anhydrous $MgSO_4$. After removal of the solvent in vacuo, the residue was purified by flash chromatography on silica gel (PE/EA) to afford the corresponding product **6b** (186.8 mg, 60% yield, *Z/E* > 20:1).

6. Optimization of Reaction Parameters for the Synthesis of 9aa

Table S1. Base, Solvent Screening[a, b]

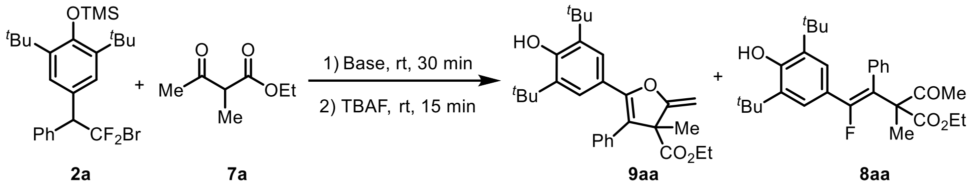

Entry	Base	The equiv of base	Solvent	Yield of **9aa** (%)	Yield of **8aa** (%)
1	^{t}BuOK	3.0	DCM	51	41
2	^{t}BuONa	3.0	DCM	45	29
3	KOH	3.0	DCM	17	51
4	K_2CO_3	3.0	DCM	n.d.	71
5	DBU	3.0	DCM	9	25
6	LDA	3.0	DCM	n.d.	n.d.
7	^{t}BuOK	2.0	DCM	3	60
8	^{t}BuOK	2.5	DCM	20	50
9	^{t}BuOK	3.5	DCM	49	40
10	^{t}BuOK	4.0	DCM	65	30
11	^{t}BuOK	4.0	Toluene	18	77
12	^{t}BuOK	4.0	DMF	8	60
13	^{t}BuOK	4.0	MTBE	48	46
14	^{t}BuOK	4.0	HMPA	4	69

[a] ^{t}BuOK (0.8 mmol, 4.0 equiv) and **7a** (0.4 mmol, 2.0 equiv) in DCM were stirred at rt for 30 min. Then TBAF (1 M in THF) and **2a** (0.2 mmol, 1.0 equiv) were added under Ar. [b]The yields were determined by ^{1}H NMR analysis with 1,3,5-trimethoxybenzene as internal standard.

Table S2. Screening of temperature and the equivalent of TBAF[a, b]

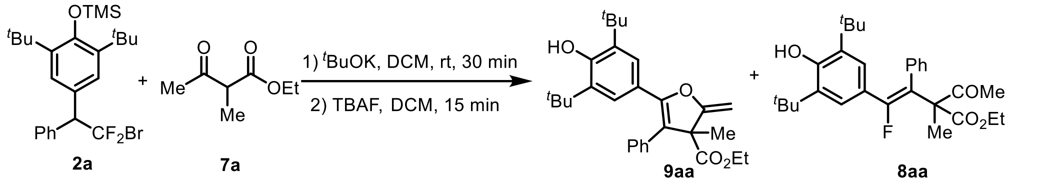

Entry	Temperature (°C)	The equiv of TBAF	Yield of **9aa** (%)	Yield of **8aa** (%)
1	r.t.	1.1	65	30
2	40	1.1	60	36
3	60	1.1	79	13
4	80	1.1	78	5
5	100	1.1	76	n.d.
6	60	0.2	72	8
7	60	0.4	85	3
8	60	0.6	80	13
9	60	0.8	78	5
10	60	1.0	75	23

[a]**7a** (0.4 mmol, 2.0 equiv) and ^{t}BuOK (0.8 mmol, 4.0 equiv) in DCM were stirred at rt for 30 min. Then TBAF (1 M in THF) and **2a** (0.2 mmol, 1.0 equiv) were added under Ar. [b]The yields were determined by ^{1}H NMR analysis with 1,3,5-trimethoxybenzene as internal standard.

7. General Procedure B for the Synthesis of Product 9

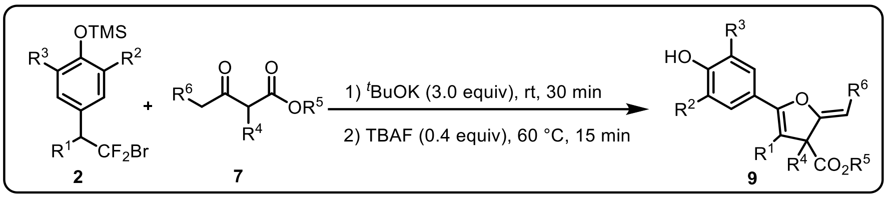

To an oven-dried 10 mL Schlenk tube equipped with a Teflon coated magnetic stir bar was added ^{t}BuOK (89.7 mg, 0.8 mmol, 4.0 equiv), then the Schlenk tube was evacuated and filled with argon for three times. After that, compound **7** (0.4 mmol, 2.0 equiv) dissolved in 1.0 mL anhydrous DCM was added under argon atmosphere via a syringe. The above-mentioned solution was stirred at room temperature for 30 min, followed by the sequential addition of TBAF (0.08 mL, 0.4 equiv, 1 M solution in THF, dissolved in 0.5 mL DCM) and **2** (0.2 mmol in 0.5 mL DCM) under argon atmosphere. After the starting material was completely consumed (typically 15 min at 60 °C), ice water (5 mL) was slowly added to quench the reaction. The reaction mixture was extracted with DCM (3 × 10 mL). The organic layers were combined and dried over anhydrous $MgSO_4$. After removal of the solvent in vacuo, the residue was purified by flash chromatography on silica gel (PE/EA) to afford the corresponding product **9**.

ethyl 5-(3,5-di-tert-butyl-4-hydroxyphenyl)-3-methyl-2-methylene-4-phenyl-2,3-dihydrofuran-3-carboxylate (9aa)

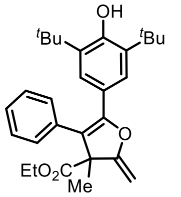

Prepared through general procedure B to give **9aa** in 70.9 mg, 79% yield, white solid, m.p. 165 – 166 °C, R_f = 0.6 (PE/EA = 20/1). **^{1}H NMR (300 MHz, CDCl$_3$)** δ 7.37 – 7.22 (m, 7H), 5.31 (s, 1H), 4.74 (d, *J* = 2.7 Hz, 1H), 4.31 (d, *J* = 2.6 Hz, 1H), 4.26 – 4.16 (m, 2H), 1.56 (s, 3H), 1.28 – 1.23 (m, 21H) ppm. **^{13}C NMR (75 MHz, CDCl$_3$)** δ 172.7, 165.1, 154.6, 150.7, 135.5, 133.9, 130.4, 128.8, 127.6, 124.6, 120.7, 114.4, 83.8, 61.7, 60.0, 34.4, 30.1, 23.9, 14.2 ppm. **HRMS (ESI)** *m/z* Calcd for $[C_{29}H_{36}O_4 + H]^+$ 449.2686, found 449.2698.

ethyl 5-(3,5-di-tert-butyl-4-hydroxyphenyl)-3-methyl-2-methylene-4-(p-tolyl)-2,3-dihydrofuran-3-carboxylate (9ab)

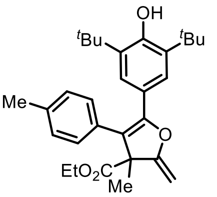

Prepared through general procedure B to give **9ab** in 62.8 mg, 68% yield, yellow solid, m.p. 130 – 131 °C, R_f = 0.6 (PE/EA = 20/1). **^{1}H NMR (300 MHz, CDCl3)** δ 7.23 (s, 1H), 7.18 – 7.05 (m, 2H), 5.28 (s, 1H), 4.71 (d, *J* = 2.6 Hz, 1H), 4.28 (d, *J* = 2.6 Hz, 1H), 4.23 – 4.12 (m, 2H), 2.33 (s, 3H), 1.53 (s, 3H), 1.34 – 1.22 (m, 21H) ppm. **^{13}C NMR (75 MHz, CDCl$_3$)** δ 172.7, 165.1, 154.5, 150.4, 137.3, 135.5, 130.7, 130.3, 129.5, 124.5, 120.8, 114.4, 83.7, 61.6, 60.0, 34.4, 30.1, 23.9, 21.3, 14.2 ppm. **HRMS (ESI)** *m/z* Calcd for $[C_{30}H_{38}O_4 + H]^+$ 463.2843, found 463.2857.

ethyl 5-(3,5-di-tert-butyl-4-hydroxyphenyl)-4-(4-methoxyphenyl)-3-methyl-2-methylene-2,3-dihydrofuran-3-carboxylate (9ac)

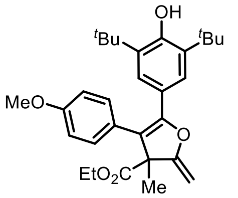

Prepared through general procedure B to give **9ac** in 66.9 mg, 70% yield, yellow solid, m.p. 146 – 147 °C, R_f = 0.5 (PE/EA = 20/1). **^{1}H NMR (300 MHz, $CDCl_3$)** δ 7.26 (s, 2H), 7.17 – 7.12 (m, 2H), 6.90 – 6.85 (m, 2H), 5.30 (s, 1H), 4.72 (d, *J* = 2.6 Hz, 1H), 4.28 (d, *J* = 2.6 Hz, 1H), 4.24 – 4.14 (m, 2H), 3.80 (s, 3H), 1.53 (s, 3H), 1.28 – 1.22 (m, 21H) ppm. **^{13}C NMR (75 MHz, $CDCl_3$)** δ 172.7, 165.1, 159.2, 154.5, 150.4, 135.4, 131.6, 125.9, 124.4, 120.8, 114.4, 114.0, 83.7, 61.6, 59.9, 55.5, 34.4, 30.1, 23.9, 14.2 ppm. **HRMS (ESI)** *m*/*z* Calcd for $[C_{30}H_{38}O_5 + H]^+$ 479.2792, found 479.2807.

ethyl 4-([1,1'-biphenyl]-4-yl)-5-(3,5-di-tert-butyl-4-hydroxyphenyl)-3-methyl-2-methylene-2,3-dihydrofuran-3-carboxylate (9ad)

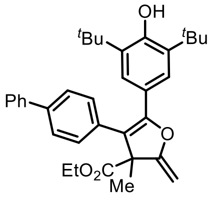

Prepared through general procedure B to give **9ad** in 68.1 mg, 65% yield, yellow solid, m.p. 156 – 157 °C, R_f = 0.6 (PE/EA = 20/1). **^{1}H NMR (300 MHz, $CDCl_3$)** 7.71 – 7.67 (m, 4H), 7.57 (t, *J* = 7.3 Hz, 2H), 7.50 – 7.36 (m, 5H), 5.44 (s, 1H), 4.88 (d, *J* = 2.7 Hz, 1H), 4.45 (d, *J* = 2.7 Hz, 1H), 4.40 – 4.27 (m, 2H), 1.72 (s, 3H), 1.40 – 1.37 (m, 21H) ppm. **^{13}C NMR (75 MHz, $CDCl_3$)** δ 172.7, 165.0, 154.6, 150.9, 141.1, 140.5, 135.5, 132.9, 130.8, 128.9, 127.6, 127.4, 127.2, 124.6, 120.6, 114.0, 84.0, 61.7, 59.9, 34.4, 30.1, 24.0, 14.2 ppm. **HRMS (ESI)** *m*/*z* Calcd for $[C_{35}H_{40}O_4 + H]^+$ 525.2999, found 525.3014.

ethyl 4-(4-chlorophenyl)-5-(3,5-di-tert-butyl-4-hydroxyphenyl)-3-methyl-2-methylene-2,3-dihydrofuran-3-carboxylate (9ae)

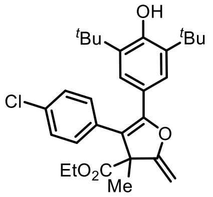

Prepared through general procedure B to give **9ae** in 64.6 mg, 67% yield, purple solid, m.p. 141 – 142 °C, R_f = 0.6 (PE/EA = 20/1). **^{1}H NMR (300 MHz, $CDCl_3$)** δ 7.35 – 7.32 (m, 2H), 7.24 (s, 2H), 7.21 – 7.18 (m, 2H), 5.37 (s, 1H), 4.77 (d, *J* = 2.8 Hz, 1H), 4.35 (d, *J* = 2.7 Hz, 1H), 4.28 – 4.15 (m, 2H), 1.57 (s, 3H), 1.32 – 1.24 (m, 21H) ppm. **^{13}C NMR (75 MHz, $CDCl_3$)** δ 172.5, 164.7, 154.8, 151.2, 135.6, 133.5, 132.5, 131.8, 129.0, 124.6, 120.3, 113.0, 84.3, 61.8, 59.7, 34.4, 30.1, 23.9, 14.2 ppm. **HRMS (ESI)** *m*/*z* Calcd for $[C_{29}H_{35}ClO_4 + H]^+$ 483.2297, found 483.2312.

ethyl 5-(3,5-di-tert-butyl-4-hydroxyphenyl)-4-(4-(methoxycarbonyl)phenyl)-3-methyl-2-methylene-2,3-dihydrofuran-3-carboxylate (9af)

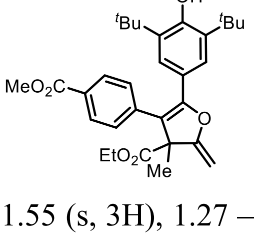

Prepared through general procedure B to give **9af** in 57.7 mg, 57% yield, yellow solid, m.p. 135 – 136 °C, R_f = 0.5 (PE/EA = 20/1). **^{1}H NMR (300 MHz, $CDCl_3$)** δ 7.98 (d, *J* = 8.4 Hz, 2H), 7.30 (d, *J* = 8.4 Hz, 2H), 7.23 (s, 2H), 5.36 (s, 1H), 4.75 (d, *J* = 2.8 Hz, 1H), 4.34 (d, *J* = 2.8 Hz, 1H), 4.26 – 4.12 (m, 2H), 3.92 (s, 2H), 1.55 (s, 3H), 1.27 – 1.20 (m, 21H) ppm. **^{13}C NMR (75 MHz, $CDCl_3$)** δ 172.4, 167.0, 164.7, 155.0, 151.9, 139.2, 135.7, 130.1, 130.0, 128.9, 124.9, 120.2, 113.4, 84.4, 61.8, 59.6, 52.3, 34.4, 30.1, 23.9, 14.2 ppm. **HRMS (ESI)** *m*/*z* Calcd for $[C_{31}H_{38}O_6 + H]^+$

507.2741, found 507.2751.

ethyl 5-(3,5-di-tert-butyl-4-hydroxyphenyl)-3-methyl-2-methylene-4-(4-nitrophenyl)-2,3-dihydrofuran-3-carboxylate (9ag)

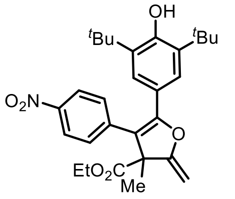

Prepared through general procedure B to give **9ag** in 45.4 mg, 46% yield, yellow solid, m.p. 185 – 186 °C, R_f = 0.5 (PE/EA = 20/1). **^{1}H NMR (300 MHz, CDCl$_3$)** δ 8.15 (d, *J* = 8.6 Hz, 2H), 7.37 (d, *J* = 8.7 Hz, 2H), 7.22 (s, 2H), 5.42 (s, 1H), 4.78 (d, *J* = 2.9 Hz, 1H), 4.39 (d, *J* = 2.9 Hz, 1H), 4.30 – 4.14 (m, 2H), 1.58 (s, 3H), 1.29 – 1.21 (m, 21H) ppm. **^{13}C NMR (75 MHz, CDCl$_3$)** δ 172.4, 164.4, 155.5, 153.7, 146.6, 141.6, 136.0, 130.5, 125.2, 123.9, 119.9, 112.4, 85.1, 62.0, 59.3, 34.5, 30.1, 24.0, 14.2 ppm. **HRMS (ESI)** *m*/*z* Calcd for $[C_{29}H_{35}NO_6 + H]^+$ 494.2537, found 494.2547.

ethyl 5-(3,5-di-tert-butyl-4-hydroxyphenyl)-3-methyl-2-methylene-4-(4-(trifluoromethyl)phenyl)-2,3-dihydrofuran-3-carboxylate (9ah)

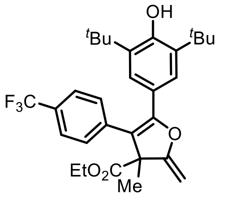

Prepared through general procedure B to give **9ah** in 64.0 mg, 62% yield, white solid, m.p. 140 – 141 °C, R_f = 0.7 (PE/EA = 20/1). **^{1}H NMR (300 MHz, CDCl$_3$)** δ 7.62 (d, *J* = 7.9 Hz, 2H), 7.38 (d, *J* = 8.0 Hz, 2H), 7.20 (s, 2H), 5.38 (s, 1H), 4.79 (d, *J* = 2.6 Hz, 1H), 4.38 (d, *J* = 2.6 Hz, 1H), 4.27 – 4.20 (m, 2H), 1.59 (s, 3H), 1.36 – 1.25 (m, 21H) ppm. **^{13}C NMR (75 MHz, CDCl$_3$)** δ 172.4, 164.6, 155.0, 151.9, 130.2, 129.6 (q, ^2J = 32.4 Hz), 125.7 (q, ^3J = 3.8 Hz), 124.7, 124.3 (q, ^1J = 270.2 Hz), 120.1, 112.9, 84.6, 61.9, 59.7, 34.4, 30.1, 24.0, 14.2 ppm. **^{19}F NMR (282 MHz, CDCl$_3$)** δ –62.76 ppm. **HRMS (ESI)** *m*/*z* Calcd for $[C_{30}H_{35}F_3O_4 + H]^+$ 517.2560, found 517.2559.

ethyl 5-(3,5-di-tert-butyl-4-hydroxyphenyl)-4-(3-methoxyphenyl)-3-methyl-2-methylene-2,3-dihydrofuran-3-carboxylate (9ai)

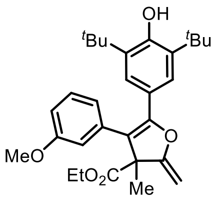

Prepared through general procedure B to give **9ai** in 66.9 mg, 70% yield, yellow solid, m.p. 150 – 151 °C, R_f = 0.6 (PE/EA = 20/1). **^{1}H NMR (300 MHz, CDCl$_3$)** δ 7.27 (s, 2H), 7.26 – 7.22 (m, 1H), 6.85 – 6.76 (m, 3H), 5.31 (s, 1H), 4.72 (d, *J* = 2.6 Hz, 1H), 4.29 (d, *J* = 2.7 Hz, 1H), 4.25 – 4.15 (m, 2H), 3.74 (s, 3H), 1.54 (s, 3H), 1.28 – 1.23 (m, 21H) ppm. **^{13}C NMR (75 MHz, CDCl$_3$)** δ 172.7, 165.0, 160.0, 154.6, 150.7, 135.5, 135.1, 129.8, 124.6, 122.8, 120.6, 115.8, 114.2, 113.3, 83.8, 61.7, 59.9, 55.3, 34.4, 30.2, 23.9, 14.2 ppm. **HRMS (ESI)** *m*/*z* Calcd for $[C_{30}H_{38}O_5 + H]^+$ 479.2792, found 479.2808.

ethyl 5-(3,5-di-tert-butyl-4-hydroxyphenyl)-3-methyl-2-methylene-4-(3-nitrophenyl)-2,3-dihydrofuran-3-carboxylate (9aj)

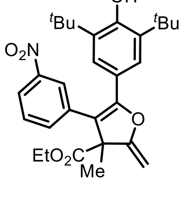

Prepared through general procedure B to give **9aj** in 52.3 mg, 53% yield, green solid, m.p. 133 – 134 °C, R_f = 0.5 (PE/EA = 20/1). **^{1}H NMR (300 MHz, CDCl$_3$)** δ 8.16 – 8.12 (m, 2H), 7.59 – 7.48 (m, 2H), 7.20 (s, 2H), 5.39 (s, 1H), 4.79 (d, *J* = 2.8 Hz, 1H), 4.39 (d, *J* = 2.9 Hz, 1H), 4.32 – 4.14 (m, 2H), 1.58 (s, 3H), 1.29 – 1.25 (m, 21H) ppm. **^{13}C**

NMR (75 MHz, $CDCl_3$) δ 172.3, 164.3, 155.2, 152.6, 148.6, 136.7, 136.1, 135.9, 129.7, 125.2, 124.8, 122.3, 119.8, 111.7, 85.0, 62.1, 59.5, 34.4, 30.1, 24.1, 14.2 ppm. **HRMS (ESI)** *m/z* Calcd for $[C_{29}H_{35}NO_6 + H]^+$ 494.2537, found 494.2548.

ethyl 5-(3,5-di-tert-butyl-4-hydroxyphenyl)-4-(2-methoxyphenyl)-3-methyl-2-methylene-2,3-dihydrofuran-3-carboxylate (9ak)

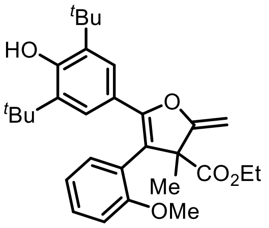

Prepared through general procedure B to give **9ak** in 67.9 mg, 71% yield, green solid, m.p. 159 – 160 °C, R_f = 0.5 (PE/EA = 20/1). **^{1}H NMR (300 MHz, $CDCl_3$)** δ 7.27 (s, 2H), 7.26 – 7.22 (m, 1H), 6.84 – 6.76 (m, 3H), 5.31 (s, 1H), 4.75 (d, J = 2.6 Hz, 1H), 4.32 (d, J = 2.7 Hz, 1H), 4.25 – 4.15 (m, 2H), 3.74 (s, 3H), 1.54 (s, 3H), 1.28 – 1.23 (m, 21H) ppm. **^{13}C NMR (75 MHz, $CDCl_3$)** δ 172.7, 165.0, 160.0, 154.6, 150.7, 135.5, 135.1, 129.8, 124.6, 122.8, 120.6, 115.8, 114.2, 113.3, 83.8, 61.7, 59.9, 55.3, 34.4, 30.1, 23.9, 14.2 ppm. **HRMS (ESI)** *m/z* Calcd for $[C_{30}H_{38}O_5 + H]^+$ 479.2792, found 479.2806.

ethyl 4-(2-chlorophenyl)-5-(3,5-di-tert-butyl-4-hydroxyphenyl)-3-methyl-2-methylene-2,3-dihydrofuran-3-carboxylate (9al)

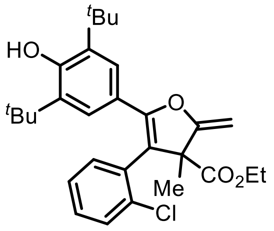

Prepared through general procedure B to give **9al** in 75.2 mg, 78% yield, yellow solid, m.p. 115 – 116 °C, R_f = 0.4 (PE/EA = 20/1). **^{1}H NMR (300 MHz, $CDCl_3$)** δ 7.39 – 7.35 (m, 1H), 7.20 – 7.12 (m, 5H), 5.23 (s, 1H), 4.70 (d, J = 2.6 Hz, 1H), 4.21 (d, J = 2.8 Hz, 1H), 4.18 – 4.09 (m, 2H), 1.47 (s, 3H), 1.26 – 1.13 (m, 21H) ppm. **^{13}C NMR (75 MHz, $CDCl_3$)** δ 172.3, 165.0, 154.8, 151.6, 136.3, 135.6, 132.9, 132.5, 130.2, 129.3, 127.2, 123.9, 120.6, 109.8, 84.4, 61.7, 34.4 (two overlapping carbon signals), 30.1 (two overlapping carbon signals), 14.2 ppm. **HRMS (ESI)** *m/z* Calcd for $[C_{29}H_{35}ClO_4 + H]^+$ 483.2297, found 483.2312.

ethyl 4-(2-bromophenyl)-5-(3,5-di-tert-butyl-4-hydroxyphenyl)-3-methyl-2-methylene-2,3-dihydrofuran-3-carboxylate (9am)

Prepared through general procedure B to give **9am** in 86.3 mg, 82% yield, yellow solid,

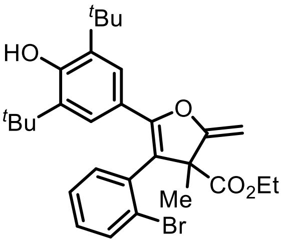

m.p. 119 – 120 °C, R_f = 0.4 (PE/EA = 20/1). **^{1}H NMR (300 MHz, $CDCl_3$)** δ 7.69 (d, J = 8.0 Hz, 1H), 7.37 – 7.18 (m, 5H), 5.35 (s, 1H), 4.79 (d, J = 2.4 Hz,1H), 4.32 – 4.26 (m, 3H), 1.60 (s, 3H), 1.35 – 1.27 (m, 21H) ppm. **^{13}C NMR (75 MHz, $CDCl_3$)** δ 172.5, 165.0, 154.8, 151.5, 135.5, 134.9, 133.5, 132.2, 129.5, 127.9, 127.2, 123.9, 120.6, 111.3, 84.1, 61.7, 61.4, 34.4, 30.1, 22.7, 14.2 ppm. **HRMS (ESI)** *m/z* Calcd for $[C_{29}H_{35}BrO_4 + H]^+$ 527.1791, found 527.1802.

ethyl 5-(3,5-di-tert-butyl-4-hydroxyphenyl)-3-methyl-2-methylene-4-(1-tosyl-1H-indol-5-yl)-2,3-dihydrofuran-3-carboxylate (9an)

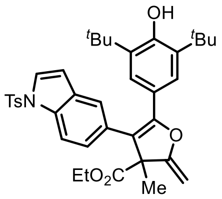

Prepared through general procedure B to give **9an** in 55.1 mg, 43% yield, white solid, m.p. 199 – 200 °C, R_f = 0.5 (PE/EA = 20/1). **^{1}H NMR (300 MHz, CDCl$_3$)** δ 8.03 (d, *J* = 8.3 Hz, 1H), 7.83 (d, *J* = 7.9 Hz, 2H), 7.52 (s, 1H), 7.33 – 7.25 (m, 3H), 7.17 – 7.07 (m, 4H), 5.27 (s, 1H), 4.82 (d, *J* = 2.7 Hz, 1H), 4.40 (d, *J* = 2.7 Hz, 1H), 4.19 – 4.10 (m, 2H), 2.37 (s, 3H), 1.57 (s, 3H), 1.14 (t, *J* = 7.1 Hz, 3H), 1.03 (s, 18H) ppm. **^{13}C NMR (75 MHz, CDCl$_3$)** δ 172.5, 164.5, 154.7, 152.2, 145.1, 135.3, 135.1, 135.0, 130.4, 130.1, 127.1, 125.0, 124.4, 124.2, 123.3, 121.8, 120.1, 115.5, 113.6, 104.2, 84.6, 61.8, 60.1, 34.1, 29.7, 24.4, 21.7, 14.1 ppm. **HRMS (ESI)** *m*/*z* Calcd for [$C_{38}H_{43}NO_6S$ + H]$^+$ 642.2884, found 642.2900.

ethyl 5-(3,5-di-tert-butyl-4-hydroxyphenyl)-3-methyl-2-methylene-4-(thiophen-2-yl)-2,3-dihydrofuran-3-carboxylate (9ao)

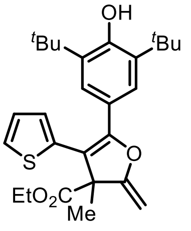

Prepared through general procedure B to give **9ao** in 55.4 mg, 61% yield, yellow solid, m.p. 135 – 136 °C, R_f = 0.6 (PE/EA = 20/1). **^{1}H NMR (300 MHz, CDCl$_3$)** δ 7.29 (s, 2H), 7.22 – 7.18 (m, 1H), 6.95 – 6.92 (m, 1H), 6.82 – 6.81 (m, 1H), 5.29 (s, 1H), 4.66 (d, *J* = 2.8 Hz, 1H), 4.26 (d, *J* = 2.9 Hz, 1H), 4.18 – 4.07 (m, 2H), 1.51 (s, 3H), 1.25 – 1.16 (m, 21H) ppm. **^{13}C NMR (75 MHz, CDCl$_3$)** δ 172.4, 164.6, 155.0, 152.4, 135.6, 134.6, 128.1, 127.4, 126.5, 124.8, 120.2, 107.6, 84.5, 61.8, 59.7, 34.4, 30.2, 24.0, 14.2 ppm. **HRMS (ESI)** *m*/*z* Calcd for [$C_{27}H_{34}O_4S$ + H]$^+$ 455.2251, found 455.2264.

ethyl 4-cyclopropyl-5-(3,5-di-tert-butyl-4-hydroxyphenyl)-3-methyl-2-methylene-2,3-dihydrofuran-3-carboxylate (9ap)

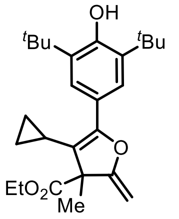

Prepared through general procedure B to give **9ap** in 35 mg, 42% yield, white solid, m.p. 134 – 136 °C, R_f = 0.5 (PE/EA = 20/1). **^{1}H NMR (300 MHz, CDCl$_3$)** δ 7.65 (s, 2H), 5.37 (s, 1H), 4.59 (d, *J* = 2.6 Hz, 1H), 4.24 – 4.07 (m, 3H), 1.60 (s, 3H), 1.52 – 1.47 (m, 19H), 1.23 (t, *J* = 7.1 Hz, 3H), 0.85 – 0.68 (m, 2H), 0.55 – 0.47 (m, 1H), 0.41 – 0.33 (m, 3H) ppm. **^{13}C NMR (75 MHz, CDCl$_3$)** δ 173.6, 165.2, 154.3, 151.6, 135.5, 124.8, 121.4, 113.6, 83.0, 61.5, 59.3, 34.6, 30.4, 24.4, 14.1, 7.4, 6.9, 6.2 ppm. **HRMS (ESI)** *m*/*z* Calcd for [$C_{26}H_{36}O_4$ + H]$^+$ 413.2686, found 413.2689.

ethyl 5-(3-(tert-butyl)-4-hydroxy-5-methylphenyl)-3-methyl-2-methylene-4-phenyl-2,3-dihydrofuran-3-carboxylate (9aq)

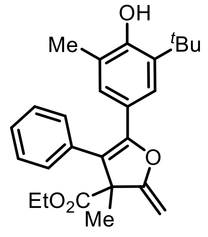

Prepared through general procedure B to give **9aq** in 51.9 mg, 64% yield, yellow solid, m.p. 81 – 82 °C, R_f = 0.5 (PE/EA = 20/1). **^{1}H NMR (300 MHz, CDCl$_3$)** δ 7.34 – 7.18 (m, 6H), 7.07 (s, 1H), 4.92 (s, 1H), 4.71 (d, *J* = 2.7 Hz, 1H), 4.30 (d, *J* = 2.4 Hz, 1H), 4.23 – 4.16 (m, 2H), 2.16 (s, 3H), 1.54 (s, 3H), 1.24 (t, *J* = 7.0 Hz, 3H), 1.16 (s, 9H) ppm. **^{13}C NMR (75 MHz, CDCl$_3$)** δ 172.7, 165.0, 153.4, 150.4,

135.2, 133.7, 130.3, 128.8, 127.6, 127.6, 125.2, 122.8, 121.2, 114.6, 83.9, 61.7, 59.9, 34.5, 29.5, 23.8, 16.0, 14.2 ppm. **HRMS (ESI)** *m*/*z* Calcd for [$C_{26}H_{30}O_4$ + H]$^+$ 407.2217, found 407.2230.

ethyl 5-(3,5-di-tert-butyl-4-hydroxyphenyl)-3-ethyl-2-methylene-4-phenyl-2,3-dihydrofuran-3-carboxylate (9ba)

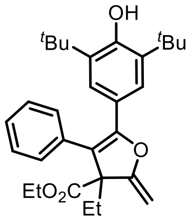

Prepared through general procedure B to give **9ba** in 65.6 mg, 71% yield, white solid, m.p. 183 – 184 °C, R_f = 0.5 (PE/EA = 20/1). **^{1}H NMR (300 MHz, $CDCl_3$)** δ 7.34 – 7.19 (m, 7H), 5.31 (s, 1H), 4.80 (d, *J* = 2.6 Hz, 1H), 4.23 (d, *J* = 2.7 Hz, 1H), 4.23 – 4.11 (m, 2H), 1.95 (q, *J* = 7.4 Hz, 2H), 1.27 (s, 18H), 1.21 (t, *J* = 7.1 Hz, 3H), 0.94 (t, *J* = 7.4 Hz, 3H) ppm. **^{13}C NMR (75 MHz, $CDCl_3$)** δ 172.7, 162.5, 154.6, 151.8, 135.4, 133.9, 130.2, 128.8, 127.5, 124.7, 120.6, 111.5, 84.1, 65.1, 61.5, 34.4, 30.1, 27.6, 14.2, 8.5 ppm. **HRMS (ESI)** *m*/*z* Calcd for [$C_{30}H_{38}O_4$ + H]$^+$ 463.2843, found 463.2860.

ethyl 5-(3,5-di-tert-butyl-4-hydroxyphenyl)-3-isopropyl-2-methylene-4-phenyl-2,3-dihydrofuran-3-carboxylate (9ca)

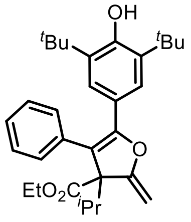

Prepared through general procedure B to give **9ca** in 42.8 mg, 45% yield, purple solid, m.p. 141 – 142 °C, R_f = 0.7 (PE/EA = 20/1). **^{1}H NMR (300 MHz, $CDCl_3$)** δ 7.48 – 7.37 (m, 5H), 7.31 (s, 2H), 5.40 (s, 1H), 5.04 (d, *J* = 2.5 Hz, 1H), 4.46 (d, *J* = 2.5 Hz, 1H), 4.27 (q, *J* = 7.1 Hz, 2H), 2.65 – 2.56 (m, 1H), 1.38 (s, 18H), 1.27 (t, *J* = 7.2 Hz, 3H), 1.17 (d, *J* = 6.6 Hz, 3H), 1.03 (d, *J* = 6.8 Hz, 3H) ppm. **^{13}C NMR (75 MHz, $CDCl_3$)** δ 171.6, 161.0, 154.4, 151.3, 135.3, 134.8, 130.9, 128.7, 127.5, 124.8, 120.7, 111.8, 86.7, 69.1, 61.2, 34.3, 33.4, 30.1, 18.1, 16.9, 14.1 ppm. **HRMS (ESI)** *m*/*z* Calcd for [$C_{31}H_{40}O_4$ + H]$^+$ 477.2999, found 477.3012.

ethyl 3-benzyl-5-(3,5-di-tert-butyl-4-hydroxyphenyl)-2-methylene-4-phenyl-2,3-dihydrofuran-3-carboxylate (9da)

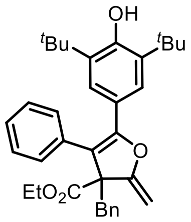

Prepared through general procedure B to give **9da** in 87.0 mg, 83% yield, yellow solid, m.p. 132 – 133 °C, R_f = 0.5 (PE/EA = 20/1). **^{1}H NMR (300 MHz, $CDCl_3$)** δ 7.39 – 7.31 (m, 3H), 7.26 – 7.20 (m, 9H), 5.41 (s, 1H), 4.92 (d, *J* = 2.9 Hz, 1H), 4.41 – 4.34 (m, 3H), 3.56 – 3.40 (m, 2H), 1.39 – 1.32 (m, 21H) ppm. **^{13}C NMR (75 MHz, $CDCl_3$)** δ 172.6, 162.2, 154.7, 152.4, 136.5, 135.5, 133.6, 130.6, 129.6, 128.5, 127.8, 127.1, 126.6, 125.1, 120.8, 112.4, 85.8, 64.5, 61.8, 41.1, 34.3, 30.2, 14.1 ppm. **HRMS (ESI)** *m*/*z* Calcd for [$C_{35}H_{40}O_4$ + H]$^+$ 525.2999, found 525.3011.

benzyl 5-(3,5-di-tert-butyl-4-hydroxyphenyl)-3-methyl-2-methylene-4-phenyl-2,3-dihydrofuran-3-carboxylate (9ea)

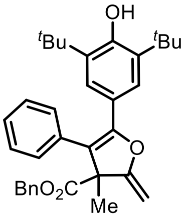

Prepared through general procedure B to give **9ea** in 71.4 mg, 70% yield, yellow solid, m.p. 177 – 178 °C, R_f = 0.6 (PE/EA = 20/1). **^{1}H NMR (300 MHz, CDCl$_3$)** δ 7.30 – 7.29 (m, 5H), 7.23 – 7.21 (m, 5H), 7.07– 7.04 (m, 2H), 5.30 (s, 1H), 5.27 – 5.10 (m, 2H), 4.74 (d, *J* = 2.7 Hz, 1H), 4.29 (d, *J* = 2.7 Hz, 1H), 1.55 (s, 3H), 1.26 (s, 18H) ppm. **^{13}C NMR (75 MHz, CDCl$_3$)** δ 172.4, 164.8, 154.6, 150.9, 135.9, 135.5, 133.6, 130.3, 128.8, 128.5, 128.3, 128.2, 127.5, 124.7, 120.6, 114.2, 84.2, 67.2, 59.9, 34.3, 30.1, 23.7 ppm. **HRMS (ESI)** *m*/*z* Calcd for $[C_{34}H_{38}O_4 + H]^+$ 511.2843, found 511.2858.

ethyl (Z)-5-(3,5-di-tert-butyl-4-hydroxyphenyl)-2-ethylidene-3-methyl-4-phenyl-2,3-dihydrofuran-3-carboxylate (9fa)

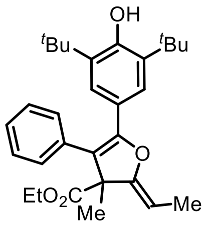

Prepared through general procedure B to give **9fa** in 52.8 mg, 57% yield, yellow solid, m.p. 164 – 166 °C, R_f = 0.5 (PE/EA = 20/1). **^{1}H NMR (300 MHz, CDCl$_3$)** δ 7.33 – 7.20 (m, 7H), 5.29 (s, 1H), 4.65 (q, *J* = 6.9 Hz, 1H), 4.26 – 4.09 (m, 2H), 1.79 (d, *J* = 6.9 Hz, 3H), 1.50 (s, 3H), 1.27 – 1.20 (m, 21H) ppm. **^{13}C NMR (75 MHz, CDCl$_3$)** δ 173.1, 157.6, 154.5, 150.4, 135.4, 134.1, 130.5, 128.7, 127.4, 124.6, 121.0, 114.2, 94.8, 61.5, 59.3, 34.3, 30.1, 23.9, 14.3, 10.6 ppm. **HRMS (ESI)** *m*/*z* Calcd for $[C_{30}H_{38}O_4 + H]^+$ 463.2843, found 463.2841.

ethyl (Z)-2-benzylidene-5-(3,5-di-tert-butyl-4-hydroxyphenyl)-3-methyl-4-phenyl-2,3-dihydrofuran-3-carboxylate (9ga)

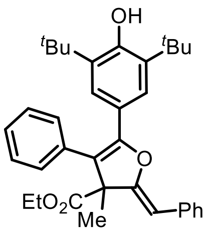

Prepared through general procedure B at room temperature to give **9ga** in 52.7 mg, 50% yield, yellow solid, m.p. 163 – 165 °C, R_f = 0.5 (PE/EA = 20/1). **^{1}H NMR (300 MHz, CDCl$_3$)** δ 7.75 (d, *J* = 7.6 Hz, 2H), 7.38 – 7.27 (m, 9H), 7.23 – 7.17 (m, 1H), 5.59 (s, 1H), 5.34 (s, 1H), 4.26 – 4.14 (m, 2H), 1.63 (s, 3H), 1.31 – 1.21 (m, 21H) ppm. **^{13}C NMR (75 MHz, CDCl$_3$)** δ 172.5, 158.2, 154.6, 150.6, 135.6, 133.5, 130.5, 128.9, 128.4, 128.3, 127.8, 126.0, 124.5, 120.6, 114.3, 100.5, 61.8, 61.5, 34.4, 30.1, 23.8, 14.3 ppm. **HRMS (ESI)** *m*/*z* Calcd for $[C_{35}H_{40}O_4 + H]^+$ 525.3000, found 525.2997.

ethyl 2-(3,5-di-tert-butyl-4-hydroxyphenyl)-3-phenyl-5,6-dihydrobenzofuran-3a(4H)-carboxylate (9ha)

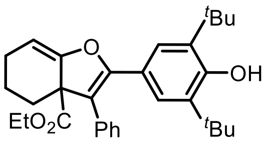

Prepared through general procedure B to give **9ha** in 40.1 mg, 42% yield, green solid, m.p. 165 – 167 °C, R_f = 0.4 (PE/EA = 20/1). **^{1}H NMR (300 MHz, CDCl$_3$)** δ 7.34 – 7.19 (m, 7H), 5.29 – 5.27 (m, 2H), 4.26 – 4.08 (m, 2H), 2.47 – 2.42 (m, 1H), 2.27 – 2.21 (m, 1H), 1.84 – 1.76 (m, 1H), 1.63 – 1.46 (m, 2H), 1.26 (s, 18H), 1.19 (t, *J* = 7.1 Hz, 3H) ppm. **^{13}C NMR (75 MHz, CDCl$_3$)** δ 172.2, 155.1, 154.6,

152.6, 135.4, 134.3, 130.0, 128.7, 127.2, 124.9, 121.0, 113.2, 99.0, 61.2, 58.9, 34.4, 30.2, 29.2, 22.2, 18.9, 14.4 ppm. **HRMS (ESI)** *m/z* Calcd for [$C_{31}H_{38}O_4$ + H] $^+$ 475.2843, found 475.2836.

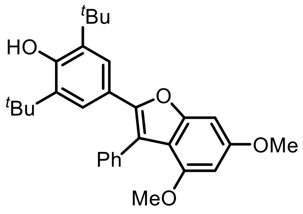

2,6-di-tert-butyl-4-(4,6-dimethoxy-3-phenylbenzofuran-2-yl)phenol (9ia) Prepared through general procedure B in 25 min to give **9ia** in 40.3 mg, 44% yield, yellow solid, m.p. 178 – 181 °C, R_f = 0.7 (PE/EA = 20/1). **^{1}H NMR (300 MHz, $CDCl_3$)** δ 7.48 – 7.44 (m, 2H), 7.41 – 7.31 (m, 5H), 6.74 (d, *J* = 2.0 Hz, 1H), 6.28 (d, *J* = 2.0 Hz, 1H), 5.23 (s, 1H), 3.87 (s, 3H), 3.65 (s, 3H), 1.30 (s, 18H) ppm. **^{13}C NMR (75 MHz, $CDCl_3$)** δ 158.8, 155.6, 154.7, 153.7, 150.0, 135.7, 134.6, 130.9, 128.1, 127.0, 123.8, 122.3, 115.4, 113.5, 94.5, 88.2, 55.9, 55.6, 34.4, 30.2 ppm. **HRMS (ESI)** *m/z* Calcd for [$C_{30}H_{34}O_4$ + H]$^+$ 459.2530, found 459.2523.

2,6-di-tert-butyl-4-(5-methyl-3-phenyl-5H-pyrano[4,3,2-cd]indol-2-yl)phenol (9ja)

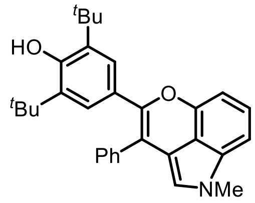

Prepared through general procedure B to give **9ja** in 42.5 mg, 47% yield, yellow oil, R_f = 0.7 (PE/EA = 20/1). **^{1}H NMR (300 MHz, $CDCl_3$)** δ 7.57 – 7.43 (m, 6H), 7.38 – 7.33 (m, 1H), 7.28 – 7.15 (m, 2H), 7.07 (s, 1H), 6.83 (s, 1H), 5.23 (s, 1H), 3.83 (s, 3H), 1.35 (s, 18H) ppm. **^{13}C NMR (75 MHz, $CDCl_3$)** δ 153.7, 149.5, 147.2, 136.2, 135.9, 134.6, 130.3, 128.9, 128.3, 127.2, 123.8, 122.8, 122.2, 116.7, 114.3, 113.4, 105.6, 97.2, 34.5, 33.6, 30.3 ppm. **HRMS (ESI)** *m/z* Calcd for [$C_{31}H_{33}NO_2$ + H]$^+$ 452.2584, found 452.2583.

2,6-di-tert-butyl-4-(1-phenylnaphtho[2,1-b]furan-2-yl)phenol (9ka)

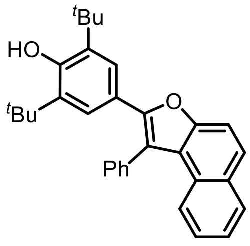

Prepared through general procedure B to give **9ka** in 60.1 mg, 67% yield, m.p. 173 – 174 °C, R_f = 0.6 (PE/EA = 20/1). **^{1}H NMR (300 MHz, $CDCl_3$)** δ 7.90 (d, *J* = 8.1 Hz, 1H), 7.76 – 7.69 (m, 2H), 7.57 – 7.46 (m, 8H), 7.36 (t, *J* = 7.4 Hz, 1H), 7.26 – 7.21 (m, 1H), 5.27 (s, 1H), 1.32 (s, 18H) ppm. **^{13}C NMR (75 MHz, $CDCl_3$)** δ 153.9, 151.3, 151.0, 135.9, 135.5, 130.92, 130.87, 129.4, 128.9, 128.2, 128.0, 125.8, 125.0, 124.1, 124.0, 123.5, 123.2, 122.2, 117.5, 112.3, 34.4, 30.1 ppm. **HRMS (ESI)** *m/z* Calcd for [$C_{32}H_{32}O_2$ + Na]$^+$ 471.2295, found 471.2296.

8. Gram-scale Reaction for the Synthesis of 9aa

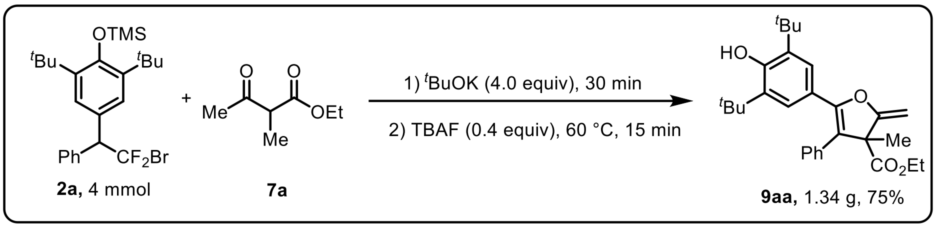

To a 100 mL two necked bottle equipped with a Teflon coated magnetic stir bar was added ^{t}BuOK (1.79 g, 16.0 mmol, 4.0 equiv) and **7a** (8.0 mmol, 2.0 equiv). Then the bottle was evacuated and filled with argon for three times. After that, anhydrous DCM (10 mL) was added under argon atmosphere via a syringe. The above-mentioned solution was stirred at room temperature for 30 min. Then TBAF (1.6 mL, 1.6 mmol, 1 M in THF) dissolved in 10 mL DCM, and compound **2a** (4.0 mmol in 10 mL DCM) was added in sequential under argon atmosphere. After stirring at 60 ℃ for 15 min, ice water (15 mL) was slowly added to quench the reaction. The reaction mixture was extracted with DCM (3 × 10 mL). The organic layers were combined and dried over anhydrous $MgSO_4$. After removal of the solvent in vacuo, the residue was purified by flash chromatography on silica gel (PE/EA) to afford the corresponding product **9aa** as a white solid (1.34 g, 75% yield).

9. One-pot Reaction for the Synthesis of 9aa and 9ka

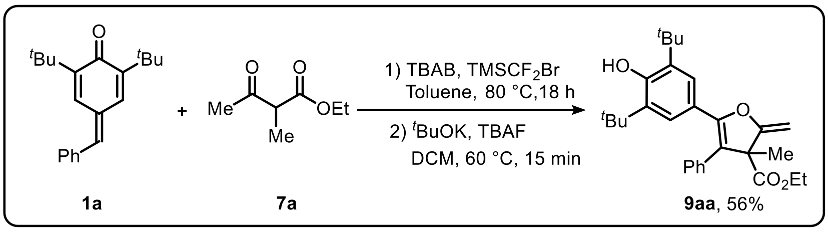

To an oven-dried 10-mL Schlenk tube equipped with a Teflon coated magnetic stir bar was added **1a** (147.2 mg, 0.5 mmol, 1.0 equiv) and TBAB (8.0 mg, 0.025 mmol, 5 mol%), then the Schlenk tube was evacuated and backfilled with argon for three times. After that, $TMSCF_2Br$ (203.1 mg, 1.0 mmol, 2.0 equiv) dissolved in toluene (1.0 mL) was added under argon atmosphere via a syringe. The reaction mixture was stirred at 80 °C (oil bath) for 18 h to afford the toluene solution of crude intermediate.

To another oven-dried 10-mL Schlenk tube equipped with a Teflon coated magnetic stir bar was added ^{t}BuOK (224.4 mg, 2.0 mmol, 4.0 equiv), then the Schlenk tube was evacuated and filled with argon for three times. After that, anhydrous DCM (1.0 mL) and **7a** (1.0 mmol, 2.0 equiv, dissolved in 1.0 mL THF) was added under argon atmosphere via a syringe. After stirred at room temperature for 30 min, the toluene solution of crude intermediate was added, and then TBAF (0.5 mmol, 0.4 equiv, 1 M solution in THF) dissolved in 1.0 mL DCM was dropped slowly under argon atmosphere. The reaction mixture was stirred at 60 °C for 15 min. After the starting material was completely consumed, ice water (5 mL) was slowly added to quench the reaction. The reaction mixture was extracted with DCM (3 × 5 mL). The organic layers were combined and dried over anhydrous $MgSO_4$. After removal of the solvent in vacuo, the residue was purified by flash chromatography on silica gel (PE/EA) to afford the corresponding product **9aa** as a white solid (125.6 mg, 56% yield).

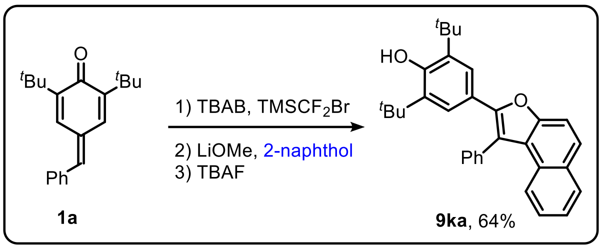

2,6-di-tert-butyl-4-(1-phenylnaphtho[2,1-b]furan-2-yl)phenol (15) To an oven-dried 10-mL Schlenk tube equipped with a Teflon coated magnetic stir bar was added *p*-QM **1a** (147.2 mg, 0.5 mmol, 1.0 equiv) and TBAB (8 mg, 0.025 mmol, 5 mol%), then the Schlenk tube was evacuated and filled with argon for three times. After that, $TMSCF_2Br$ (203.1 mg, 1.0 mmol, 2.0 equiv) dissolved in toluene (1.0 mL) was added under argon atmosphere via a syringe. The reaction mixture was stirred at 80 °C in oil bath for 18 h to afford the toluene solution of crude intermediate. To another oven-dried 10-mL Schlenk tube equipped with a Teflon coated magnetic stir bar was added LiOMe (75.8 mg, 2.0 mmol, 4.0 equiv), then the Schlenk tube was evacuated and filled with argon for three times. After that, anhydrous DCM (0.5 mL) and 2-naphthol (1.0 mmol, 2.0

equiv, dissolved in 0.5 mL DCM) was added under argon atmosphere via a syringe. After stirred for 30 min at room temperature, the toluene solution of crude intermediate was added, and then TBAF (0.2 mmol, 0.4 equiv, dissolved in 1.0 mL DCM) was dropped slowly under argon atmosphere stirred for 15 min at room temperature. When the starting material was completely consumed, saturated solution of NH_4Cl (5 mL) was slowly added to quench the reaction. The reaction mixture was extracted with DCM (3 × 10 mL). The organic layers were combined and dried over anhydrous $MgSO_4$. After removal of the solvent in vacuo, the residue was purified by flash chromatography on silica gel (PE/EA) to afford the corresponding product **9ka** as a purple solid (143.6 mg, 64% yield).

10. Transformations of the Products

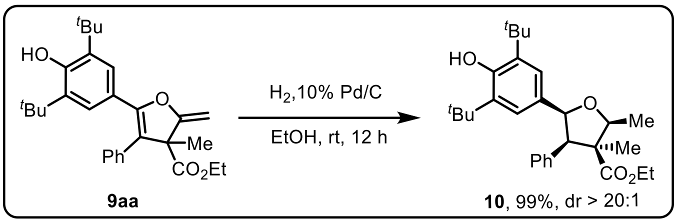

ethyl (2S,3S,4R,5S)-5-(3,5-di-tert-butyl-4-hydroxyphenyl)-2,3-dimethyl-4-phenyltetrahydrofuran-3-carboxylate (10) To an oven-dried 50 mL two necked bottle equipped with a Teflon coated magnetic stir bar was added **9aa** (89.7 mg, 0.2 mmol, 1.0 equiv), 10% Pd/C (17.9 mg, 20% wt) and 2 mL EtOH. The bottle was evacuated and filled with H_2 for three times then stirred at room temperature under H_2 atmosphere for 12 h. After **9aa** was completely consumed, the reaction mixture was filtered through a pad of celite and concentrated under reduced pressure. Purification by flash chromatography on silica gel (PE/EA) to afford **10** as a white solid (89.5 mg, 99% yield). **m.p.** 106 – 107 °C, R_f = 0.3 (PE/EA = 20/1). **^{1}H NMR (300 MHz, $CDCl_3$)** δ 6.92 – 6.84 (m, 3H), 6.77 (s, 2H), 6.57 (d, J = 7.3 Hz, 2H), 5.25 (d, J = 8.2 Hz, 1H), 4.88 (s, 1H), 3.95 – 3.89 (m, 1H), 3.80 – 3.61 (m, 2H), 3.53 (d, J = 8.9 Hz, 1H), 1.47 (d, J = 6.5 Hz, 3H), 1.32 (s, 3H), 1.20 (s, 18H), 0.90 (t, J = 6.8 Hz, 3H) ppm. **^{13}C NMR (75 MHz, $CDCl_3$)** δ 172.2, 152.2, 136.9, 134.5, 130.9, 129.7, 127.0, 126.3, 123.7, 83.3, 81.5, 63.5, 59.8, 58.0, 34.2, 30.2, 23.4, 15.3, 14.0 ppm. **HRMS (ESI)** m/z Calcd for $[C_{29}H_{40}O_4 + Na]^+$ 475.2819, found 475.2834.

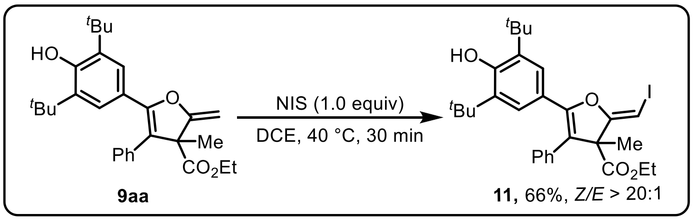

ethyl (Z)-5-(3,5-di-tert-butyl-4-hydroxyphenyl)-2-(iodomethylene)-3-methyl-4-phenyl-2,3-dihydrofuran-3-carboxylate (11) To an oven-dried 10 mL Schlenk tube equipped with a Teflon coated magnetic stir bar was added NIS (45.0 mg, 0.2 mmol, 1.0 equiv) and **9aa** (89.7 mg, 0.2 mmol, 1.0 equiv). Then the Schlenk tube was evacuated and filled with argon for three times. After that, anhydrous DCE (2 mL) was added under argon atmosphere via a syringe under argon atmosphere stirred for 30 min at 40 °C. After **9aa** was completely consumed, the mixture was concentrated and the residue was purified by chromatography on silica gel (PE/EA) to afford **11** as a white solid (75.8 mg, 66% yield, *Z/E* > 20:1). **m.p.** 170 – 172 °C, R_f = 0.4 (PE/EA = 20/1). **^{1}H NMR (300 MHz, $CDCl_3$)** δ 7.35 – 7.21 (m, 7H), 5.33 (s, 1H), 5.19 (s, 1H), 4.23 – 4.16 (m, 2H), 1.55 (s, 3H), 1.28 – 1.22 (m, 22H) ppm. **^{13}C NMR (75 MHz, $CDCl_3$)** δ 171.4, 163.9, 154.8, 150.3, 135.6, 133.4, 130.5, 128.9, 127.9, 124.6, 120.1, 115.4, 62.0, 61.0, 44.5, 34.4, 30.1, 23.6, 14.2 ppm. **HRMS (ESI)** m/z Calcd for $[C_{29}H_{35}IO_4 + H]^+$ 575.1653, found 575.1636.

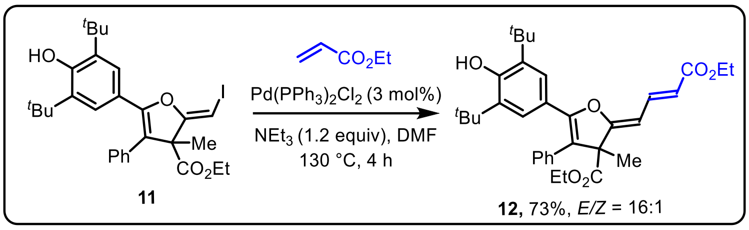

ethyl (Z)-5-(3,5-di-tert-butyl-4-hydroxyphenyl)-2-((E)-4-ethoxy-4-oxobut-2-en-1-ylidene)-3-methyl-4-phenyl-2,3-dihydrofuran-3-carboxylate (12) To an oven-dried 10 mL Schlenk tube equipped with a Teflon coated magnetic stir bar was added **11** (114.9 mg, 0.2 mmol, 1.0 equiv) and $Pd(PPh_3)_2Cl_2$ (4.2 mg, 0.006 mmol, 3 mol%). Then the Schlenk tube was evacuated and filled with argon for three times. After that, ethyl acrylate (56.1 mg, 0.56 mmol, 2.8 equiv) and NEt_3 (24.3 mg, 0.24 mmol, 1.2 equiv) in anhydrous DMF (2 ml) was added via a syringe under argon atmosphere. After stirring for 4 h at 130 °C, the reaction mixture was filtered through a pad of celite and concentrated under reduced pressure. Purification by flash chromatography on silica gel (PE/EA) to afford **12** as a white solid (79.6 mg, 73% yield, *E/Z* = 16:1). **m.p.** 167 – 169 °C, R_f = 0.2 (PE/EA = 20/1). **^{1}H NMR (300 MHz, $CDCl_3$)** δ 7.86 (dd, *J* = 15.5, 11.3 Hz, 1H), 7.38 – 7.34 (m, 2H), 7.31 (s, 2H), 7.28 – 7.20 (m, 3H), 5.89 (d, *J* = 15.5 Hz, 1H), 5.51 (d, *J* = 11.4 Hz, 1H), 5.38 (s, 1H), 4.28 – 4.18 (m, 4H), 1.59 (s, 3H), 1.35 – 1.23 (m, 6H), 1.31 (s, 18H) ppm. **^{13}C NMR (75 MHz, $CDCl_3$)** δ 171.5, 167.6, 165.1, 154.9, 150.6, 138.4, 135.7, 132.9, 130.4, 129.0, 128.0, 124.5, 120.0, 117.6, 114.8, 98.8, 62.0, 61.1, 60.2, 34.4, 30.1, 23.1, 14.4, 14.2 ppm. **HRMS (ESI)** *m/z* Calcd for $[C_{34}H_{42}O_6 + H]^+$ 547.3055, found 547.3058.

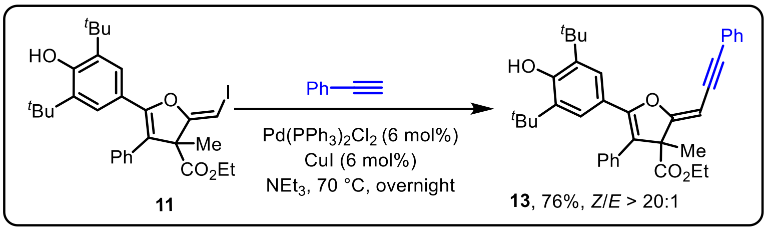

ethyl (Z)-5-(3,5-di-tert-butyl-4-hydroxyphenyl)-3-methyl-4-phenyl-2-(3-phenylprop-2-yn-1-ylidene)-2,3-dihydrofuran-3-carboxylate (13) To an oven-dried 10 mL Schlenk tube equipped with a Teflon coated magnetic stir bar was added **11** (114.9 mg, 0.2 mmol, 1.0 equiv), $Pd(PPh_3)_2Cl_2$ (8.4 mg, 0.012 mmol, 6 mol%), and CuI (2.3 mg, 0.012 mmol, 6 mol%). Then the Schlenk tube was evacuated and filled with argon for three times. After that, phenylacetylene (24.5 mg, 0.24 mmol, 1.2 equiv) in anhydrous NEt_3 (2 mL) was added via a syringe under argon atmosphere. After stirring at 70 °C overnight, the reaction mixture was filtered through a pad of celite and concentrated under reduced pressure. Purification by flash chromatography on silica gel (PE/EA) to afford **13** as a white solid (83.4 mg, 76% yield, *Z/E* > 20:1). **m.p.** 142– 144 °C, R_f = 0.3 (PE/EA = 20/1). **^{1}H NMR (300 MHz, $CDCl_3$)** δ 7.49 – 7.46 (m, 2H), 7.39 – 7.23 (m, 10H), 5.33 (s, 1H), 5.04 (s, 1H), 4.29 – 4.15 (m, 2H), 1.58 (s, 3H), 1.30 – 1.23 (m, 21H) ppm. **^{13}C NMR (75 MHz, CDCl3)** δ 171.6, 168.3, 154.8, 150.6, 135.6, 133.2, 131.3, 130.5, 129.0, 128.4, 128.0, 127.8, 124.6, 124.3, 120.2, 114.8, 94.5, 84.3, 80.8, 62.0, 61.0, 34.4, 30.1,

23.6, 14.3 ppm. **HRMS (ESI)** *m/z* Calcd for $[C_{37}H_{41}O_4 + H]^+$ 549.3000, found 549.2991.

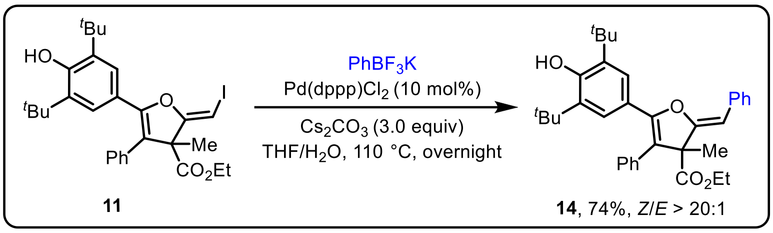

ethyl (Z)-2-benzylidene-5-(3-(tert-butyl)-4-hydroxy-5-methylphenyl)-3-methyl-4-phenyl-2,3-dihydrofuran-3-carboxylate (14) To an oven-dried 10 mL Schlenk tube equipped with a Teflon coated magnetic stir bar was added **11** (114.9 mg, 0.2 mmol, 1.0 equiv), Cs_2CO_3 (211.7 mg, 0.6 mmol, 3.0 equiv), $PhBF_3K$ (44.2 mg, 0.24 mmol, 1.2 equiv), and $Pd(dppp)Cl_2$ (11.8 mg, 0.02 mmol, 10 mol%). Then the Schlenk tube was evacuated and filled with argon for three times. After that, THF (2.0 mL) and H_2O (0.6 mL) were added under argon atmosphere via a syringe. After stirred at 110 °C overnight, the reaction was quenched with brine and extracted with EA (3 × 4 mL). The combined organic phase was washed with brine, dried over $MgSO_4$, evaporated to give the crude products. The residue was purified by flash chromatography (PE/EA) to afford **14** as a yellow solid (77.7 mg, 74% yield, *Z/E* > 20:1). **m.p.** 163 – 165 °C, R_f = 0.3 (PE/EA = 20/1). **^{1}H NMR (300 MHz, CDCl₃)** δ 7.76 – 7.73 (m, 2H), 7.38 – 7.16 (m, 10H), 5.58 (s, 1H), 5.33 (s, 1H), 4.29 – 4.11 (m, 2H), 1.62 (s, 3H), 1.30 (s, 18H), 1.23 (t, *J* = 7.1 Hz, 3H) ppm. **^{13}C NMR (75 MHz, $CDCl_3$)** δ 172.5, 158.3, 154.7, 150.6, 135.7, 133.5, 130.6, 128.9, 128.4, 128.3, 127.8, 126.0, 124.5, 120.6, 114.3, 100.5, 61.8, 61.5, 34.4, 30.1, 23.8, 14.3 ppm. **HRMS (ESI)** *m/z* Calcd for $[C_{35}H_{40}O_4 + H]^+$ 525.3000, found 525.2998.

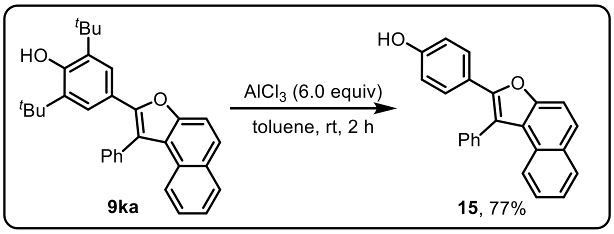

4-(1-phenylnaphtho[2,1-b]furan-2-yl)phenol (15) To an oven dried 10 mL Schlenk tube equipped with a Teflon coated magnetic stir bar was added $AlCl_3$ (160.0 mg, 1.2 mmol, 6.0 equiv). Then the Schlenk tube was evacuated and filled with argon for three times. After that, **9ka** (89.7 mg, 0.2 mmol, 1.0 equiv) dissolved in toluene (4.0 mL) was added. The reaction was stirred for 2 h under at room temperature. After complete consumption of **9ka**, ice-water was added to quench the reaction. The reaction mixture was extracted with EA (3 × 10 mL). The organic layers were combined and dried over anhydrous $MgSO_4$, filtered, and concentrated in vacuo. The residue was purified by flash chromatography on silica gel (PE/EA) to afford product **15** as yellow oil (51.8 mg, 77% yield), R_f = 0.3 (PE/EA = 5/1). **^{1}H NMR (300 MHz, $CDCl_3$)** δ 7.85 (d, *J* = 7.7 Hz, 1H), 7.65 (*app.* s, 2H), 7.54 – 7.48 (m, 6H), 7.41 – 7.30 (m, 3H), 7.24 – 7.19 (m, 1H),

6.65 (d, J = 8.7 Hz, 2H), 5.52 (s, 1H) ppm. **^{13}C NMR (75 MHz, CDCl$_3$)** δ 155.3, 151.2, 150.3, 134.9, 131.0, 130.8, 129.5, 129.1, 128.4, 128.2, 128.0, 126.0, 125.6, 124.3, 124.0, 123.8, 123.2, 118.1, 115.6, 112.3 ppm. **HRMS (ESI)** *m/z* Calcd for $[C_{24}H_{16}O_2 + H]^+$ 337.1223, found 337.1225.

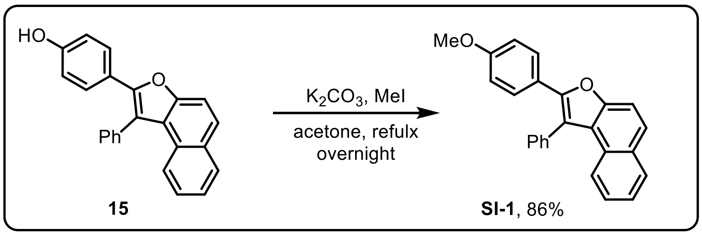

2-(4-methoxyphenyl)-1-phenylnaphtho[2,1-b]furan (SI-1) To an oven-dried 10 mL Schlenk tube equipped with a Teflon coated magnetic stir bar was added **15** (67.3 mg, 0.2 mmol, 1.0 equiv) and K_2CO_3 (138.2 mg, 1.0 mmol, 5.0 equiv). Then the Schlenk tube was evacuated and filled with argon for three times. After that, MeI (56.8 mg, 0.4 mmol, 2.0 equiv) dissolved in acetone (2 mL) was added under argon atmosphere via a syringe under argon atmosphere stirred at 70 °C overnight. When the starting material was completely consumed, water was slowly added to quench the reaction. The reaction mixture was extracted with EA (3 × 10 mL). The organic layers were combined and dried over anhydrous $MgSO_4$. After removal of the solvent in vacuo, the residue was purified by flash chromatography on silica gel (PE/EA) to afford **SI-1** as a white solid (60.1 mg, 86% yield). **m.p.** 110 – 111 °C, R_f = 0.4 (PE/EA = 5/1). **^{1}H NMR (300 MHz, CDCl$_3$)** δ 7.88 (d, J = 8.1 Hz, 1H), 7.68 (s, 2H), 7.56 – 7.44 (m, 8H), 7.38 – 7.32 (m, 1H), 7.25 – 7.18 (m, 1H), 6.80 – 6.75 (m, 2H), 3.73 (s, 3H) ppm. **^{13}C NMR (75 MHz, CDCl$_3$)** δ 159.4, 151.2, 150.4, 135.0, 131.0, 130.9, 130.8, 129.6, 129.5, 129.5, 129.0, 128.3, 128.2, 127.8, 127.7, 125.9, 125.5, 124.3, 123.8, 123.7, 123.2, 118.1, 114.0, 112.2, 55.3 ppm. **HRMS (ESI)** *m/z* Calcd for $[C_{25}H_{18}O_2 + H]^+$ 351.1380, found 351.1379. Product **SI-1** is a known compound. The regioselectivity in forming **9ka** was unambiguously established by NMR spectral congruence with reported data.[2]

[2] V. K. Rao, G. M. Shelke, R. Tiwari, K. Parang and A. Kumar, *Org. Lett.* **2013**, *15*, 2190.

11. Control Experiments

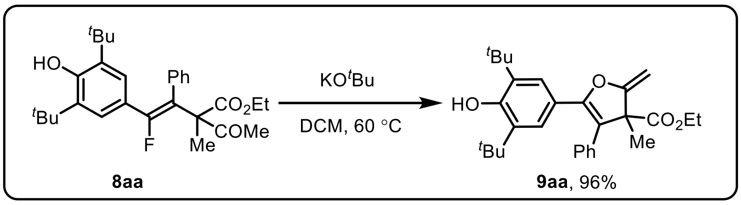

To an oven-dried 10 mL Schlenk tube equipped with a Teflon coated magnetic stir bar was added ^{t}BuOK (89.8 mg, 0.8 mmol, 4.0 equiv) and **8aa** (99.7 mg, 0.2 mmol, 1.0 equiv) then the Schlenk tube was evacuated and filled with argon for three times. After that, anhydrous DCM (2 mL) was added under argon atmosphere via a syringe under argon atmosphere stirred for 15 min at 60 °C. After the starting material was completely consumed, ice water (5 mL) was slowly added to quench the reaction. The reaction mixture was extracted with DCM (3 × 10 mL). The organic layers were combined and dried over anhydrous $MgSO_4$. After removal of the solvent in vacuo, the residue was purified by flash chromatography on silica gel (PE/EA) to afford the corresponding product **9aa** as a white solid (86.0 mg, 96% yield).

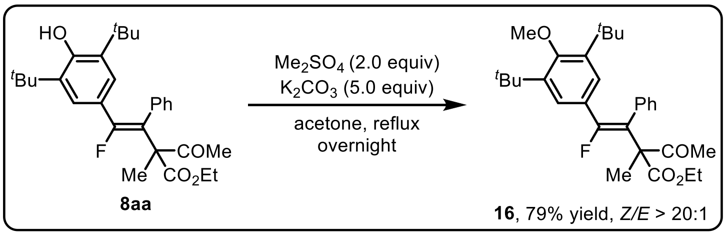

To an oven-dried 10 mL Schlenk tube equipped with a Teflon coated magnetic stir bar was added **8aa** (93.7 mg, 0.2 mmol, 1.0 equiv) and K_2CO_3 (138.2 mg, 1.0 mmol, 5.0 equiv). Then the Schlenk tube was evacuated and filled with argon for three times. After that, Me_2SO_4 (50.5 mg, 0.4 mmol, 2.0 equiv) dissolved in acetone (2 mL) was added under argon atmosphere via a syringe under argon atmosphere stirred at 70 °C overnight. When the starting material was completely consumed, water was slowly added to quench the reaction. The reaction mixture was extracted with EA (3 × 10 mL). The organic layers were combined and dried over anhydrous $MgSO_4$. After removal of the solvent in vacuo, the residue was purified by flash chromatography on silica gel (PE/EA) to afford **16** as a white solid (76.2 mg, 79% yield, *Z/E* > 20:1). **m.p.** 131 – 133 °C, R_f = 0.6 (PE/EA = 10/1). **^{1}H NMR (300 MHz, $CDCl_3$)** δ 7.37 – 7.24 (m, 3H), 7.19 – 7.16 (m, 2H), 7.01 (s, 2H), 4.10 – 3.98 (m, 2H), 3.60 (s, 3H), 2.48 (d, *J* = 1.9 Hz, 3H), 1.48 (s, 3H), 1.20 (s, 19H), 1.14 (t, *J* = 7.1 Hz, 3H) ppm. **^{13}C NMR (75 MHz, $CDCl_3$)** δ 204.7 (d, ^{4}J = 2.3 Hz), 171.4, 160.1, 156.1 (d, ^{1}J = 250.1 Hz), 143.2, 136.1 (d, ^{3}J = 8.6 Hz), 130.4 (d, ^{4}J = 3.0 Hz), 128.9, 128.0, 126.6 (d, ^{3}J = 7.5 Hz), 125.7 (d, ^{2}J = 28.0 Hz), 119.2 (d, ^{2}J = 19.4 Hz), 64.2, 64.0, 61.6, 35.7, 31.8, 27.3 (d, ^{4}J = 4.6 Hz), 20.9 (d, ^{4}J = 1.6 Hz), 13.9 ppm. **^{19}F NMR (282 MHz, $CDCl_3$)** δ -96.3 ppm. **HRMS (ESI)** *m/z* Calcd for $[C_{30}H_{39}FO_4 + Na]^+$ 505.2725, found 505.2727.

12. Calculation Studies

To validate bond order quantification, semi-empirical quantum mechanical (QM) calculations were performed using the PM7 Hamiltonian as implemented in MOPAC v22.1.1 (Digital Object Identifier (DOI): 10.5281/zenodo.6511958). Initial molecular geometries were defined through Cartesian coordinates, with explicit generation of Mayer bond order matrices enabled by the BONDS keyword. All geometry optimizations were conducted under gas-phase conditions to simulate isolated molecular environments.

The computational results are presented as follows.

Intermediate I (INT-I)

C	-0.00000038	0.01144502	0.00234211
C	2.09269800	1.21919308	-0.00000046
C	1.39504438	2.42769771	-0.00354261
C	0.00021938	2.42761922	-0.00402101
C	-0.69738200	1.21941765	-0.00068200
H	-0.54975692	-0.94087567	0.00044960
H	3.19237342	1.21927944	0.00482213
O	-0.71509425	3.66584907	-0.00766095
C	-2.23738196	1.21967394	-0.00093409
C	-2.71308564	1.94172337	1.24194665
C	-2.71281788	1.93482693	-1.24784514
C	-2.71316312	-0.21766301	0.00296366
H	-2.33913584	1.42529621	2.15841942
H	-2.33838435	2.99339448	1.24774255
H	-3.82888196	1.96398262	1.27935692
H	-2.33823551	1.41374577	-2.16142981
H	-3.82862522	1.95635430	-1.28582080
H	-2.33862626	2.98664312	-1.25922127
H	-3.82899581	-0.26104966	0.00346840
H	-2.33912787	-0.75323909	-0.90241572
H	-2.33860715	-0.74847827	0.91093958
C	2.16554888	3.76108276	-0.00621100
C	1.78218598	4.52943596	-1.25323472
C	1.78066425	4.53545807	1.23655888
C	3.64804661	3.45364488	-0.00452897
H	2.04483861	3.94366483	-2.16665881
H	0.68419383	4.73133546	-1.26306274
H	2.32145066	5.50643129	-1.29286009
H	2.04162095	3.95394379	2.15319252
H	2.32034527	5.51239051	1.27232182
H	0.68275082	4.73793835	1.24390106
H	4.24405320	4.39796238	-0.00695329

H	3.92185032	2.86531815	0.90396380
H	3.92282722	2.86018141	-0.90939029
C	1.39515962	0.01144458	0.00234166
C	2.04664035	-1.11490464	-0.74616120
C	2.04411989	-1.11357837	0.75493997
H	1.41112866	-1.84072875	-1.28314242
F	1.26471187	-1.99969824	1.41054253
F	3.19997999	-0.88184161	1.41280433
C	3.36779261	-0.85214179	-1.49256083
C	3.34534058	-0.38612883	-2.80739894
C	4.58683438	-1.07991601	-0.85412194
C	4.54170803	-0.14861393	-3.48379625
H	2.38410696	-0.20738771	-3.31071129
C	5.78365748	-0.84143958	-1.53025310
H	4.60471337	-1.44703666	0.18223311
C	5.76128801	-0.37598971	-2.84493673
H	4.52409000	0.21810517	-4.52037872
H	6.74466839	-1.02072786	-1.02647937
H	6.70445212	-0.18859034	-3.37843392

Atomic orbital electron populations

	Atom	s	px	py	pz
1	C	1.08707	0.96466	1.08074	1.00091
2	C	1.08926	1.09612	0.95474	0.98780
3	C	1.10260	1.00651	1.01235	1.00362
4	C	1.11508	0.88846	0.85767	0.67963
5	C	1.10414	1.02353	0.99565	0.99395
6	H	0.83158			
7	H	0.82287			
8	O	1.87871	1.72964	1.44210	1.39942
9	C	1.04659	0.90773	0.94832	0.96041
10	C	1.05918	1.15276	1.16978	1.08133
11	C	1.05962	1.16652	1.16255	1.07665
12	C	1.06070	1.16325	1.07476	1.16955
13	H	0.85437			
14	H	0.82835			
15	H	0.85560			
16	H	0.85728			
17	H	0.85486			
18	H	0.83095			
19	H	0.85134			
20	H	0.85607			
21	H	0.85324			
22	C	1.04624	0.93256	0.92071	0.95805

23	C	1.05931	1.18351	1.14378	1.07665
24	C	1.05913	1.19082	1.13089	1.08159
25	C	1.06070	1.05379	1.18125	1.17265
26	H	0.85797			
27	H	0.83354			
28	H	0.85520			
29	H	0.85400			
30	H	0.85646			
31	H	0.83012			
32	H	0.85045			
33	H	0.85225			
34	H	0.85520			
35	C	1.04935	0.99300	0.95344	1.08859
36	C	1.06769	1.07998	1.07754	0.99898
37	C	1.09675	0.80899	0.90580	0.85537
38	H	0.80461			
39	F	1.96710	1.78153	1.65448	1.75621
40	F	1.96685	1.47258	1.95075	1.76152
41	C	1.05723	0.95407	1.00333	0.98701
42	C	1.08491	1.06116	1.01514	0.99000
43	C	1.08449	0.98300	1.03620	1.04892
44	C	1.08358	0.98641	1.03225	1.04186
45	H	0.83705			
46	C	1.08364	1.05291	1.01249	0.99173
47	H	0.83993			
48	C	1.08420	1.04809	0.99484	1.01933
49	H	0.84830			
50	H	0.84878			
51	H	0.85065			

(Valencies) bond orders

1 C (3.968) 5 C 1.883 35 C 1.025 6 H 0.953 8 O
0.037 4 C 0.013 10 C 0.013
11 C 0.012

2 C (3.965) 3 C 1.881 35 C 1.025 7 H 0.950 8 O
0.038 4 C 0.014 24 C 0.013
23 C 0.012

3 C (3.984) 2 C 1.881 22 C 0.985 4 C 0.976 8 O
0.048 36 C 0.019 5 C 0.019
37 C 0.017

4 C (3.836) 8 O 1.819 3 C 0.976 5 C 0.972 2 C 0.014 1 C 0.013

5 C (3.985) 1 C 1.883 9 C 0.986 4 C 0.972 8 O 0.048 36 C 0.019 3 C 0.019 37 C 0.017

6 H (0.972) 1 C 0.953

7 H (0.969) 2 C 0.950

8 O (2.015) 4 C 1.819 5 C 0.048 3 C 0.048 2 C 0.038 1 C 0.037

9 C (3.984) 12 C 0.994 10 C 0.990 11 C 0.990 5 C 0.986

10 C (3.924) 9 C 0.990 13 H 0.971 15 H 0.970 14 H 0.960 1 C 0.013

11 C (3.925) 9 C 0.990 16 H 0.972 17 H 0.970 18 H 0.961 1 C 0.012

12 C (3.930) 9 C 0.994 20 H 0.971 21 H 0.970 19 H 0.968

13 H (0.979) 10 C 0.971

14 H (0.971) 10 C 0.960

15 H (0.979) 10 C 0.970

16 H (0.980) 11 C 0.972

17 H (0.979) 11 C 0.970

18 H (0.971) 11 C 0.961

19 H (0.978) 12 C 0.968

20 H (0.979) 12 C 0.971

21 H (0.978) 12 C 0.970

22 C (3.983) 25 C 0.995 23 C 0.990 24 C 0.990 3 C 0.985

23 C (3.926) 22 C 0.990 26 H 0.972 28 H 0.970 27 H 0.962 2 C 0.012

24 C (3.925) 22 C 0.990 29 H 0.971 30 H 0.970 31 H 0.961 2 C 0.013

25 C (3.929) 22 C 0.995 34 H 0.970 33 H 0.969 32 H 0.968

26 H (0.980) 23 C 0.972

27 H (0.972) 23 C 0.962

28 H (0.979) 23 C 0.970

29 H (0.979) 24 C 0.971

30 H (0.979) 24 C 0.970

31 H (0.971) 24 C 0.961

32 H (0.978) 25 C 0.968

33 H (0.978) 25 C 0.969

34 H (0.979) 25 C 0.970

35 C (3.977) 1 C 1.025 2 C 1.025 37 C 0.924 36 C 0.913 39 F 0.022 40 F 0.021

36 C (3.949) 41 C 1.005 37 C 0.973 38 H 0.940 35 C 0.913 39 F 0.022 40 F 0.022
5 C 0.019 3 C 0.019

37 C (3.884) 36 C 0.973 40 F 0.966 39 F 0.959 35 C 0.924 3 C 0.017 5 C 0.017

38 H (0.962) 36 C 0.940

39 F (1.054) 37 C 0.959 40 F 0.039 36 C 0.022 35 C 0.022

40 F (1.059) 37 C 0.966 39 F 0.039 36 C 0.022 35 C
0.021

41 C (3.992) 43 C 1.430 42 C 1.415 36 C 1.005 48 C
0.114

42 C (3.973) 44 C 1.452 41 C 1.415 45 H 0.960 46 C
0.112
43 C (3.974) 46 C 1.439 41 C 1.430 47 H 0.961 44 C
0.111

44 C (3.976) 42 C 1.452 48 C 1.434 49 H 0.964 43 C
0.111

45 H (0.973) 42 C 0.960

46 C (3.976) 48 C 1.446 43 C 1.439 50 H 0.964 42 C
0.112

47 H (0.974) 43 C 0.961

48 C (3.977) 46 C 1.446 44 C 1.434 51 H 0.965 41 C
0.114

49 H (0.977) 44 C 0.964

50 H (0.977) 46 C 0.964

51 H (0.978) 48 C 0.965

13. Crystal Structure of Product 10

Vapor diffusion crystallization method was used for crystal growth of **10**. The compound **10** was dissolved in diethyl ether to make saturated solution in small vial and placed in closed bottle with another solvent as *n*-hexane.

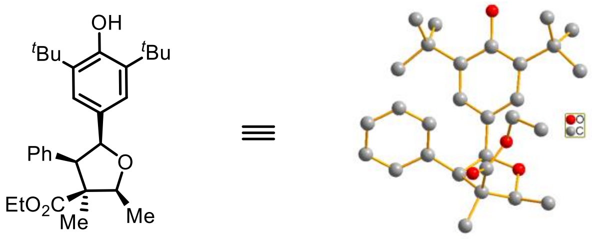

10 **CCDC 2356255**

Figure S1. ORTEP plot of the crystal structure of compound **10** and thermal ellipsoid is set at 50% probability

Table S3 X-ray Crystallographic Data of **10**

CCDC number	2356255
Bond precision	C-C = 0.0050 A Wavelength = 0.71073
Cell	a=10.6394 (6) b=10.7112 (6) c=23.9695 (13)<br>alpha=90.188 (2) beta=102.380 (2) gamma=104.147 (2)
Temperature	170 K
Volume	2582.7 (3)
Space group	P -1
Hall group	-P 1
Sum formula	$C_{29}H_{40}O_4$
Mr	452.61
Dx, g cm-3	1.164
Z	4
Mu (mm-1)	0.076
F000	984.0
F000’	984.44
h, k, lmax	0, 0, 0
Nref	10326
Tmin, Tmax	0.524, 0.856
Tmin’	0.989
Correction method	# Reported T Limits: Tmin=0.524, Tmax=0.856
AbsCorr	MULTI-SCAN
Data completeness	0.971
Theta(max)	26.423
R(reflections)	0.0866 (6371)
wR2(reflections)	wR2 (reflections) =0.2458 (10326)
S	1.049
Npar	615
Ellipsoid contour % probability levels	50

14. ^{1}H, ^{13}C and ^{19}F NMR Spectra of Title Compounds

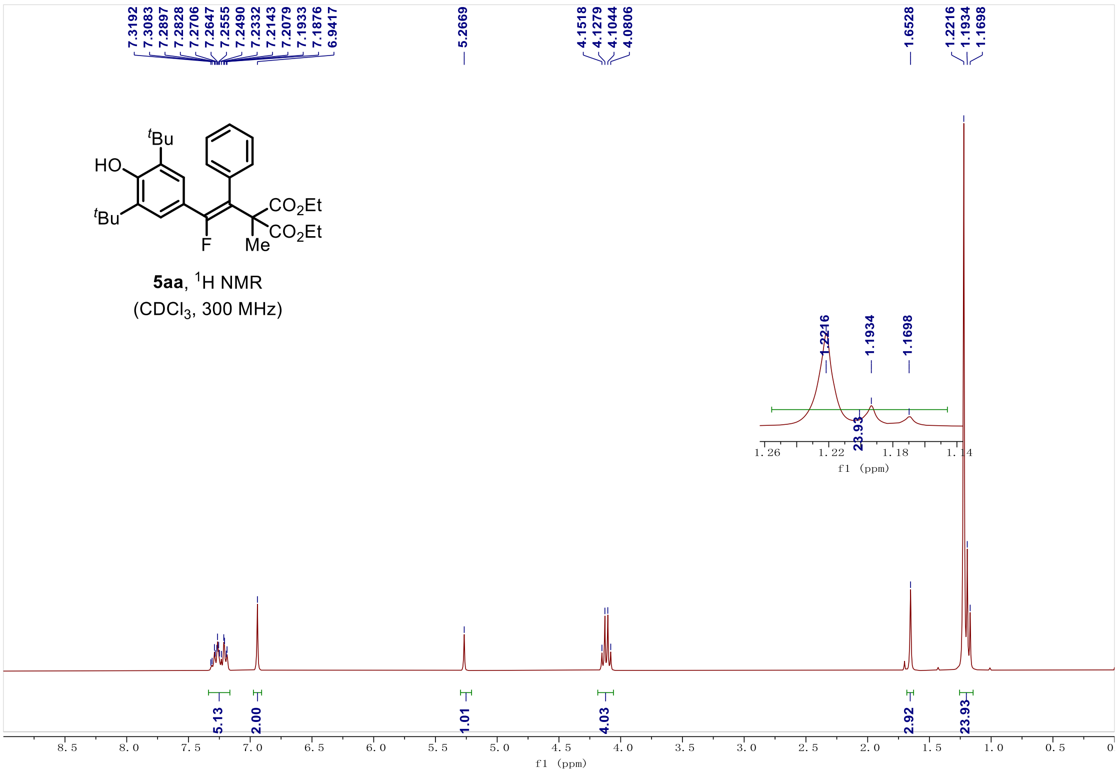

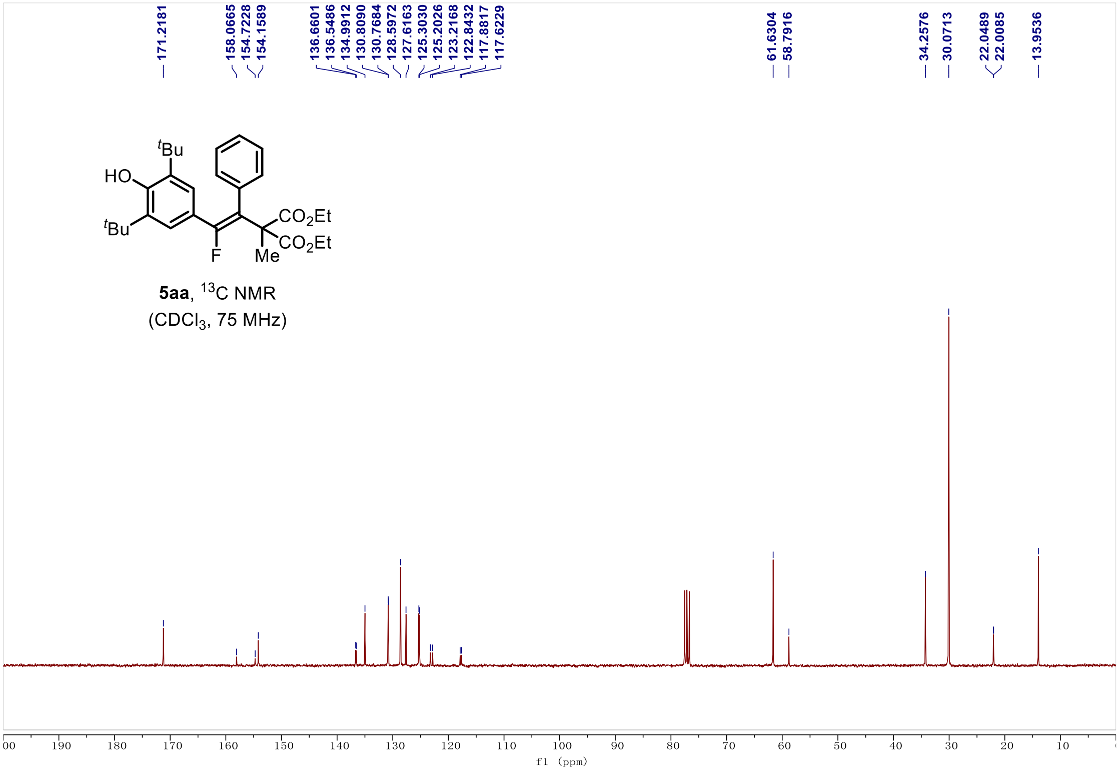

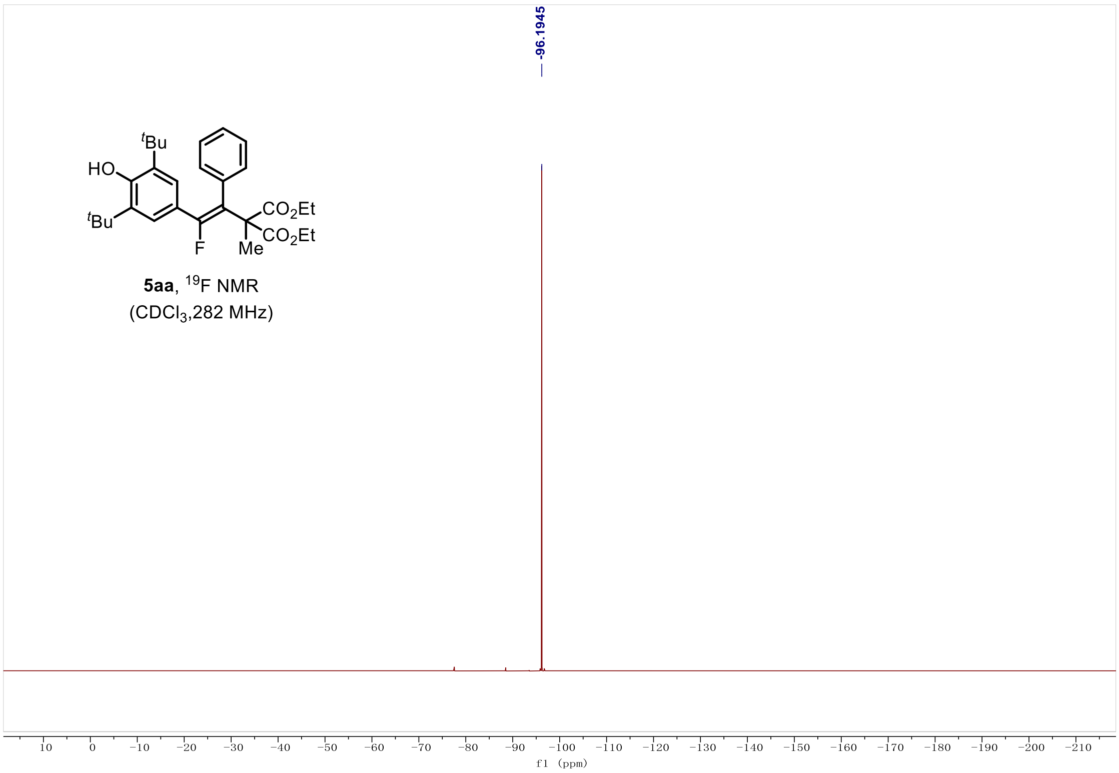
-96.1945
HO
tBu
tBu
F
Me
CO2Et
CO2Et
5aa, 19F NMR
(CDCl3,282 MHz)
f1 (ppm)

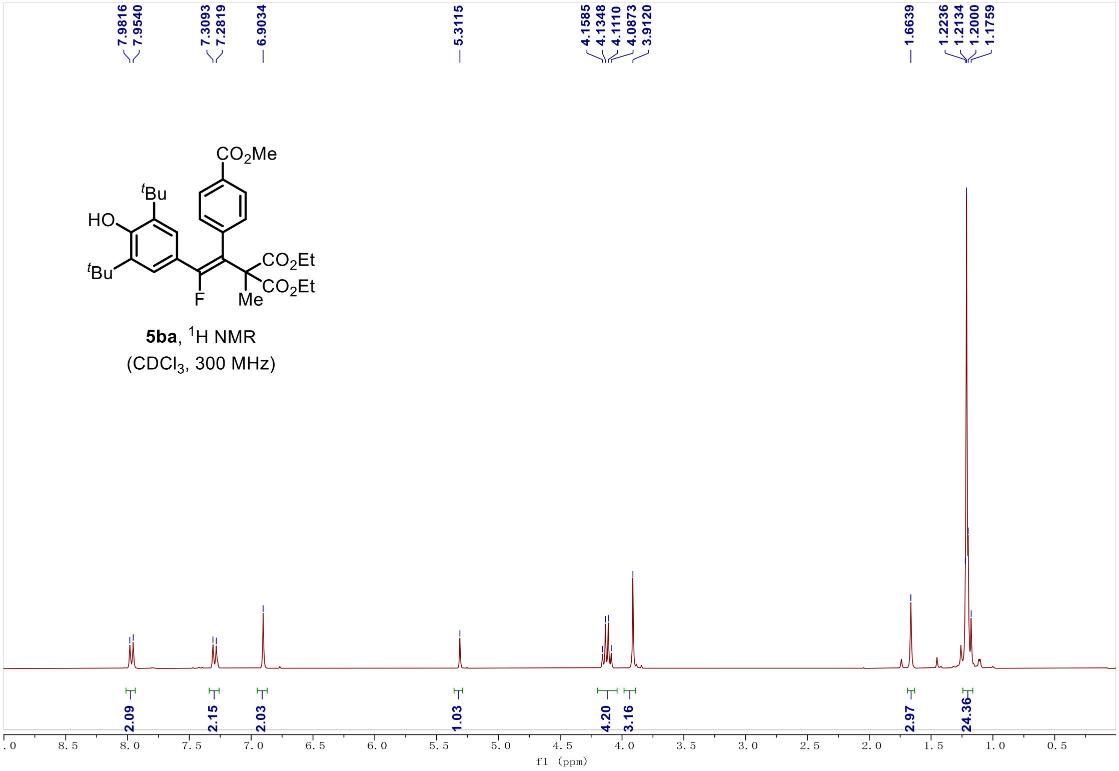
7.9816
7.9540
7.3093
7.2819
6.9034
5.3115
4.1585
4.1348
4.1110
4.0873
3.9120
1.6639
1.2236
1.2134
1.2000
1.1759
CO2Me
HO
tBu
tBu
F
Me
CO2Et
CO2Et
5ba, 1H NMR
(CDCl3, 300 MHz)
2.09
2.15
2.03
1.03
4.20
3.16
2.97
24.36
f1 (ppm)

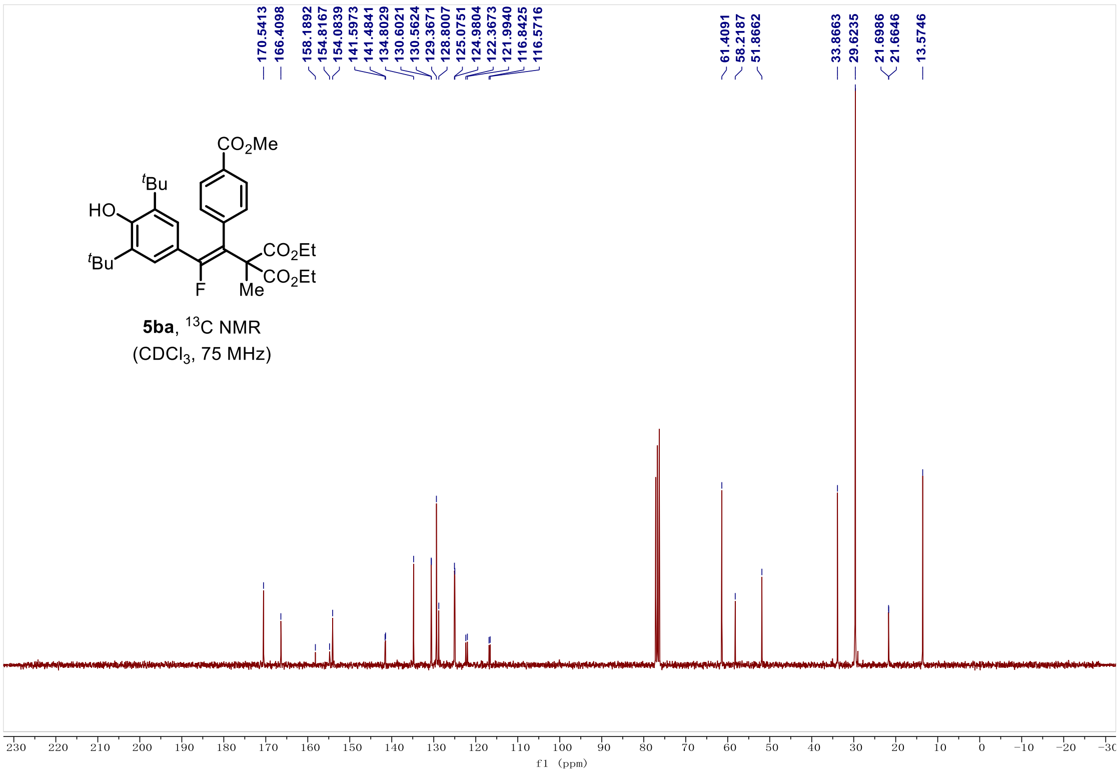
170.5413
166.4098
158.1892
154.8167
154.0839
141.5973
141.4841
134.8029
130.6021
130.5624
129.3671
128.8007
125.0751
124.9804
122.3673
121.9940
116.8425
116.5716
61.4091
58.2187
51.8662
33.8663
29.6235
21.6986
21.6646
13.5746
CO2Me
tBu
HO
tBu
CO2Et
CO2Et
F
Me
5ba, 13C NMR
(CDCl3, 75 MHz)
f1 (ppm)

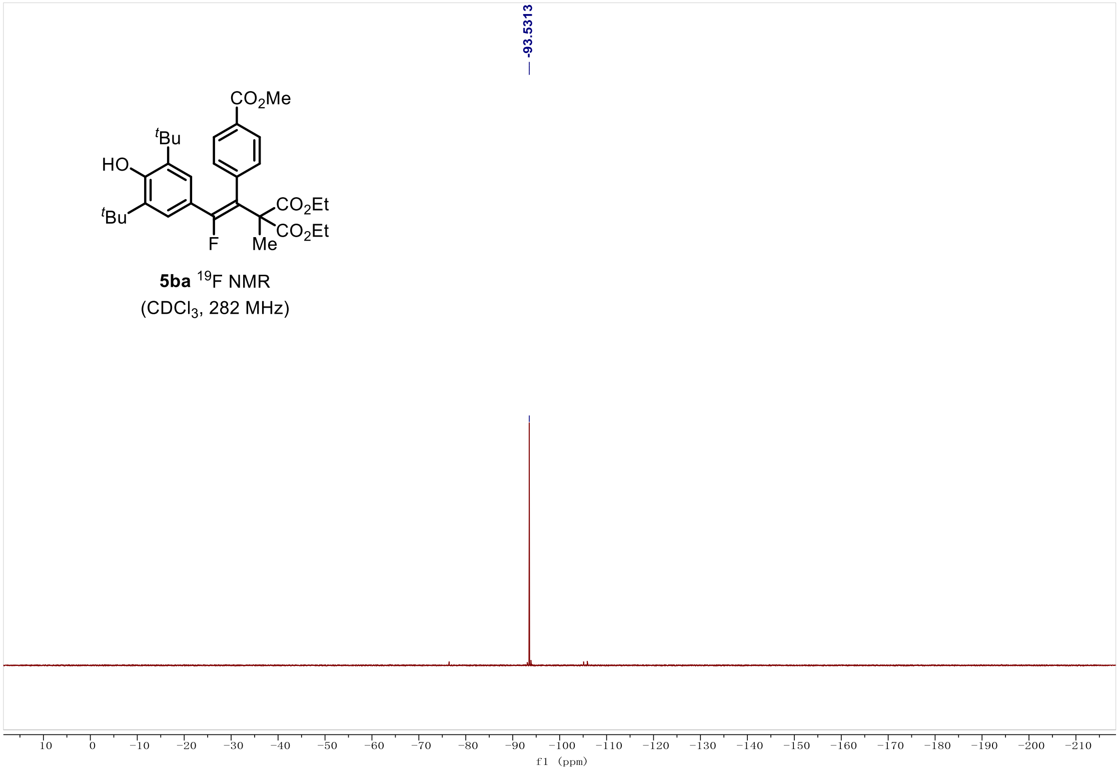
-93.5313
CO2Me
tBu
HO
tBu
CO2Et
CO2Et
F
Me
5ba 19F NMR
(CDCl3, 282 MHz)
f1 (ppm)

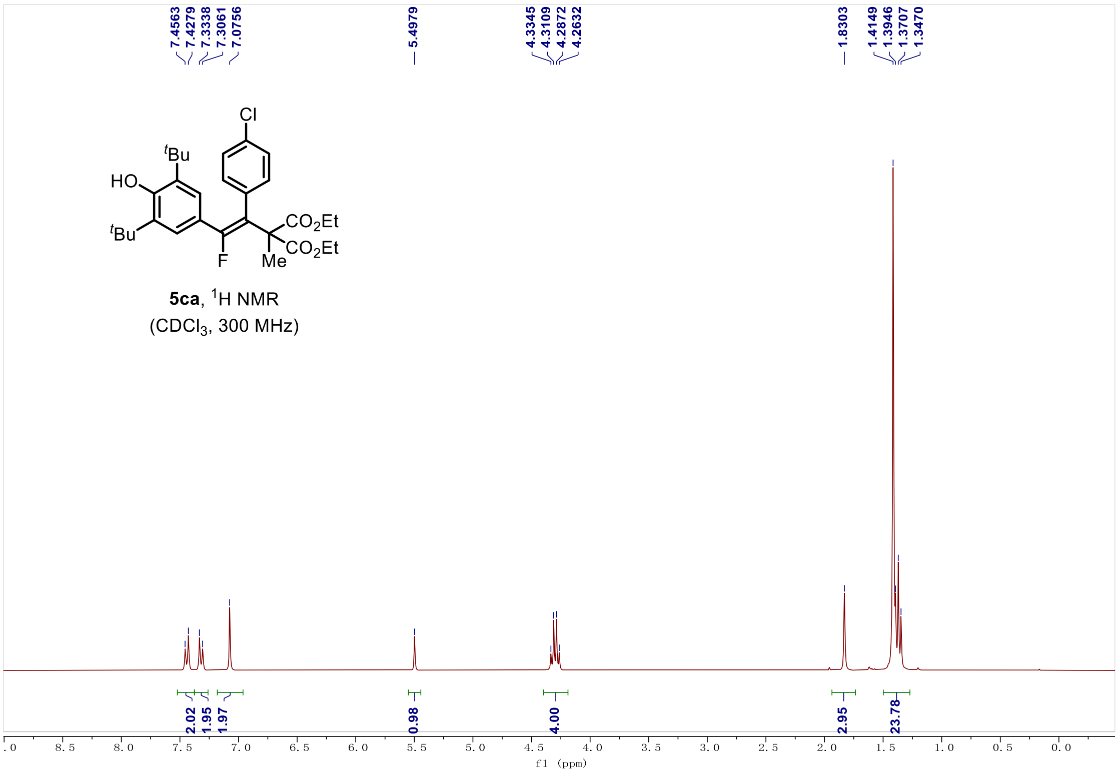
7.4563
7.4279
7.3338
7.3061
7.0756
5.4979
4.3345
4.3109
4.2872
4.2632
1.8303
1.4149
1.3946
1.3707
1.3470
HO
tBu
tBu
Cl
CO2Et
CO2Et
F
Me
5ca, 1H NMR
(CDCl3, 300 MHz)
2.02
1.95
1.97
0.98
4.00
2.95
23.78
f1 (ppm)

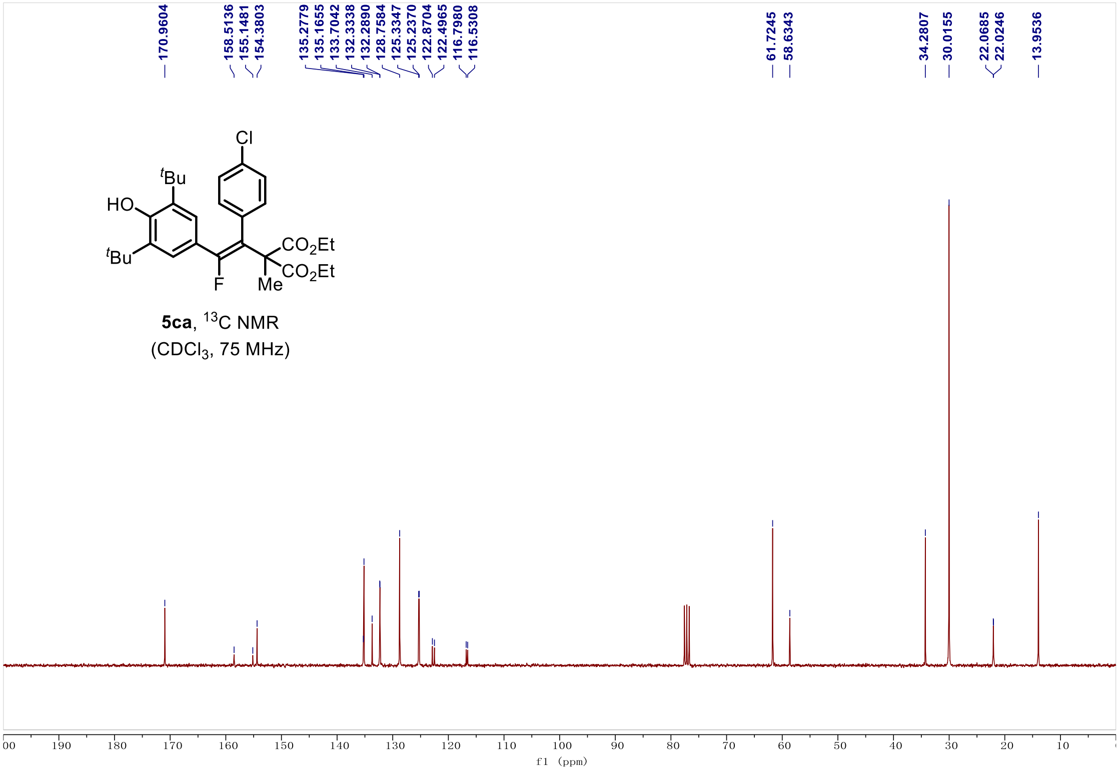
170.9604
158.5136
155.1481
154.3803
135.2779
135.1655
133.7042
132.3338
132.2890
128.7584
125.3347
125.2370
122.8704
122.4965
116.7980
116.5308
61.7245
58.6343
34.2807
30.0155
22.0685
22.0246
13.9536
HO
tBu
tBu
Cl
CO2Et
CO2Et
F
Me
5ca, 13C NMR
(CDCl3, 75 MHz)
f1 (ppm)

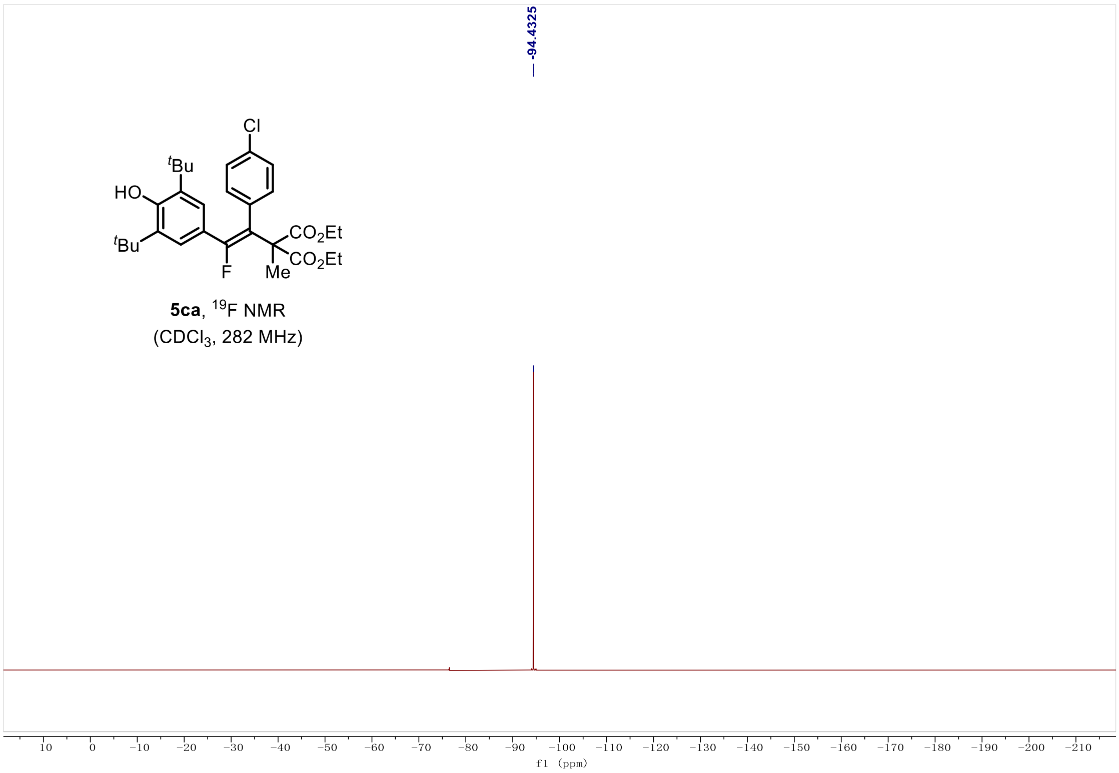

-94.4325
Cl
tBu
HO
tBu
CO2Et
CO2Et
F
Me
5ca, 19F NMR
(CDCl3, 282 MHz)
f1 (ppm)

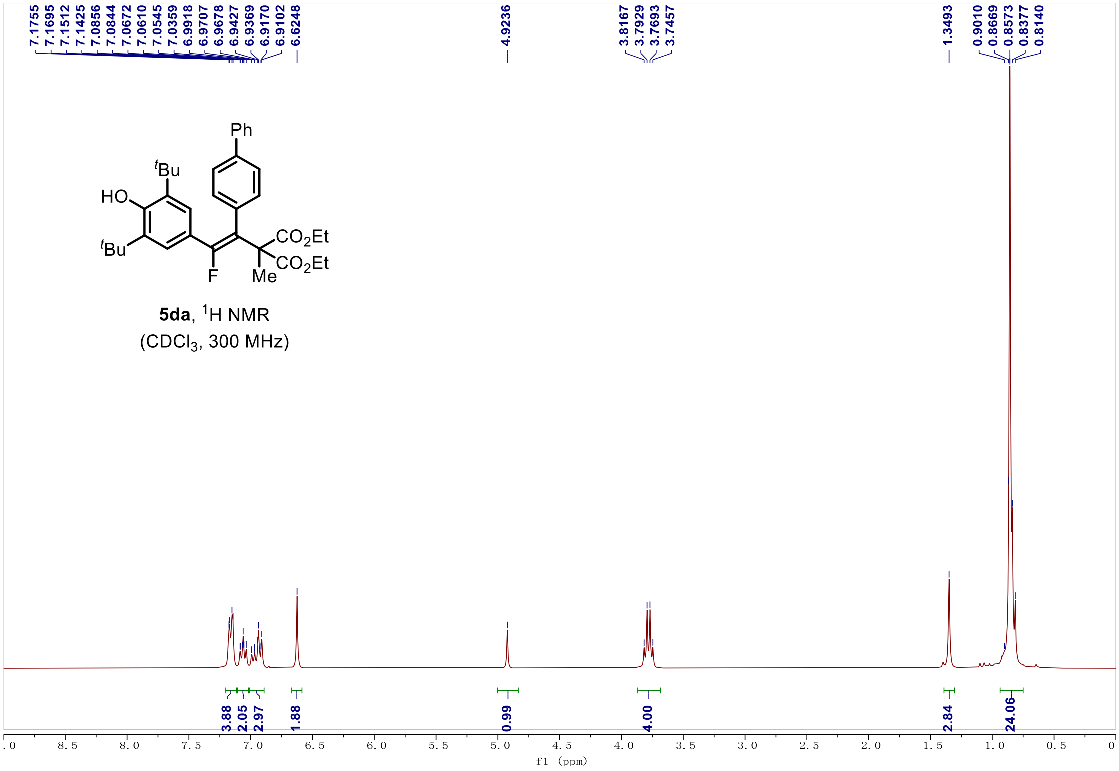

7.1755 7.1695 7.1512 7.1425 7.0856 7.0844 7.0672 7.0610 7.0545 7.0359 6.9918 6.9707 6.9678 6.9427 6.9369 6.9170 6.9102 6.6248
4.9236
3.8167 3.7929 3.7693 3.7457
1.3493
0.9010 0.8669 0.8573 0.8377 0.8140
Ph
tBu
HO
tBu
CO2Et
CO2Et
F
Me
5da, 1H NMR
(CDCl3, 300 MHz)
3.88 2.05 2.97 1.88 0.99 4.00 2.84 24.06
f1 (ppm)

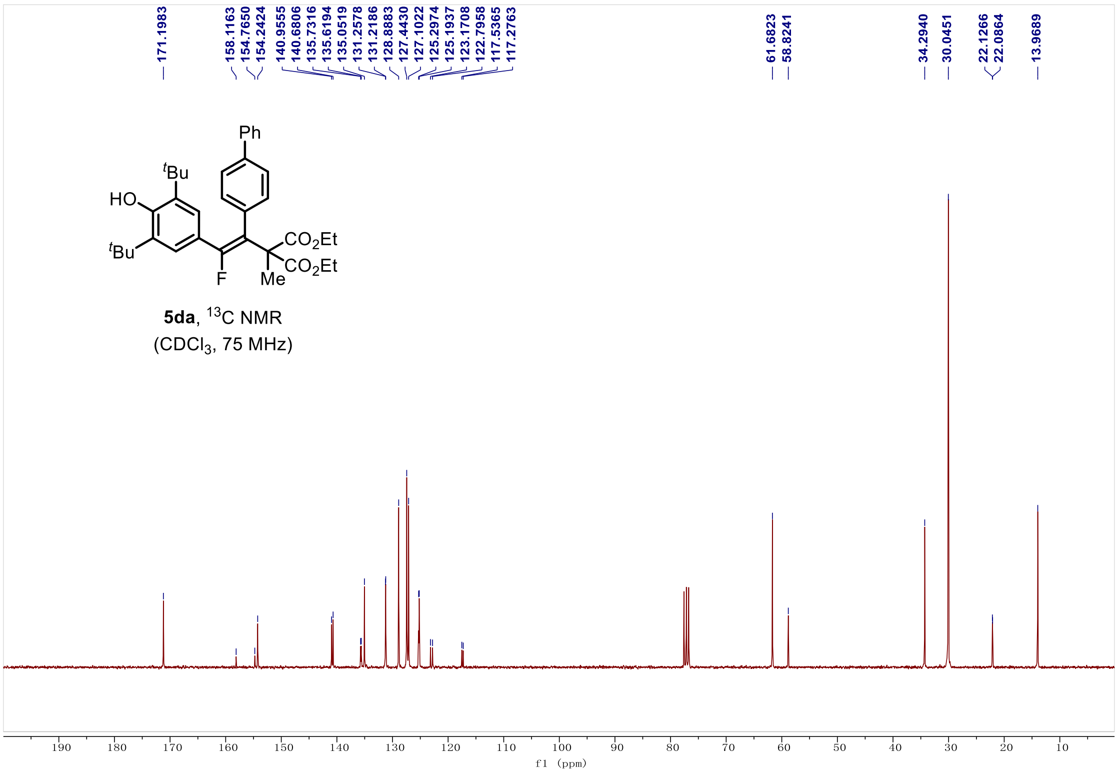
171.1983
158.1163
154.7650
154.2424
140.9555
140.6806
135.7316
135.6194
135.0519
131.2578
131.2186
128.8883
127.4430
127.1022
125.2974
125.1937
123.1708
122.7958
117.5365
117.2763
61.6823
58.8241
34.2940
30.0451
22.1266
22.0864
13.9689
Ph
HO
tBu
tBu
F
Me
CO2Et
CO2Et
5da, 13C NMR
(CDCl3, 75 MHz)
f1 (ppm)

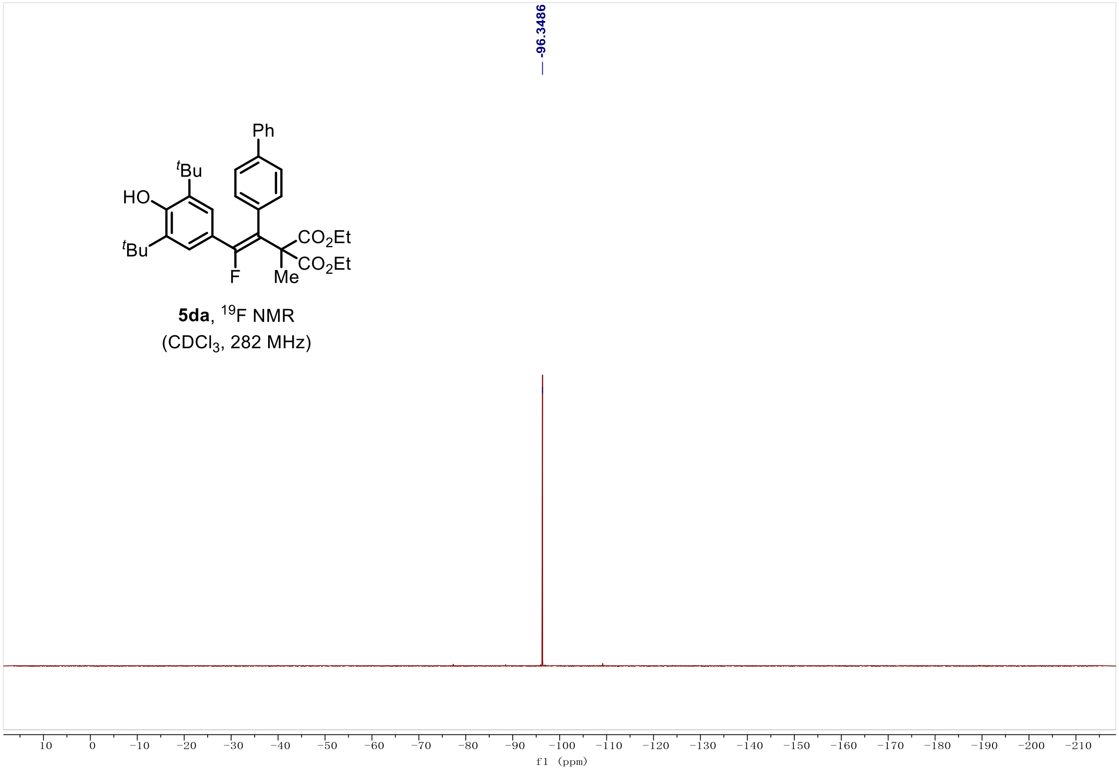
-96.3486
Ph
HO
tBu
tBu
F
Me
CO2Et
CO2Et
5da, 19F NMR
(CDCl3, 282 MHz)
f1 (ppm)

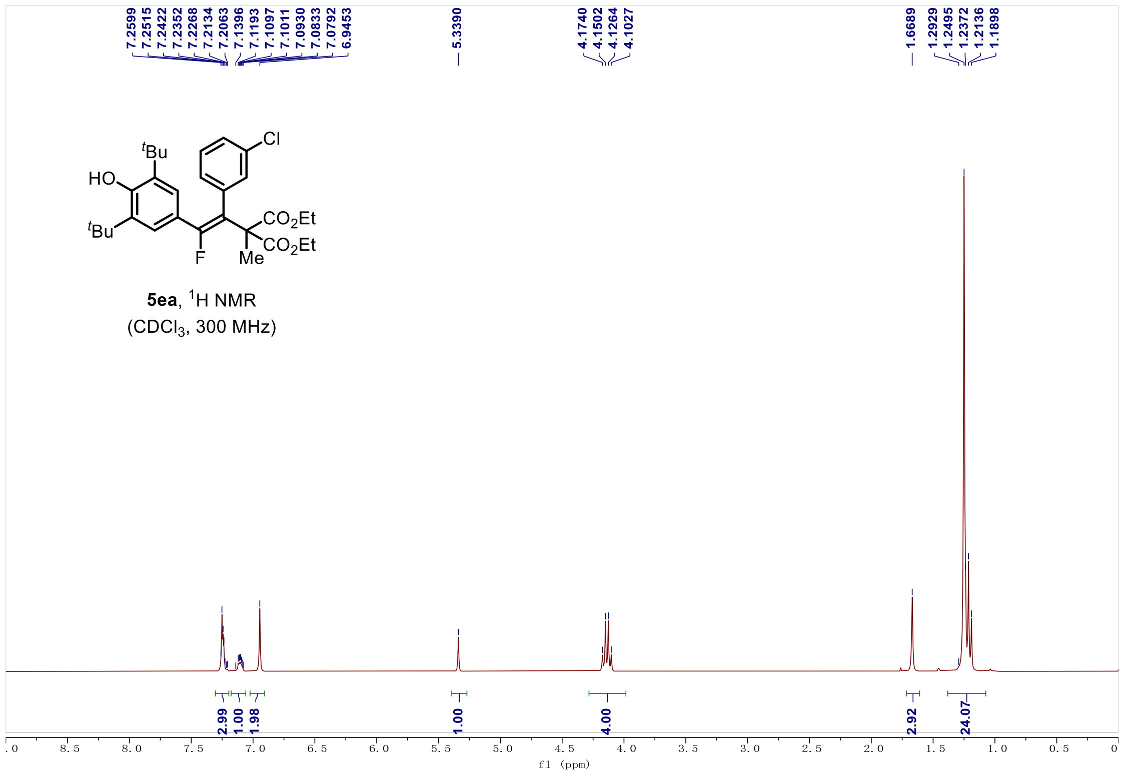
7.2599
7.2515
7.2422
7.2352
7.2268
7.2134
7.2063
7.1396
7.1193
7.1097
7.1011
7.0930
7.0833
7.0792
6.9453
5.3390
4.1740
4.1502
4.1264
4.1027
1.6689
1.2929
1.2495
1.2372
1.2136
1.1898
HO
tBu
tBu
Cl
CO2Et
CO2Et
F
Me
5ea, 1H NMR
(CDCl3, 300 MHz)
2.99
1.00
1.98
1.00
4.00
2.92
24.07
f1 (ppm)

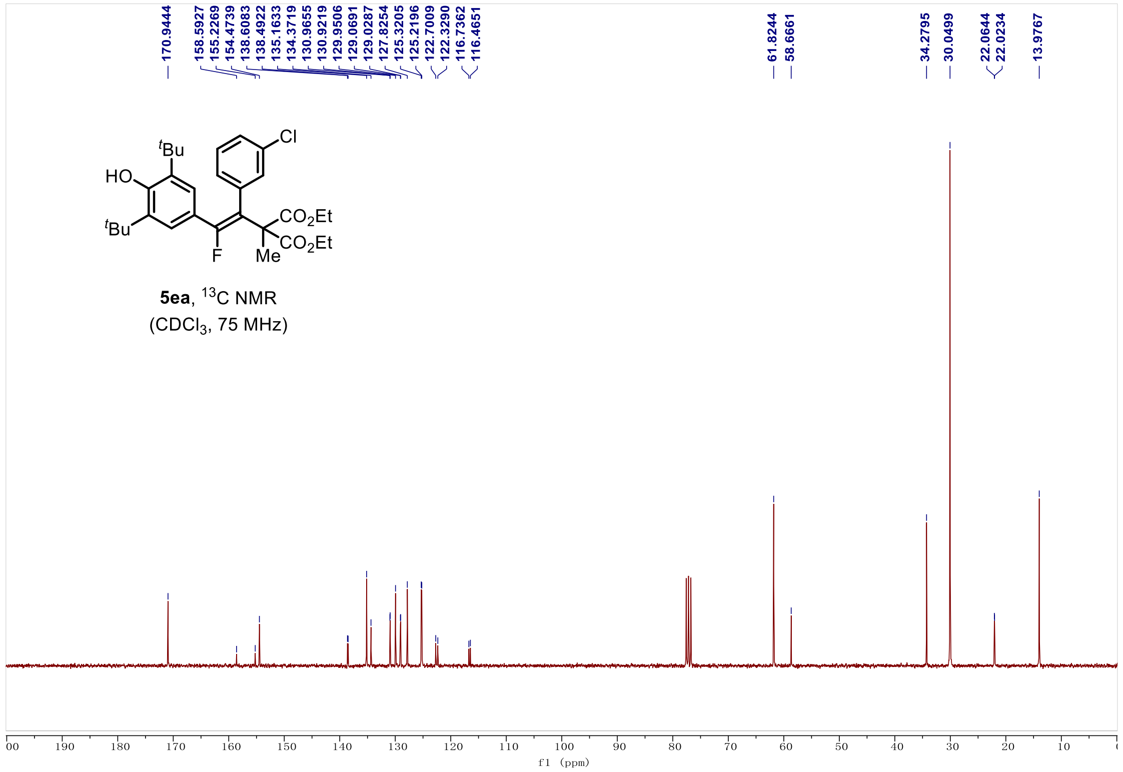
170.9444
158.5927
155.2269
154.4739
138.6083
138.4922
135.1633
134.3719
130.9655
130.9219
129.9506
129.0691
129.0287
127.8254
125.3205
125.2196
122.7009
122.3290
116.7362
116.4651
61.8244
58.6661
34.2795
30.0499
22.0644
22.0234
13.9767
HO
tBu
tBu
Cl
CO2Et
CO2Et
F
Me
5ea, 13C NMR
(CDCl3, 75 MHz)
f1 (ppm)

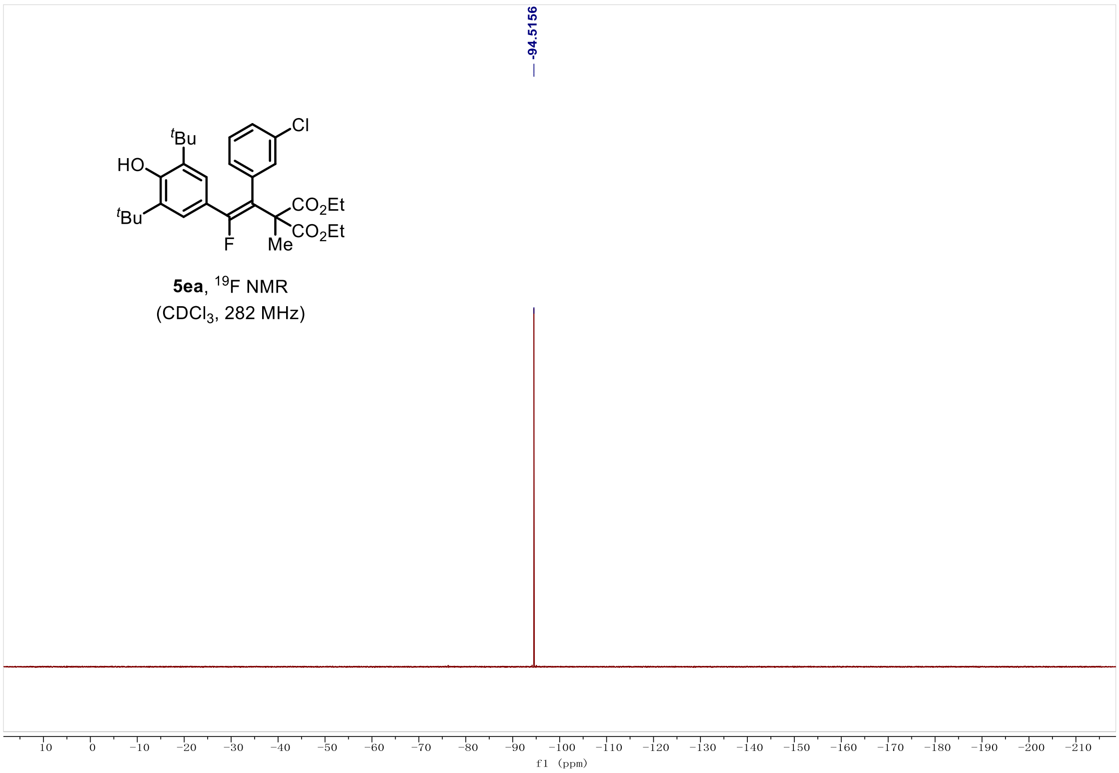

-94.5156
5ea, 19F NMR
(CDCl3, 282 MHz)
f1 (ppm)

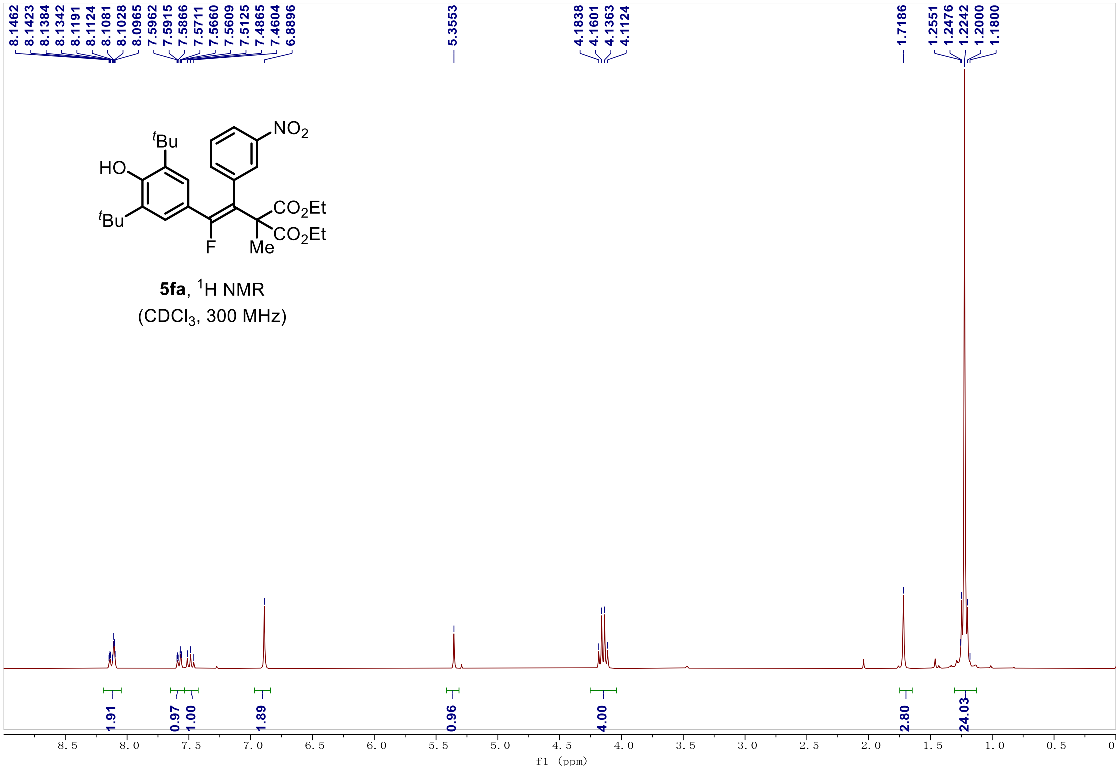

8.1462
8.1423
8.1384
8.1342
8.1191
8.1124
8.1081
8.1028
8.0965
7.5962
7.5915
7.5866
7.5711
7.5660
7.5609
7.5125
7.4865
7.4604
6.8896
5.3553
4.1838
4.1601
4.1363
4.1124
1.7186
1.2551
1.2476
1.2242
1.2000
1.1800
5fa, 1H NMR
(CDCl3, 300 MHz)
1.91
0.97
1.00
1.89
0.96
4.00
2.80
24.03
f1 (ppm)

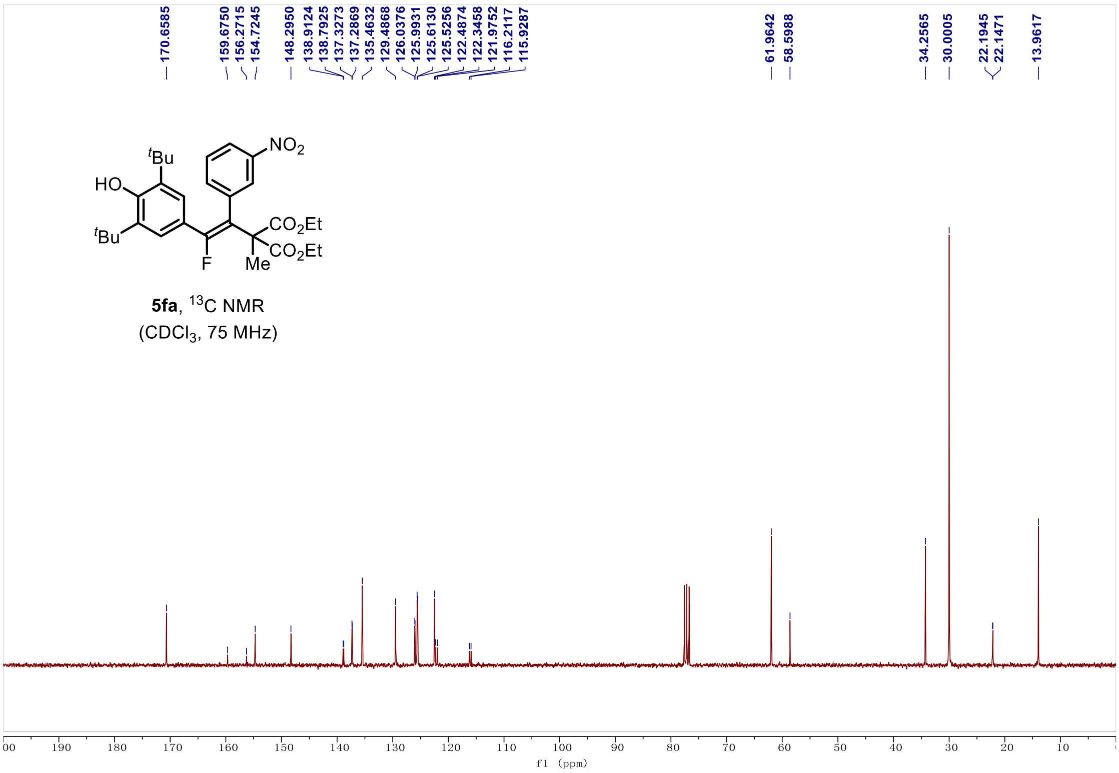
170.6585
159.6750
156.2715
154.7245
148.2950
138.9124
138.7925
137.3273
137.2869
135.4632
129.4868
126.0376
125.9931
125.6130
125.5256
122.4874
122.3458
121.9752
116.2117
115.9287
61.9642
58.5988
34.2565
30.0005
22.1945
22.1471
13.9617
NO2
tBu
HO
tBu
CO2Et
CO2Et
F
Me
5fa, 13C NMR
(CDCl3, 75 MHz)
f1 (ppm)

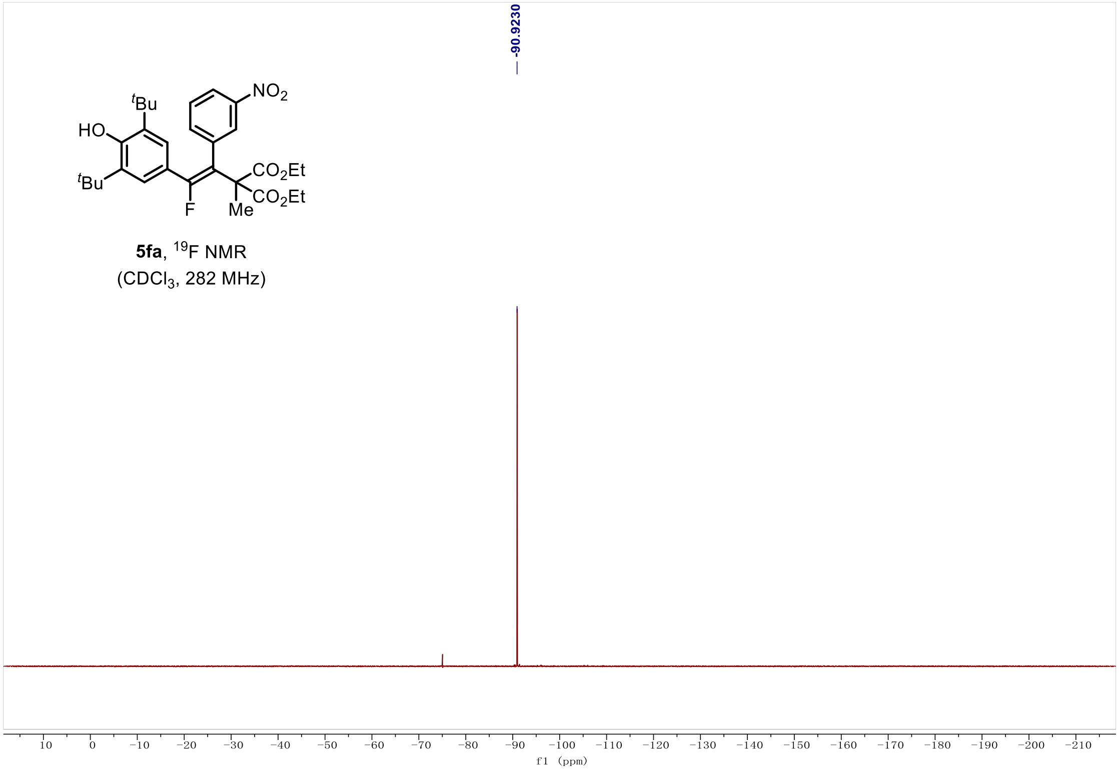
-90.9230
NO2
tBu
HO
tBu
CO2Et
CO2Et
F
Me
5fa, 19F NMR
(CDCl3, 282 MHz)
f1 (ppm)

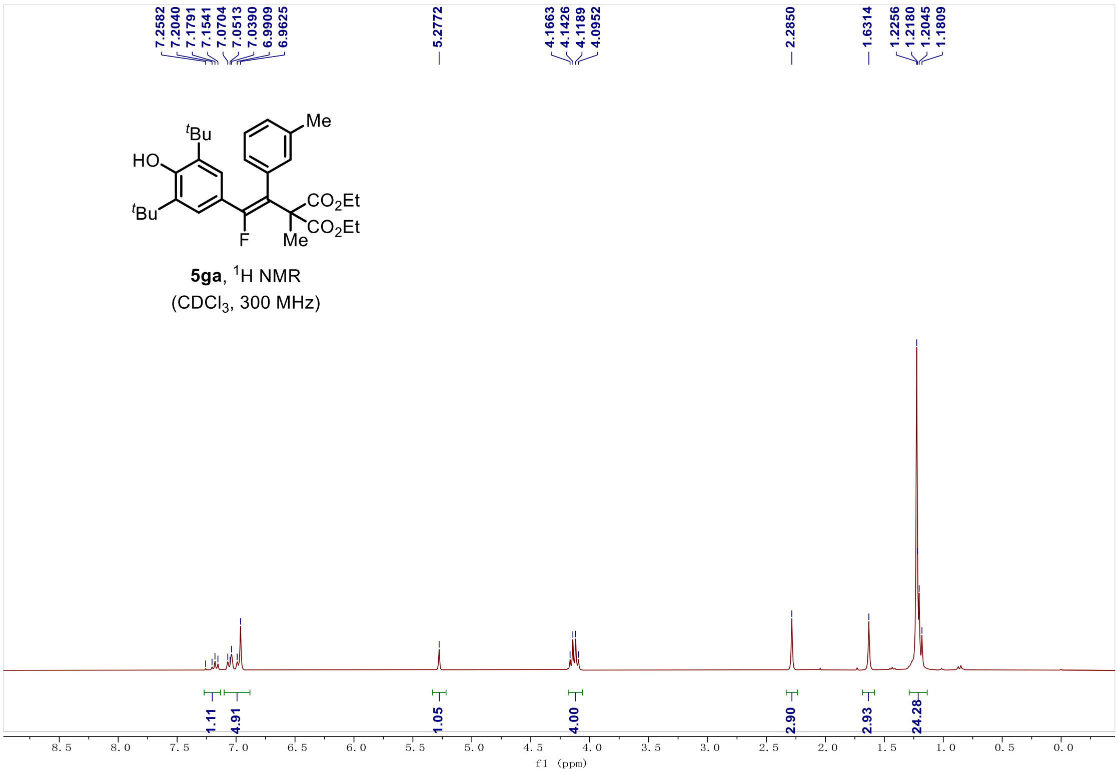
7.2582
7.2040
7.1791
7.1541
7.0704
7.0513
7.0390
6.9909
6.9625
5.2772
4.1663
4.1426
4.1189
4.0952
2.2850
1.6314
1.2256
1.2180
1.2045
1.1809
Me
tBu
HO
tBu
CO2Et
CO2Et
F
Me
5ga, 1H NMR
(CDCl3, 300 MHz)
1.11
4.91
1.05
4.00
2.90
2.93
24.28
f1 (ppm)

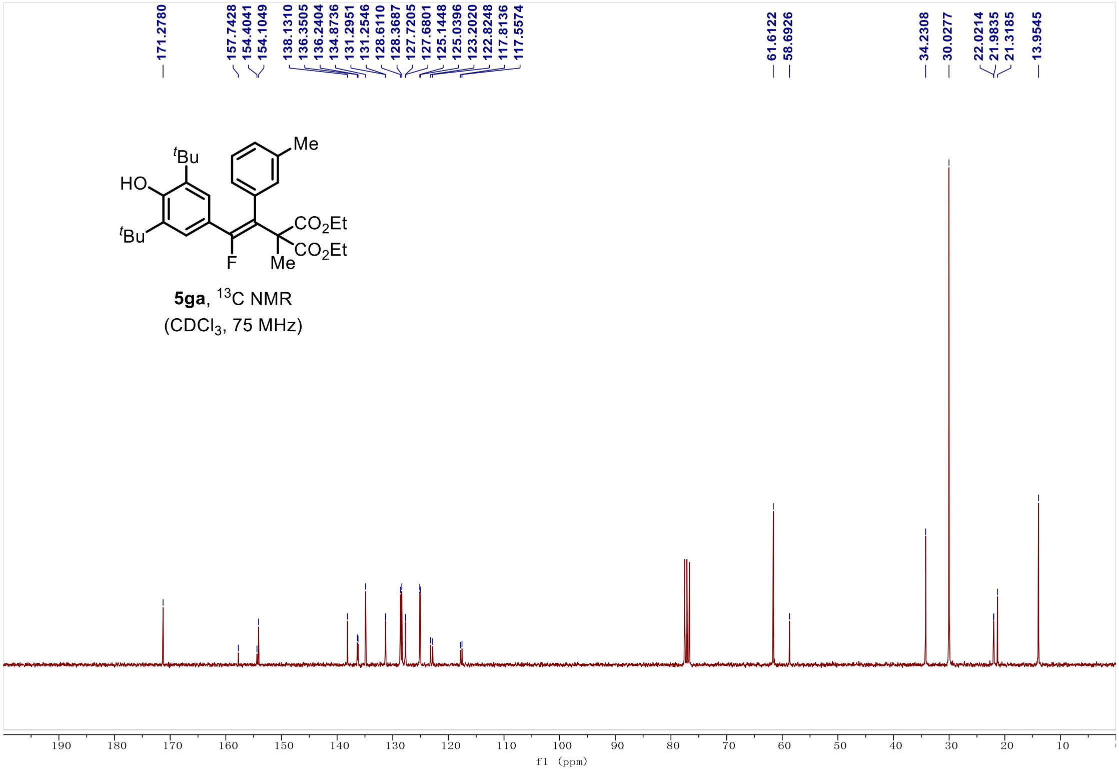
171.2780
157.7428
154.4041
154.1049
138.1310
136.3505
136.2404
134.8736
131.2951
131.2546
128.6110
128.3687
127.7205
127.6801
125.1448
125.0396
123.2020
122.8248
117.8136
117.5574
61.6122
58.6926
34.2308
30.0277
22.0214
21.9835
21.3185
13.9545
Me
tBu
HO
tBu
CO2Et
CO2Et
F
Me
5ga, 13C NMR
(CDCl3, 75 MHz)
f1 (ppm)

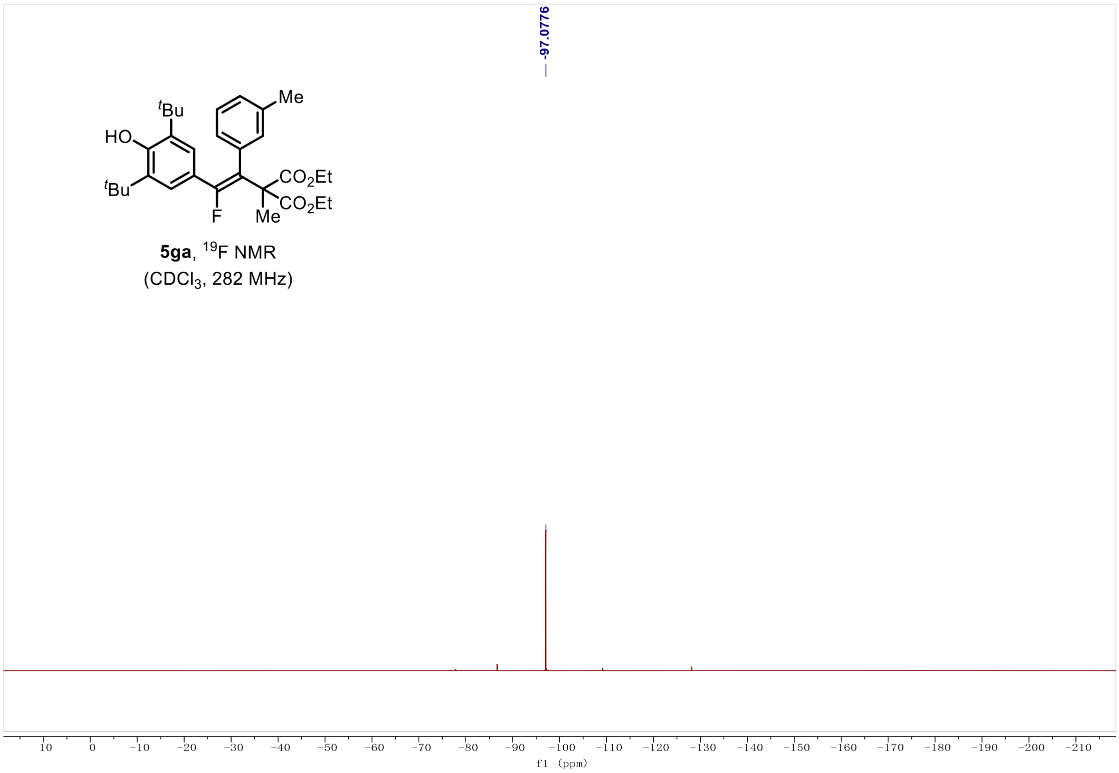

-97.0776
Me
^{t}Bu
HO
^{t}Bu
CO_2Et
CO_2Et
F
Me
5ga, ^{19}F NMR
($CDCl_3$, 282 MHz)
f1 (ppm)

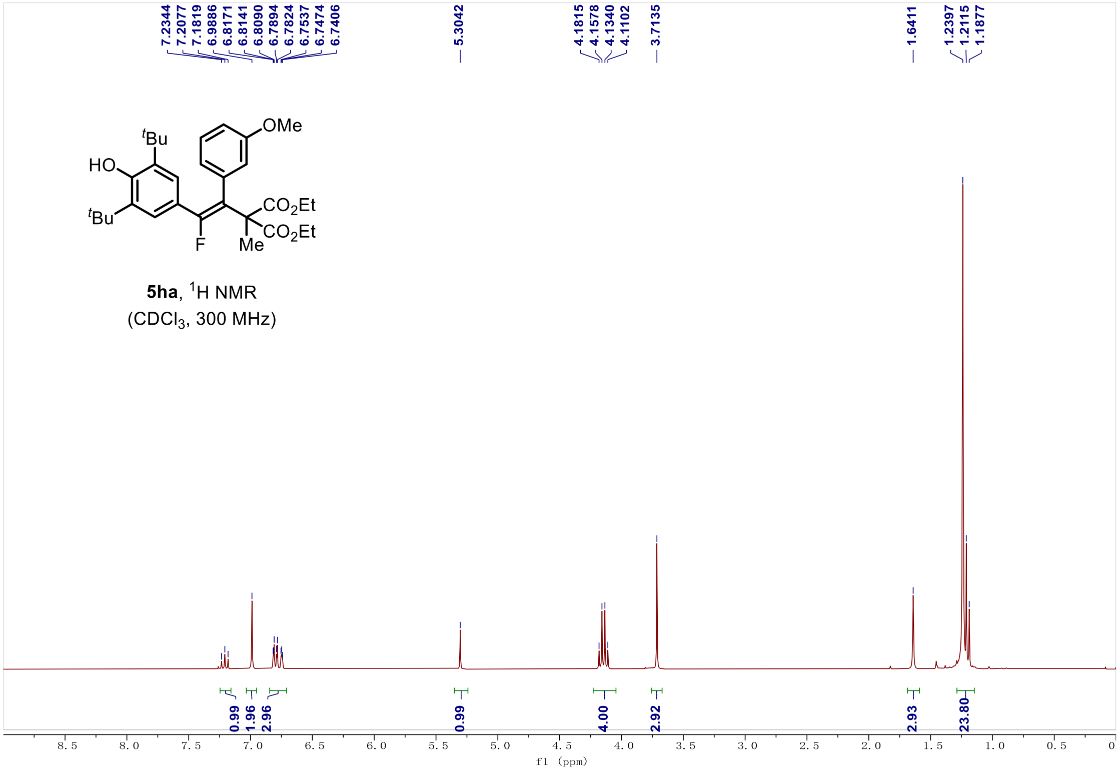

7.2344 7.2077 7.1819 6.9886 6.8171 6.8141 6.8090 6.7894 6.7824 6.7537 6.7474 6.7406
5.3042
4.1815 4.1578 4.1340 4.1102
3.7135
1.6411
1.2397 1.2115 1.1877
OMe
^{t}Bu
HO
^{t}Bu
CO_2Et
CO_2Et
F
Me
5ha, ^{1}H NMR
($CDCl_3$, 300 MHz)
0.99 1.96 2.96 0.99 4.00 2.92 2.93 23.80
f1 (ppm)

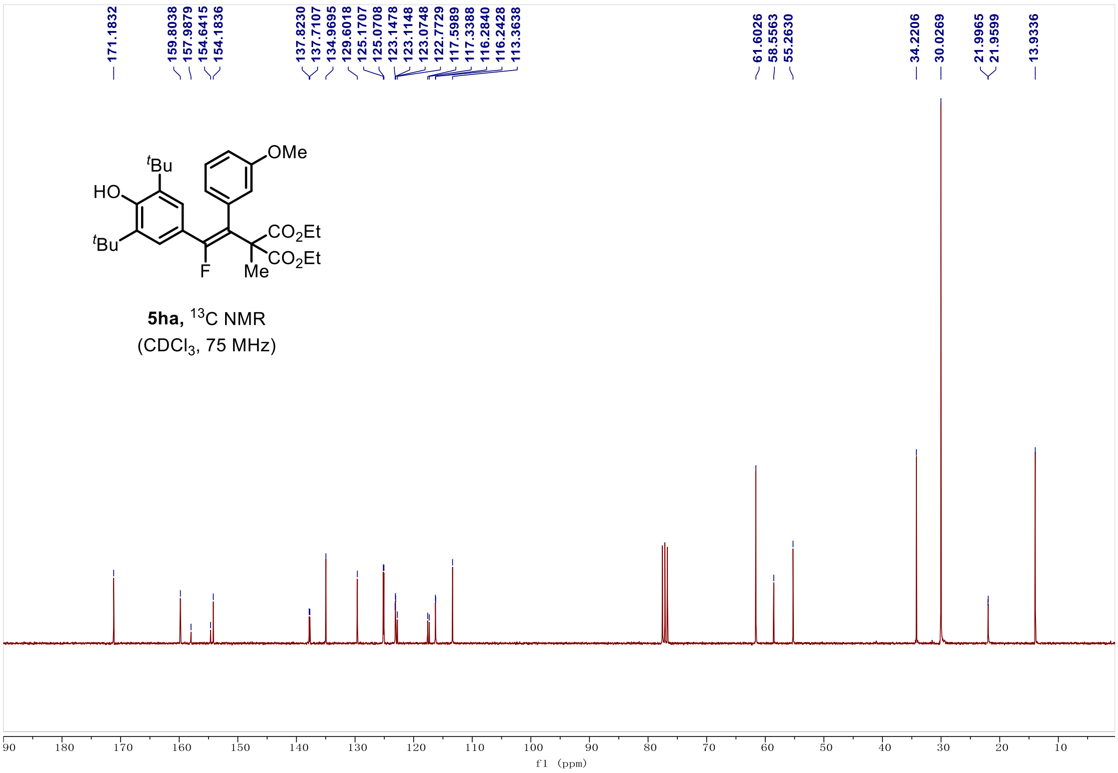

171.1832
159.8038
157.9879
154.6415
154.1836
137.8230
137.7107
134.9695
129.6018
125.1707
125.0708
123.1478
123.1148
123.0748
122.7729
117.5989
117.3388
116.2840
116.2428
113.3638
61.6026
58.5563
55.2630
34.2206
30.0269
21.9965
21.9599
13.9336
OMe
tBu
HO
tBu
CO2Et
CO2Et
F
Me
5ha, 13C NMR
(CDCl3, 75 MHz)
f1 (ppm)

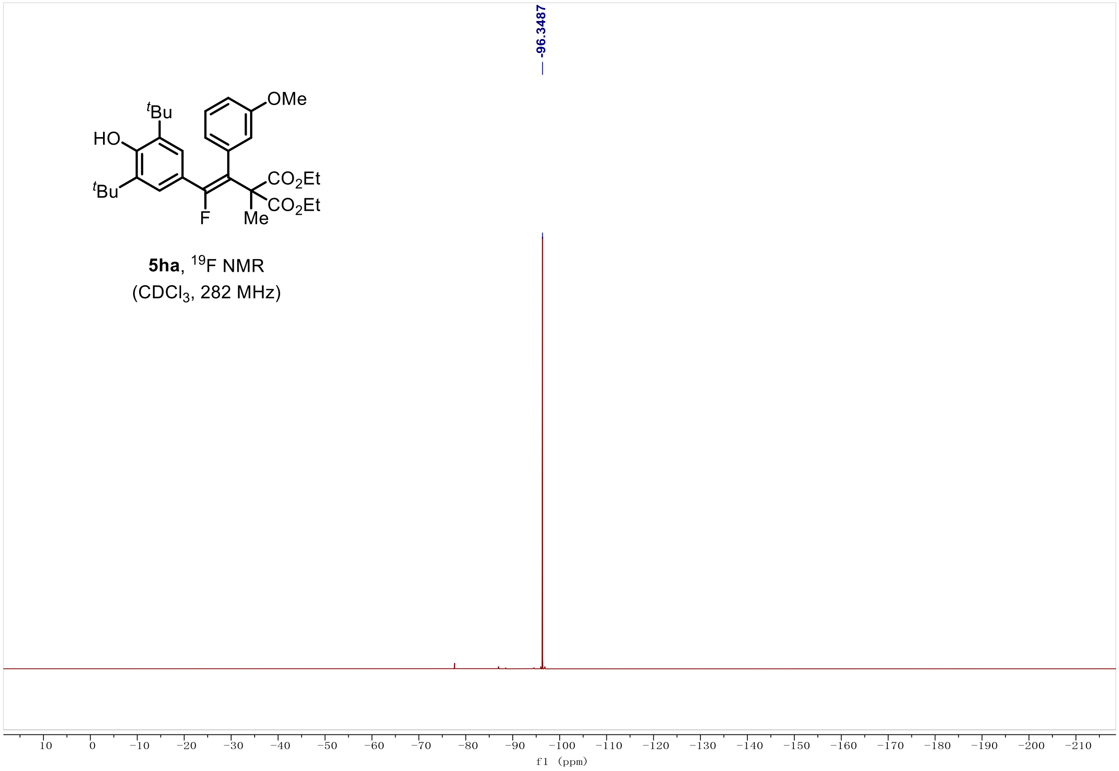

-96.3487
OMe
tBu
HO
tBu
CO2Et
CO2Et
F
Me
5ha, 19F NMR
(CDCl3, 282 MHz)
f1 (ppm)

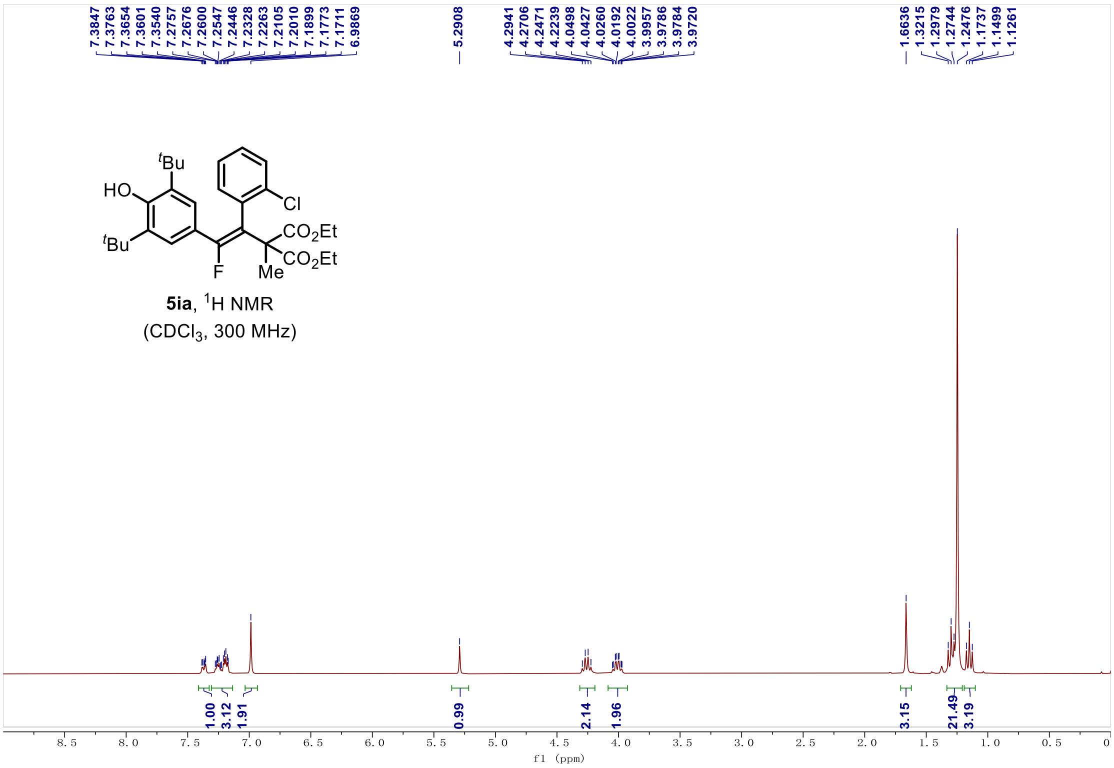
7.3847
7.3763
7.3654
7.3601
7.3540
7.2757
7.2676
7.2600
7.2547
7.2446
7.2328
7.2263
7.2105
7.2010
7.1899
7.1773
7.1711
6.9869
5.2908
4.2941
4.2706
4.2471
4.2239
4.0498
4.0427
4.0260
4.0192
4.0022
3.9957
3.9786
3.9784
3.9720
1.6636
1.3215
1.2979
1.2744
1.2476
1.1737
1.1499
1.1261
HO
tBu
Cl
CO2Et
CO2Et
F
Me
5ia, 1H NMR
(CDCl3, 300 MHz)
1.00
3.12
1.91
0.99
2.14
1.96
3.15
21.49
3.19
f1 (ppm)

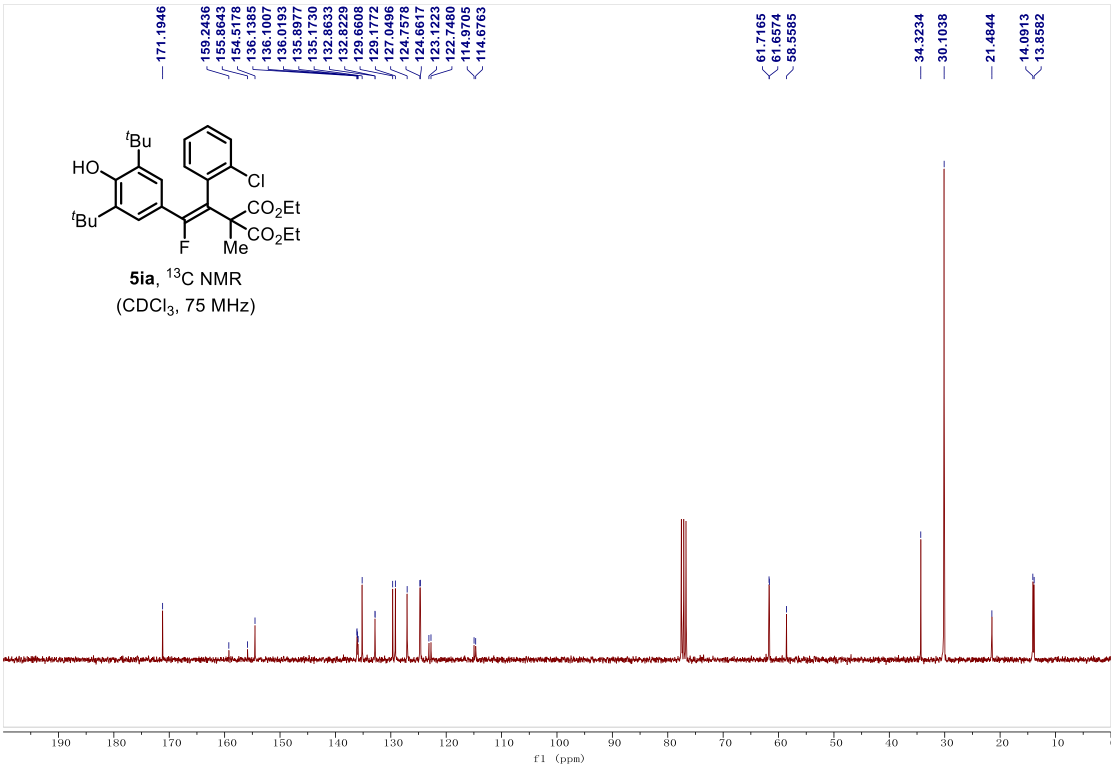
171.1946
159.2436
155.8643
154.5178
136.1385
136.1007
136.0193
135.8977
135.1730
132.8633
132.8229
129.6608
129.1772
127.0496
124.7578
124.6617
123.1223
122.7480
114.9705
114.6763
61.7165
61.6574
58.5585
34.3234
30.1038
21.4844
14.0913
13.8582
HO
tBu
Cl
CO2Et
CO2Et
F
Me
5ia, 13C NMR
(CDCl3, 75 MHz)
f1 (ppm)

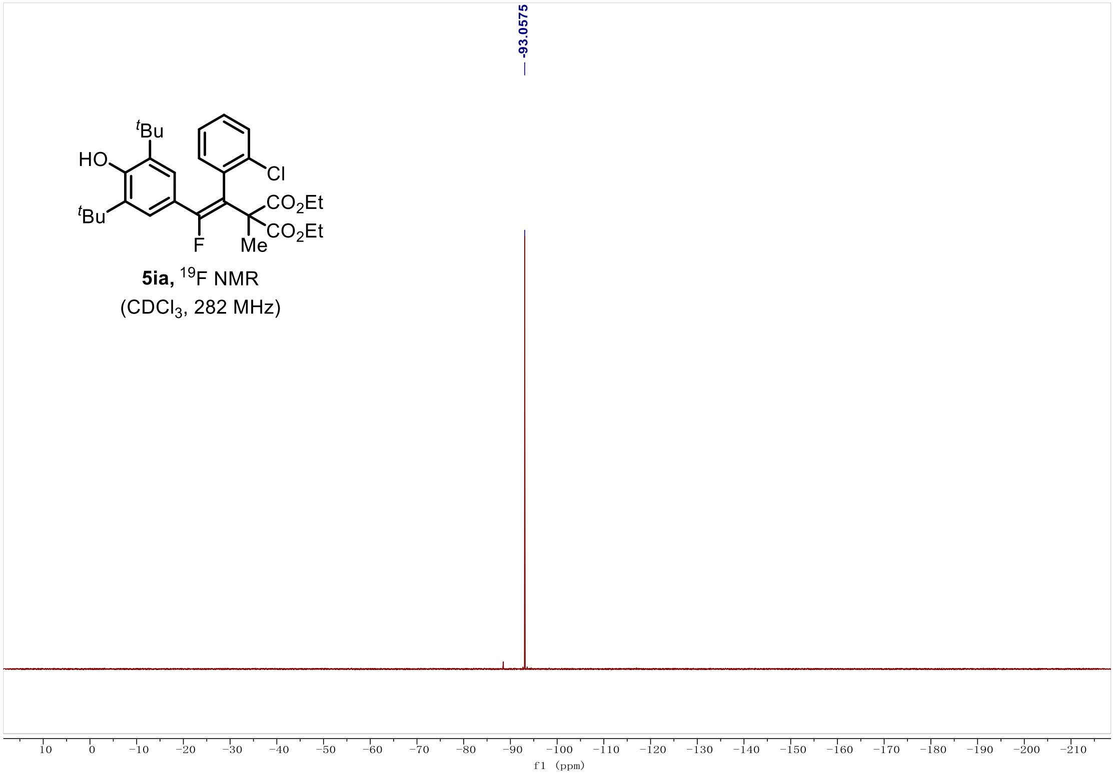
-93.0575
5ia, 19F NMR
(CDCl3, 282 MHz)
f1 (ppm)

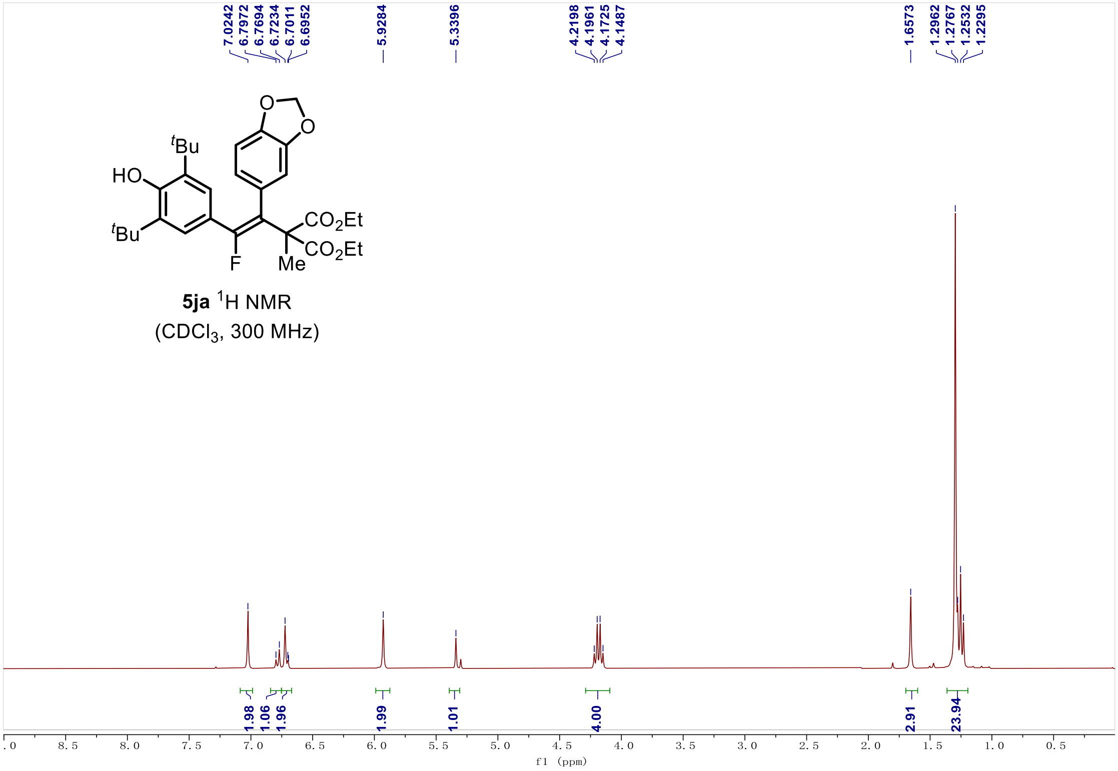
7.0242
6.7972
6.7694
6.7234
6.7011
6.6952
5.9284
5.3396
4.2198
4.1961
4.1725
4.1487
1.6573
1.2962
1.2767
1.2532
1.2295
5ja 1H NMR
(CDCl3, 300 MHz)
1.98
1.06
1.96
1.99
1.01
4.00
2.91
23.94
f1 (ppm)

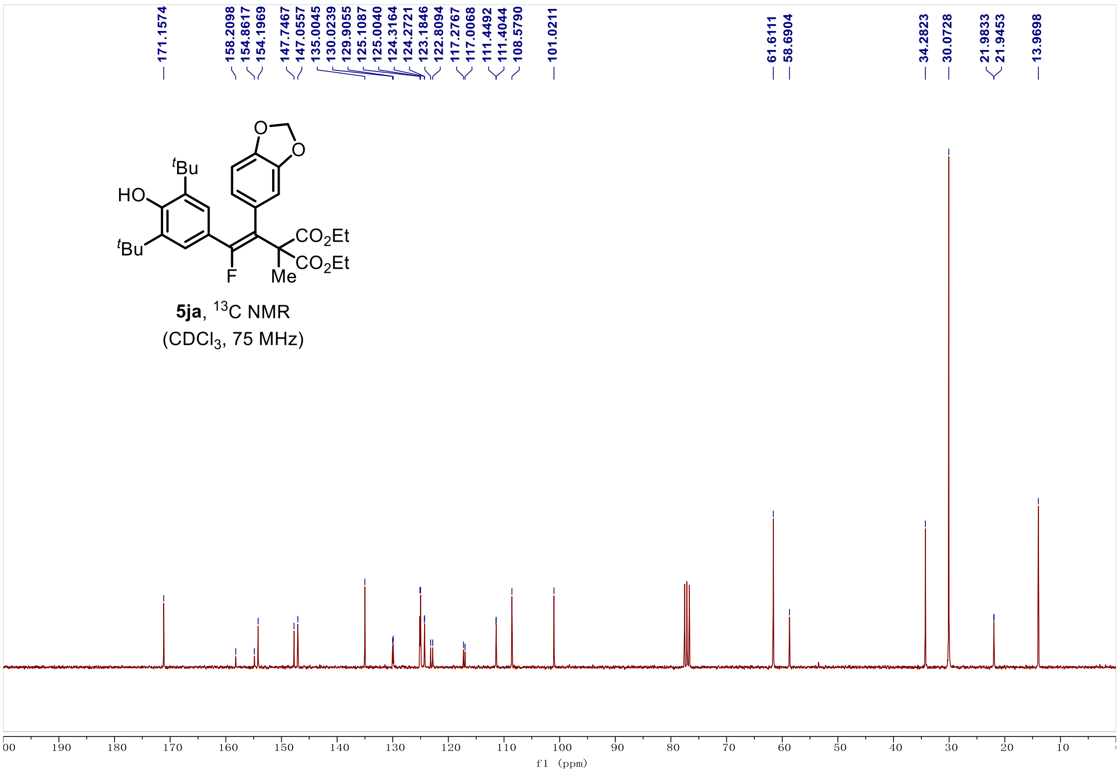
171.1574
158.2098
154.8617
154.1969
147.7467
147.0557
135.0045
130.0239
129.9055
125.1087
125.0040
124.3164
124.2721
123.1846
122.8094
117.2767
117.0068
111.4492
111.4044
108.5790
101.0211
61.6111
58.6904
34.2823
30.0728
21.9833
21.9453
13.9698
tBu
HO
tBu
O
O
CO2Et
CO2Et
F
Me
5ja, 13C NMR
(CDCl3, 75 MHz)
f1 (ppm)

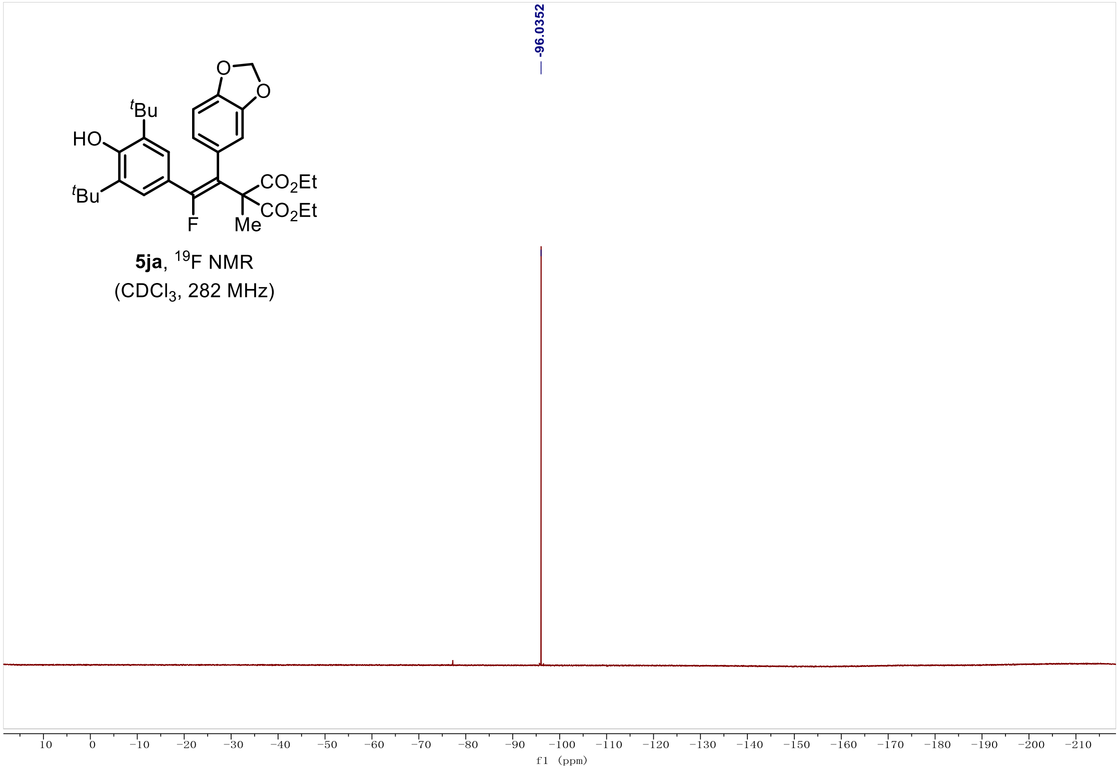
-96.0352
tBu
HO
tBu
O
O
CO2Et
CO2Et
F
Me
5ja, 19F NMR
(CDCl3, 282 MHz)
f1 (ppm)

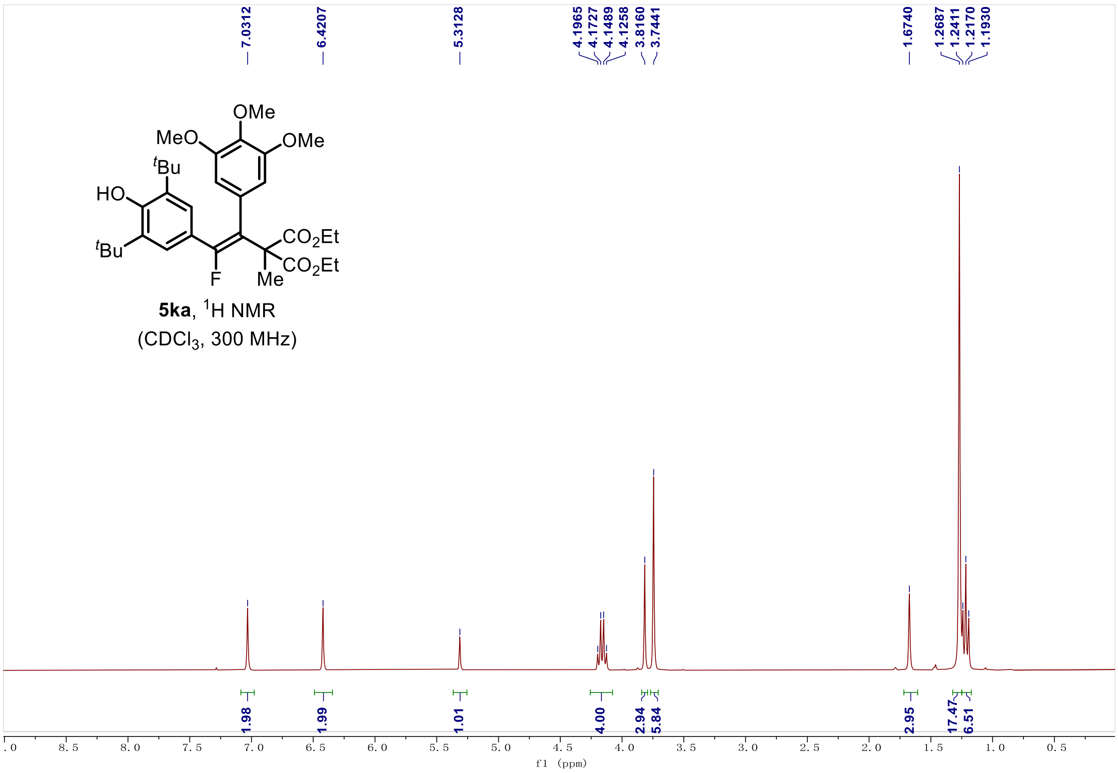
7.0312
6.4207
5.3128
4.1965
4.1727
4.1489
4.1258
3.8160
3.7441
1.6740
1.2687
1.2411
1.2170
1.1930
OMe
MeO
OMe
tBu
HO
tBu
CO2Et
CO2Et
F
Me
5ka, 1H NMR
(CDCl3, 300 MHz)
1.98
1.99
1.01
4.00
2.94
5.84
2.95
17.47
6.51
f1 (ppm)

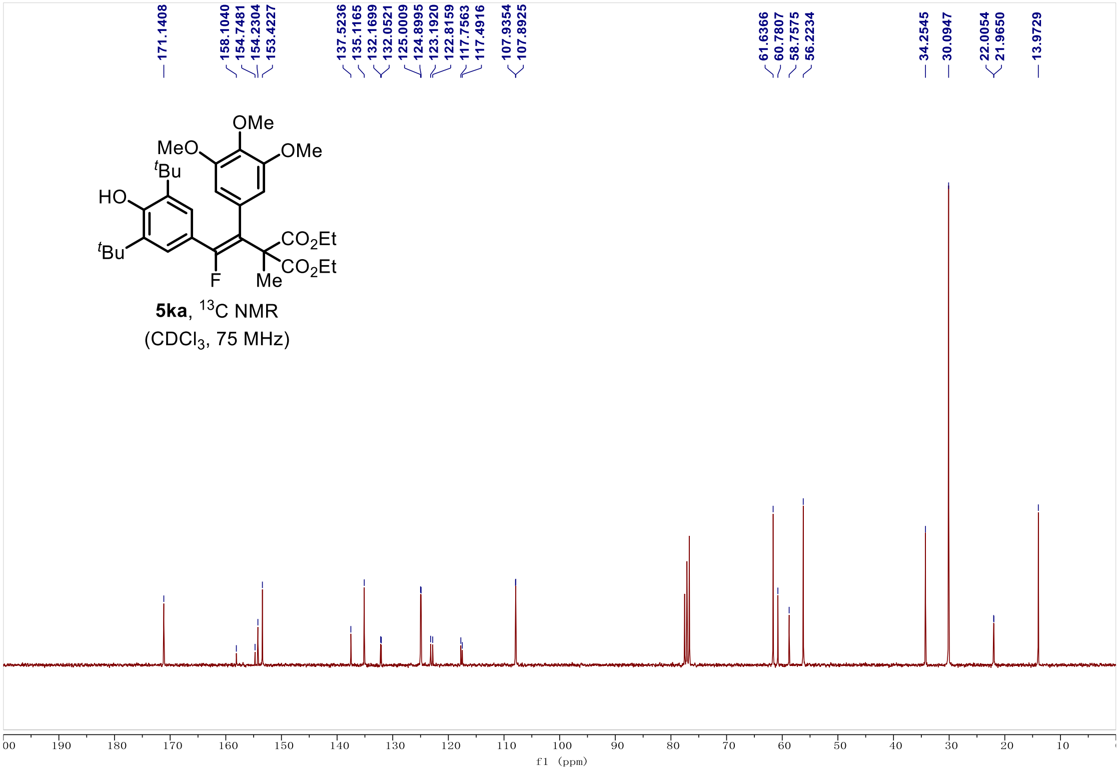
171.1408
158.1040
154.7481
154.2304
153.4227
137.5236
135.1165
132.1699
132.0521
125.0009
124.8995
123.1920
122.8159
117.7563
117.4916
107.9354
107.8925
61.6366
60.7807
58.7575
56.2234
34.2545
30.0947
22.0054
21.9650
13.9729
OMe
MeO
OMe
tBu
HO
tBu
CO2Et
CO2Et
F
Me
5ka, 13C NMR
(CDCl3, 75 MHz)
f1 (ppm)

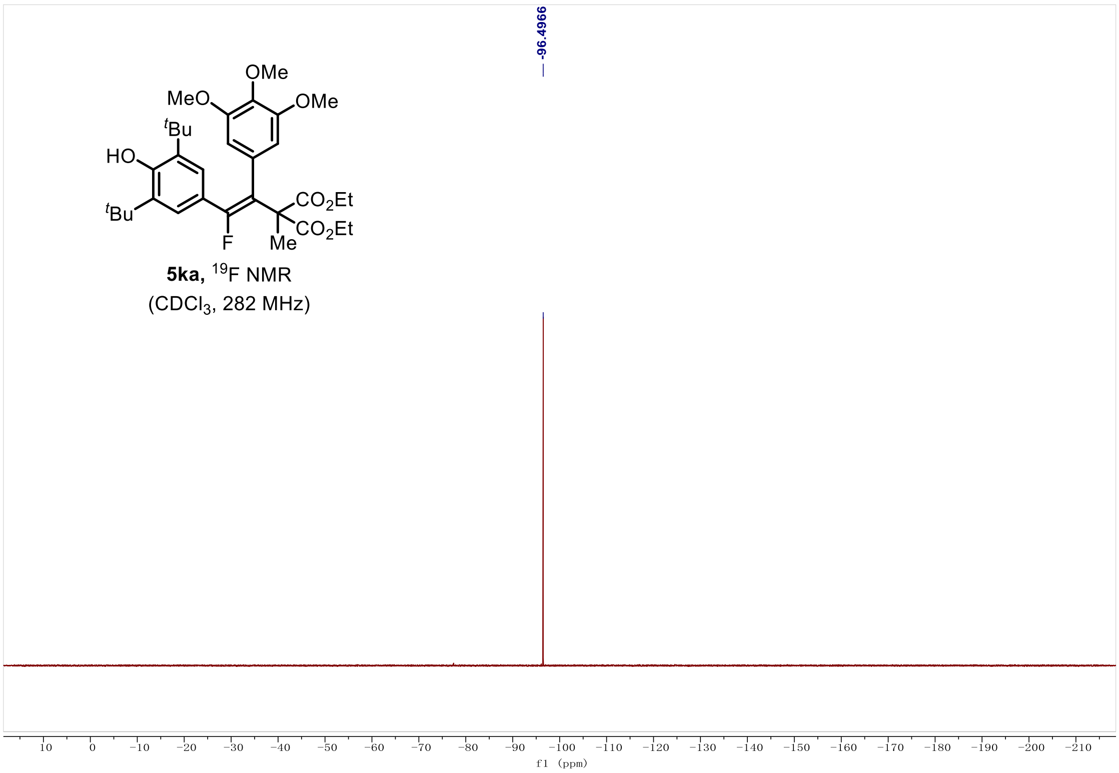

-96.4966
OMe
MeO
OMe
tBu
HO
tBu
CO2Et
CO2Et
F
Me
5ka, 19F NMR
(CDCl3, 282 MHz)
f1 (ppm)

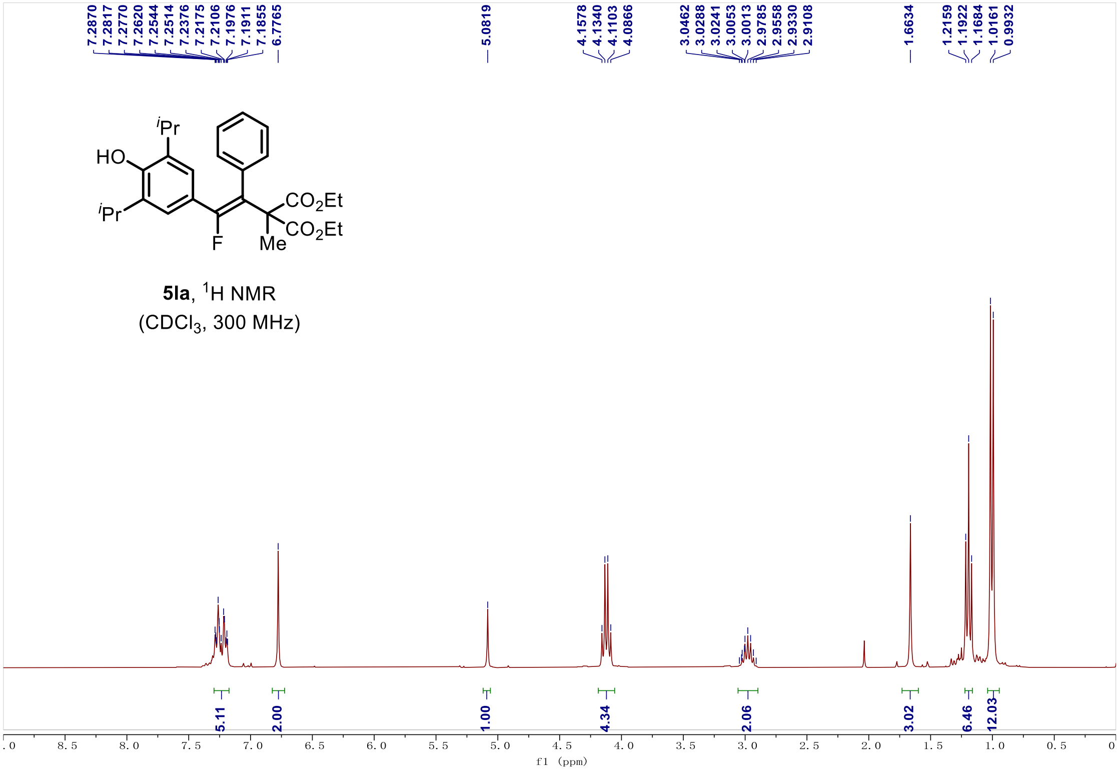

7.2870
7.2817
7.2770
7.2620
7.2544
7.2514
7.2376
7.2175
7.2106
7.1976
7.1911
7.1855
6.7765
5.0819
4.1578
4.1340
4.1103
4.0866
3.0462
3.0288
3.0241
3.0053
3.0013
2.9785
2.9558
2.9330
2.9108
1.6634
1.2159
1.1922
1.1684
1.0161
0.9932
iPr
HO
iPr
CO2Et
CO2Et
F
Me
5la, 1H NMR
(CDCl3, 300 MHz)
5.11
2.00
1.00
4.34
2.06
3.02
6.46
12.03
f1 (ppm)

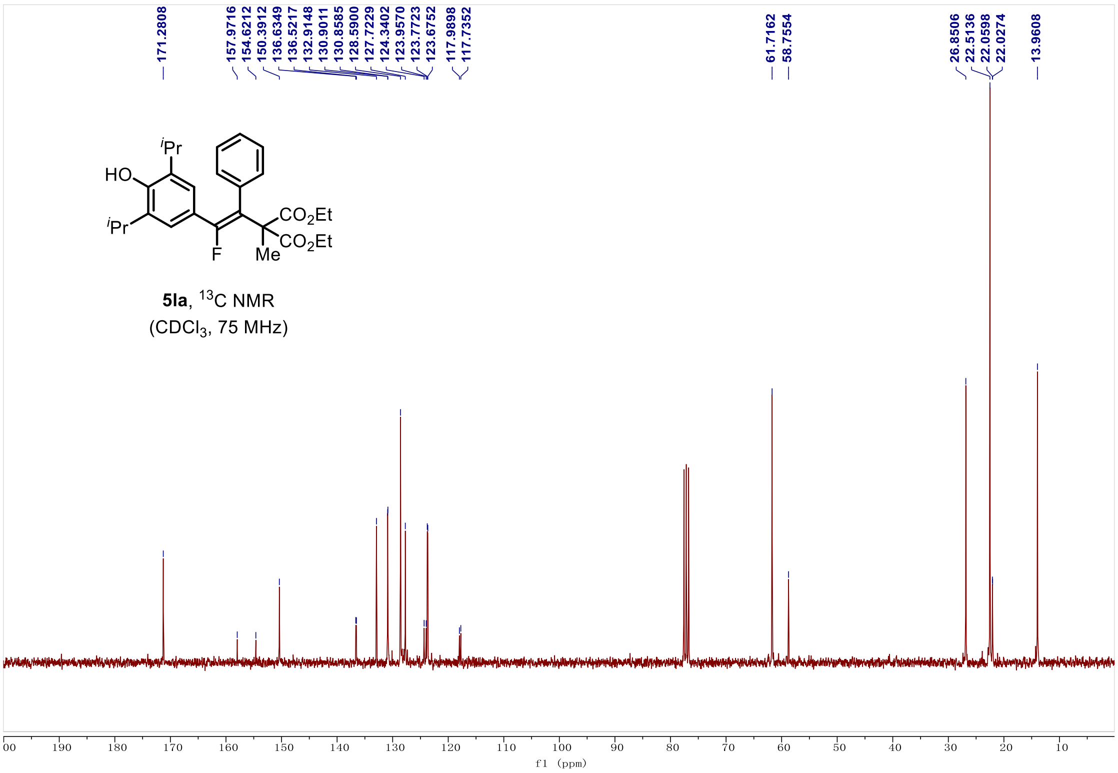
171.2808
157.9716
154.6212
150.3912
136.6349
136.5217
132.9148
130.9011
130.8585
128.5900
127.7229
124.3402
123.9570
123.7723
123.6752
117.9898
117.7352
61.7162
58.7554
26.8506
22.5136
22.0598
22.0274
13.9608
HO
iPr
iPr
CO2Et
CO2Et
F
Me
5la, 13C NMR
(CDCl3, 75 MHz)
f1 (ppm)

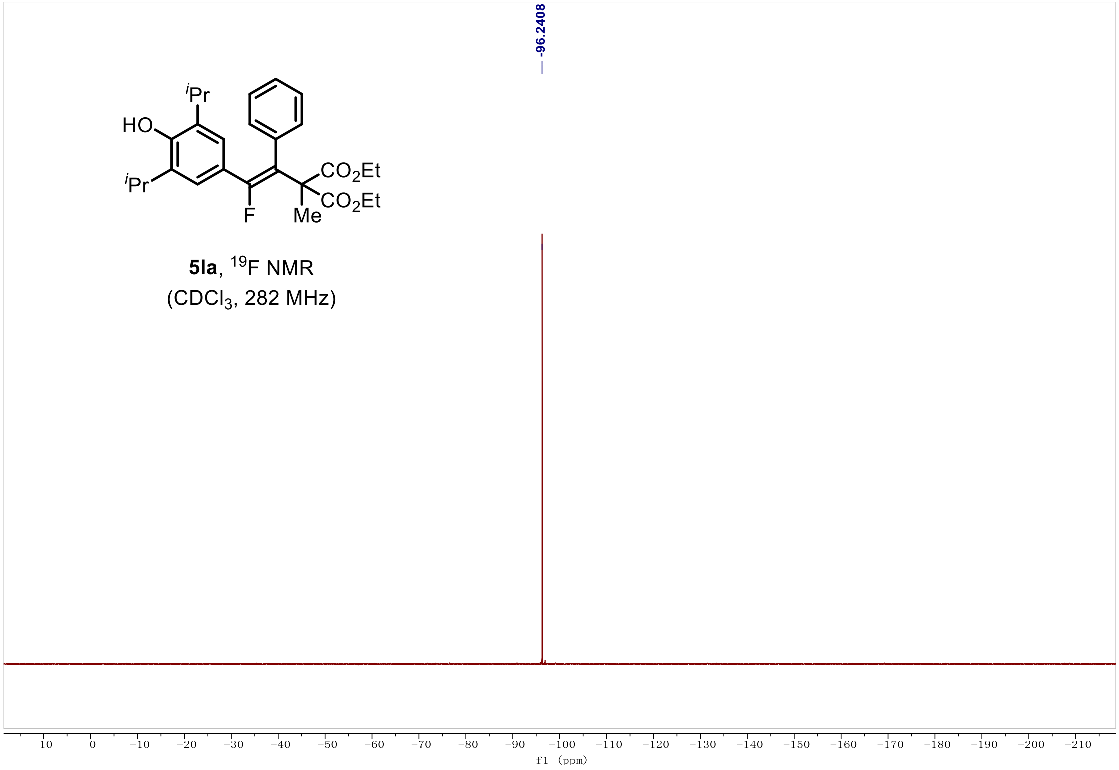
-96.2408
HO
iPr
iPr
CO2Et
CO2Et
F
Me
5la, 19F NMR
(CDCl3, 282 MHz)
f1 (ppm)

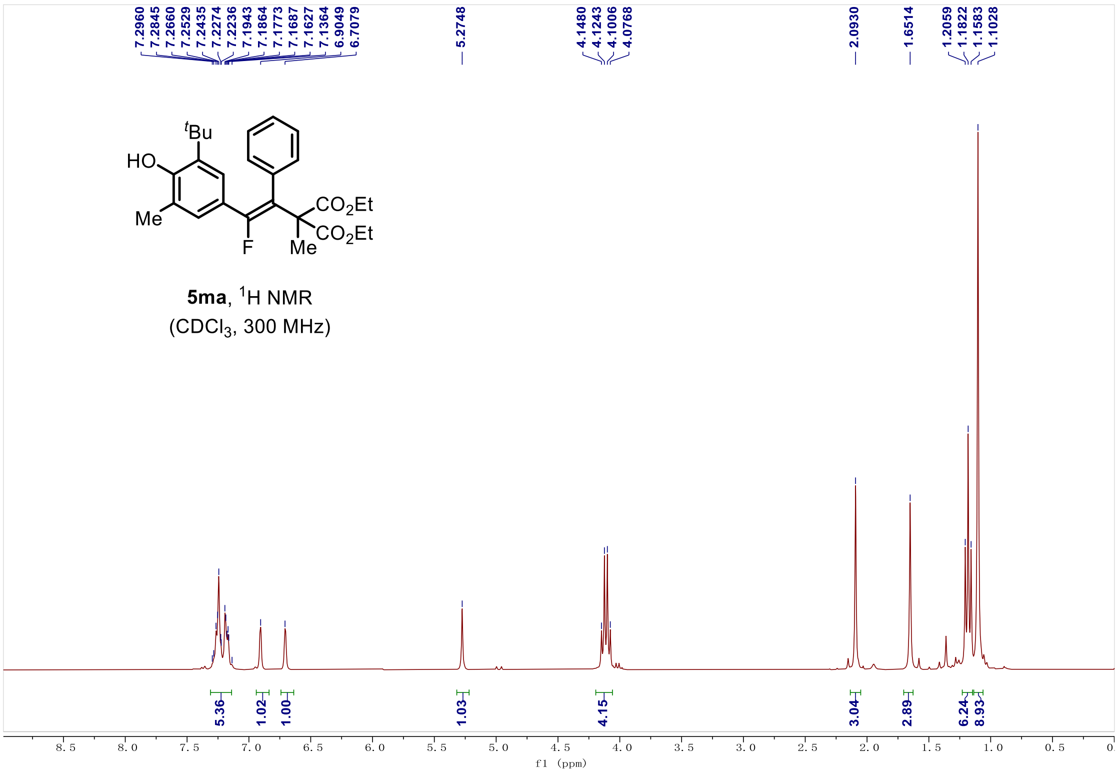
7.2960
7.2845
7.2660
7.2529
7.2435
7.2274
7.2236
7.1943
7.1864
7.1773
7.1687
7.1627
7.1364
6.9049
6.7079
5.2748
4.1480
4.1243
4.1006
4.0768
2.0930
1.6514
1.2059
1.1822
1.1583
1.1028
^{t}Bu
HO
Me
CO_2Et
CO_2Et
F
Me
5ma, ^{1}H NMR
($CDCl_3$, 300 MHz)
5.36
1.02
1.00
1.03
4.15
3.04
2.89
6.24
8.93
8.5 8.0 7.5 7.0 6.5 6.0 5.5 5.0 4.5 4.0 3.5 3.0 2.5 2.0 1.5 1.0 0.5 0.
f1 (ppm)

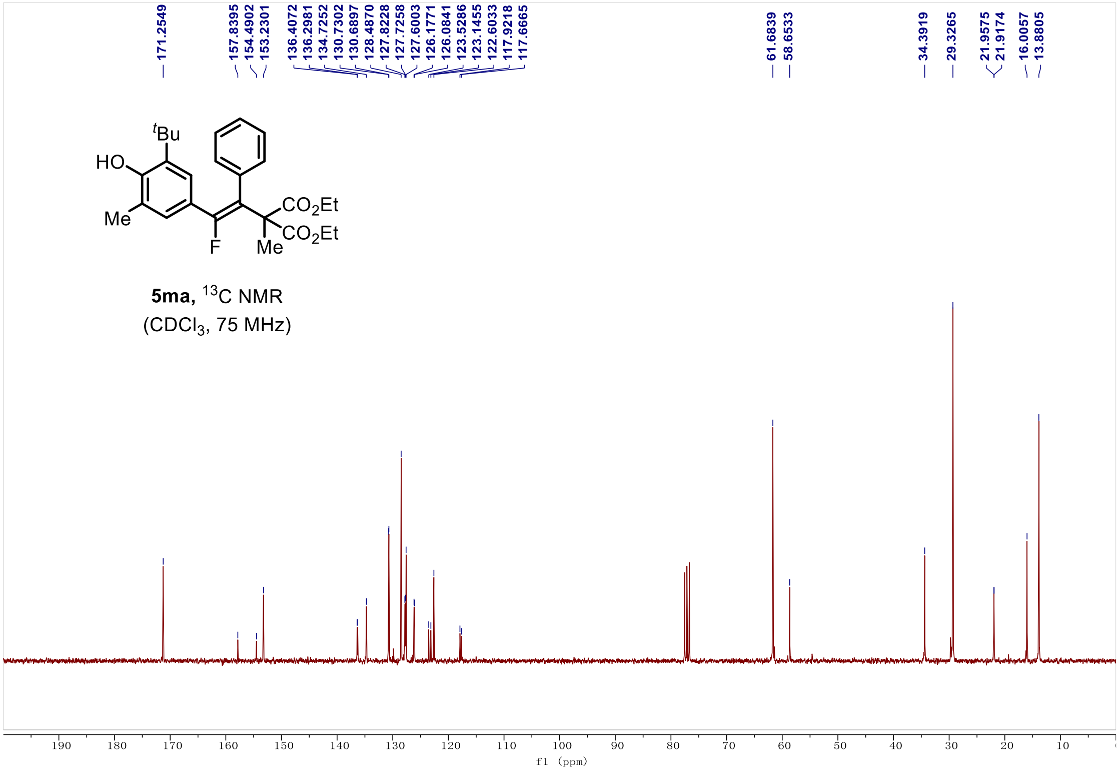
171.2549
157.8395
154.4902
153.2301
136.4072
136.2981
134.7252
130.7302
130.6897
128.4870
127.8228
127.7258
127.6003
126.1771
126.0841
123.5286
123.1455
122.6033
117.9218
117.6665
61.6839
58.6533
34.3919
29.3265
21.9575
21.9174
16.0057
13.8805
^{t}Bu
HO
Me
CO_2Et
CO_2Et
F
Me
5ma, ^{13}C NMR
($CDCl_3$, 75 MHz)
190 180 170 160 150 140 130 120 110 100 90 80 70 60 50 40 30 20 10
f1 (ppm)

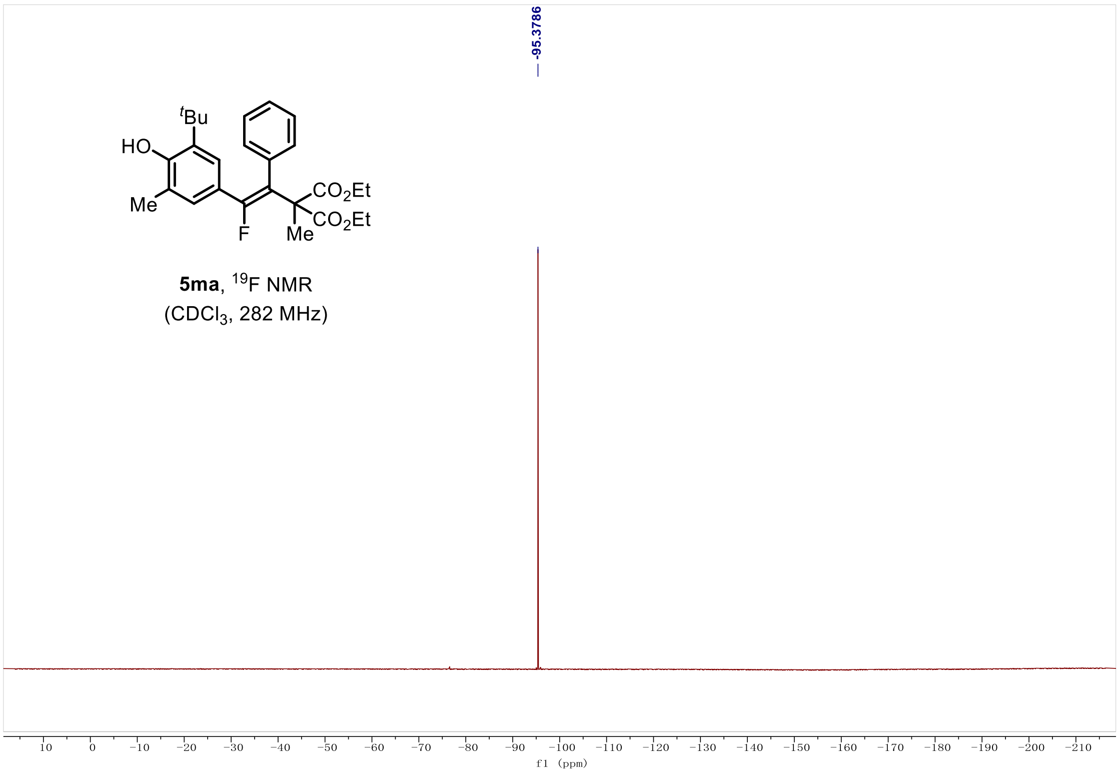

5ma, ^{19}F NMR
($CDCl_3$, 282 MHz)

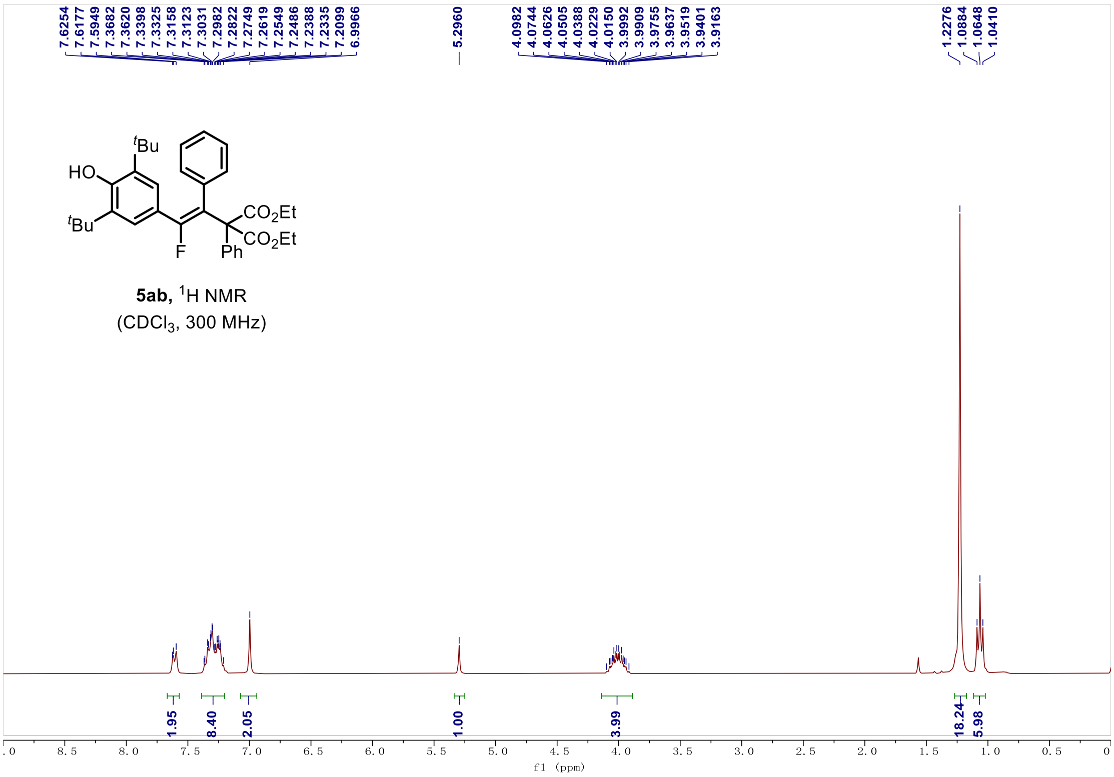

5ab, ^{1}H NMR
($CDCl_3$, 300 MHz)

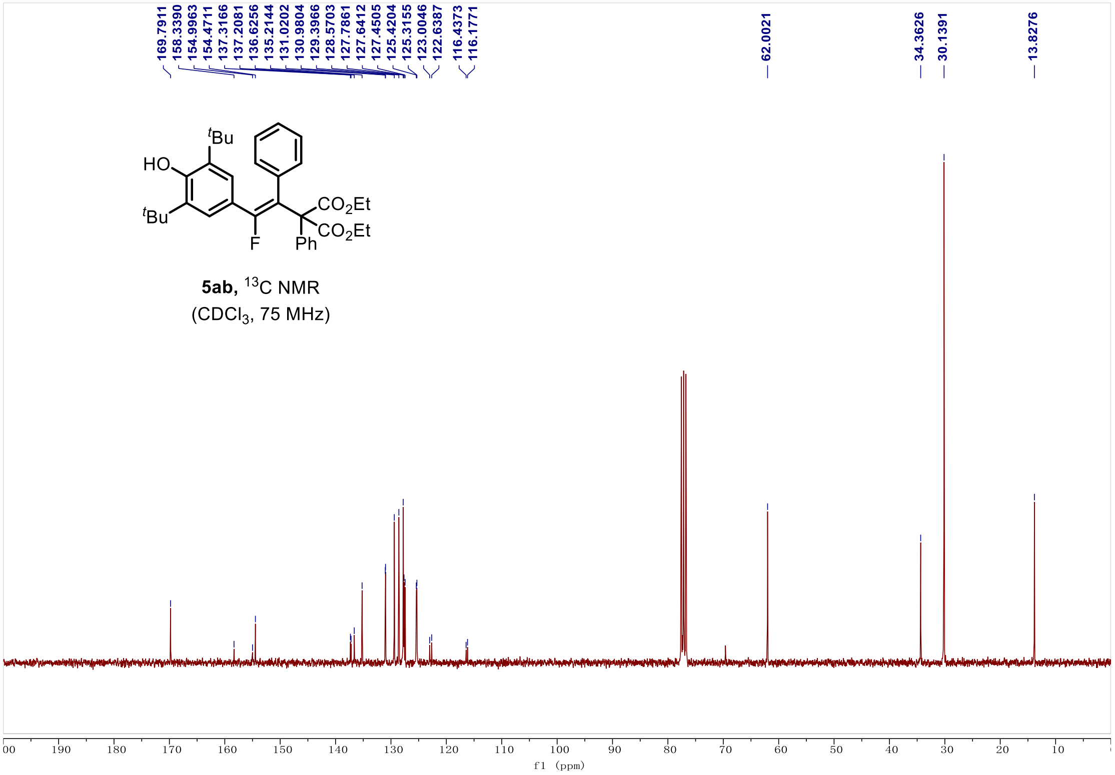
169.7911
158.3390
154.9963
154.4711
137.3166
137.2081
136.6256
135.2144
131.0202
130.9804
129.3966
128.5703
127.7861
127.6412
127.4505
125.4204
125.3155
123.0046
122.6387
116.4373
116.1771
62.0021
34.3626
30.1391
13.8276
HO
tBu
tBu
F
Ph
CO2Et
CO2Et
5ab, 13C NMR
(CDCl3, 75 MHz)
f1 (ppm)

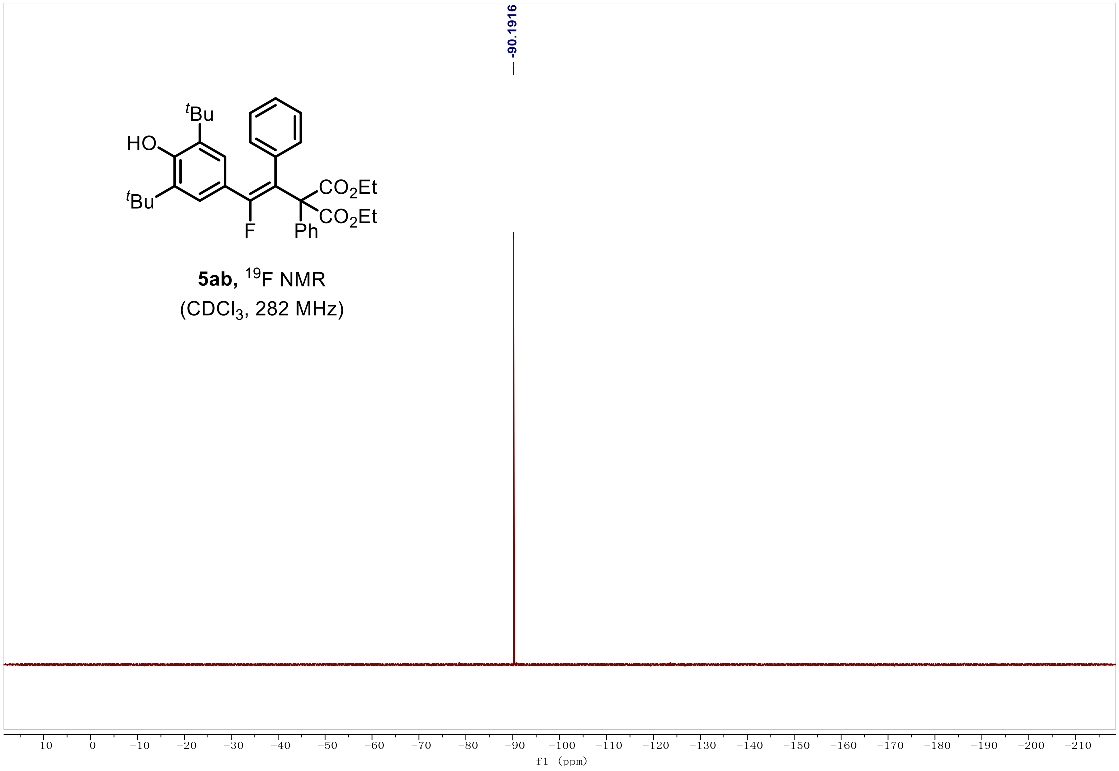
-90.1916
HO
tBu
tBu
F
Ph
CO2Et
CO2Et
5ab, 19F NMR
(CDCl3, 282 MHz)
f1 (ppm)

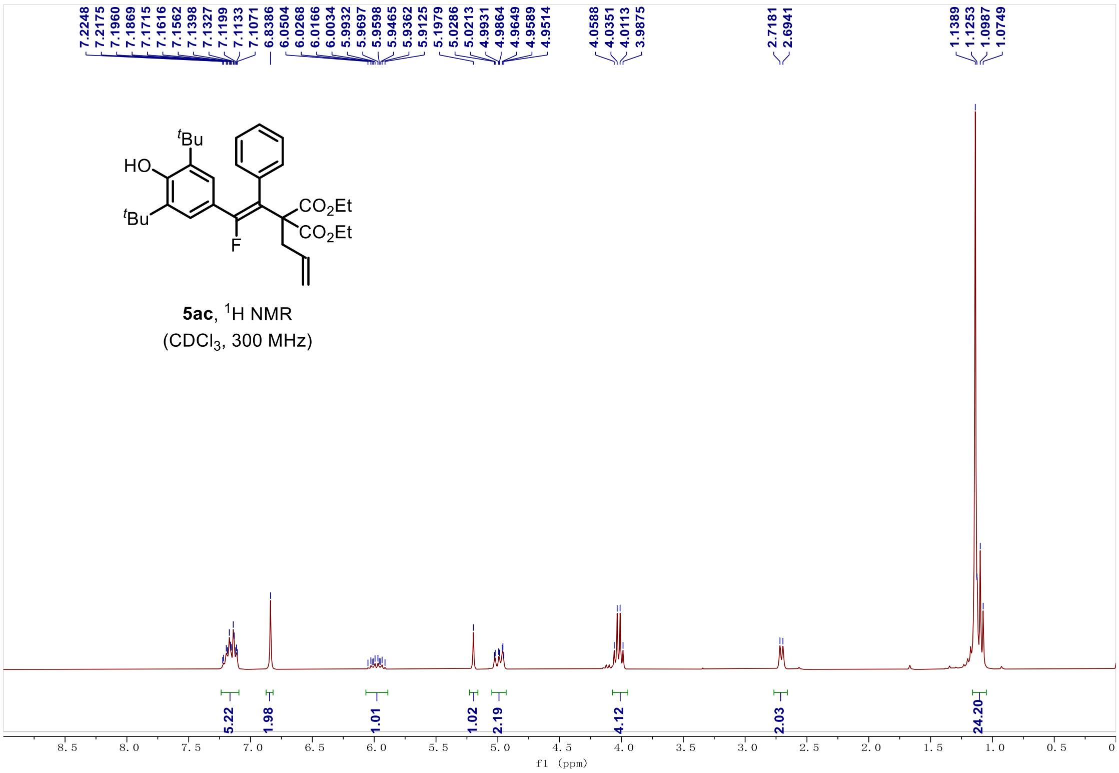
7.2248 7.2175 7.1960 7.1869 7.1715 7.1616 7.1562 7.1398 7.1327 7.1199 7.1133 7.1071
6.8386
6.0504 6.0268 6.0166 6.0034 5.9932 5.9697 5.9598 5.9465 5.9362 5.9125
5.1979 5.0286 5.0213 4.9931 4.9864 4.9649 4.9589 4.9514
4.0588 4.0351 4.0113 3.9875
2.7181 2.6941
1.1389 1.1253 1.0987 1.0749
HO
^{t}Bu
^{t}Bu
CO_2Et
CO_2Et
F
5ac, ^{1}H NMR
($CDCl_3$, 300 MHz)
5.22 1.98 1.01 1.02 2.19 4.12 2.03 24.20
8.5 8.0 7.5 7.0 6.5 6.0 5.5 5.0 4.5 4.0 3.5 3.0 2.5 2.0 1.5 1.0 0.5 0
f1 (ppm)

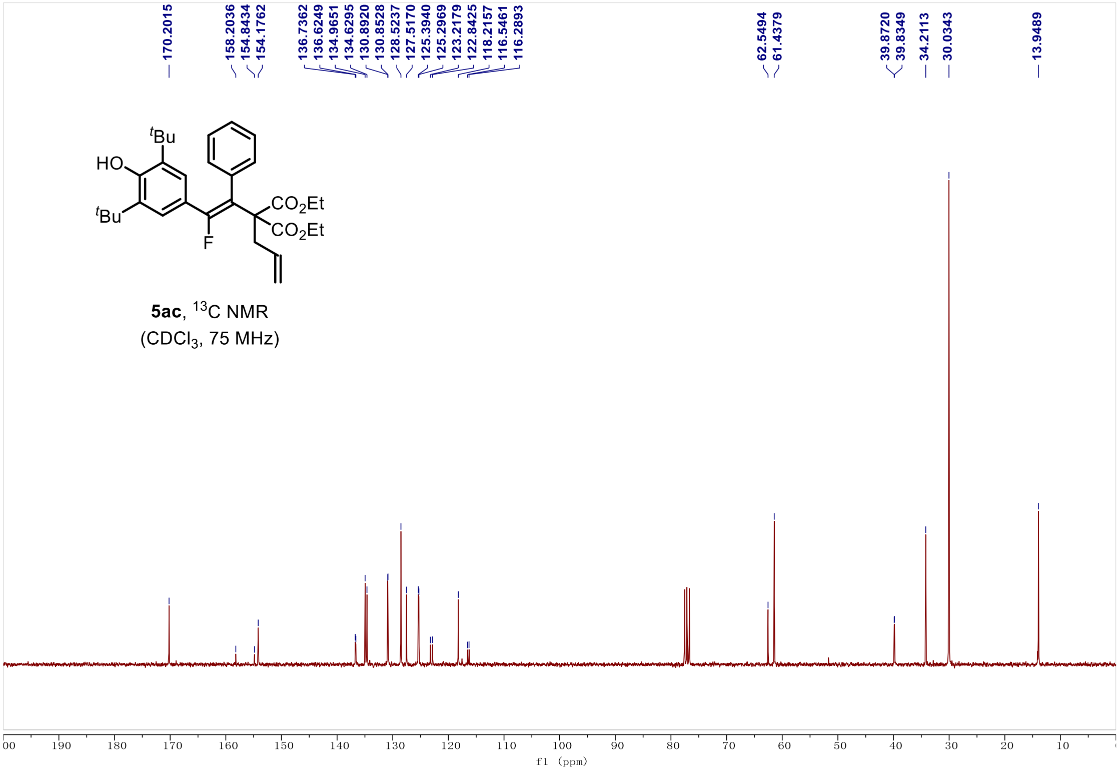
170.2015
158.2036 154.8434 154.1762
136.7362 136.6249 134.9651 134.6295 130.8920 130.8528 128.5237 127.5170 125.3940 125.2969 123.2179 122.8425 118.2157 116.5461 116.2893
62.5494 61.4379
39.8720 39.8349 34.2113 30.0343
13.9489
HO
^{t}Bu
^{t}Bu
CO_2Et
CO_2Et
F
5ac, ^{13}C NMR
($CDCl_3$, 75 MHz)
00 190 180 170 160 150 140 130 120 110 100 90 80 70 60 50 40 30 20 10
f1 (ppm)

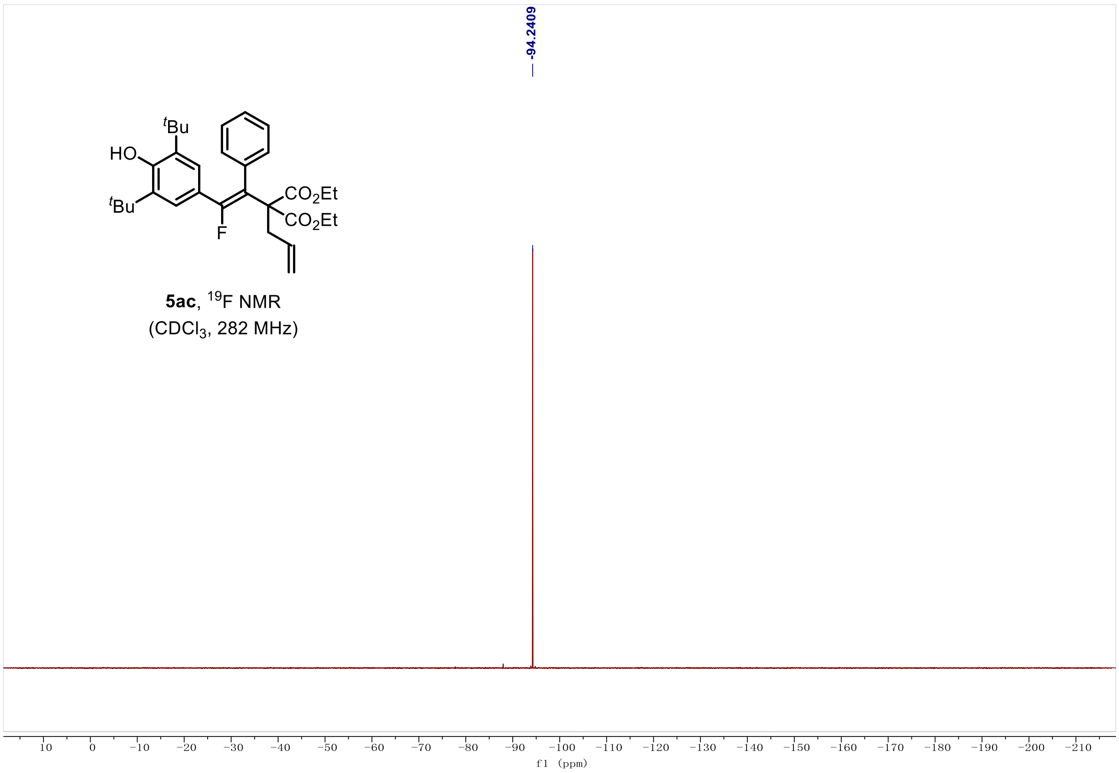
-94.2409
HO
tBu
tBu
CO2Et
CO2Et
F
5ac, 19F NMR
(CDCl3, 282 MHz)
10 0 −10 −20 −30 −40 −50 −60 −70 −80 −90 −100 −110 −120 −130 −140 −150 −160 −170 −180 −190 −200 −210
f1 (ppm)

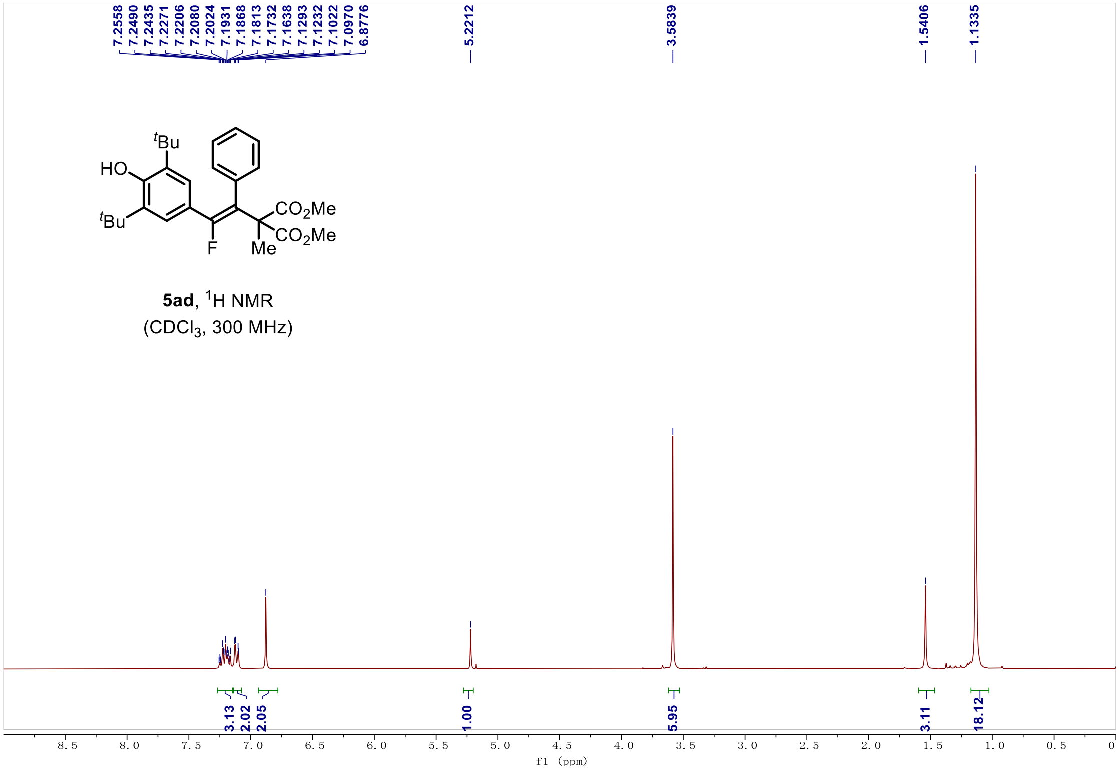
7.2558 7.2490 7.2435 7.2271 7.2206 7.2080 7.2024 7.1931 7.1868 7.1813 7.1732 7.1638 7.1293 7.1232 7.1022 7.0970 6.8776
5.2212
3.5839
1.5406
1.1335
HO
tBu
tBu
CO2Me
CO2Me
F
Me
5ad, 1H NMR
(CDCl3, 300 MHz)
3.13 2.02 2.05
1.00
5.95
3.11
18.12
8.5 8.0 7.5 7.0 6.5 6.0 5.5 5.0 4.5 4.0 3.5 3.0 2.5 2.0 1.5 1.0 0.5 0
f1 (ppm)

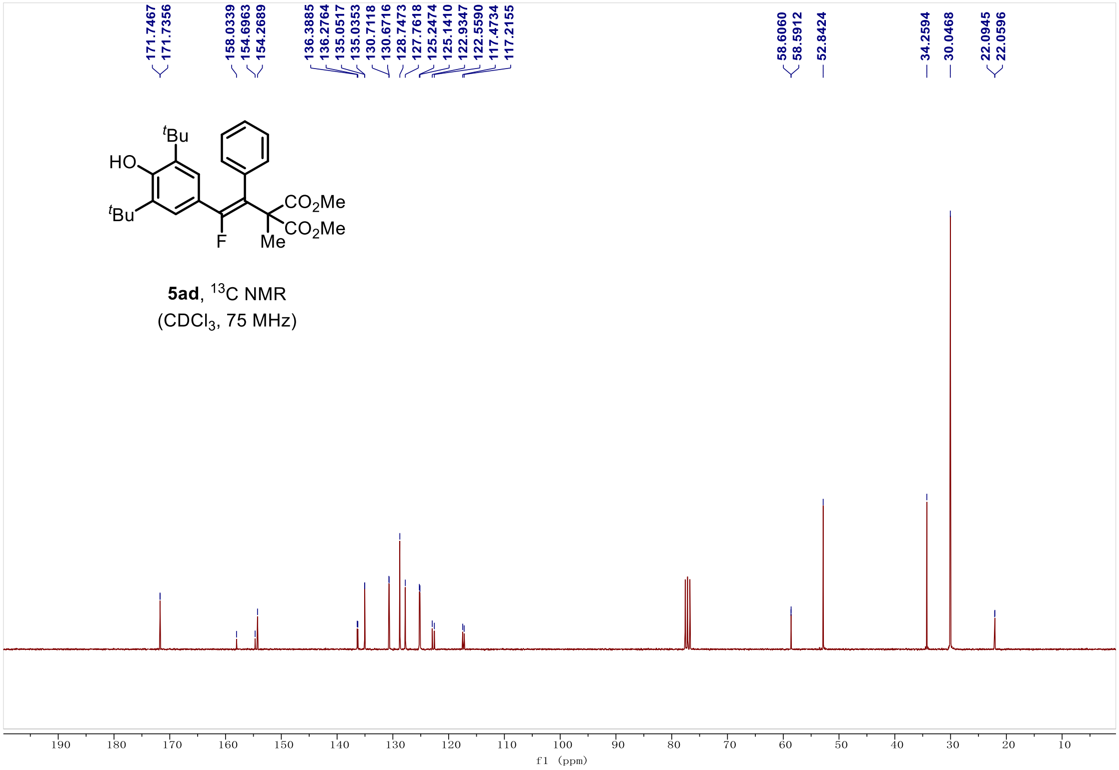

171.7467
171.7356
158.0339
154.6963
154.2689
136.3885
136.2764
135.0517
135.0353
130.7118
130.6716
128.7473
127.7618
125.2474
125.1410
122.9347
122.5590
117.4734
117.2155
58.6060
58.5912
52.8424
34.2594
30.0468
22.0945
22.0596
^{t}Bu
HO
^{t}Bu
CO_2Me
CO_2Me
F
Me
5ad, ^{13}C NMR
($CDCl_3$, 75 MHz)
f1 (ppm)

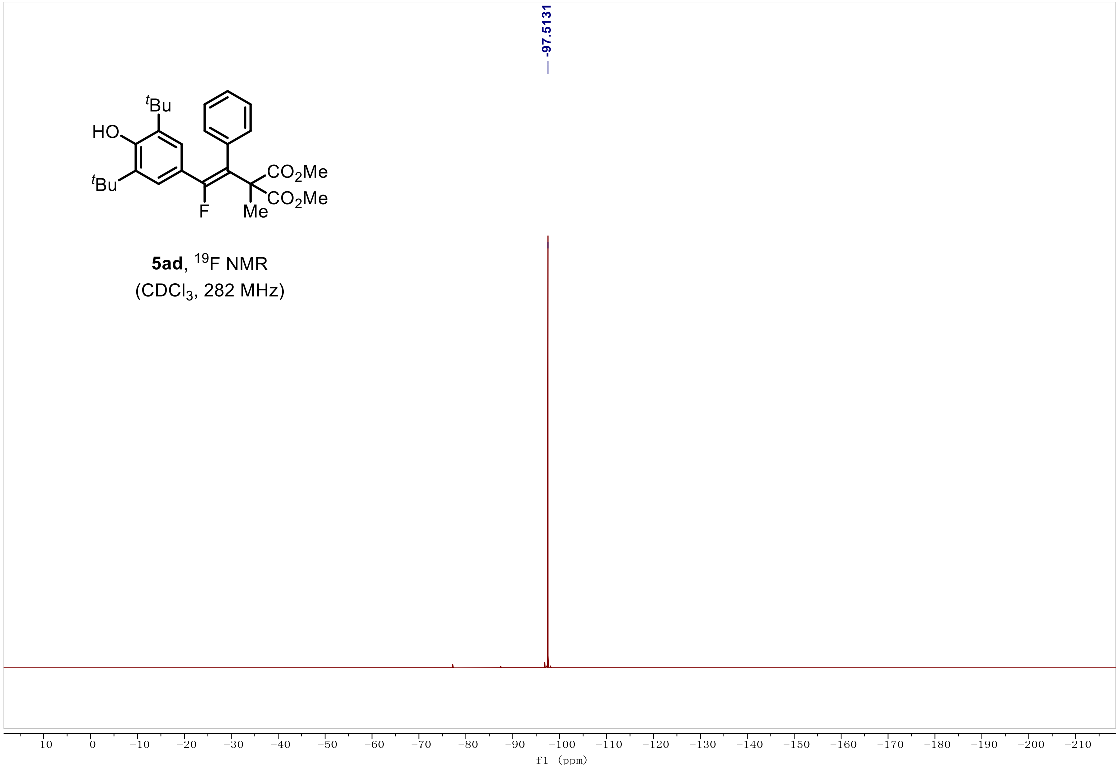

-97.5131
^{t}Bu
HO
^{t}Bu
CO_2Me
CO_2Me
F
Me
5ad, ^{19}F NMR
($CDCl_3$, 282 MHz)
f1 (ppm)

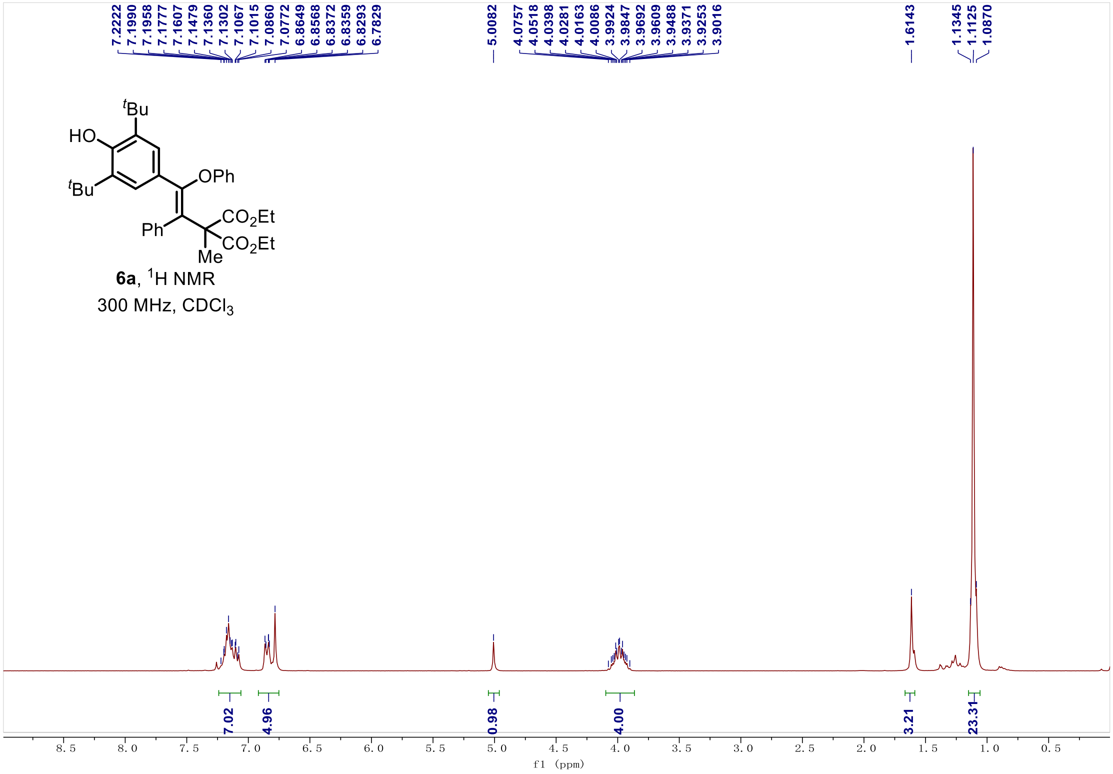
7.2222
7.1990
7.1958
7.1777
7.1607
7.1479
7.1360
7.1302
7.1067
7.1015
7.0860
7.0772
6.8649
6.8568
6.8372
6.8359
6.8293
6.7829
5.0082
4.0757
4.0518
4.0398
4.0281
4.0163
4.0086
3.9924
3.9847
3.9692
3.9609
3.9488
3.9371
3.9253
3.9016
1.6143
1.1345
1.1125
1.0870
HO
tBu
tBu
OPh
Ph
CO2Et
CO2Et
Me
6a, 1H NMR
300 MHz, CDCl3
7.02
4.96
0.98
4.00
3.21
23.31
f1 (ppm)

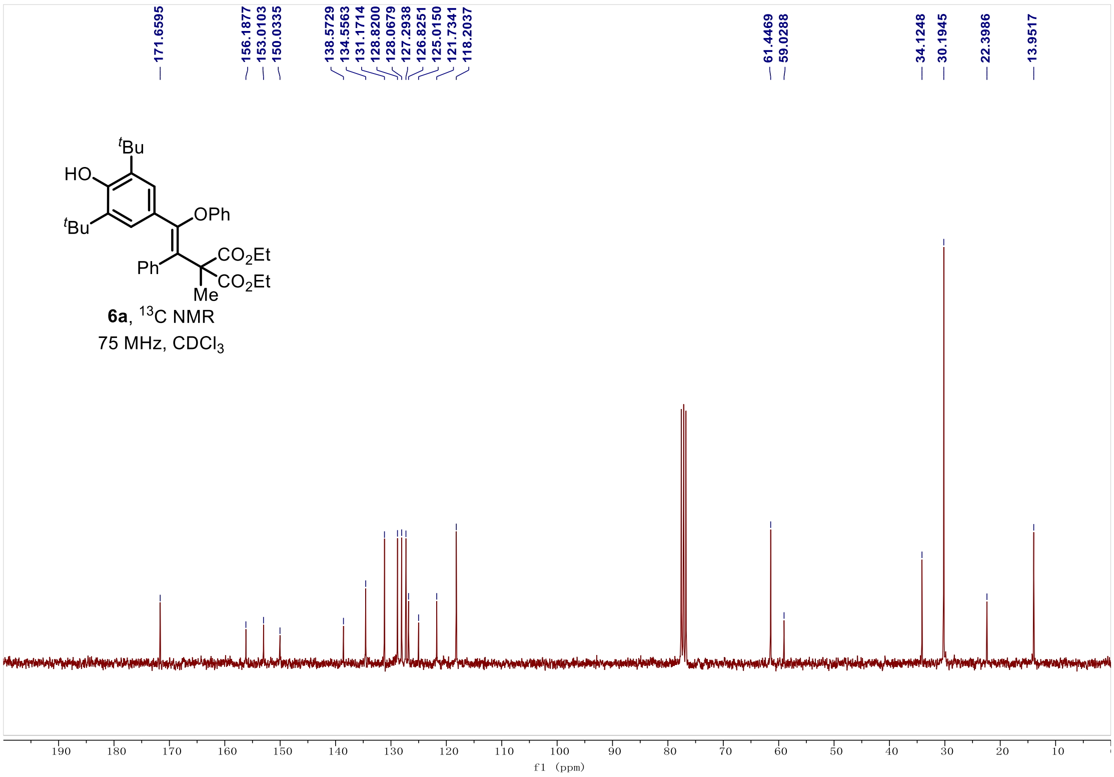
171.6595
156.1877
153.0103
150.0335
138.5729
134.5563
131.1714
128.8200
128.0679
127.2938
126.8251
125.0150
121.7341
118.2037
61.4469
59.0288
34.1248
30.1945
22.3986
13.9517
HO
tBu
tBu
OPh
Ph
CO2Et
CO2Et
Me
6a, 13C NMR
75 MHz, CDCl3
f1 (ppm)

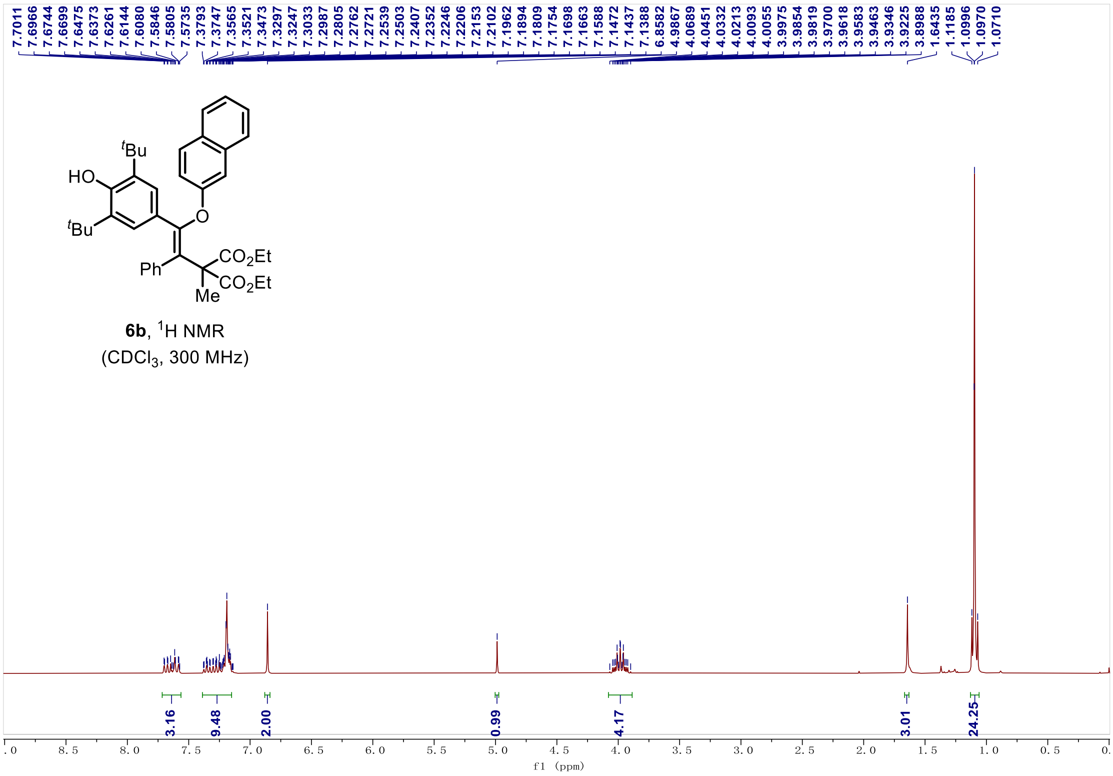

7.7011
7.6966
7.6744
7.6699
7.6475
7.6373
7.6261
7.6144
7.6080
7.5846
7.5805
7.5735
7.3793
7.3747
7.3565
7.3521
7.3473
7.3297
7.3247
7.3033
7.2987
7.2805
7.2762
7.2721
7.2539
7.2503
7.2407
7.2352
7.2246
7.2206
7.2153
7.2102
7.1962
7.1894
7.1809
7.1754
7.1698
7.1663
7.1588
7.1472
7.1437
7.1388
6.8582
4.9867
4.0689
4.0451
4.0332
4.0213
4.0093
4.0055
3.9975
3.9854
3.9819
3.9700
3.9618
3.9583
3.9463
3.9346
3.9225
3.8988
1.6435
1.1185
1.0996
1.0970
1.0710
HO
tBu
tBu
O
Ph
CO2Et
CO2Et
Me
6b, 1H NMR
(CDCl3, 300 MHz)
3.16
9.48
2.00
0.99
4.17
3.01
24.25
f1 (ppm)

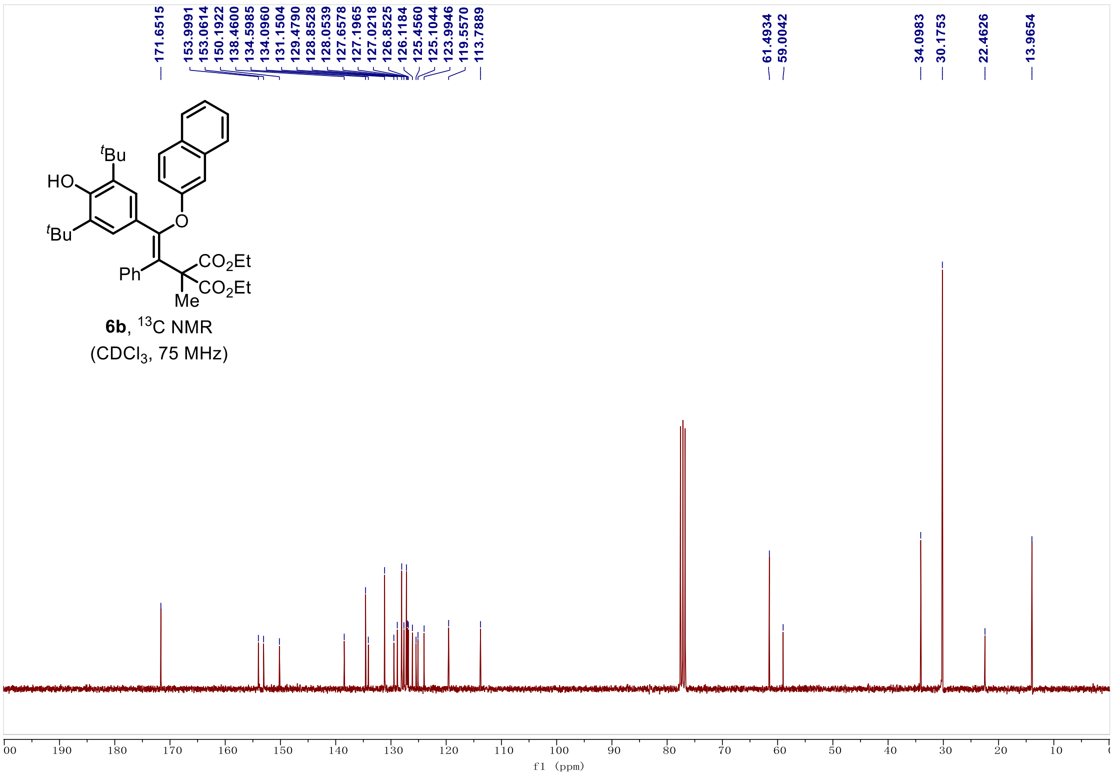

171.6515
153.9991
153.0614
150.1922
138.4600
134.5985
134.0960
131.1504
129.4790
128.8528
128.0539
127.6578
127.1965
127.0218
126.8525
126.1184
125.4560
125.1044
123.9946
119.5570
113.7889
61.4934
59.0042
34.0983
30.1753
22.4626
13.9654
HO
tBu
tBu
O
Ph
CO2Et
CO2Et
Me
6b, 13C NMR
(CDCl3, 75 MHz)
f1 (ppm)

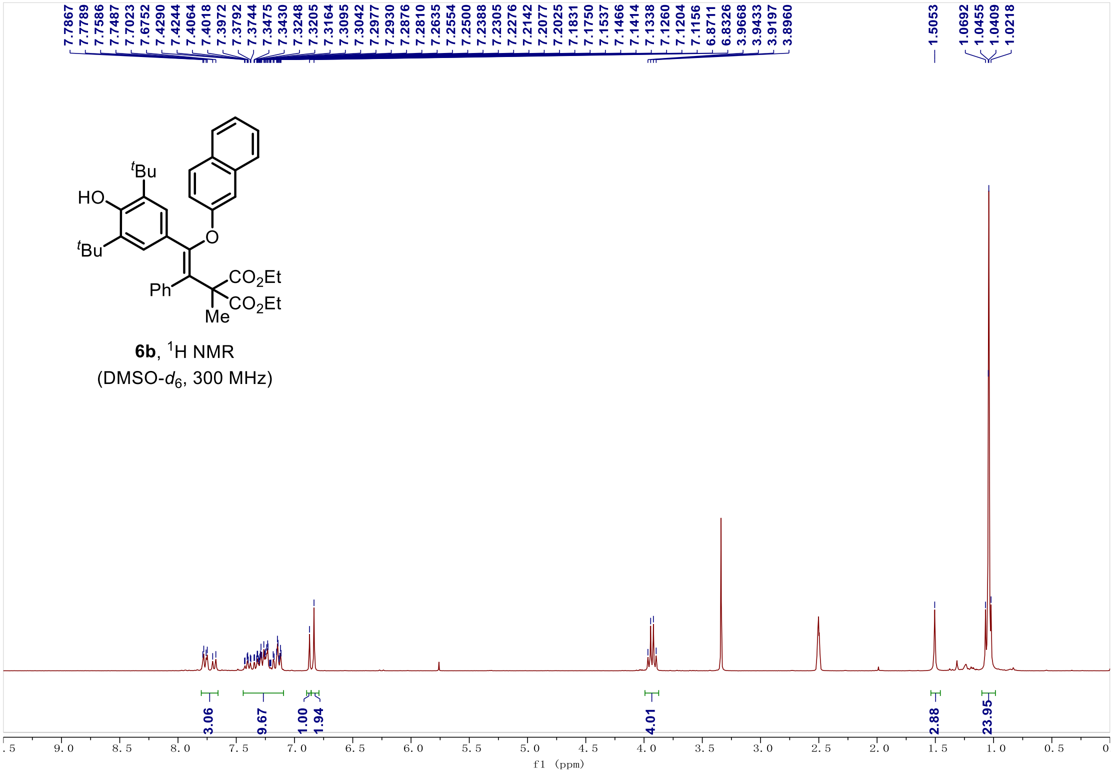
6b, ^{1}H NMR
(DMSO-d_6, 300 MHz)
f1 (ppm)

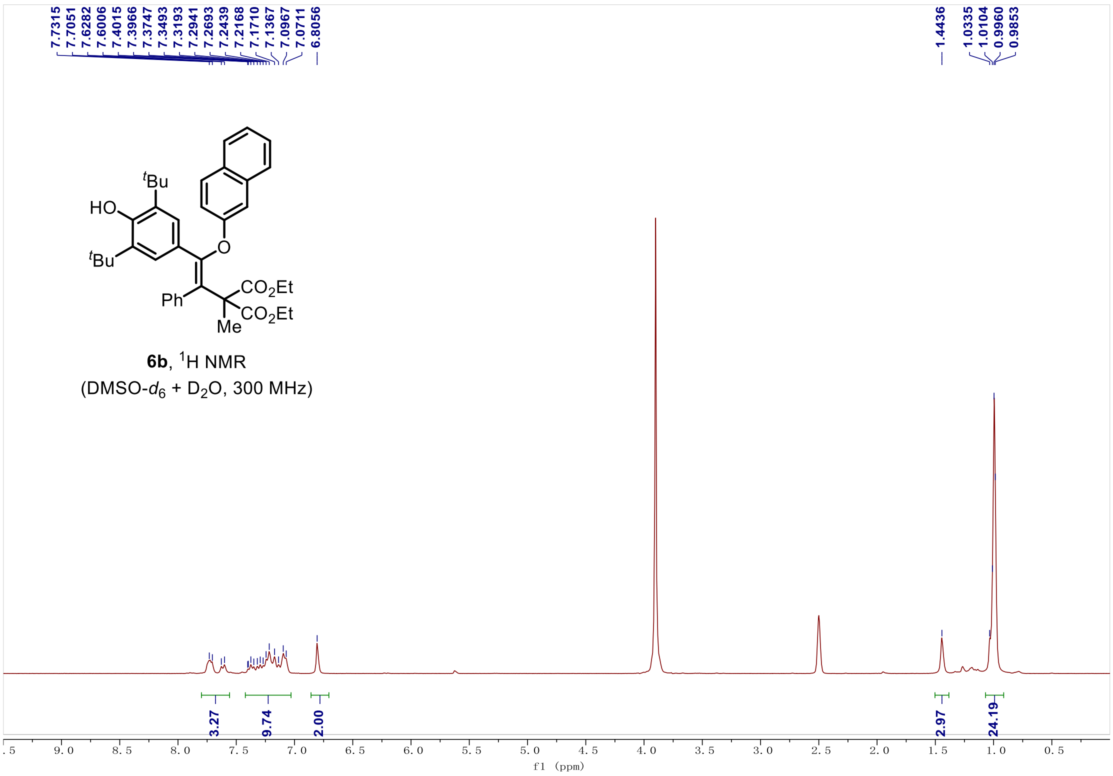
6b, ^{1}H NMR
(DMSO-d_6 + D_2O, 300 MHz)
f1 (ppm)

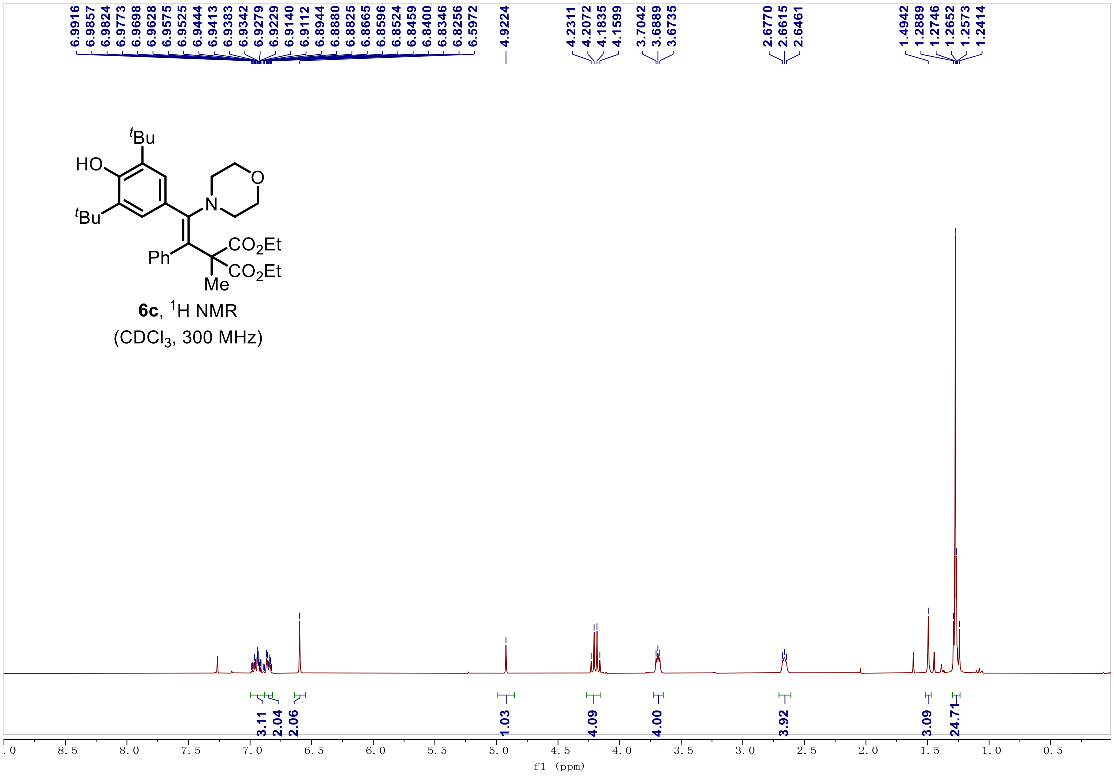
6.9916
6.9857
6.9824
6.9773
6.9698
6.9628
6.9575
6.9525
6.9444
6.9413
6.9383
6.9342
6.9279
6.9229
6.9140
6.9112
6.8944
6.8880
6.8825
6.8665
6.8596
6.8524
6.8459
6.8400
6.8346
6.8256
6.5972
4.9224
4.2311
4.2072
4.1835
4.1599
3.7042
3.6889
3.6735
2.6770
2.6615
2.6461
1.4942
1.2889
1.2746
1.2652
1.2573
1.2414
HO
tBu
tBu
N
O
CO2Et
CO2Et
Ph
Me
6c, 1H NMR
(CDCl3, 300 MHz)
3.11
2.04
2.06
1.03
4.09
4.00
3.92
3.09
24.71
8.5
8.0
7.5
7.0
6.5
6.0
5.5
5.0
4.5
4.0
3.5
3.0
2.5
2.0
1.5
1.0
0.5
f1 (ppm)

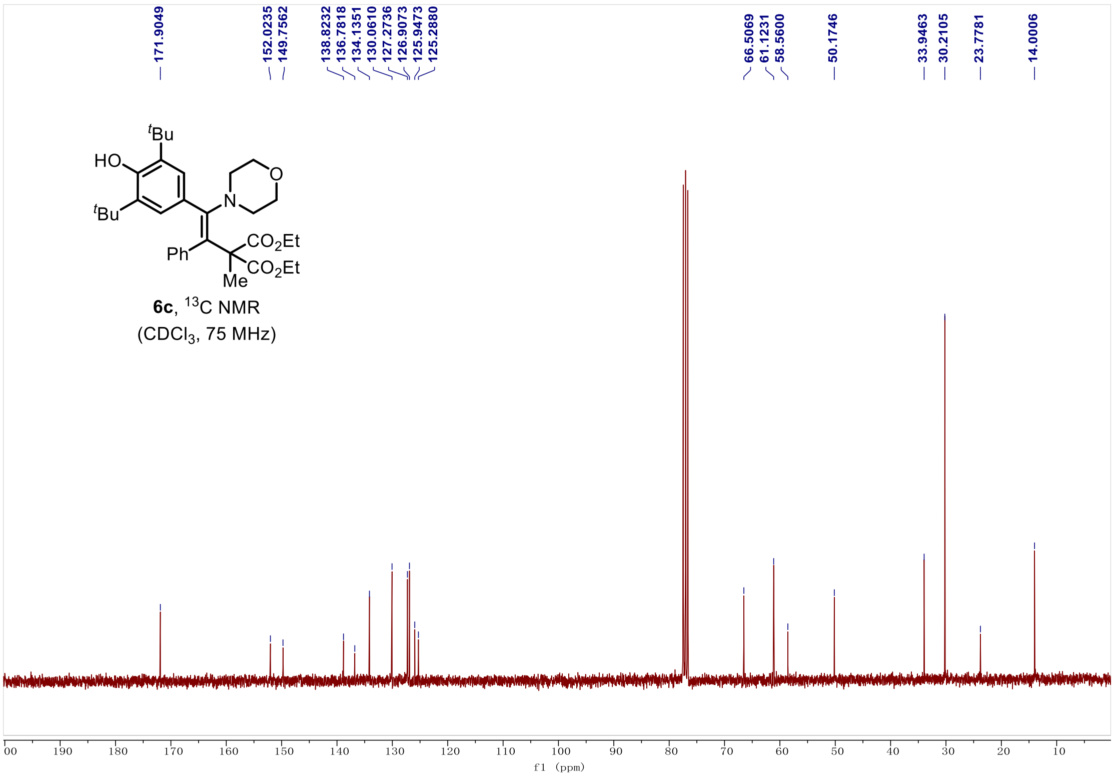
171.9049
152.0235
149.7562
138.8232
136.7818
134.1351
130.0610
127.2736
126.9073
125.9473
125.2880
66.5069
61.1231
58.5600
50.1746
33.9463
30.2105
23.7781
14.0006
HO
tBu
tBu
N
O
CO2Et
CO2Et
Ph
Me
6c, 13C NMR
(CDCl3, 75 MHz)
190
180
170
160
150
140
130
120
110
100
90
80
70
60
50
40
30
20
10
f1 (ppm)

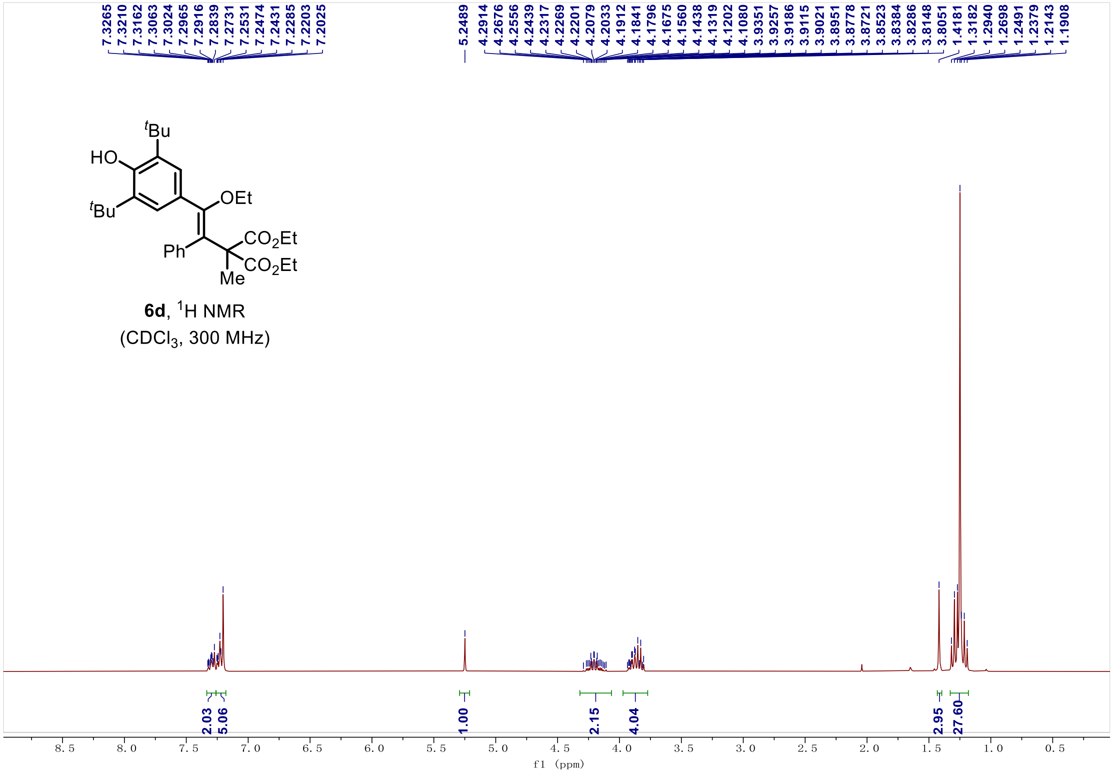
7.3265
7.3210
7.3162
7.3063
7.3024
7.2965
7.2916
7.2839
7.2731
7.2531
7.2474
7.2431
7.2285
7.2203
7.2025
5.2489
4.2914
4.2676
4.2556
4.2439
4.2317
4.2269
4.2201
4.2079
4.2033
4.1912
4.1841
4.1796
4.1675
4.1560
4.1438
4.1319
4.1202
4.1080
3.9351
3.9257
3.9186
3.9115
3.9021
3.8951
3.8778
3.8721
3.8523
3.8384
3.8286
3.8148
3.8051
1.4181
1.3182
1.2940
1.2698
1.2491
1.2379
1.2143
1.1908
6d, 1H NMR
(CDCl3, 300 MHz)
2.03
5.06
1.00
2.15
4.04
2.95
27.60
f1 (ppm)

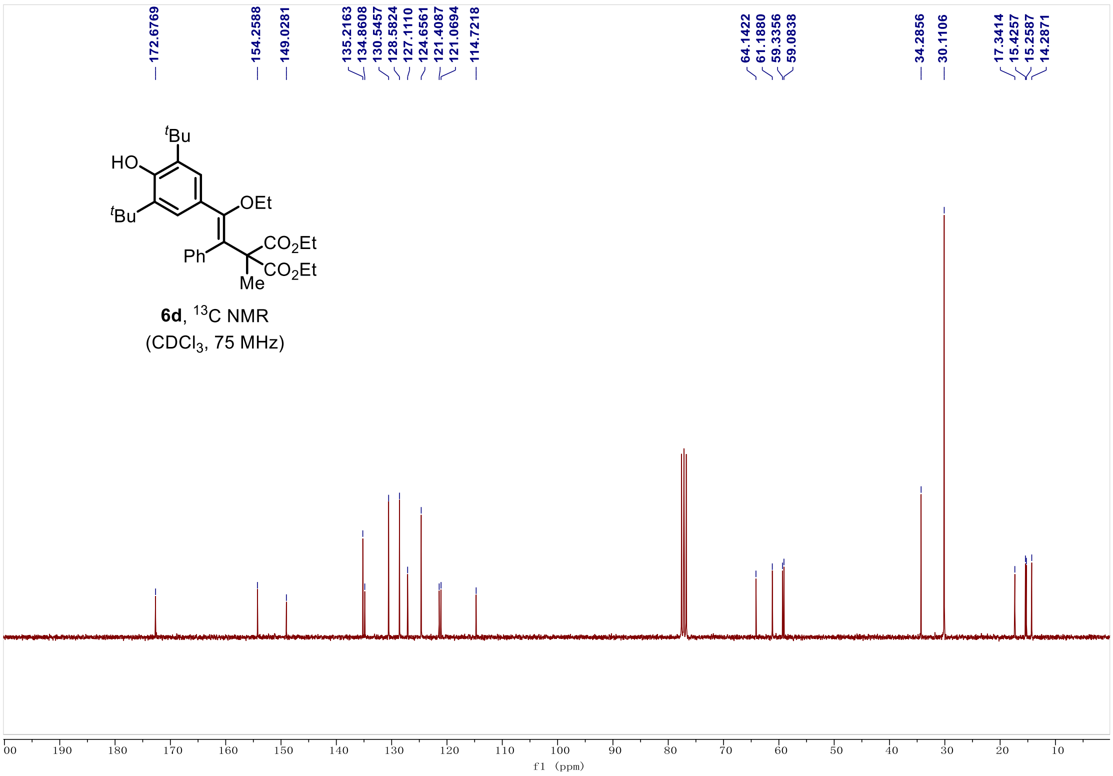
172.6769
154.2588
149.0281
135.2163
134.8608
130.5457
128.5824
127.1110
124.6561
121.4087
121.0694
114.7218
64.1422
61.1880
59.3356
59.0838
34.2856
30.1106
17.3414
15.4257
15.2587
14.2871
6d, 13C NMR
(CDCl3, 75 MHz)
f1 (ppm)

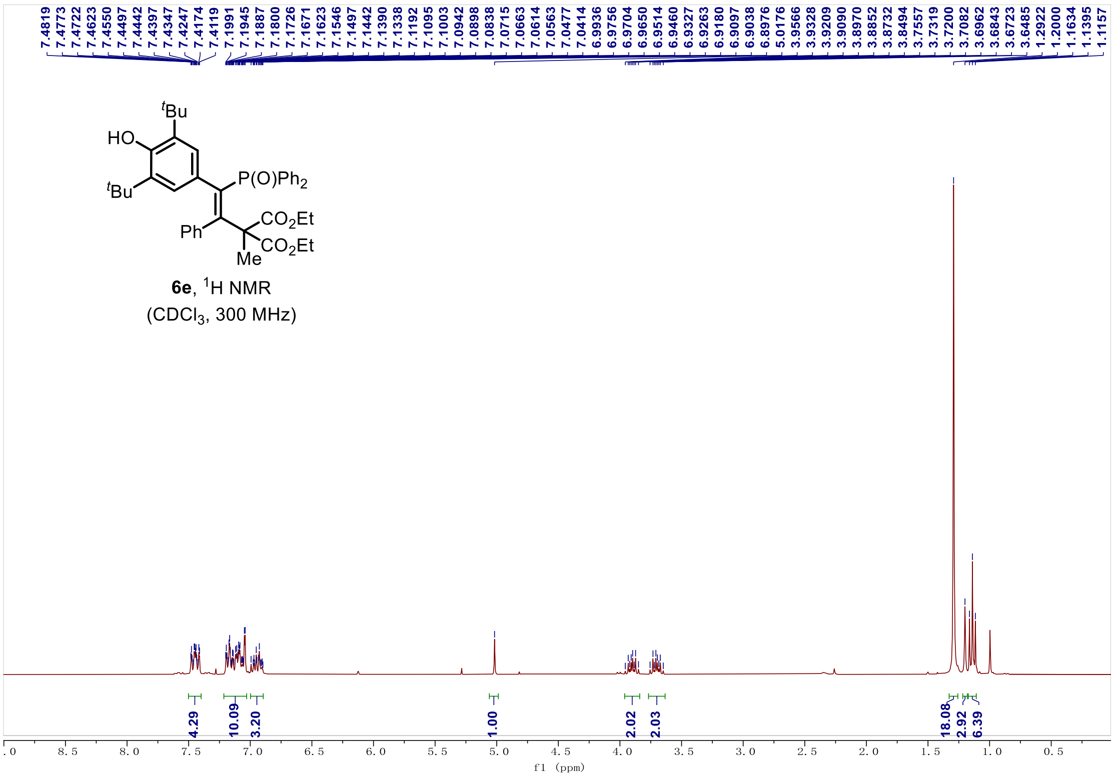
6e, 1H NMR
(CDCl3, 300 MHz)
f1 (ppm)

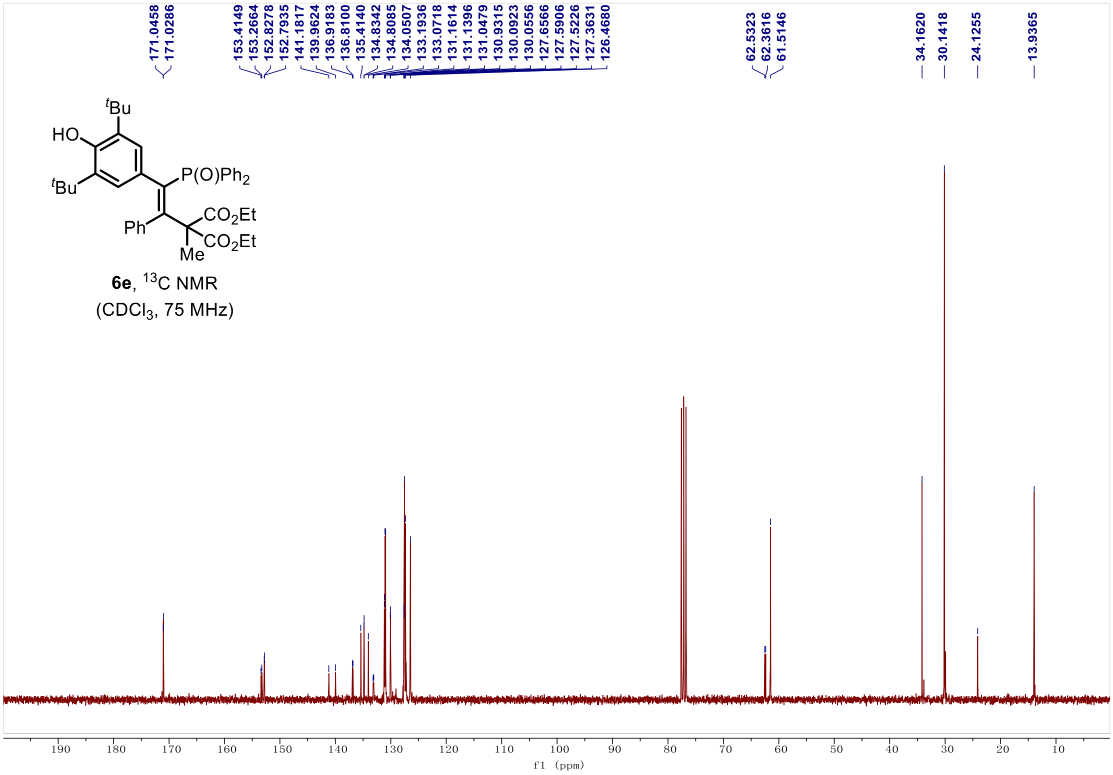
6e, 13C NMR
(CDCl3, 75 MHz)
f1 (ppm)

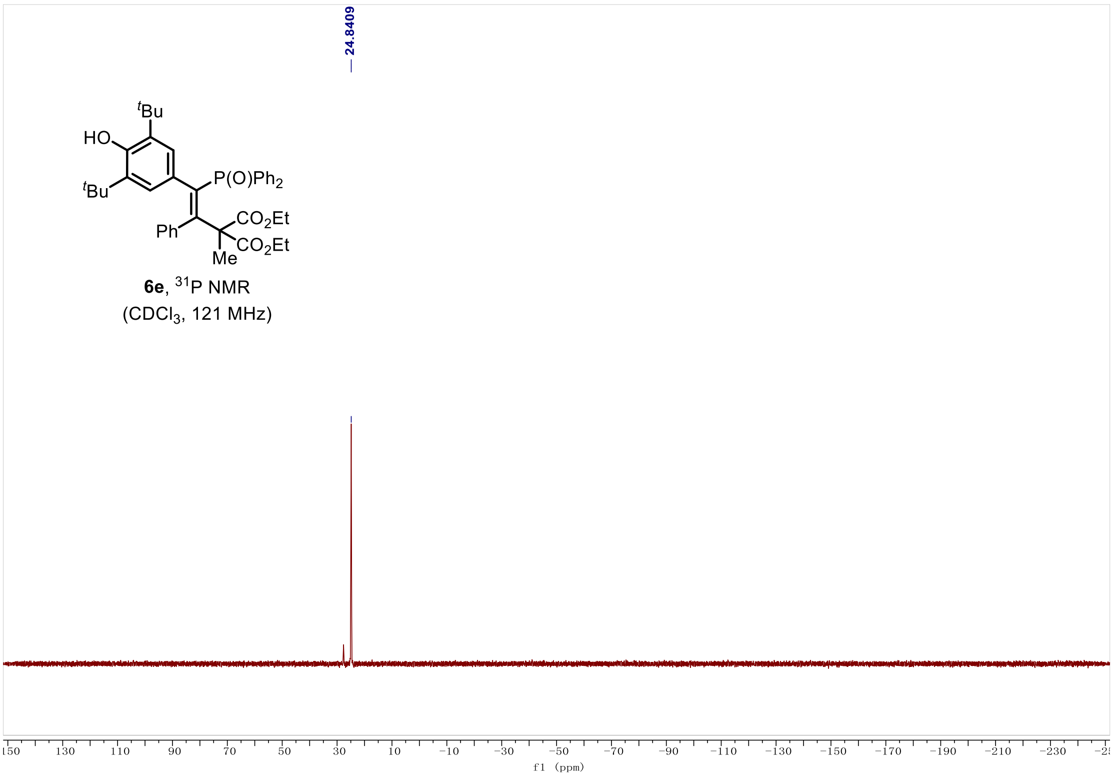

6e, ^{31}P NMR
($CDCl_3$, 121 MHz)

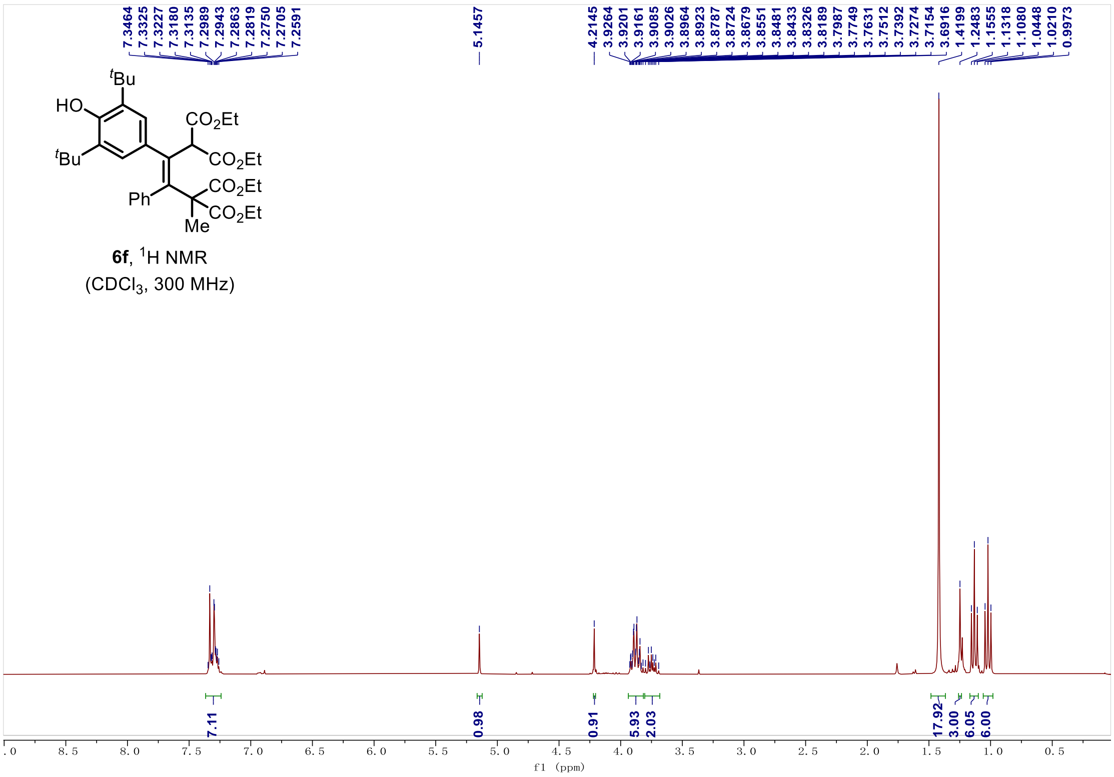

6f, ^{1}H NMR
($CDCl_3$, 300 MHz)

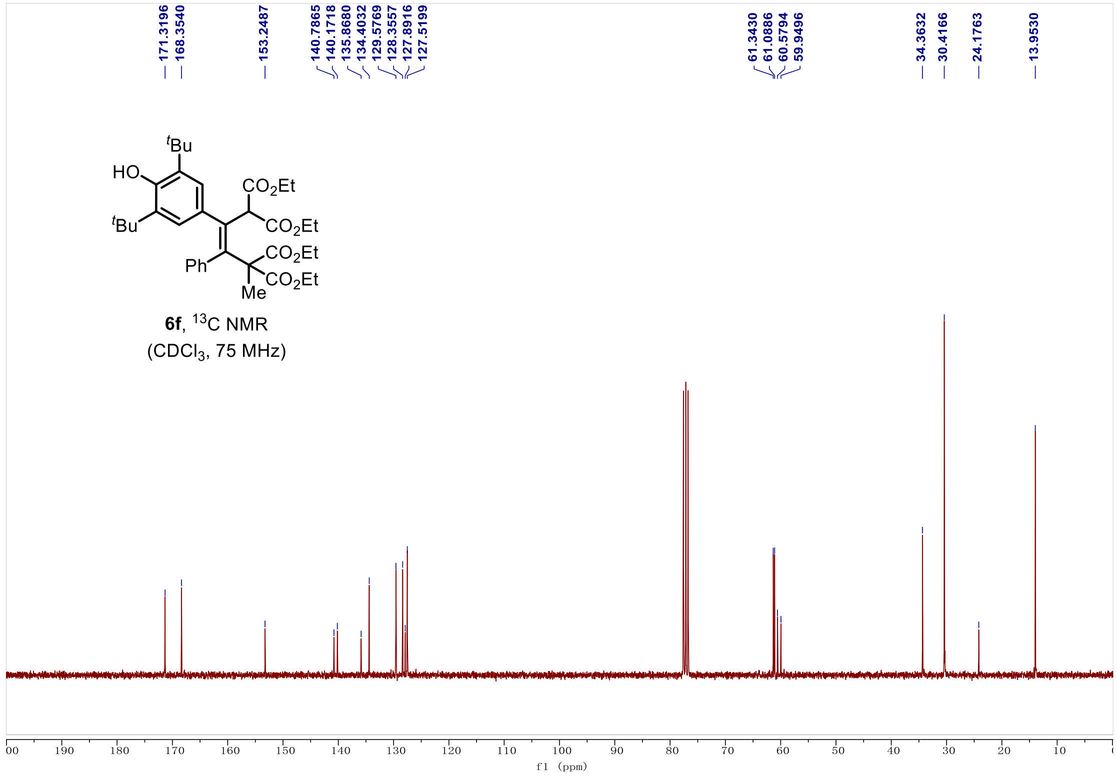
171.3196
168.3540
153.2487
140.7865
140.1718
135.8680
134.4032
129.5769
128.3557
127.8916
127.5199
61.3430
61.0886
60.5794
59.9496
34.3632
30.4166
24.1763
13.9530
6f, 13C NMR
(CDCl3, 75 MHz)
f1 (ppm)

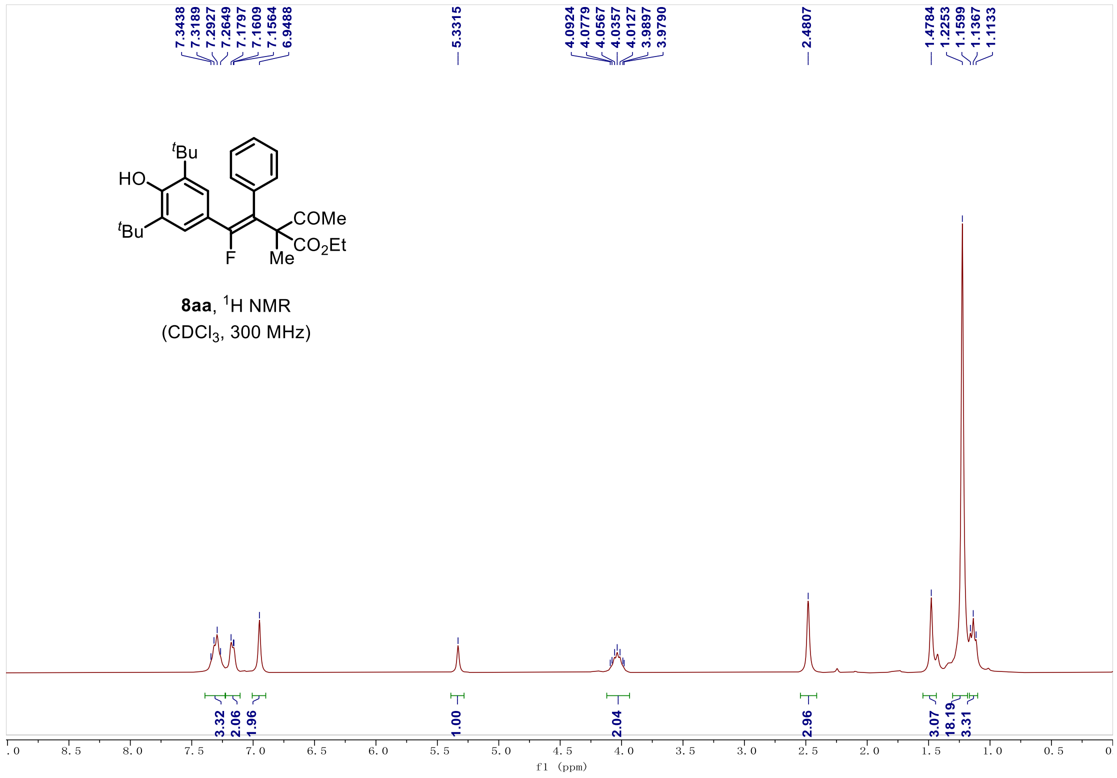
7.3438
7.3189
7.2927
7.2649
7.1797
7.1609
7.1564
6.9488
5.3315
4.0924
4.0779
4.0567
4.0357
4.0127
3.9897
3.9790
2.4807
1.4784
1.2253
1.1599
1.1367
1.1133
8aa, 1H NMR
(CDCl3, 300 MHz)
3.32
2.06
1.96
1.00
2.04
2.96
3.07
18.19
3.31
f1 (ppm)

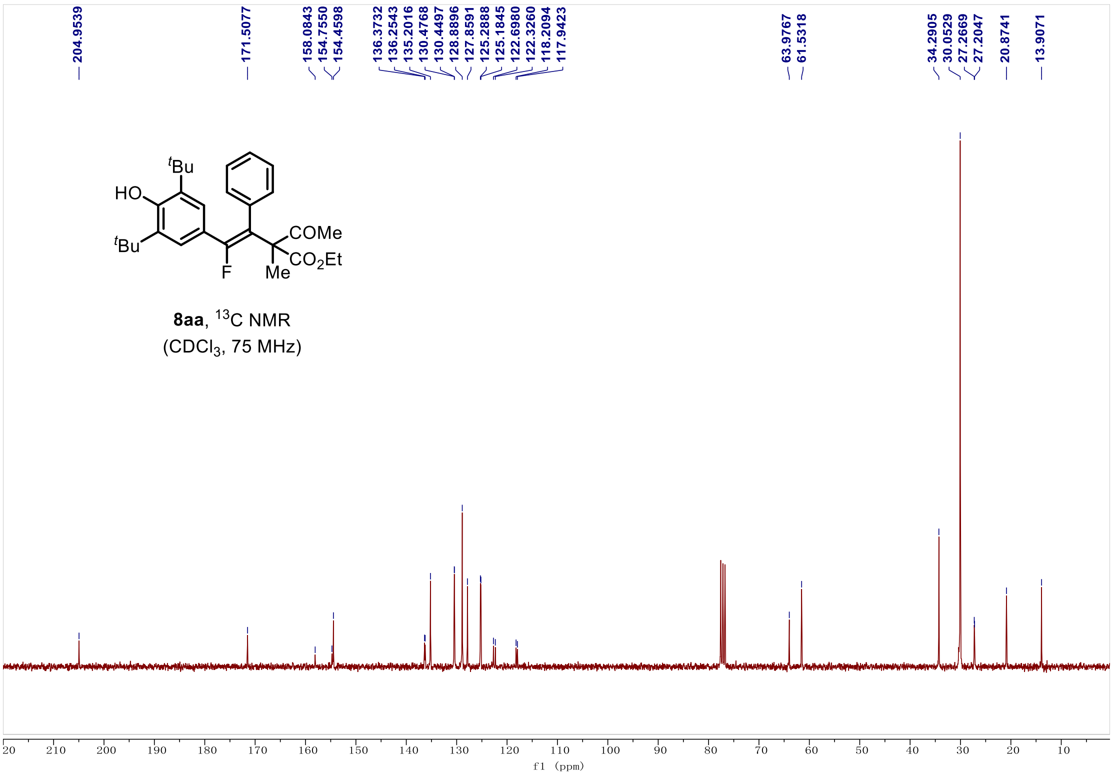
204.9539
171.5077
158.0843
154.7550
154.4598
136.3732
136.2543
135.2016
130.4768
130.4497
128.8896
127.8591
125.2888
125.1845
122.6980
122.3260
118.2094
117.9423
63.9767
61.5318
34.2905
30.0529
27.2669
27.2047
20.8741
13.9071
8aa, 13C NMR
(CDCl3, 75 MHz)
f1 (ppm)

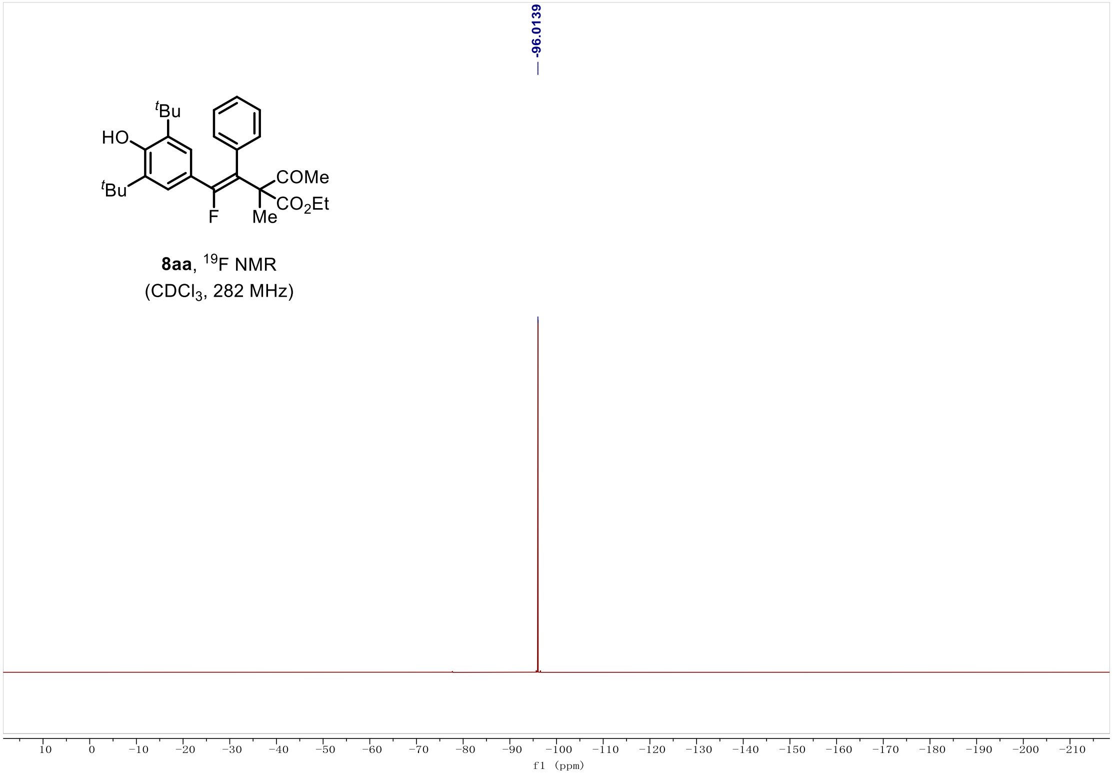
-96.0139
8aa, 19F NMR
(CDCl3, 282 MHz)
f1 (ppm)

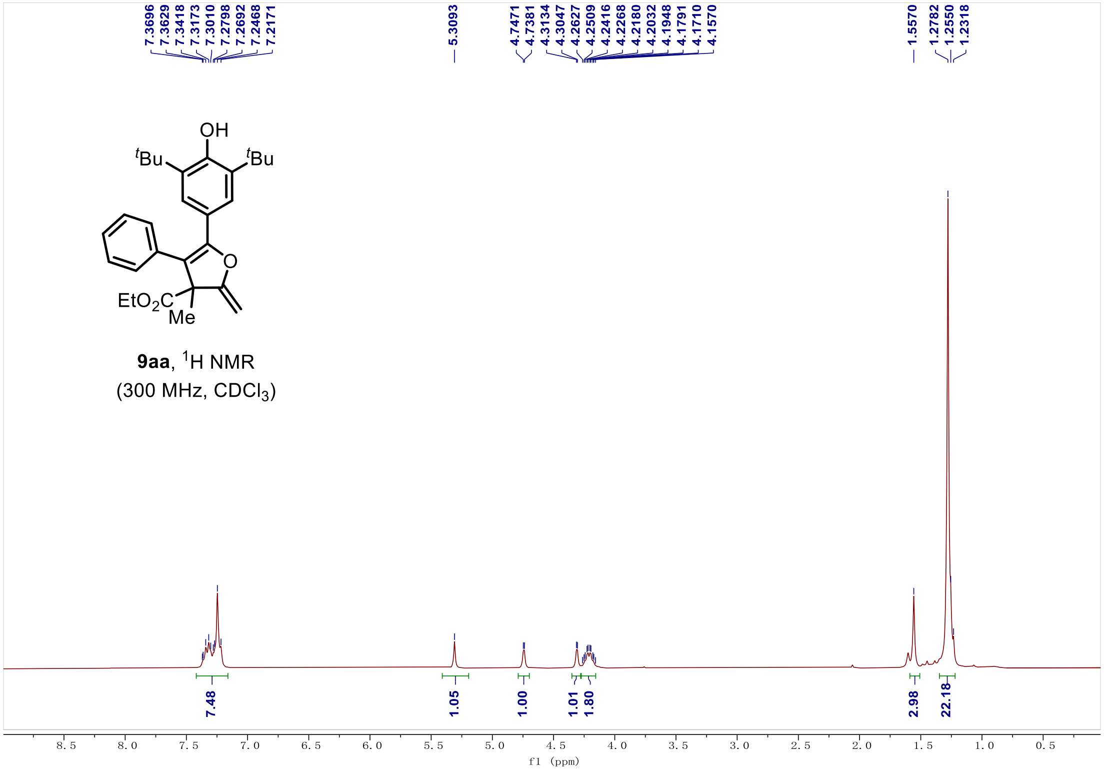

9aa, ^{1}H NMR
(300 MHz, $CDCl_3$)

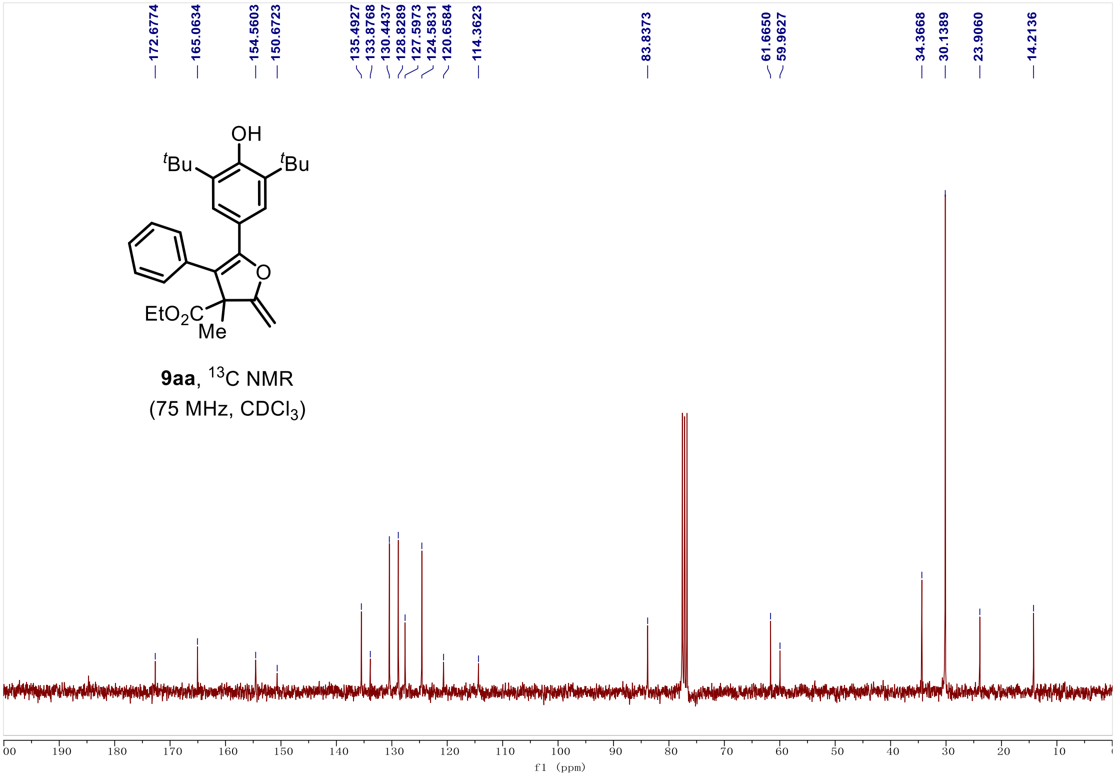

9aa, ^{13}C NMR
(75 MHz, $CDCl_3$)

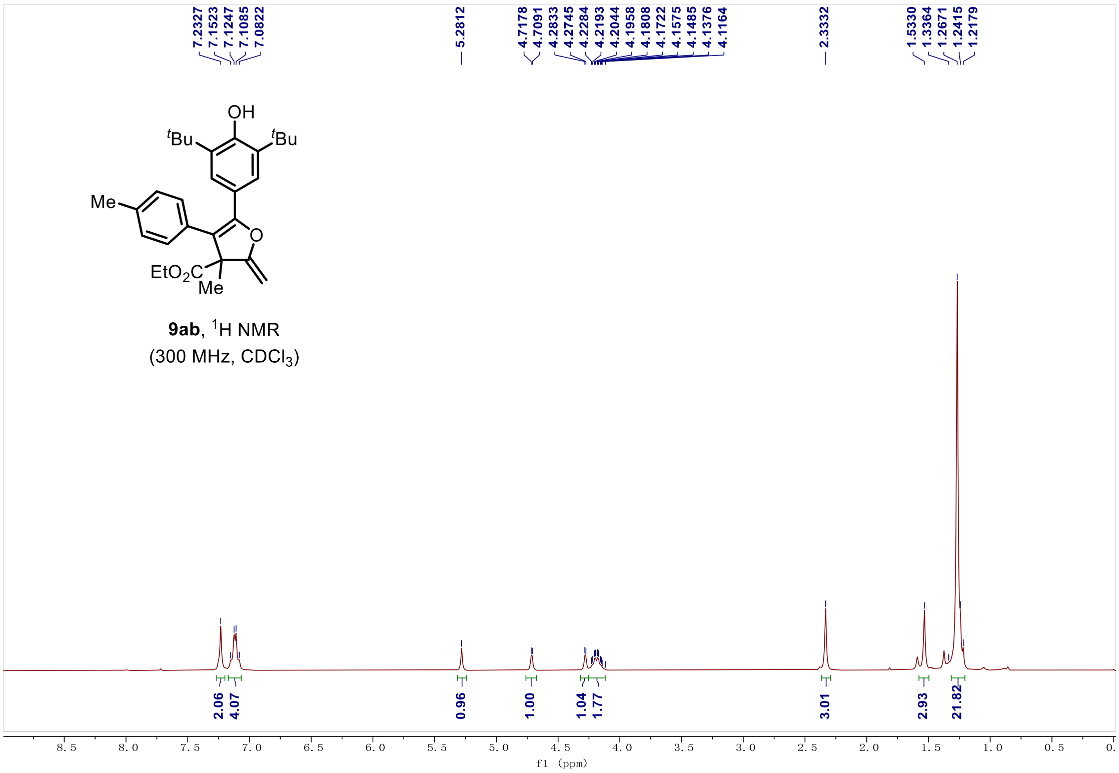
7.2327
7.1523
7.1247
7.1085
7.0822
5.2812
4.7178
4.7091
4.2833
4.2745
4.2284
4.2193
4.2044
4.1958
4.1808
4.1722
4.1575
4.1485
4.1376
4.1164
2.3332
1.5330
1.3364
1.2671
1.2415
1.2179
OH
tBu
tBu
Me
O
EtO2C
Me
9ab, 1H NMR
(300 MHz, CDCl3)
2.06
4.07
0.96
1.00
1.04
1.77
3.01
2.93
21.82
f1 (ppm)

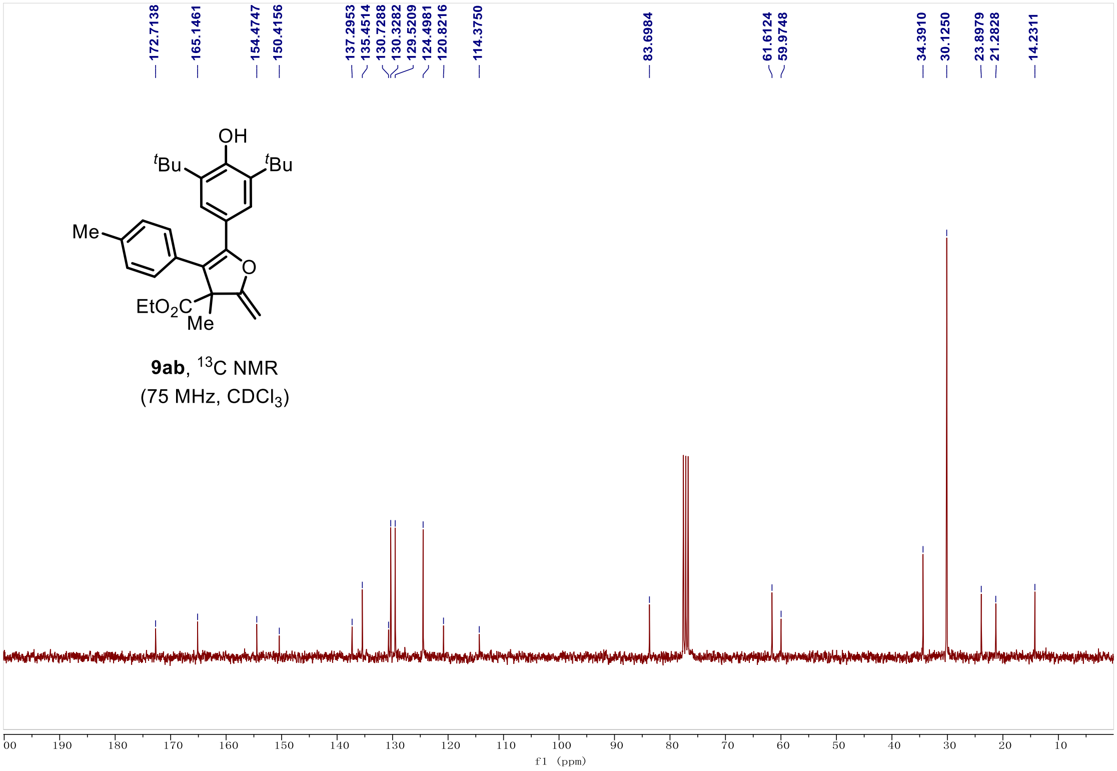
172.7138
165.1461
154.4747
150.4156
137.2953
135.4514
130.7288
130.3282
129.5209
124.4981
120.8216
114.3750
83.6984
61.6124
59.9748
34.3910
30.1250
23.8979
21.2828
14.2311
OH
tBu
tBu
Me
O
EtO2C
Me
9ab, 13C NMR
(75 MHz, CDCl3)
f1 (ppm)

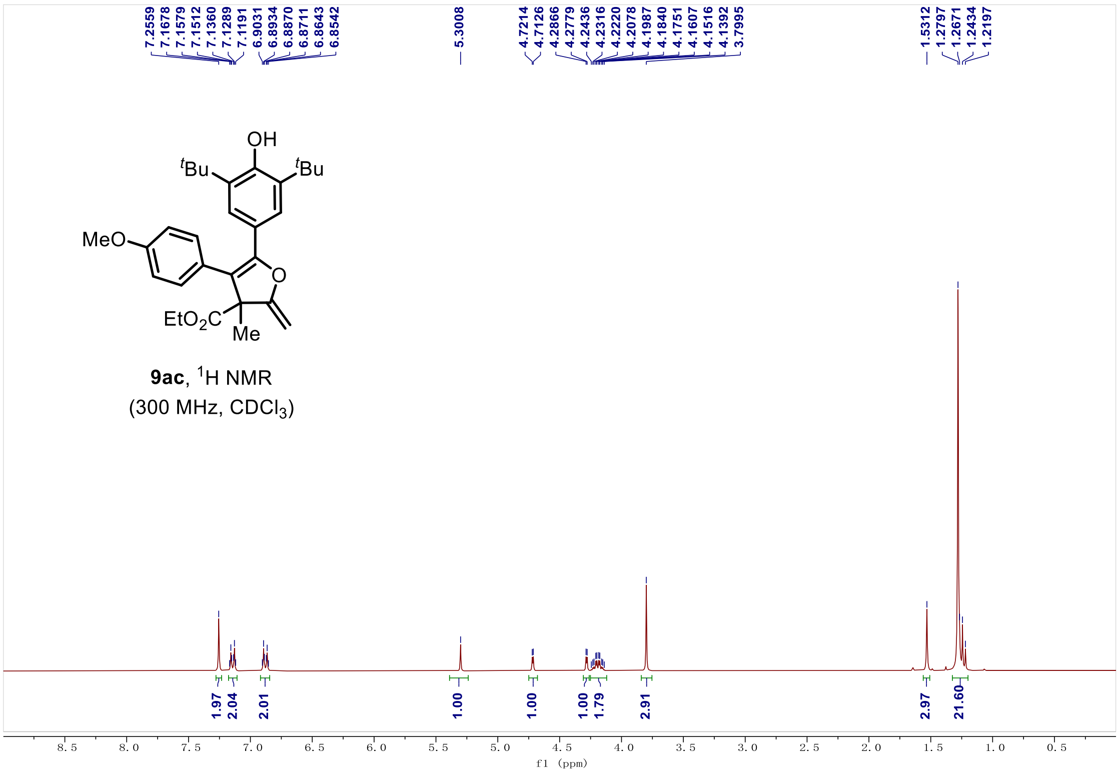

9ac, ^{1}H NMR
(300 MHz, $CDCl_3$)

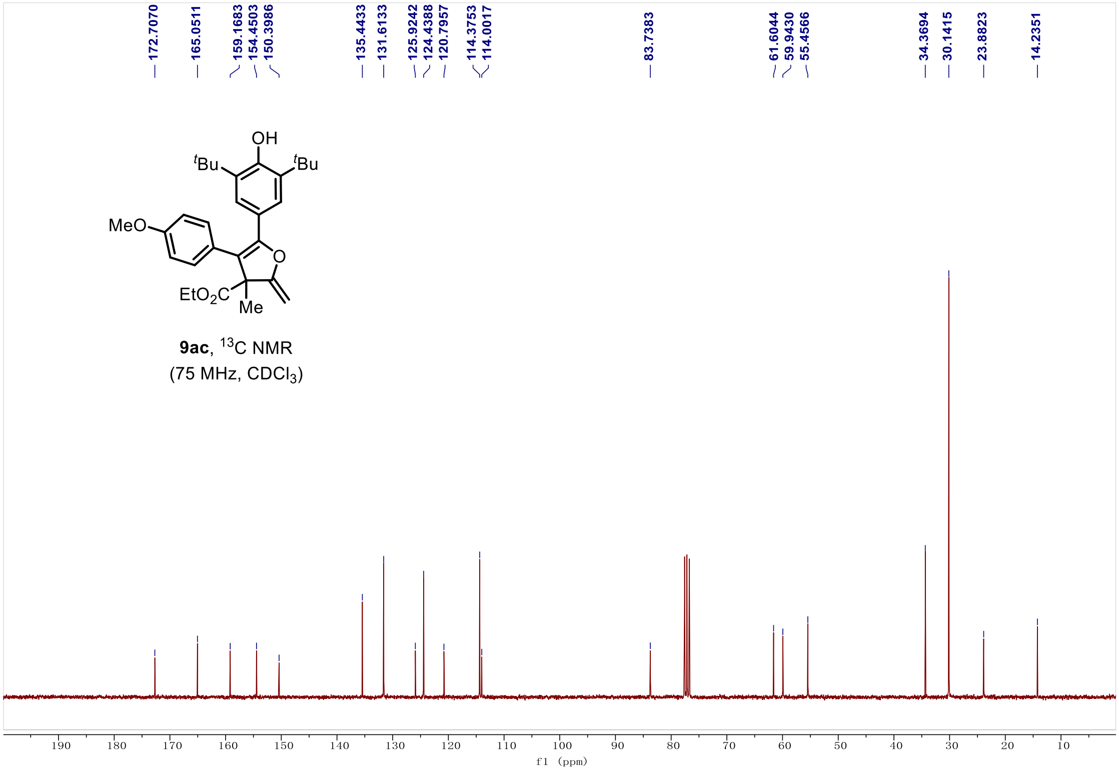

9ac, ^{13}C NMR
(75 MHz, $CDCl_3$)

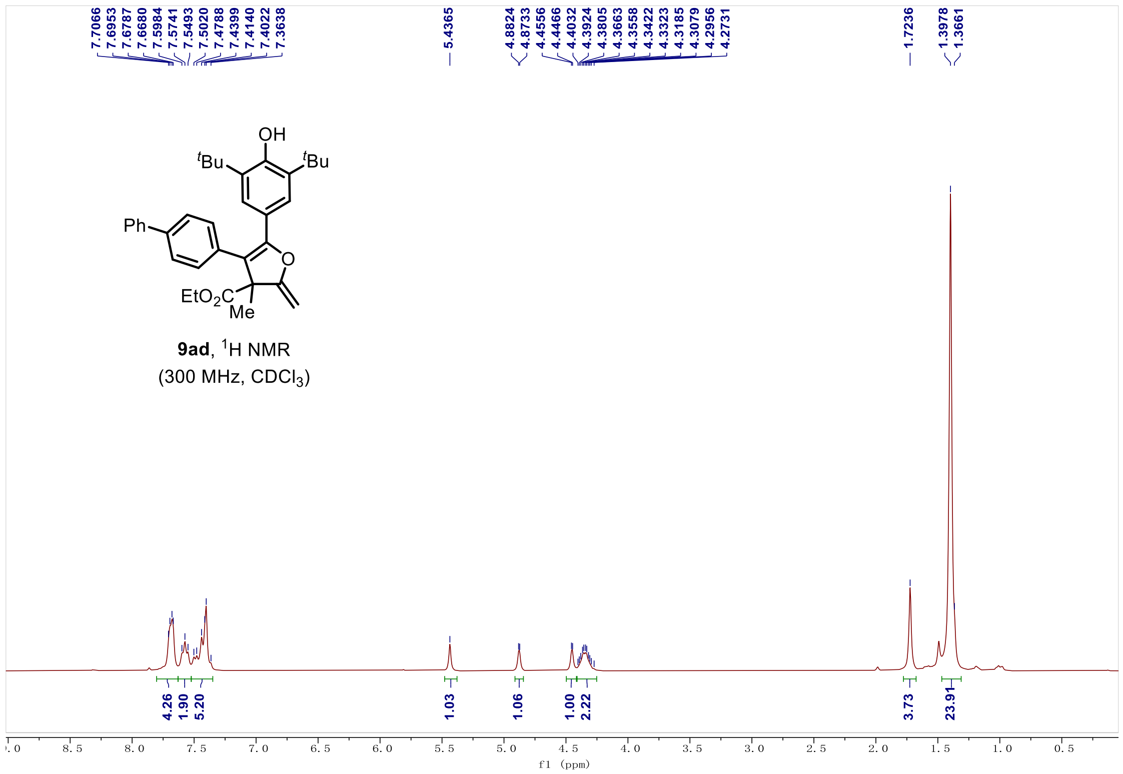
7.7066
7.6953
7.6787
7.6680
7.5984
7.5741
7.5493
7.5020
7.4788
7.4399
7.4140
7.4022
7.3638
5.4365
4.8824
4.8733
4.4556
4.4466
4.4032
4.3924
4.3805
4.3663
4.3558
4.3422
4.3323
4.3185
4.3079
4.2956
4.2731
1.7236
1.3978
1.3661
OH
tBu
tBu
Ph
O
EtO2C
Me
9ad, 1H NMR
(300 MHz, CDCl3)
4.26
1.90
5.20
1.03
1.06
1.00
2.22
3.73
23.91
f1 (ppm)

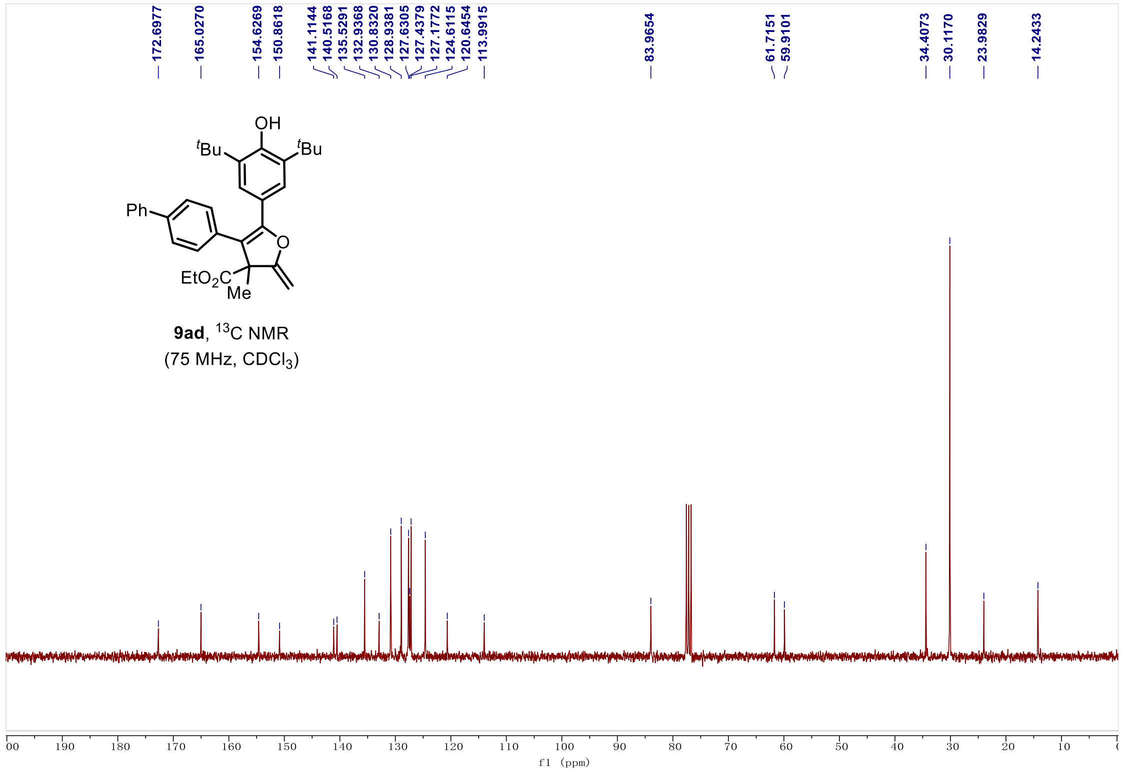
172.6977
165.0270
154.6269
150.8618
141.1144
140.5168
135.5291
132.9368
130.8320
128.9381
127.6305
127.4379
127.1772
124.6115
120.6454
113.9915
83.9654
61.7151
59.9101
34.4073
30.1170
23.9829
14.2433
OH
tBu
tBu
Ph
O
EtO2C
Me
9ad, 13C NMR
(75 MHz, CDCl3)
f1 (ppm)

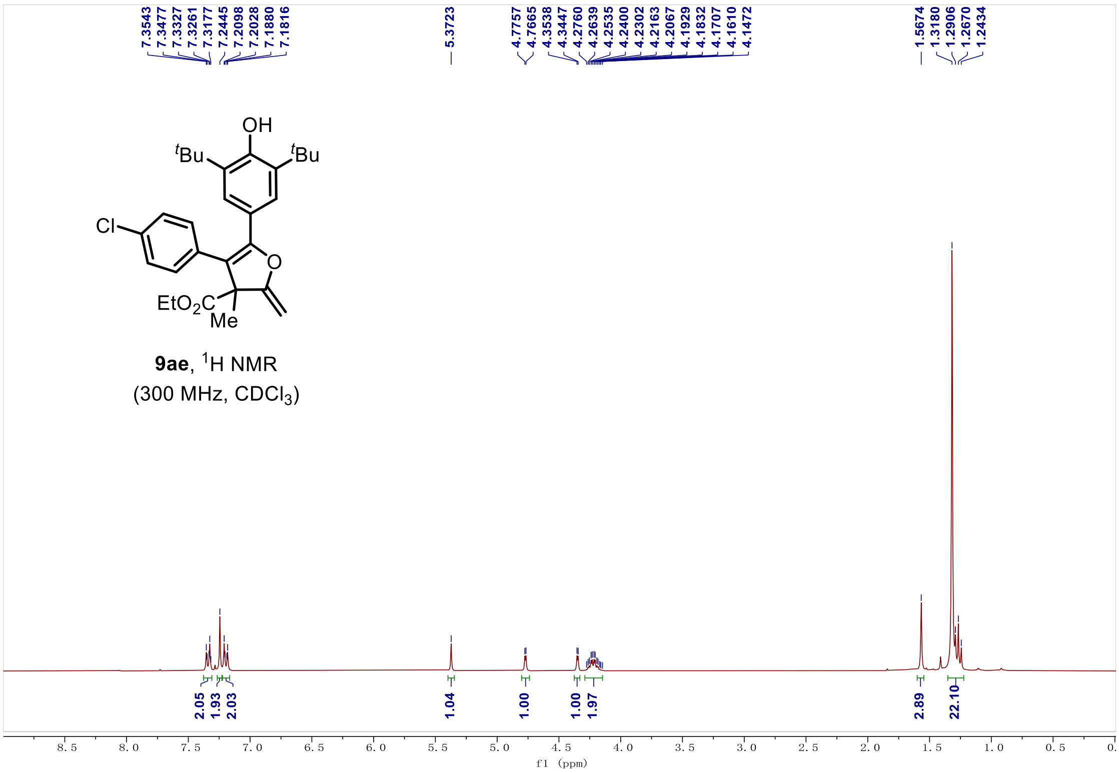

9ae, ^{1}H NMR
(300 MHz, $CDCl_3$)

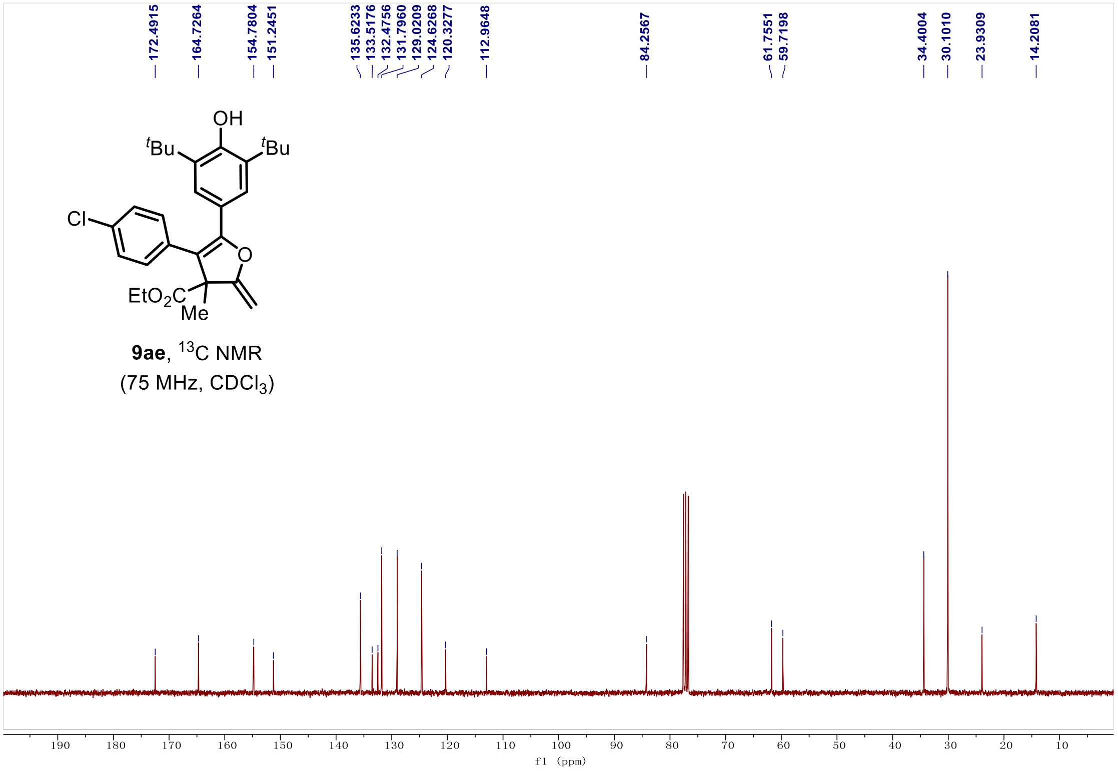

9ae, ^{13}C NMR
(75 MHz, $CDCl_3$)

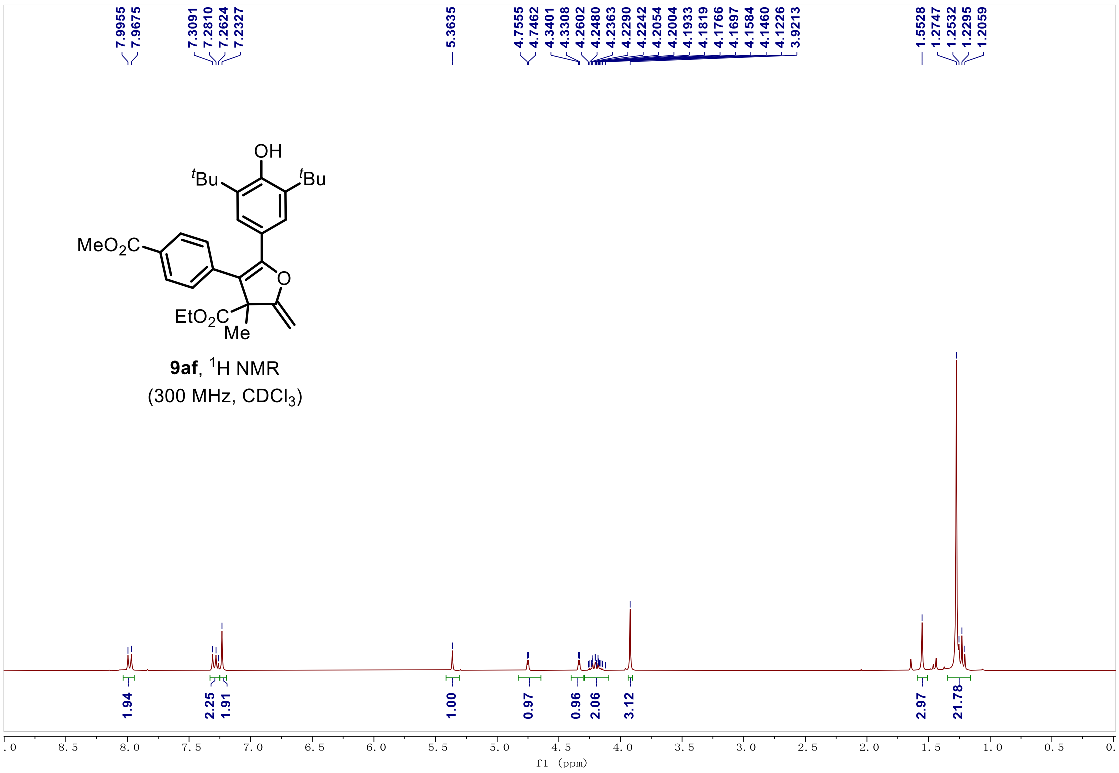
9af, 1H NMR
(300 MHz, CDCl3)
f1 (ppm)

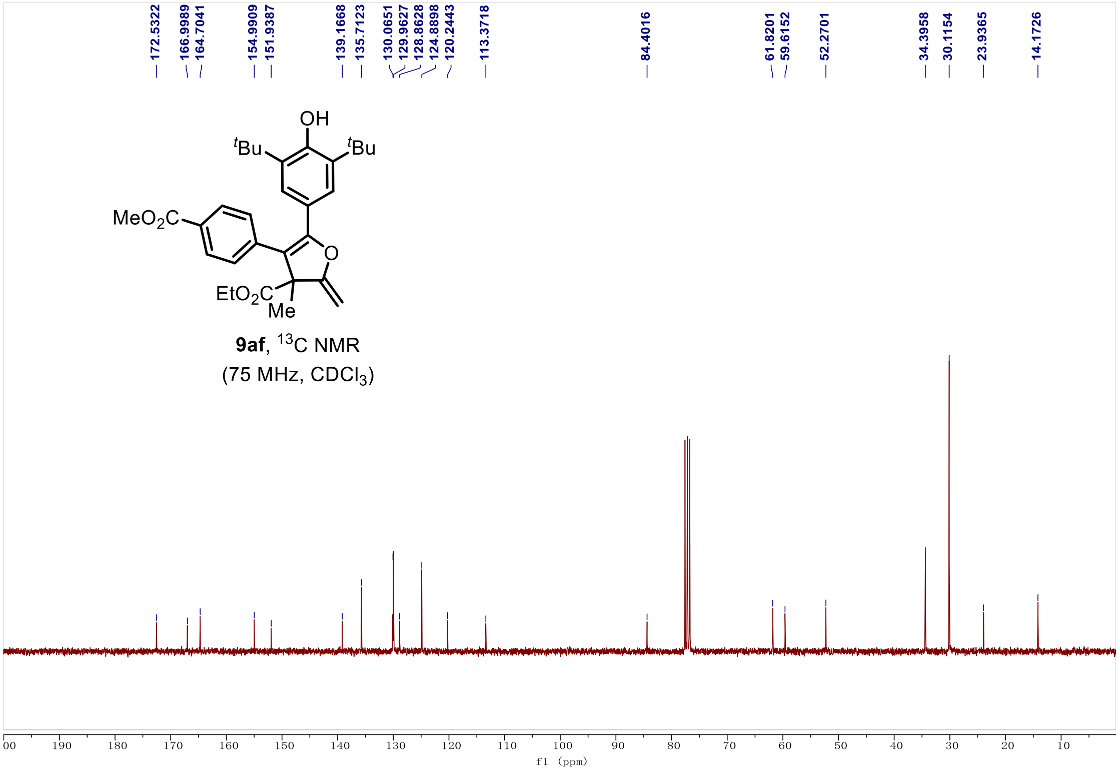
9af, 13C NMR
(75 MHz, CDCl3)
f1 (ppm)

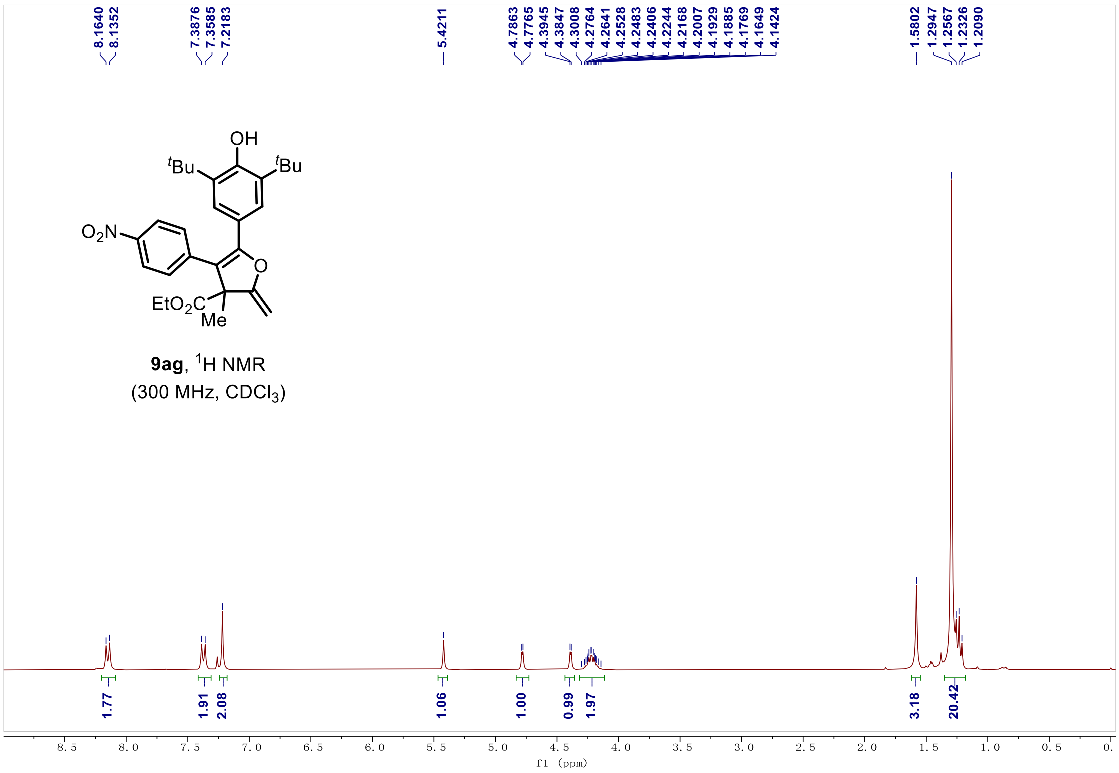
8.1640
8.1352
7.3876
7.3585
7.2183
5.4211
4.7863
4.7765
4.3945
4.3847
4.3008
4.2764
4.2641
4.2528
4.2483
4.2406
4.2244
4.2168
4.2007
4.1929
4.1885
4.1769
4.1649
4.1424
1.5802
1.2947
1.2567
1.2326
1.2090
OH
tBu
tBu
O2N
O
EtO2C
Me
9ag, 1H NMR
(300 MHz, CDCl3)
1.77
1.91
2.08
1.06
1.00
0.99
1.97
3.18
20.42
f1 (ppm)

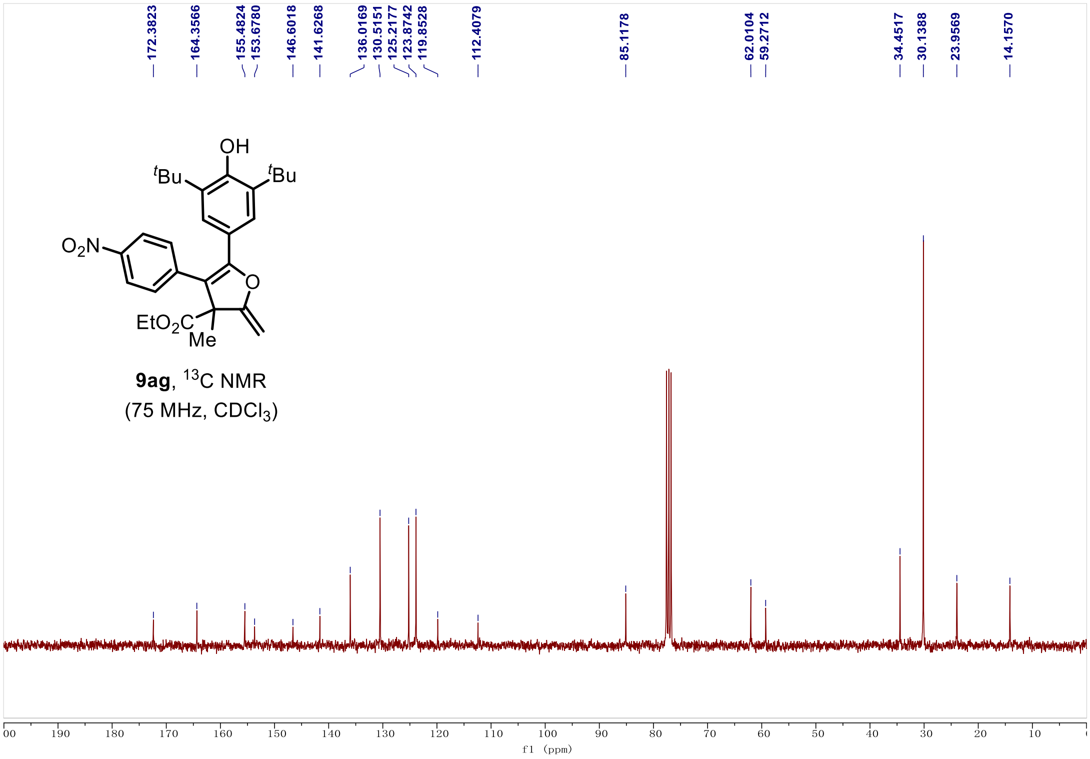
172.3823
164.3566
155.4824
153.6780
146.6018
141.6268
136.0169
130.5151
125.2177
123.8742
119.8528
112.4079
85.1178
62.0104
59.2712
34.4517
30.1388
23.9569
14.1570
OH
tBu
tBu
O2N
O
EtO2C
Me
9ag, 13C NMR
(75 MHz, CDCl3)
f1 (ppm)

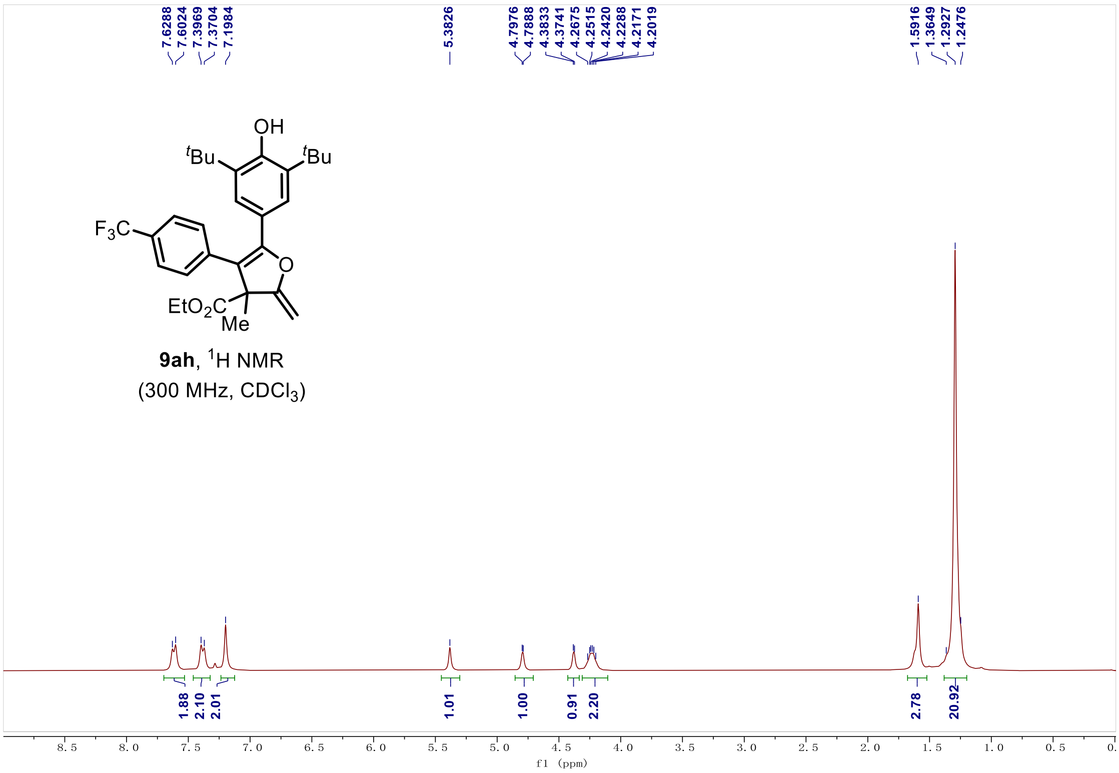

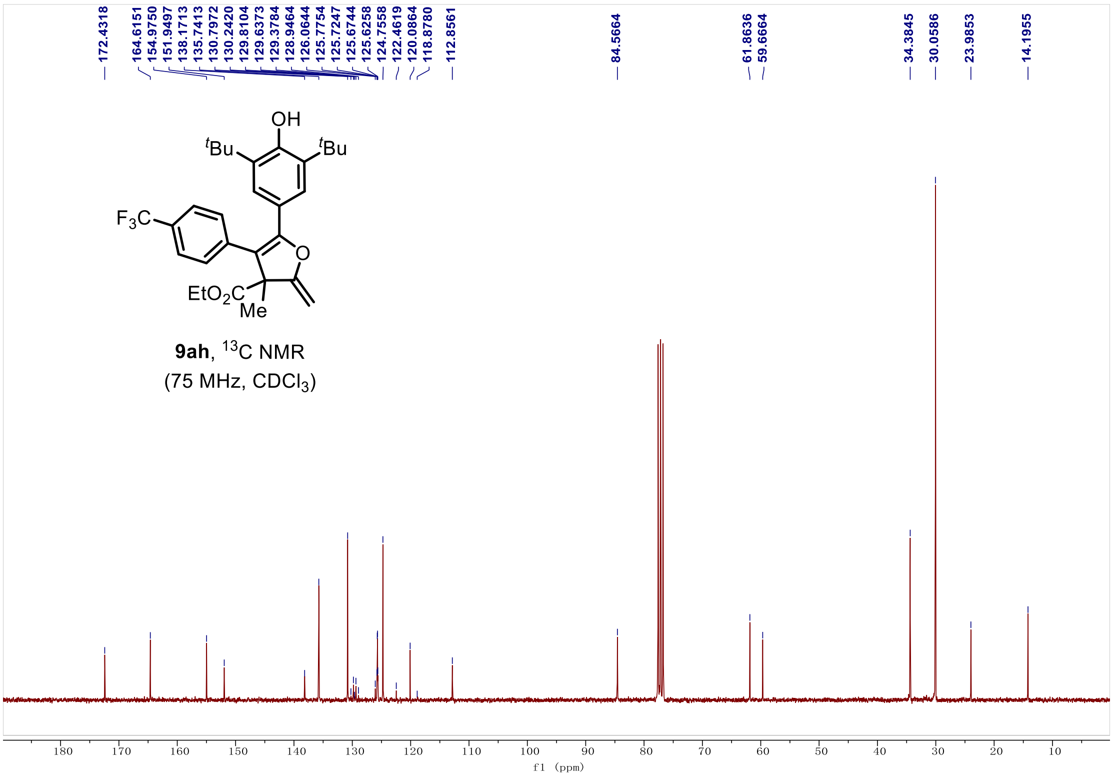

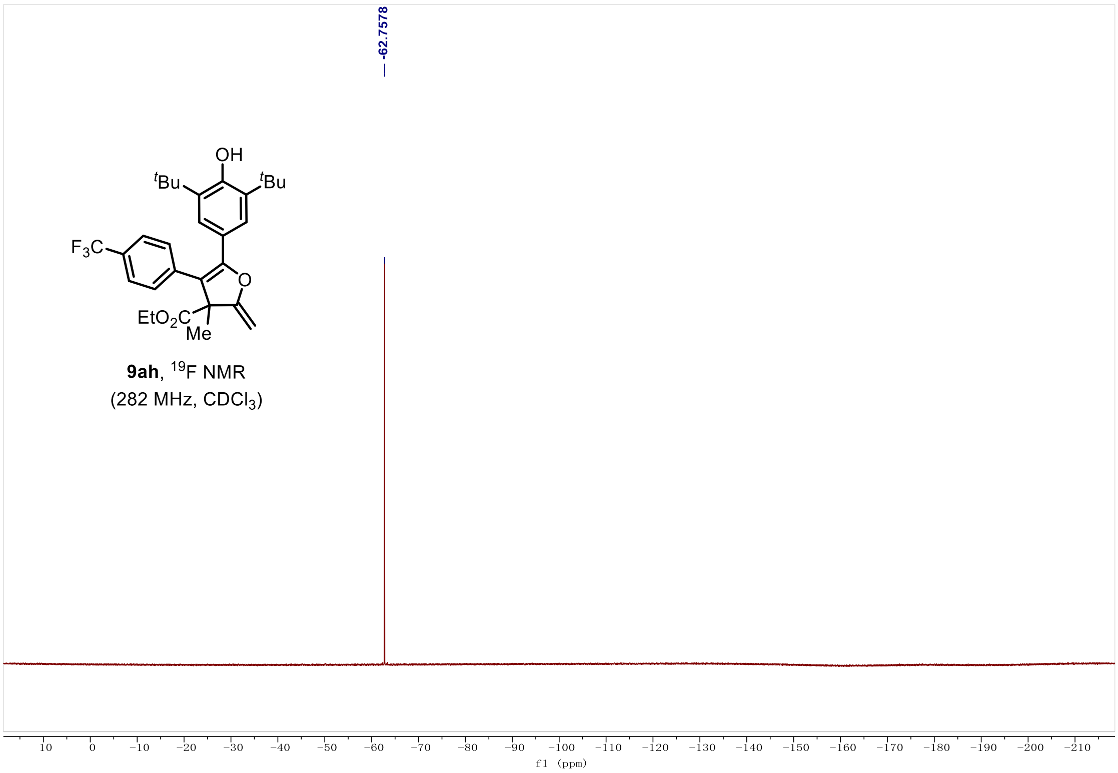
-62.7578
OH
tBu
tBu
F3C
O
EtO2C
Me
9ah, 19F NMR
(282 MHz, CDCl3)
f1 (ppm)

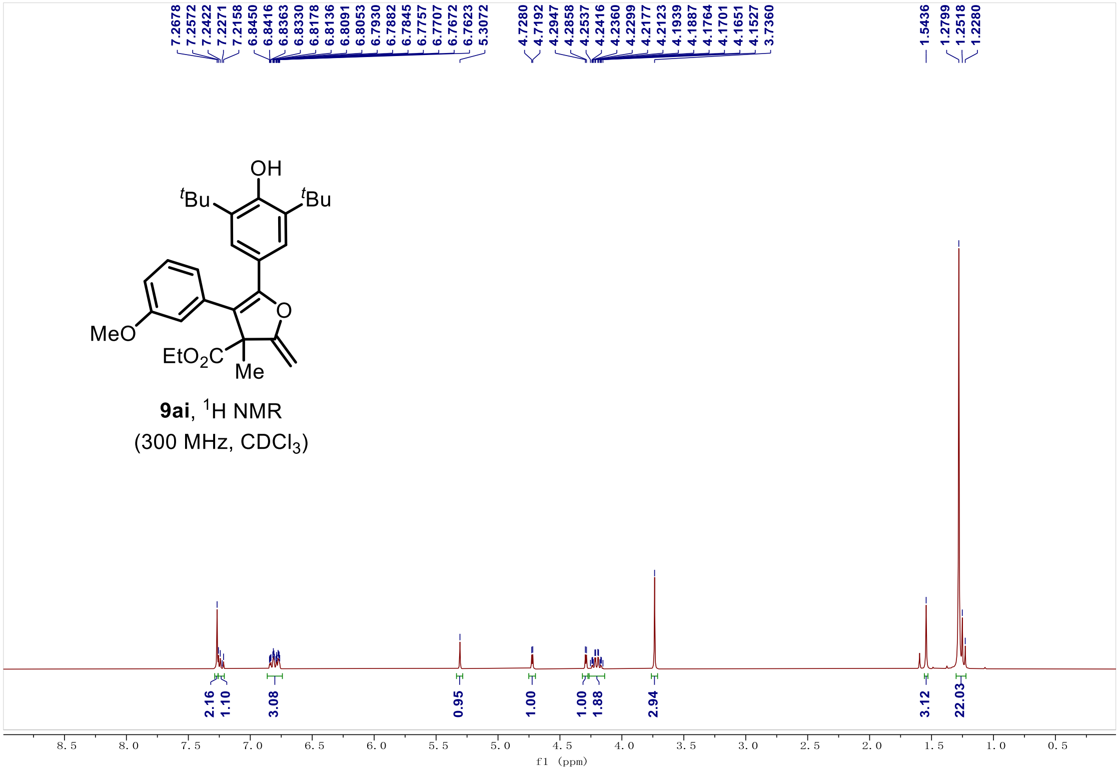
7.2678
7.2572
7.2422
7.2271
7.2158
6.8450
6.8416
6.8363
6.8330
6.8178
6.8136
6.8091
6.8053
6.7930
6.7882
6.7845
6.7757
6.7707
6.7672
6.7623
5.3072
4.7280
4.7192
4.2947
4.2858
4.2537
4.2416
4.2360
4.2299
4.2177
4.2123
4.1939
4.1887
4.1764
4.1701
4.1651
4.1527
3.7360
1.5436
1.2799
1.2518
1.2280
OH
tBu
tBu
MeO
O
EtO2C
Me
9ai, 1H NMR
(300 MHz, CDCl3)
2.16
1.10
3.08
0.95
1.00
1.00
1.88
2.94
3.12
22.03
f1 (ppm)

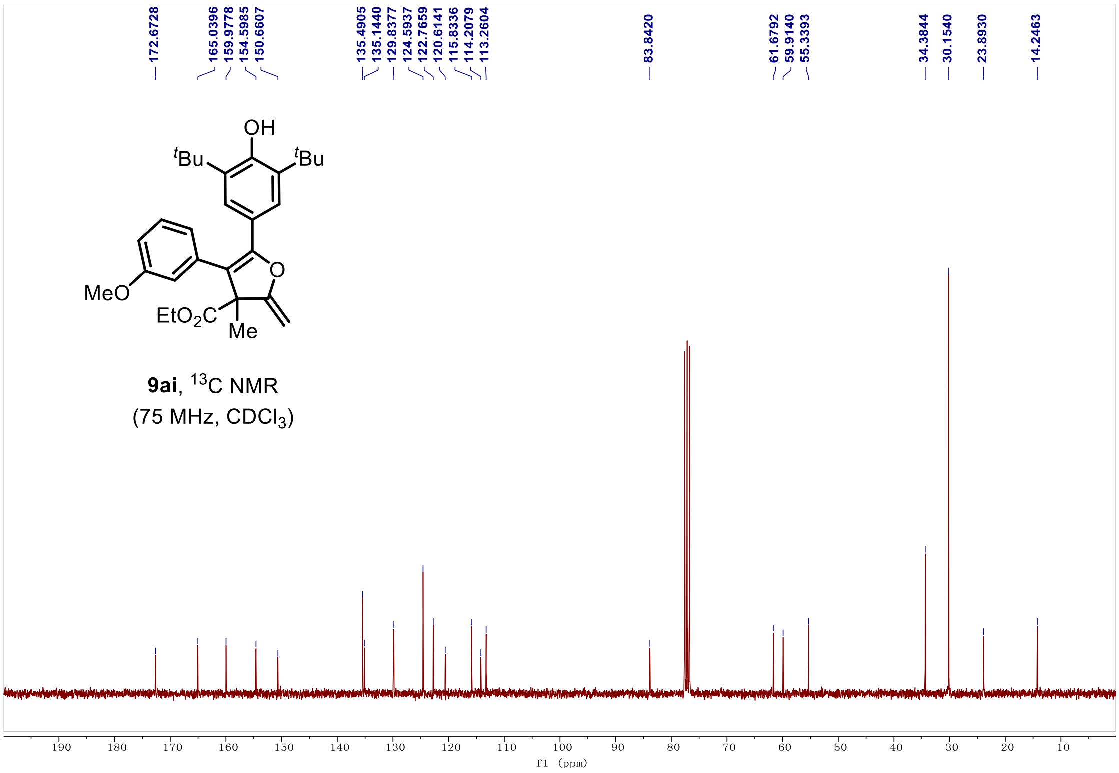
172.6728
165.0396
159.9778
154.5985
150.6607
135.4905
135.1440
129.8377
124.5937
122.7659
120.6141
115.8336
114.2079
113.2604
83.8420
61.6792
59.9140
55.3393
34.3844
30.1540
23.8930
14.2463
OH
tBu
tBu
MeO
O
EtO2C
Me
9ai, 13C NMR
(75 MHz, CDCl3)
f1 (ppm)

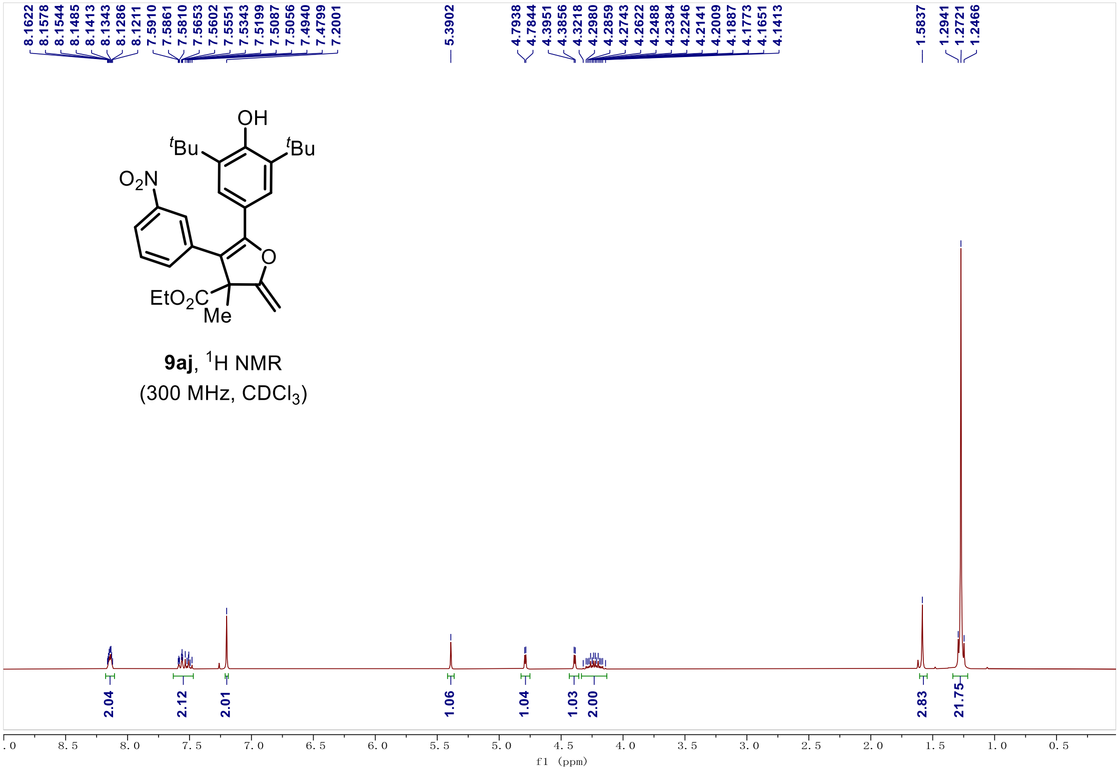
8.1622
8.1578
8.1544
8.1485
8.1413
8.1343
8.1286
8.1211
7.5910
7.5861
7.5810
7.5653
7.5602
7.5551
7.5343
7.5199
7.5087
7.5056
7.4940
7.4799
7.2001
5.3902
4.7938
4.7844
4.3951
4.3856
4.3218
4.2980
4.2859
4.2743
4.2622
4.2488
4.2384
4.2246
4.2141
4.2009
4.1887
4.1773
4.1651
4.1413
1.5837
1.2941
1.2721
1.2466
OH
tBu
tBu
O2N
O
EtO2C
Me
9aj, 1H NMR
(300 MHz, CDCl3)
2.04
2.12
2.01
1.06
1.04
1.03
2.00
2.83
21.75
f1 (ppm)

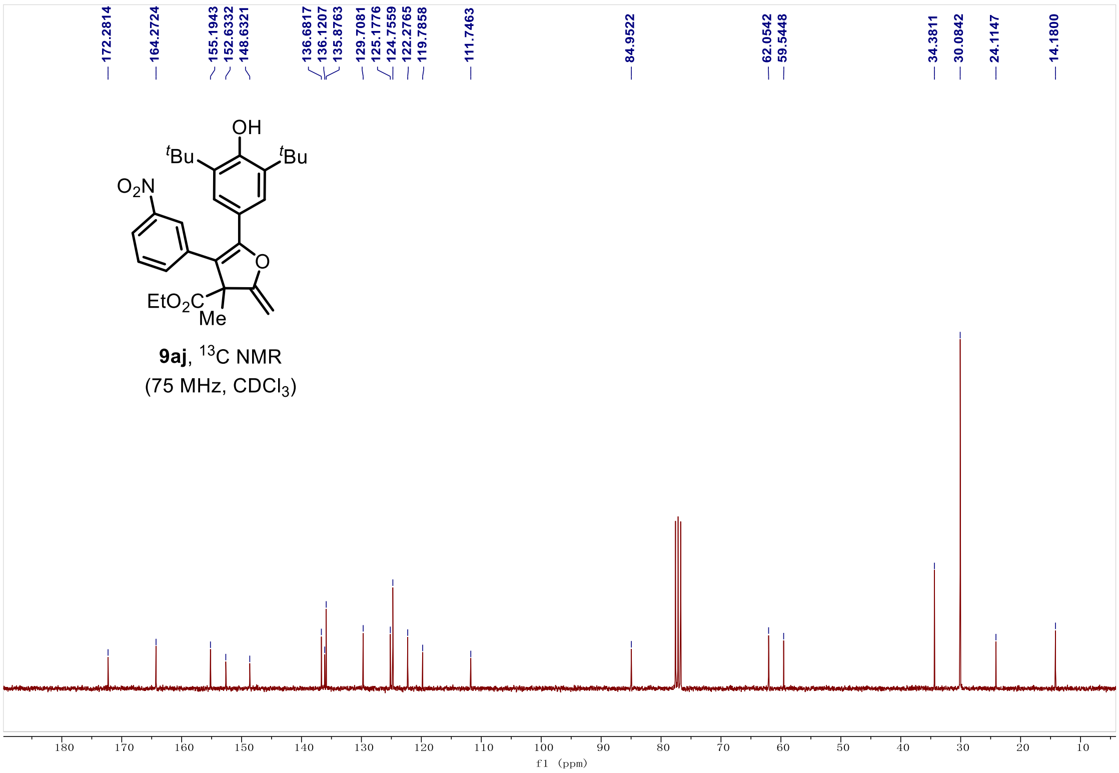
172.2814
164.2724
155.1943
152.6332
148.6321
136.6817
136.1207
135.8763
129.7081
125.1776
124.7559
122.2765
119.7858
111.7463
84.9522
62.0542
59.5448
34.3811
30.0842
24.1147
14.1800
OH
tBu
tBu
O2N
O
EtO2C
Me
9aj, 13C NMR
(75 MHz, CDCl3)
f1 (ppm)

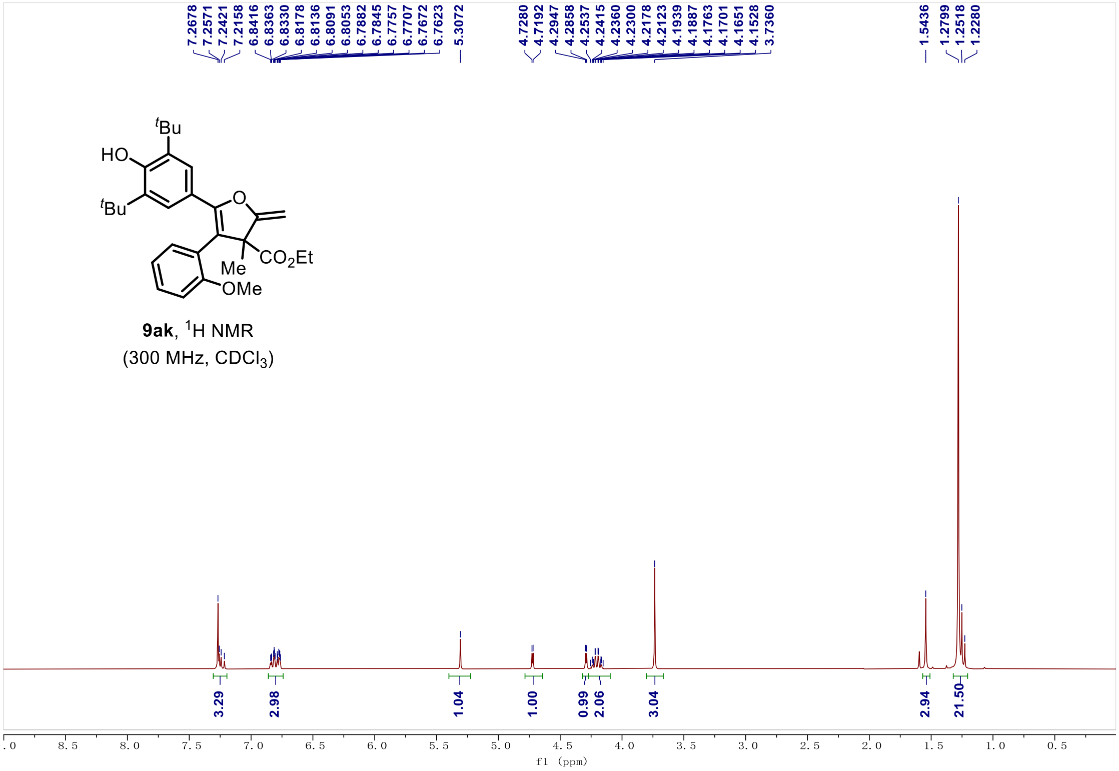
7.2678
7.2571
7.2421
7.2158
6.8416
6.8363
6.8330
6.8178
6.8136
6.8091
6.8053
6.7882
6.7845
6.7757
6.7707
6.7672
6.7623
5.3072
4.7280
4.7192
4.2947
4.2858
4.2537
4.2415
4.2360
4.2300
4.2178
4.2123
4.1939
4.1887
4.1763
4.1701
4.1651
4.1528
3.7360
1.5436
1.2799
1.2518
1.2280
tBu
HO
tBu
O
CO2Et
Me
OMe
9ak, 1H NMR
(300 MHz, CDCl3)
3.29
2.98
1.04
1.00
0.99
2.06
3.04
2.94
21.50
f1 (ppm)

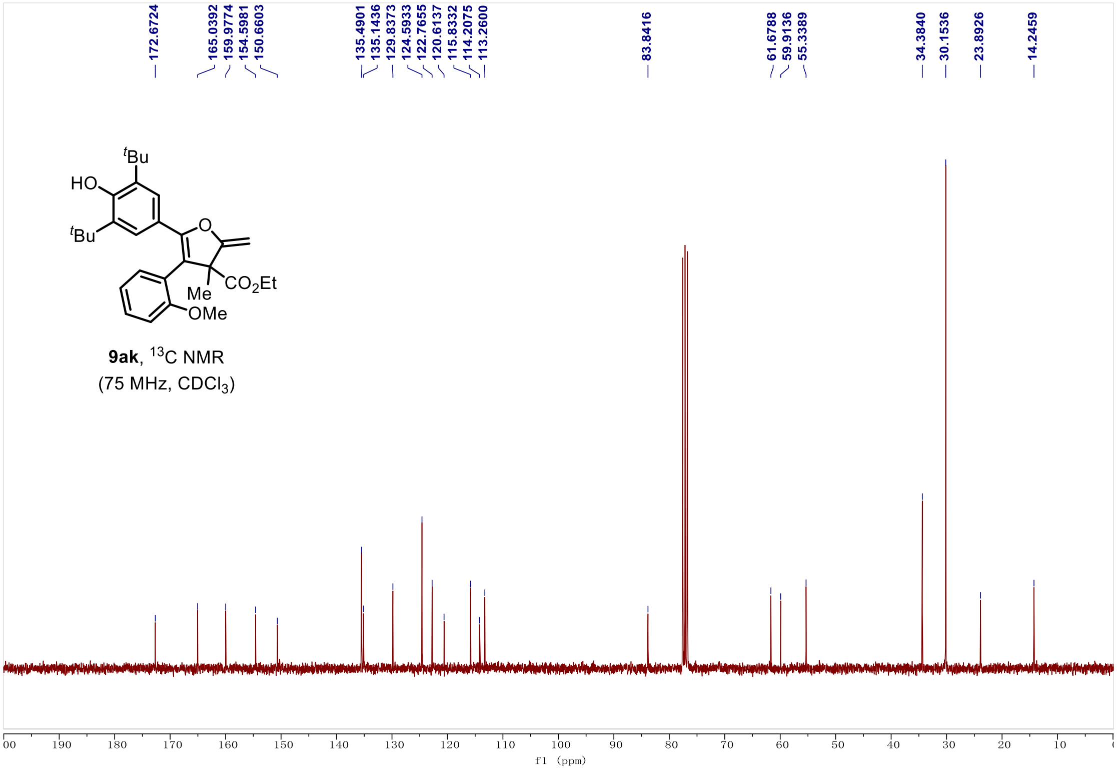
172.6724
165.0392
159.9774
154.5981
150.6603
135.4901
135.1436
129.8373
124.5933
122.7655
120.6137
115.8332
114.2075
113.2600
83.8416
61.6788
59.9136
55.3389
34.3840
30.1536
23.8926
14.2459
tBu
HO
tBu
O
CO2Et
Me
OMe
9ak, 13C NMR
(75 MHz, CDCl3)
f1 (ppm)

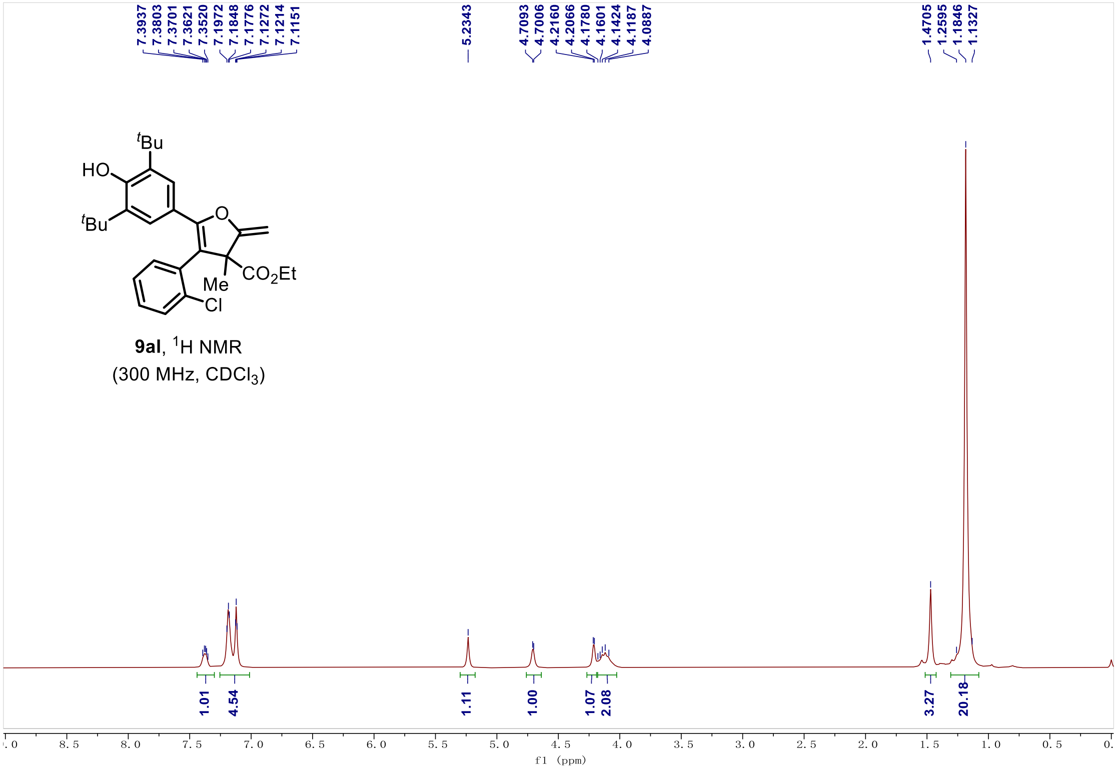
7.3937
7.3803
7.3701
7.3621
7.3520
7.1972
7.1848
7.1776
7.1272
7.1214
7.1151
5.2343
4.7093
4.7006
4.2160
4.2066
4.1780
4.1601
4.1424
4.1187
4.0887
1.4705
1.2595
1.1846
1.1327
tBu
HO
tBu
O
CO2Et
Me
Cl
9al, 1H NMR
(300 MHz, CDCl3)
1.01
4.54
1.11
1.00
1.07
2.08
3.27
20.18
f1 (ppm)

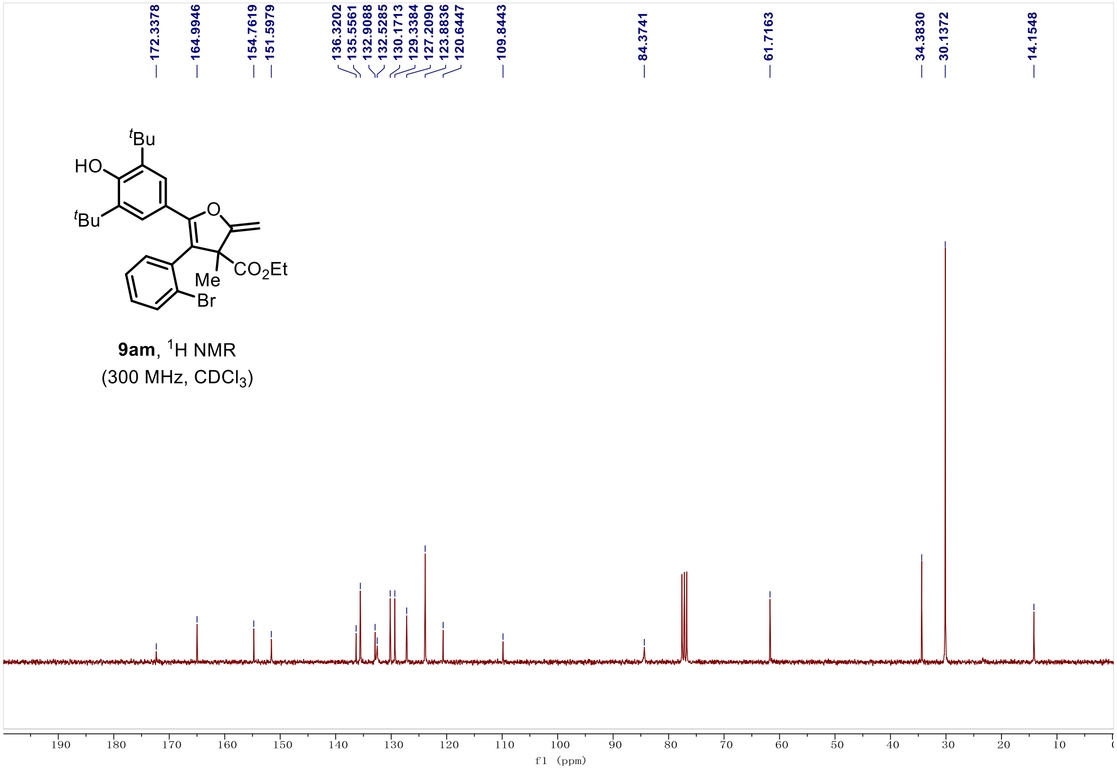
172.3378
164.9946
154.7619
151.5979
136.3202
135.5561
132.9088
132.5285
130.1713
129.3384
127.2090
123.8836
120.6447
109.8443
84.3741
61.7163
34.3830
30.1372
14.1548
tBu
HO
tBu
O
CO2Et
Me
Br
9am, 1H NMR
(300 MHz, CDCl3)
190 180 170 160 150 140 130 120 110 100 90 80 70 60 50 40 30 20 10
f1 (ppm)

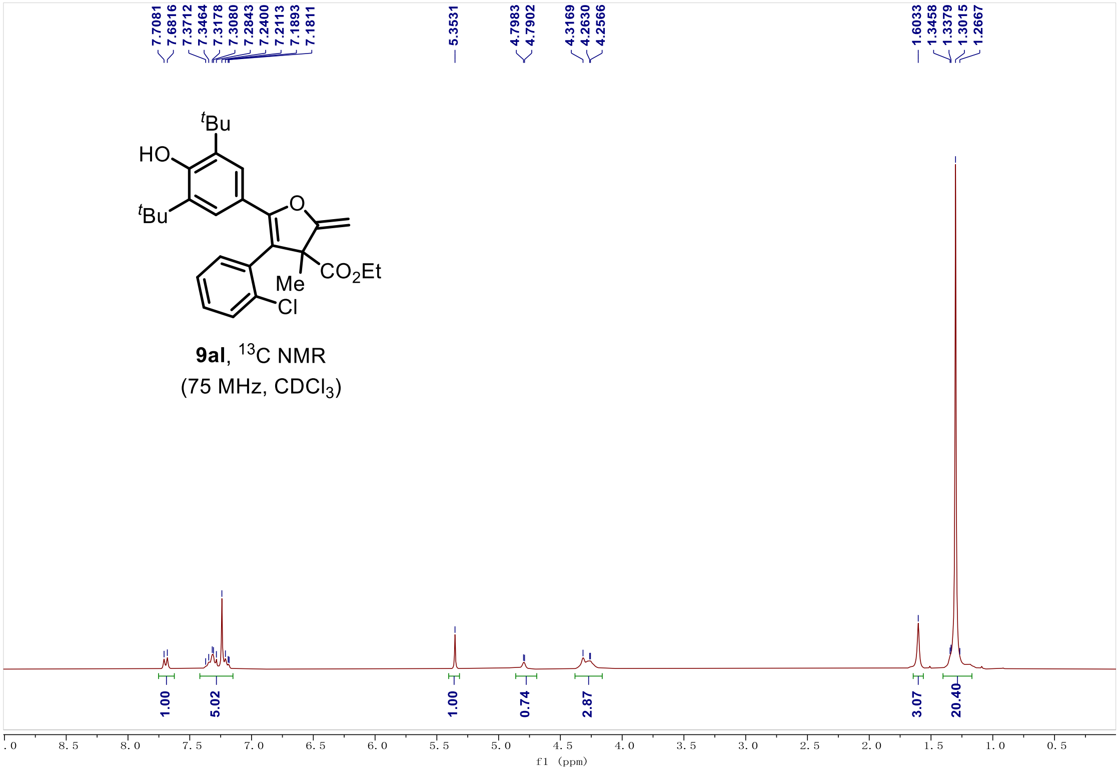
7.7081
7.6816
7.3712
7.3464
7.3178
7.3080
7.2843
7.2400
7.2113
7.1893
7.1811
5.3531
4.7983
4.7902
4.3169
4.2630
4.2566
1.6033
1.3458
1.3379
1.3015
1.2667
tBu
HO
tBu
O
CO2Et
Me
Cl
9al, 13C NMR
(75 MHz, CDCl3)
1.00
5.02
1.00
0.74
2.87
3.07
20.40
8.5 8.0 7.5 7.0 6.5 6.0 5.5 5.0 4.5 4.0 3.5 3.0 2.5 2.0 1.5 1.0 0.5
f1 (ppm)

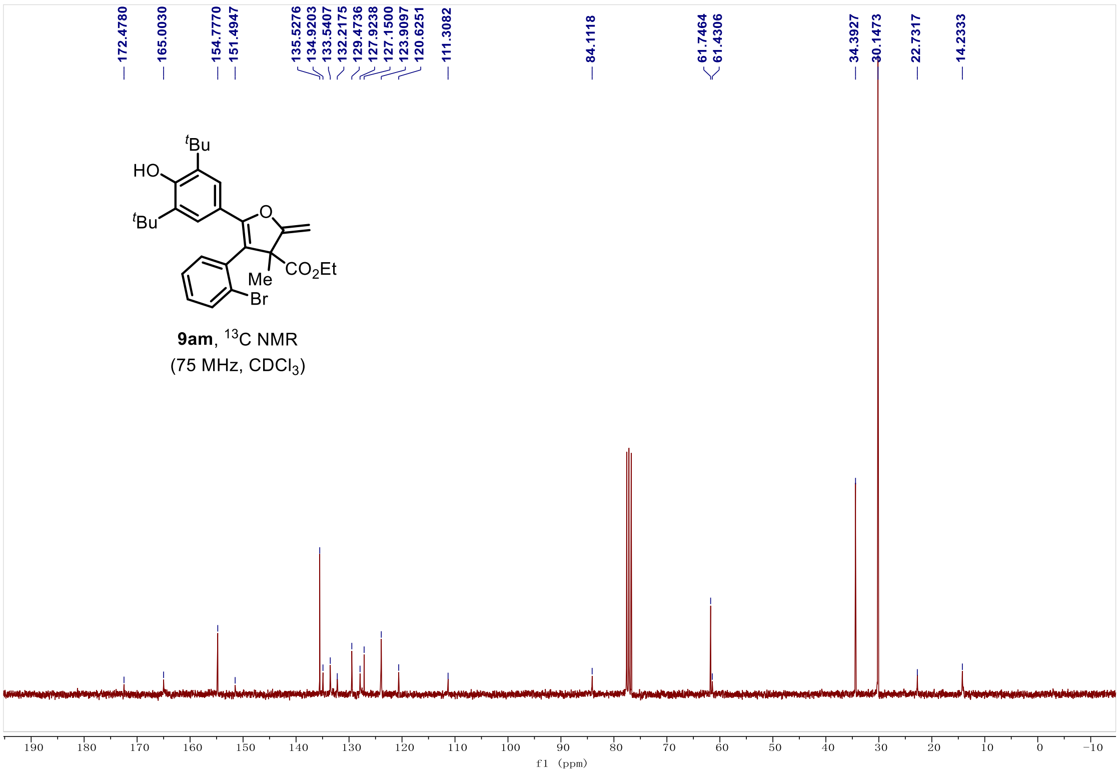
172.4780
165.0030
154.7770
151.4947
135.5276
134.9203
133.5407
132.2175
129.4736
127.9238
127.1500
123.9097
120.6251
111.3082
84.1118
61.7464
61.4306
34.3927
30.1473
22.7317
14.2333
HO
tBu
O
CO2Et
Me
Br
9am, 13C NMR
(75 MHz, CDCl3)
f1 (ppm)

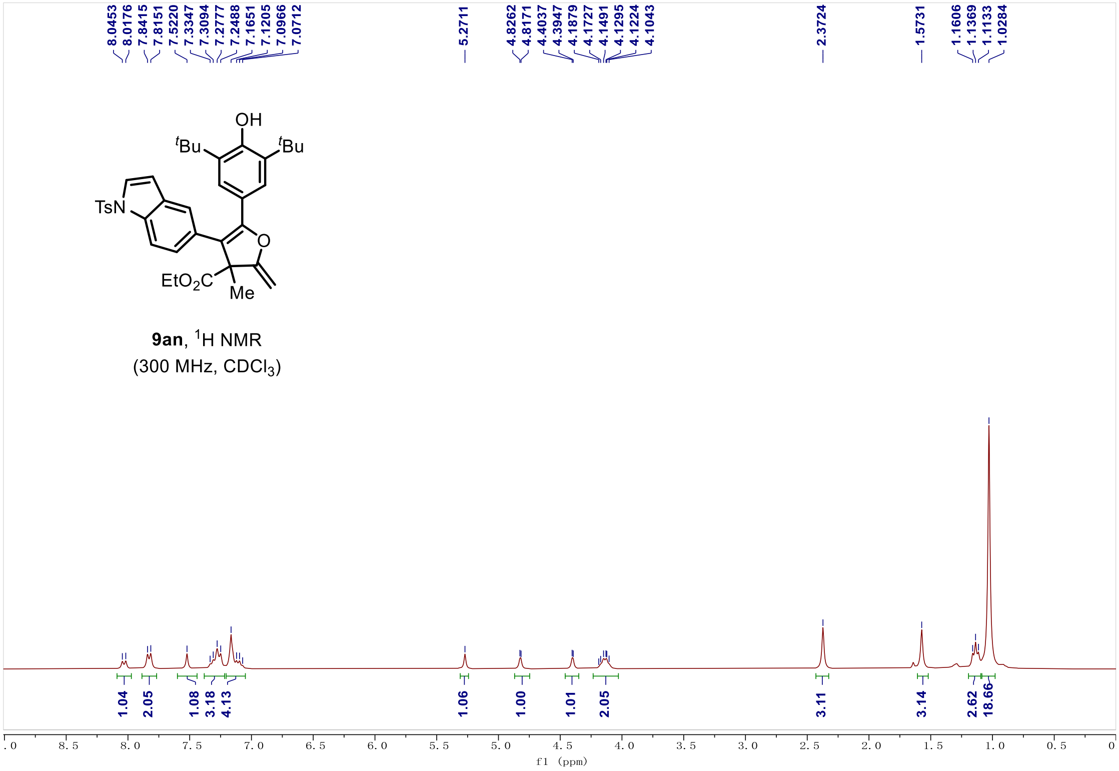
8.0453
8.0176
7.8415
7.8151
7.5220
7.3347
7.3094
7.2777
7.2488
7.1651
7.1205
7.0966
7.0712
5.2711
4.8262
4.8171
4.4037
4.3947
4.1879
4.1727
4.1491
4.1295
4.1224
4.1043
2.3724
1.5731
1.1606
1.1369
1.1133
1.0284
OH
tBu
TsN
O
EtO2C
Me
9an, 1H NMR
(300 MHz, CDCl3)
1.04
2.05
1.08
3.18
4.13
1.06
1.00
1.01
2.05
3.11
3.14
2.62
18.66
f1 (ppm)

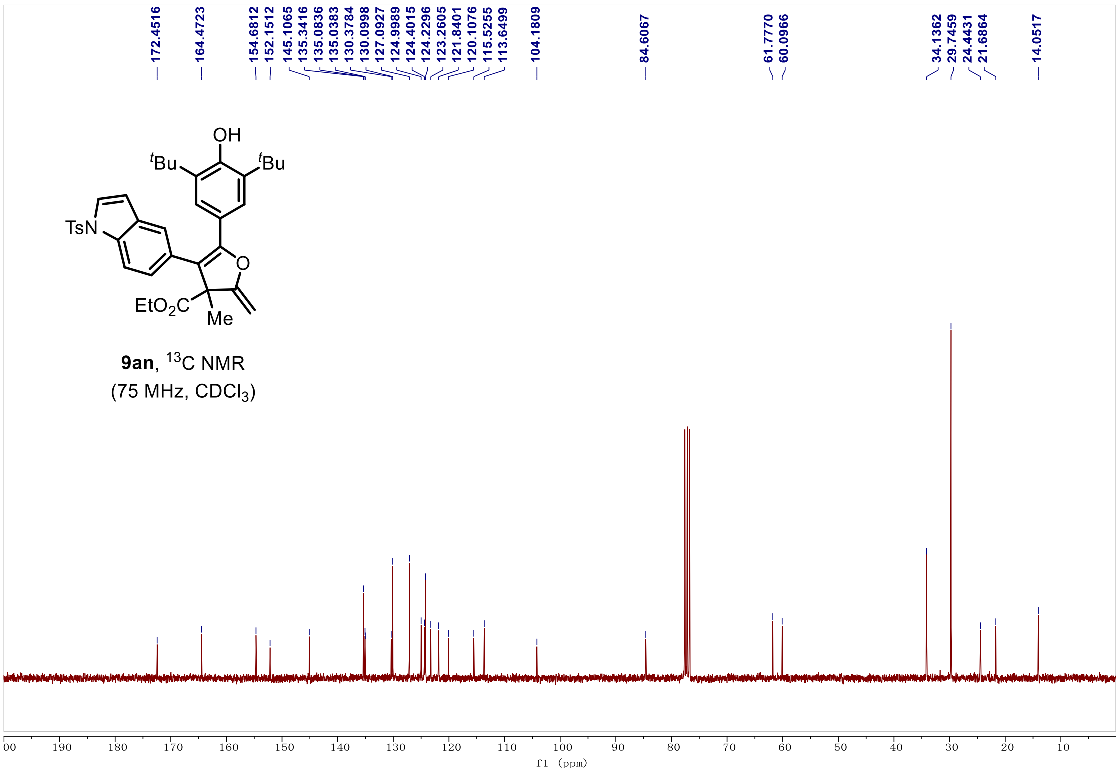

9an, ^{13}C NMR
(75 MHz, $CDCl_3$)

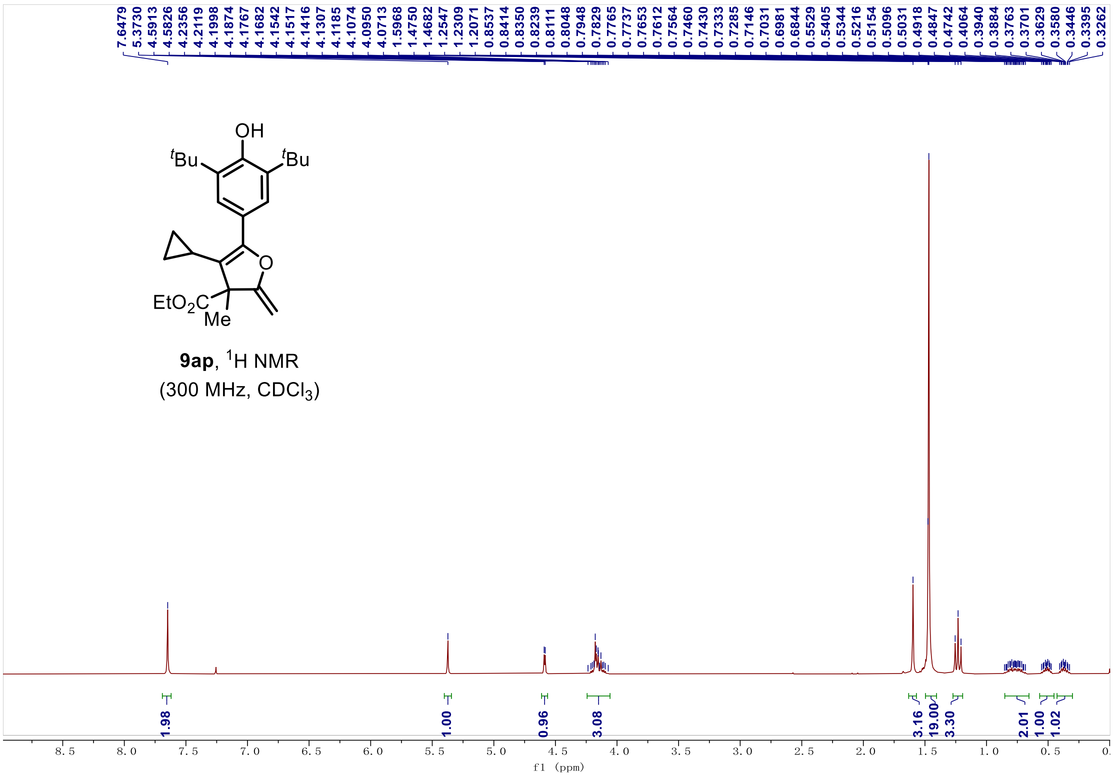

9ap, ^{1}H NMR
(300 MHz, $CDCl_3$)

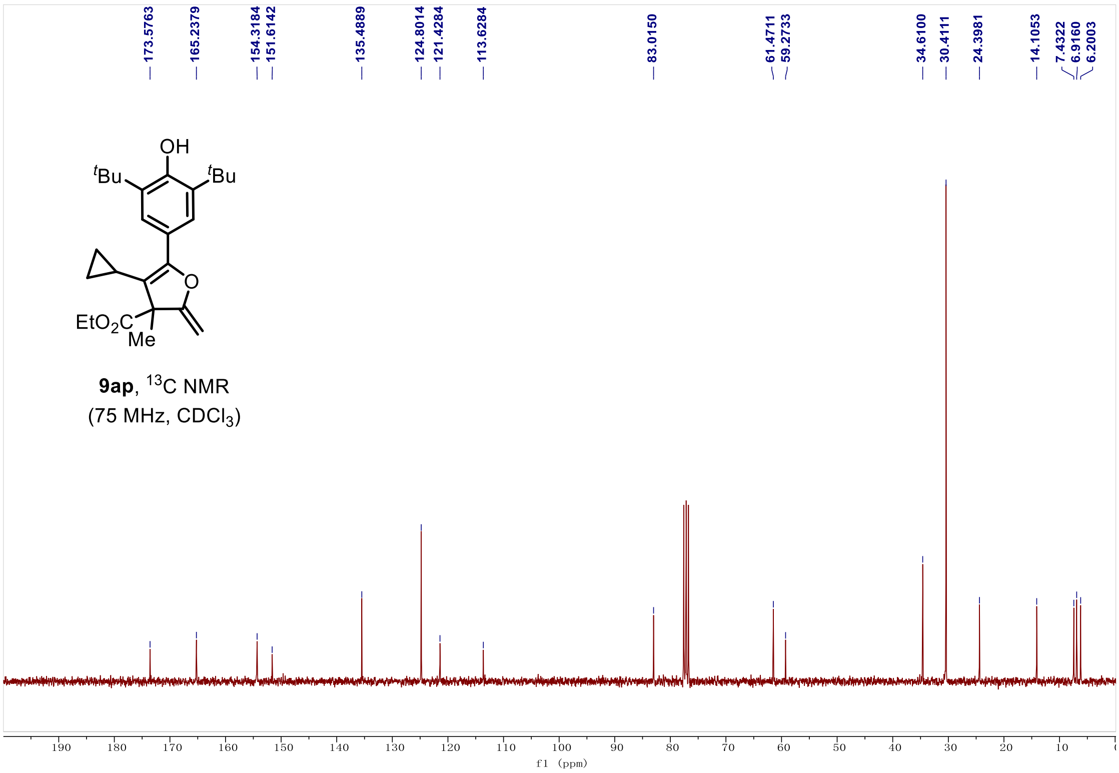
173.5763
165.2379
154.3184
151.6142
135.4889
124.8014
121.4284
113.6284
83.0150
61.4711
59.2733
34.6100
30.4111
24.3981
14.1053
7.4322
6.9160
6.2003
OH
tBu
tBu
O
EtO2C
Me
9ap, 13C NMR
(75 MHz, CDCl3)
f1 (ppm)

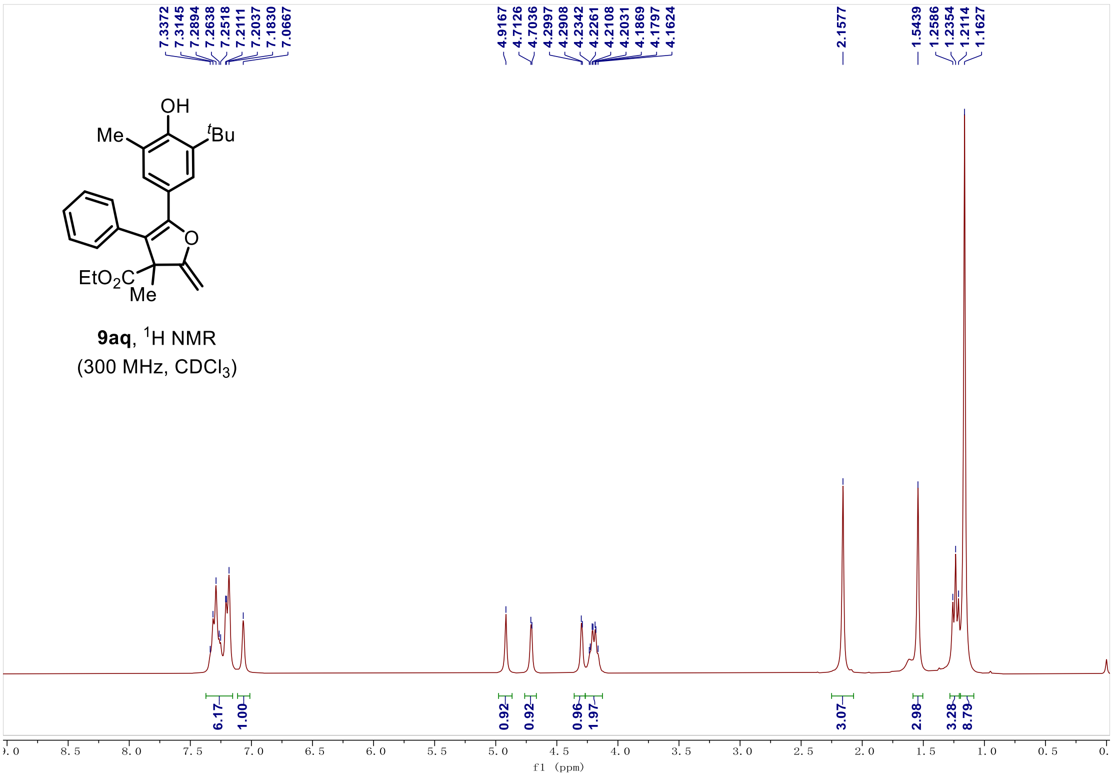
7.3372
7.3145
7.2894
7.2638
7.2518
7.2111
7.2037
7.1830
7.0667
4.9167
4.7126
4.7036
4.2997
4.2908
4.2342
4.2261
4.2108
4.2031
4.1869
4.1797
4.1624
2.1577
1.5439
1.2586
1.2354
1.2114
1.1627
OH
Me
tBu
O
EtO2C
Me
9aq, 1H NMR
(300 MHz, CDCl3)
6.17
1.00
0.92
0.92
0.96
1.97
3.07
2.98
3.28
8.79
f1 (ppm)

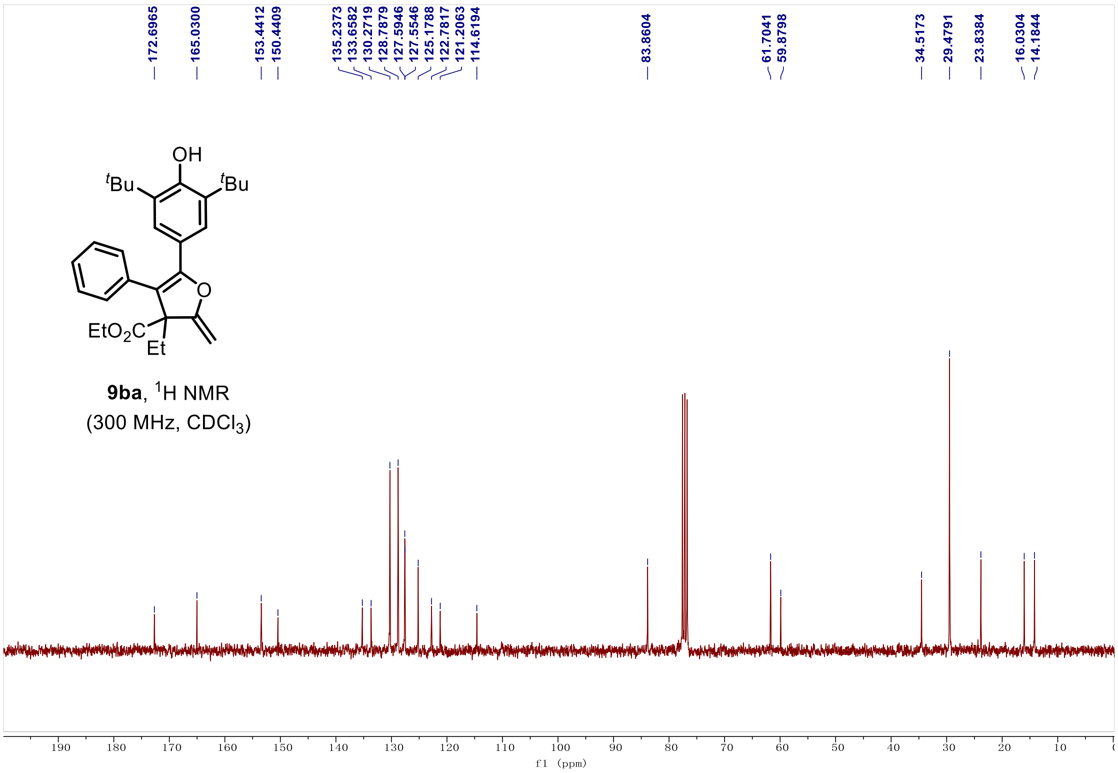
172.6965
165.0300
153.4412
150.4409
135.2373
133.6582
130.2719
128.7879
127.5946
127.5546
125.1788
122.7817
121.2063
114.6194
83.8604
61.7041
59.8798
34.5173
29.4791
23.8384
16.0304
14.1844
OH
^{t}Bu
^{t}Bu
O
EtO_2C
Et
9ba, ^{1}H NMR
(300 MHz, $CDCl_3$)
190 180 170 160 150 140 130 120 110 100 90 80 70 60 50 40 30 20 10
f1 (ppm)

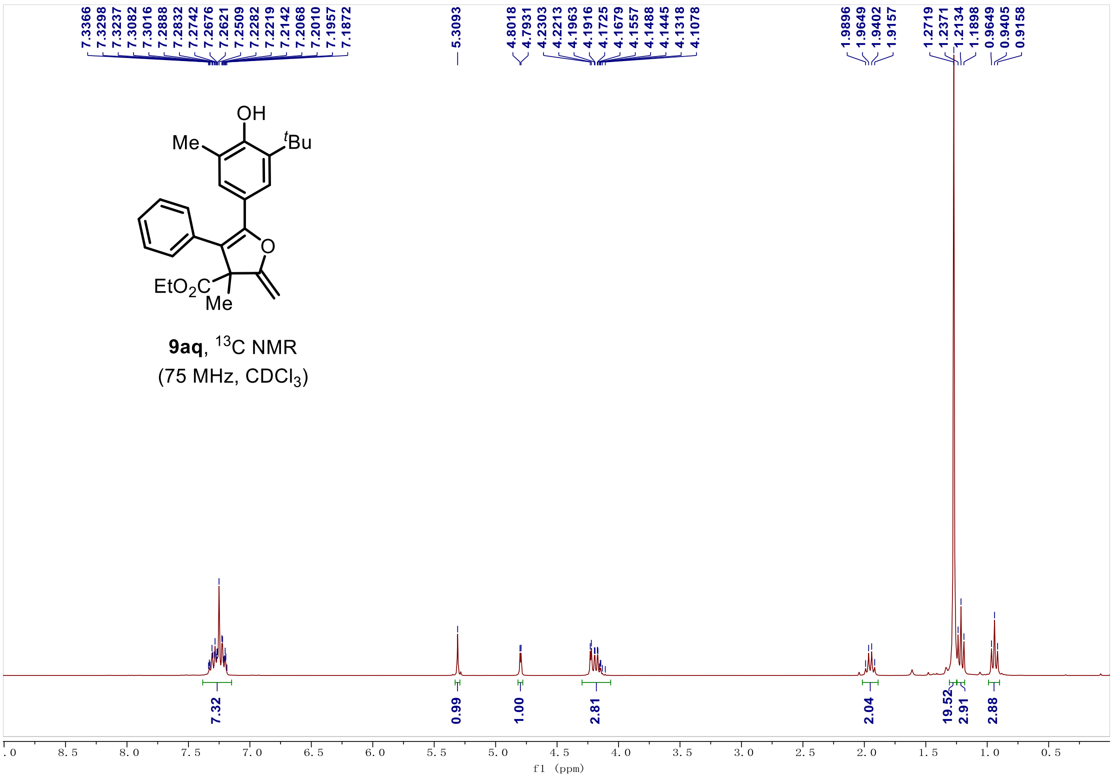
7.3366
7.3298
7.3237
7.3082
7.3016
7.2888
7.2832
7.2742
7.2676
7.2621
7.2509
7.2282
7.2219
7.2142
7.2068
7.2010
7.1957
7.1872
5.3093
4.8018
4.7931
4.2303
4.2213
4.1963
4.1916
4.1725
4.1679
4.1557
4.1488
4.1445
4.1318
4.1078
1.9896
1.9649
1.9402
1.9157
1.2719
1.2371
1.2134
1.1898
0.9649
0.9405
0.9158
OH
Me
^{t}Bu
O
EtO_2C
Me
9aq, ^{13}C NMR
(75 MHz, $CDCl_3$)
7.32
0.99
1.00
2.81
2.04
19.52
2.91
2.88
8.5 8.0 7.5 7.0 6.5 6.0 5.5 5.0 4.5 4.0 3.5 3.0 2.5 2.0 1.5 1.0 0.5
f1 (ppm)

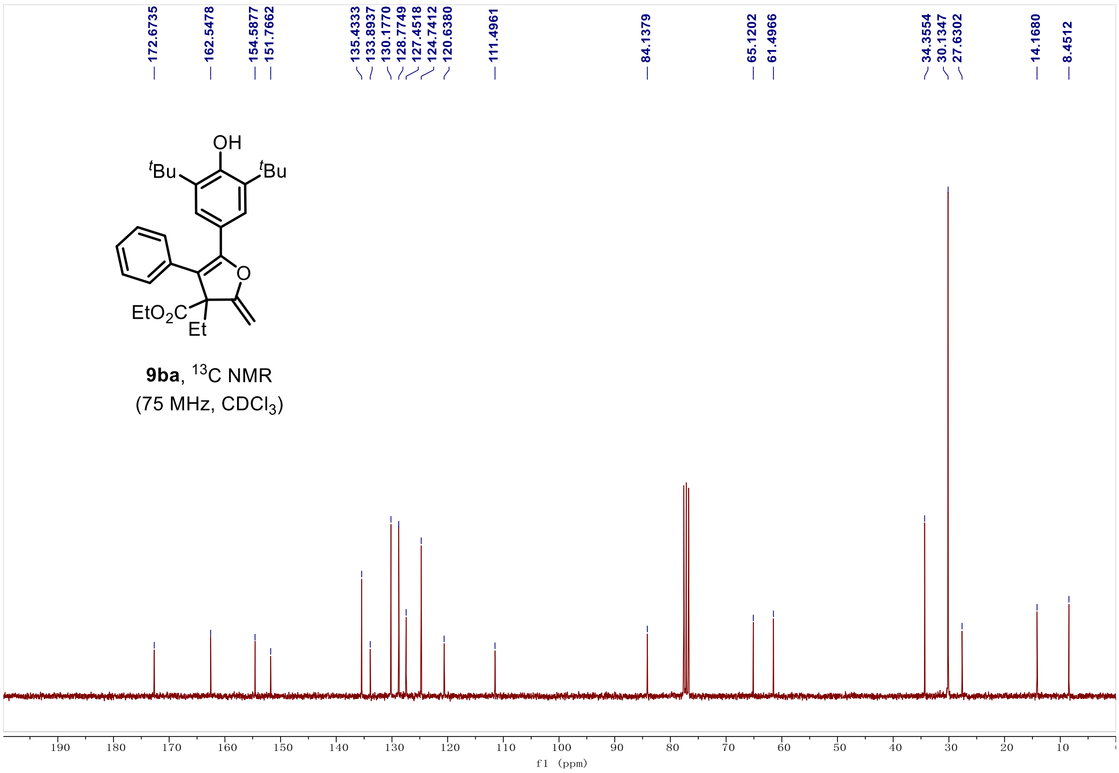
172.6735
162.5478
154.5877
151.7662
135.4333
133.8937
130.1770
128.7749
127.4518
124.7412
120.6380
111.4961
84.1379
65.1202
61.4966
34.3554
30.1347
27.6302
14.1680
8.4512
OH
tBu
tBu
O
EtO2C
Et
9ba, 13C NMR
(75 MHz, CDCl3)
f1 (ppm)

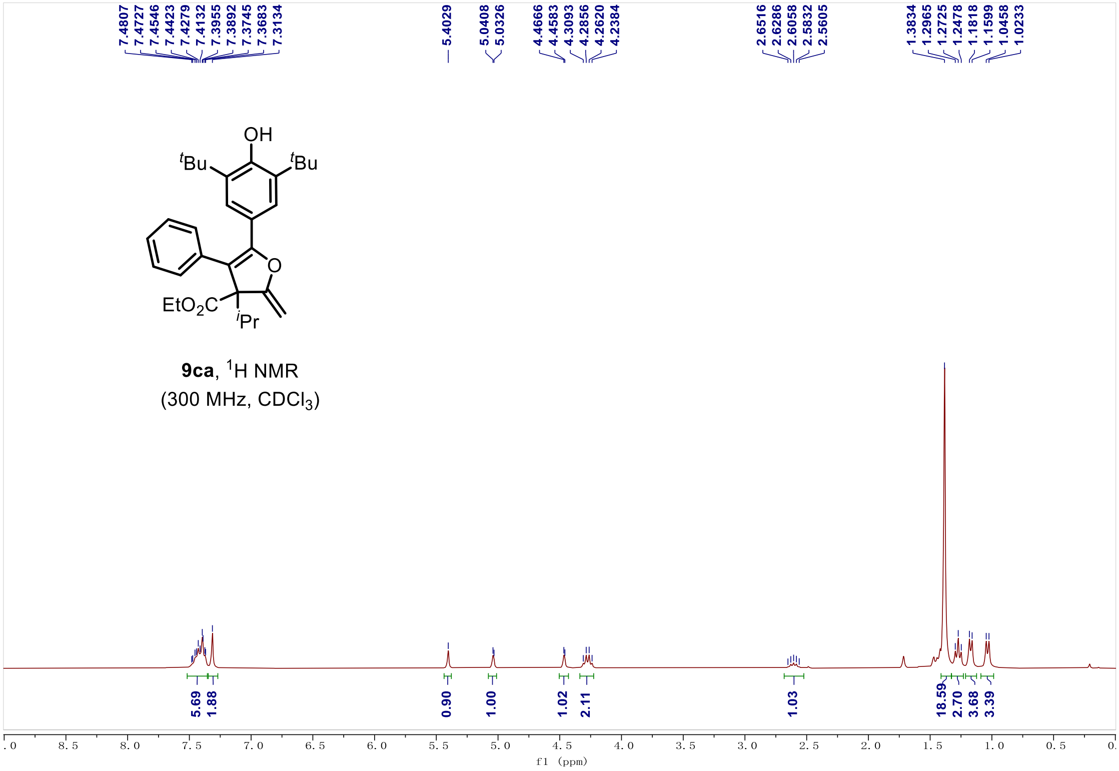
7.4807
7.4727
7.4546
7.4423
7.4279
7.4132
7.3955
7.3892
7.3745
7.3683
7.3134
5.4029
5.0408
5.0326
4.4666
4.4583
4.3093
4.2856
4.2620
4.2384
2.6516
2.6286
2.6058
2.5832
2.5605
1.3834
1.2965
1.2725
1.2478
1.1818
1.1599
1.0458
1.0233
OH
tBu
tBu
O
EtO2C
iPr
9ca, 1H NMR
(300 MHz, CDCl3)
5.69
1.88
0.90
1.00
1.02
2.11
1.03
18.59
2.70
3.68
3.39
f1 (ppm)

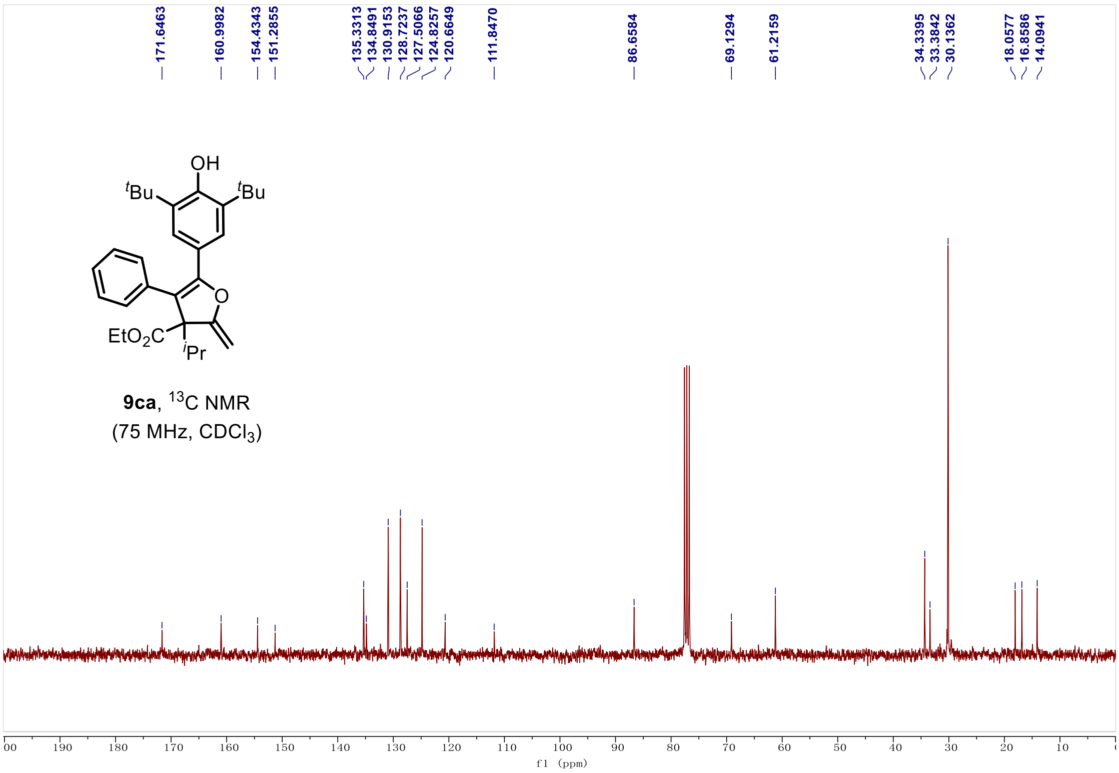
171.6463
160.9982
154.4343
151.2855
135.3313
134.8491
130.9153
128.7237
127.5066
124.8257
120.6649
111.8470
86.6584
69.1294
61.2159
34.3395
33.3842
30.1362
18.0577
16.8586
14.0941
OH
tBu
tBu
O
EtO2C
iPr
9ca, 13C NMR
(75 MHz, CDCl3)
f1 (ppm)

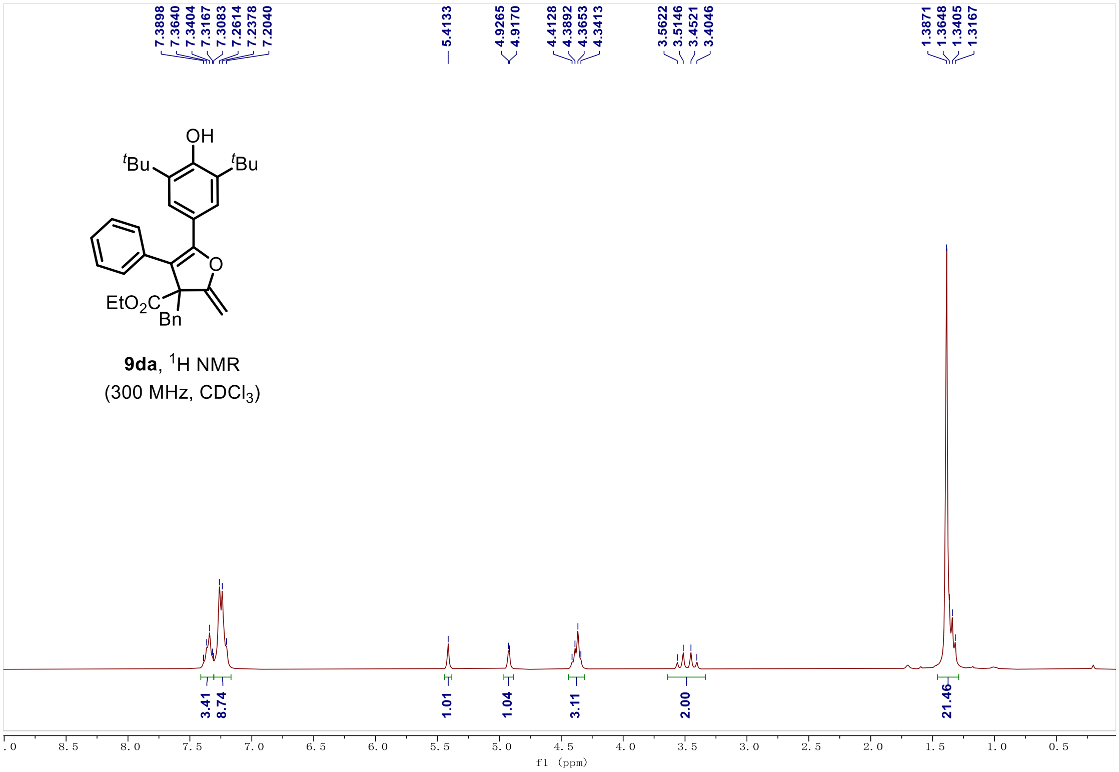
7.3898
7.3640
7.3404
7.3167
7.3083
7.2614
7.2378
7.2040
5.4133
4.9265
4.9170
4.4128
4.3892
4.3653
4.3413
3.5622
3.5146
3.4521
3.4046
1.3871
1.3648
1.3405
1.3167
OH
tBu
tBu
O
EtO2C
Bn
9da, 1H NMR
(300 MHz, CDCl3)
3.41
8.74
1.01
1.04
3.11
2.00
21.46
f1 (ppm)

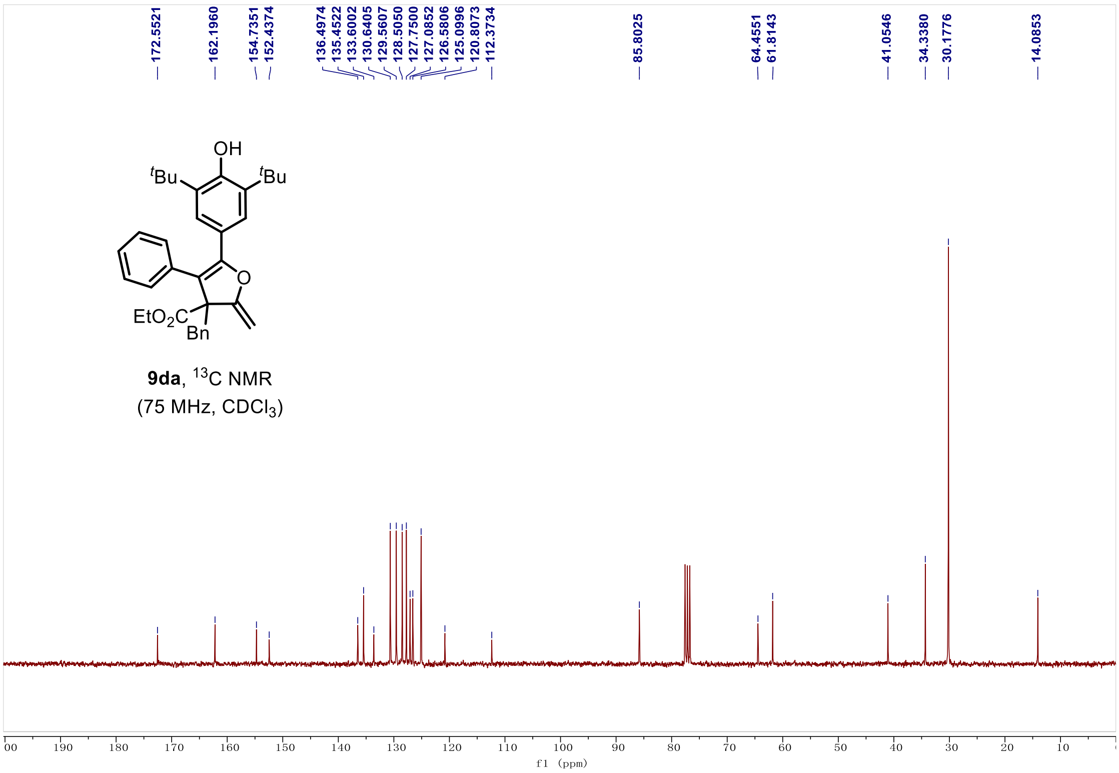

9da, ^{13}C NMR
(75 MHz, $CDCl_3$)

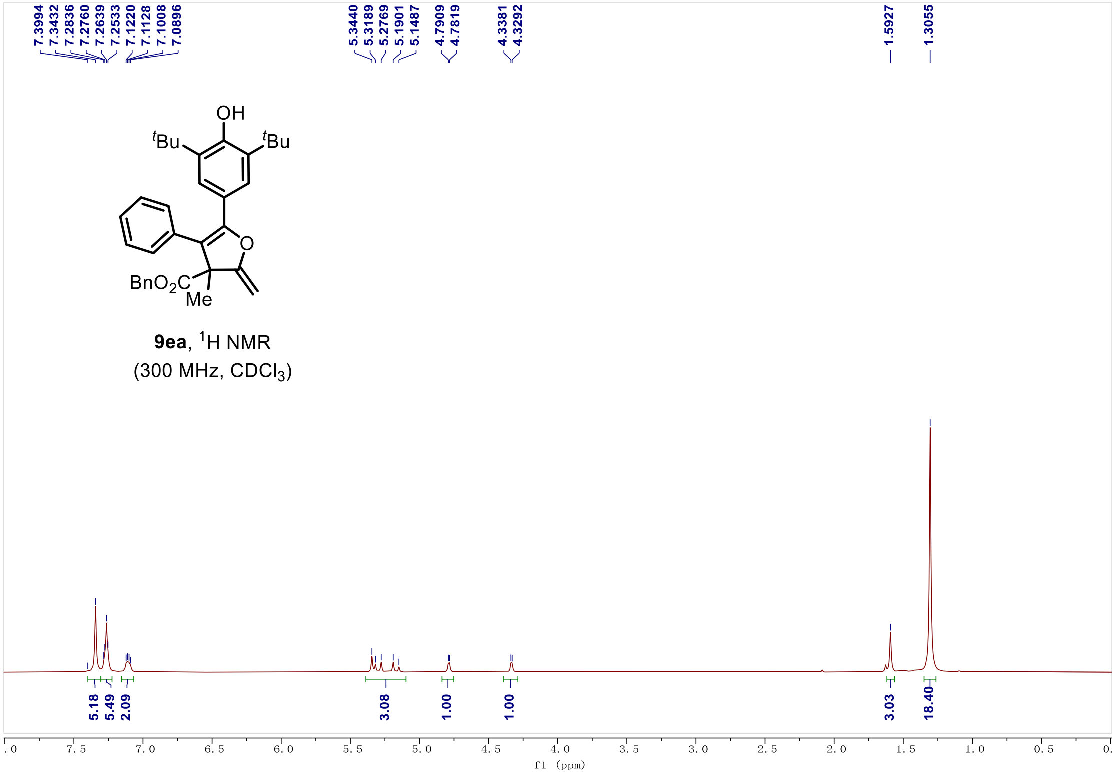

9ea, ^{1}H NMR
(300 MHz, $CDCl_3$)

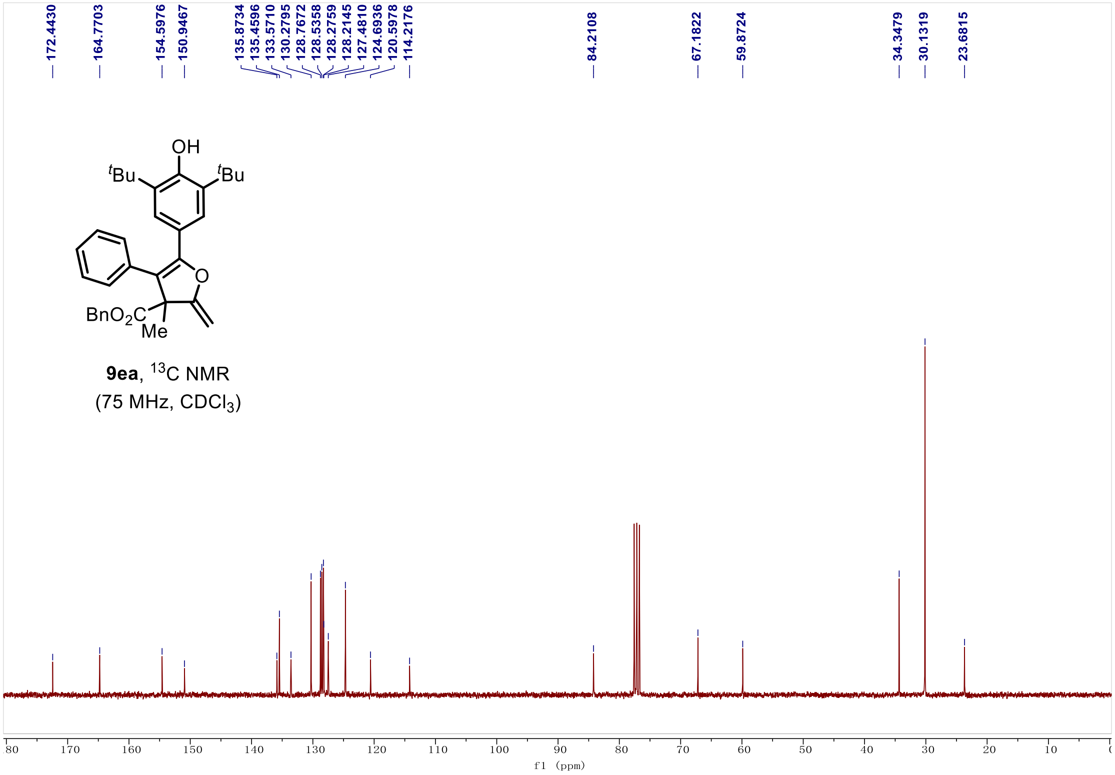
172.4430
164.7703
154.5976
150.9467
135.8734
135.4596
133.5710
130.2795
128.7672
128.5358
128.2759
128.2145
127.4810
124.6936
120.5978
114.2176
84.2108
67.1822
59.8724
34.3479
30.1319
23.6815
OH
tBu
tBu
O
BnO2C
Me
9ea, 13C NMR
(75 MHz, CDCl3)
f1 (ppm)

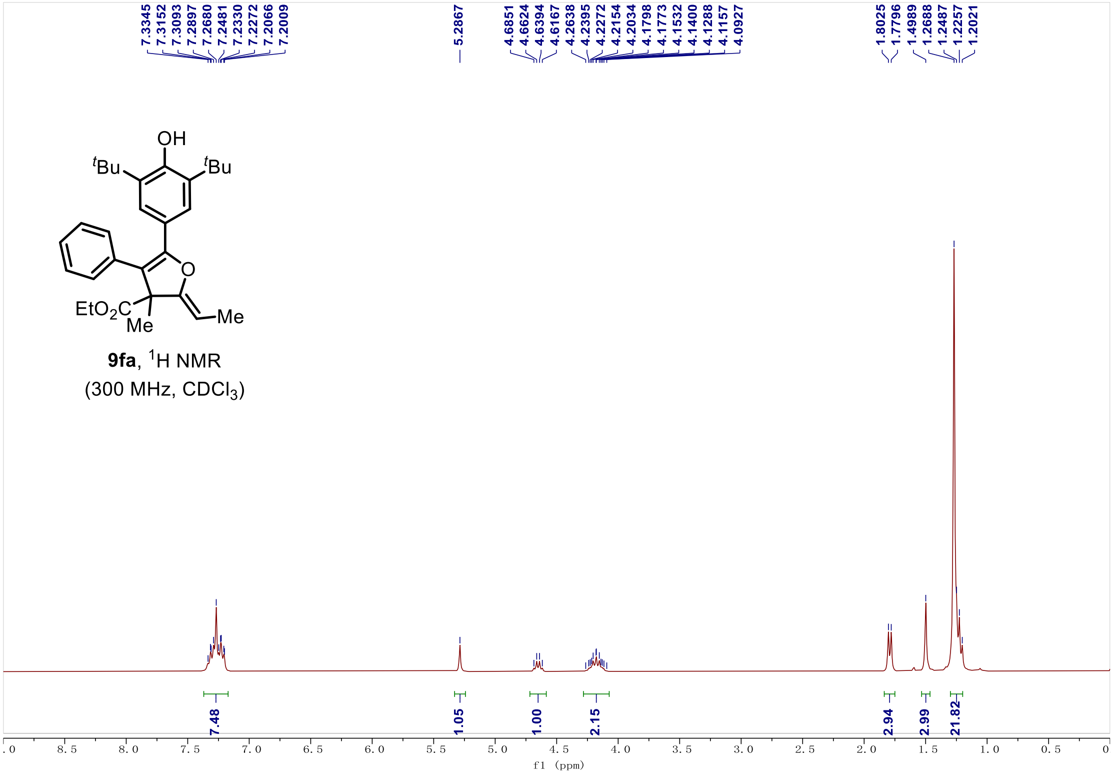
7.3345
7.3152
7.3093
7.2897
7.2680
7.2481
7.2330
7.2272
7.2066
7.2009
5.2867
4.6851
4.6624
4.6394
4.6167
4.2638
4.2395
4.2272
4.2154
4.2034
4.1798
4.1773
4.1532
4.1400
4.1288
4.1157
4.0927
1.8025
1.7796
1.4989
1.2688
1.2487
1.2257
1.2021
OH
tBu
tBu
O
EtO2C
Me
Me
9fa, 1H NMR
(300 MHz, CDCl3)
7.48
1.05
1.00
2.15
2.94
2.99
21.82
f1 (ppm)

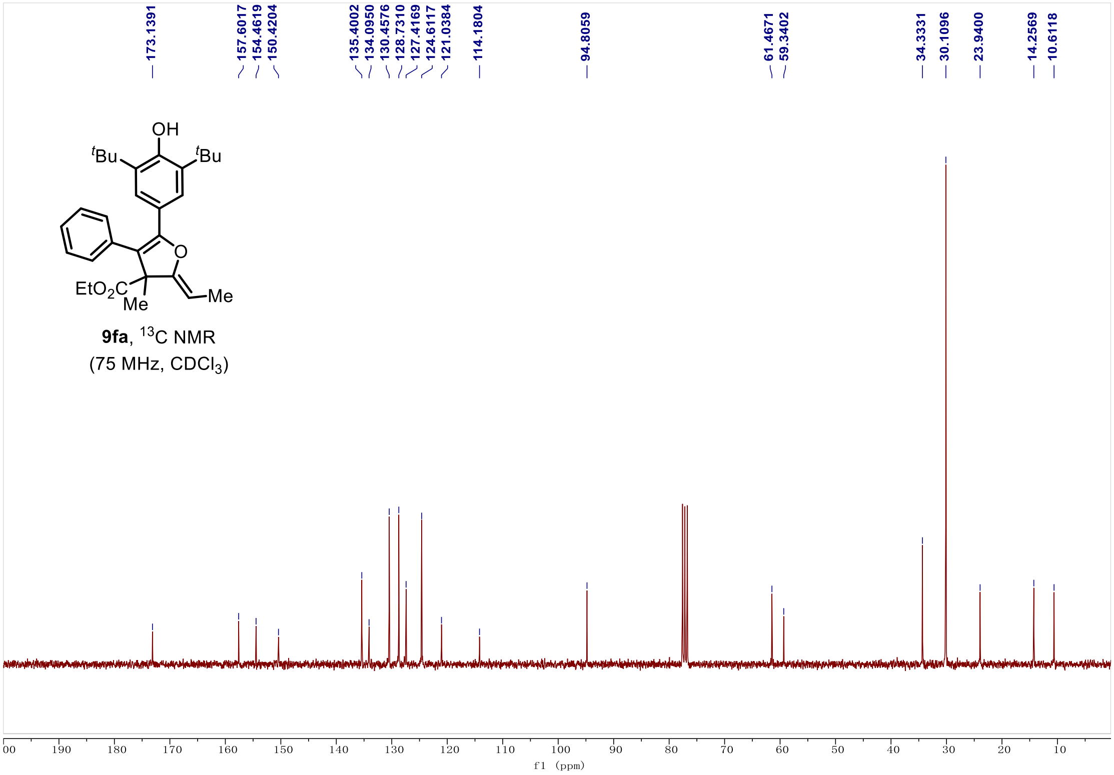
173.1391
157.6017
154.4619
150.4204
135.4002
134.0950
130.4576
128.7310
127.4169
124.6117
121.0384
114.1804
94.8059
61.4671
59.3402
34.3331
30.1096
23.9400
14.2569
10.6118
OH
tBu
tBu
O
EtO2C
Me
Me
9fa, 13C NMR
(75 MHz, CDCl3)
f1 (ppm)

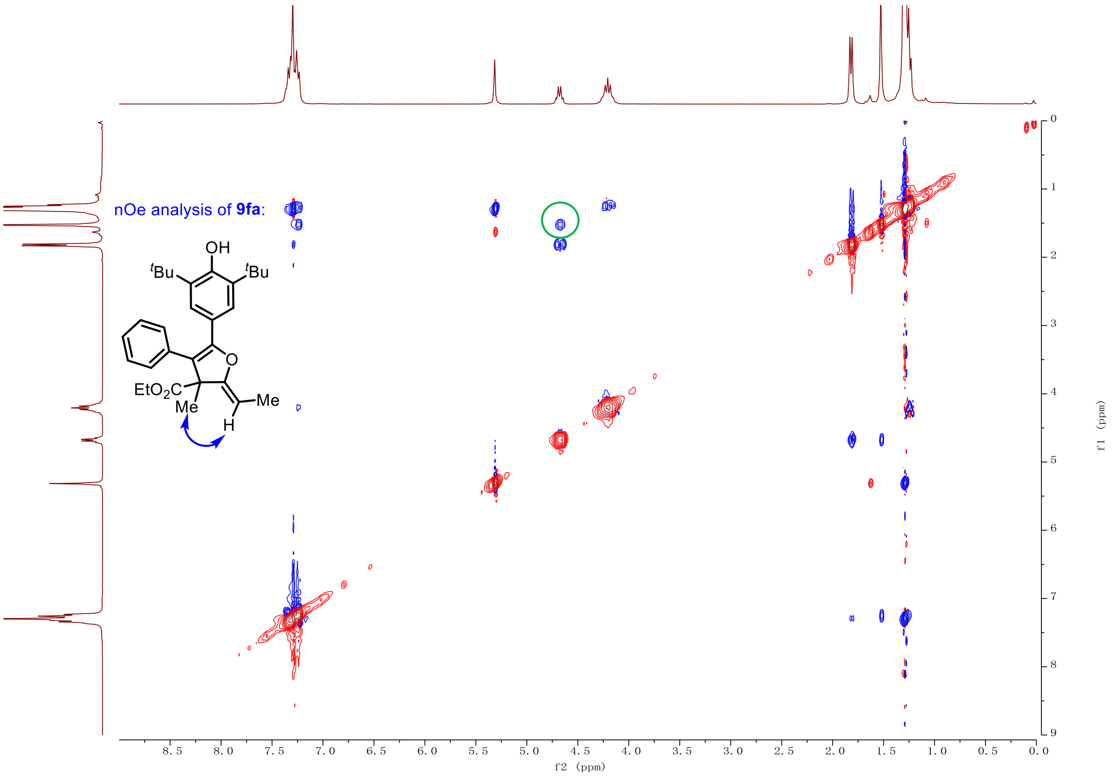
nOe analysis of 9fa:
OH
tBu
tBu
O
EtO2C
Me
Me
H
f2 (ppm)
f1 (ppm)

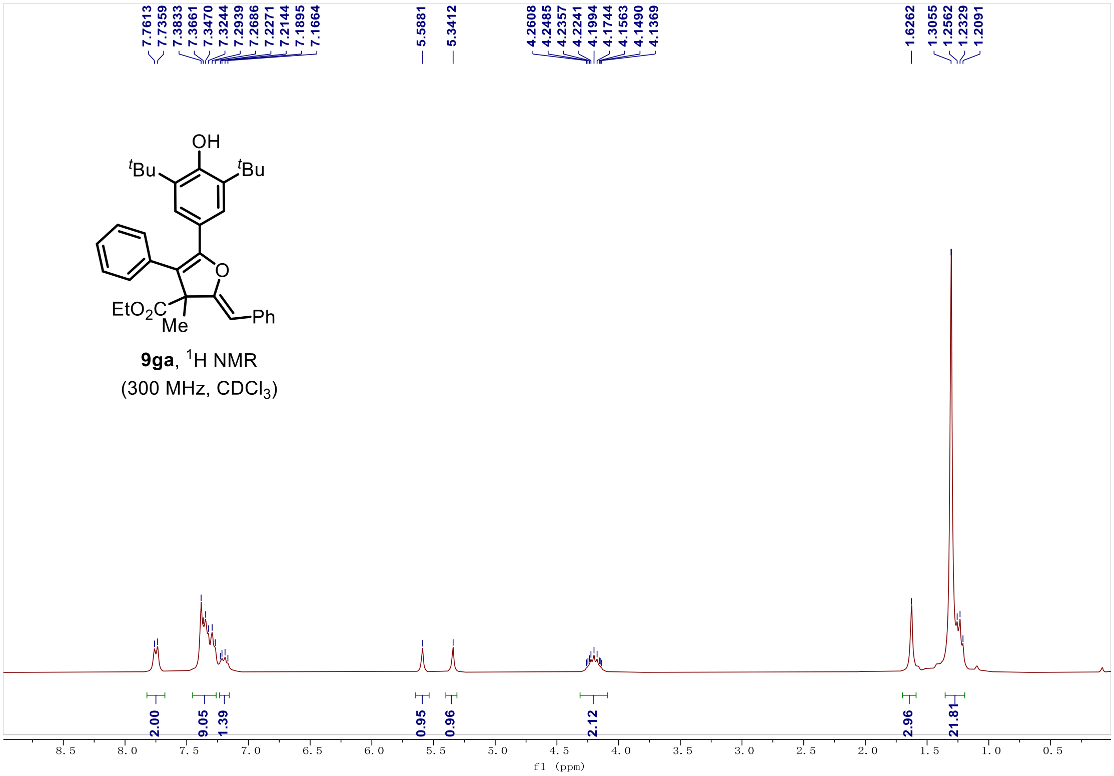
7.7613
7.7359
7.3833
7.3661
7.3470
7.3244
7.2939
7.2686
7.2271
7.2144
7.1895
7.1664
5.5881
5.3412
4.2608
4.2485
4.2357
4.2241
4.1994
4.1744
4.1563
4.1490
4.1369
1.6262
1.3055
1.2562
1.2329
1.2091
OH
tBu
tBu
O
EtO2C
Me
Ph
9ga, 1H NMR
(300 MHz, CDCl3)
2.00
9.05
1.39
0.95
0.96
2.12
2.96
21.81
f1 (ppm)

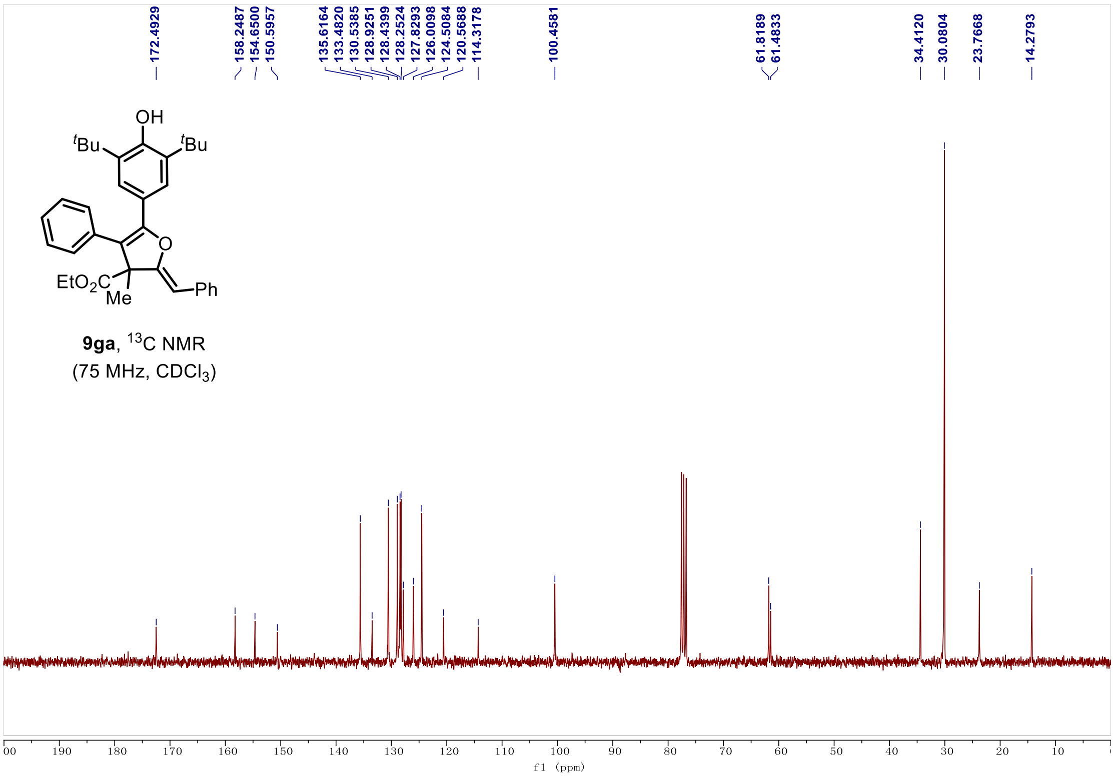
172.4929
158.2487
154.6500
150.5957
135.6164
133.4820
130.5385
128.9251
128.4399
128.2524
127.8293
126.0098
124.5084
120.5688
114.3178
100.4581
61.8189
61.4833
34.4120
30.0804
23.7668
14.2793
OH
tBu
tBu
O
EtO2C
Me
Ph
9ga, 13C NMR
(75 MHz, CDCl3)
f1 (ppm)

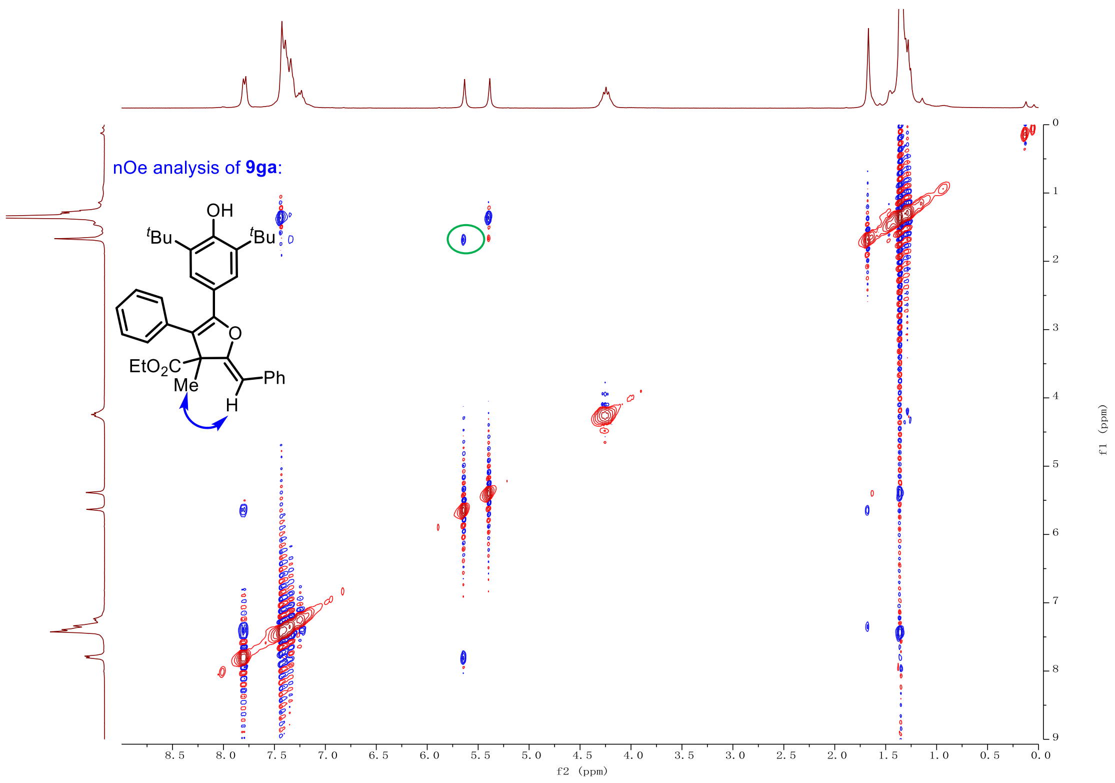

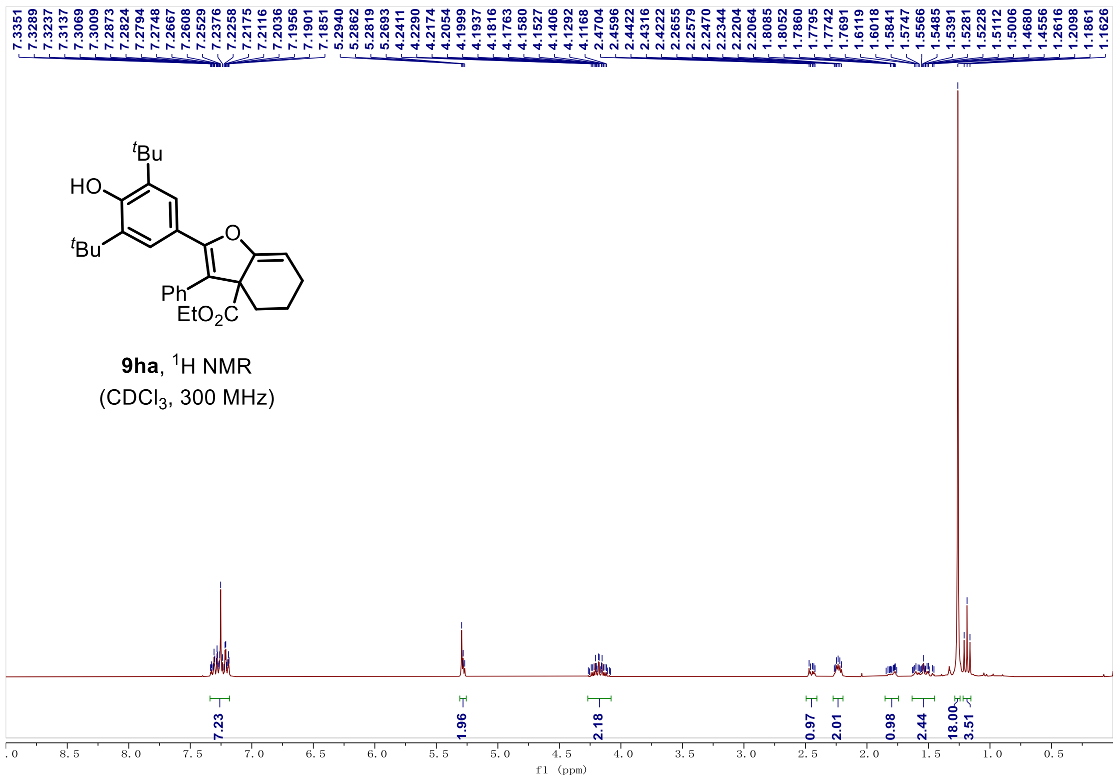

9ha, ^{1}H NMR
(CDCl$_3$, 300 MHz)

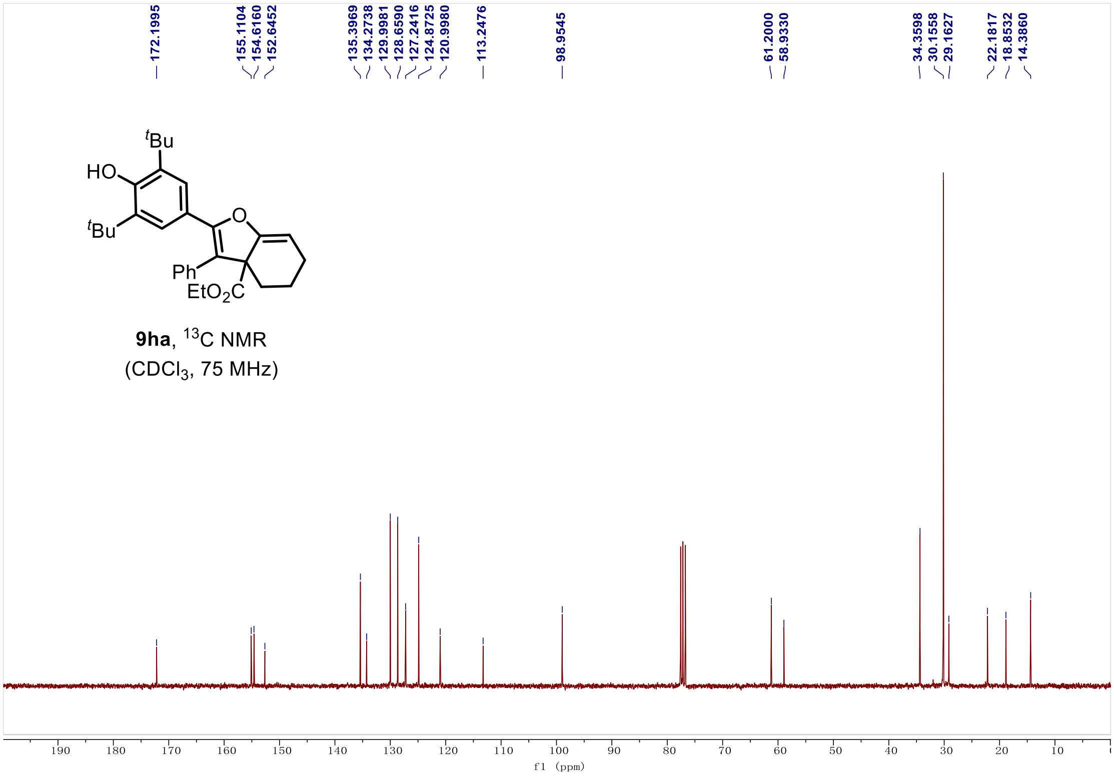
9ha, ^{13}C NMR
(CDCl$_3$, 75 MHz)
HO
^{t}Bu
Ph
EtO$_2$C
172.1995
155.1104
154.6160
152.6452
135.3969
134.2738
129.9981
128.6590
127.2416
124.8725
120.9980
113.2476
98.9545
61.2000
58.9330
34.3598
30.1558
29.1627
22.1817
18.8532
14.3860
f1 (ppm)

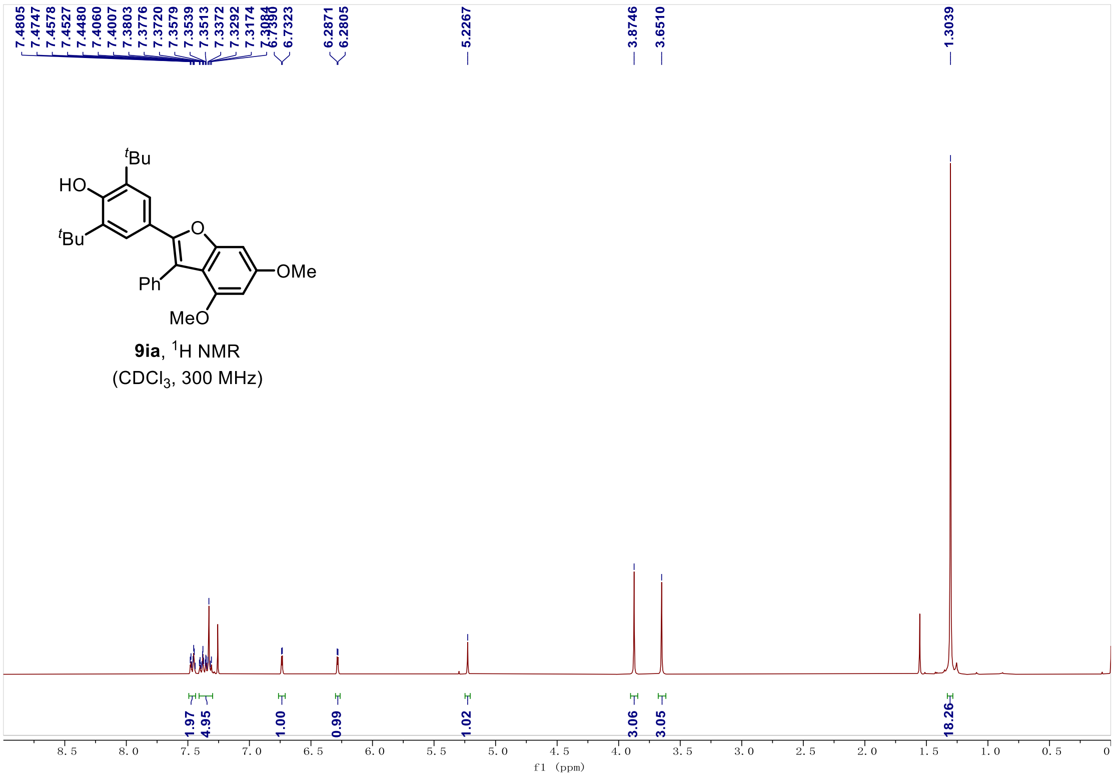
9ia, ^{1}H NMR
(CDCl$_3$, 300 MHz)
HO
^{t}Bu
Ph
OMe
MeO
6.2871
6.2805
5.2267
3.8746
3.6510
1.3039
1.97
4.95
1.00
0.99
1.02
3.06
3.05
18.26
f1 (ppm)

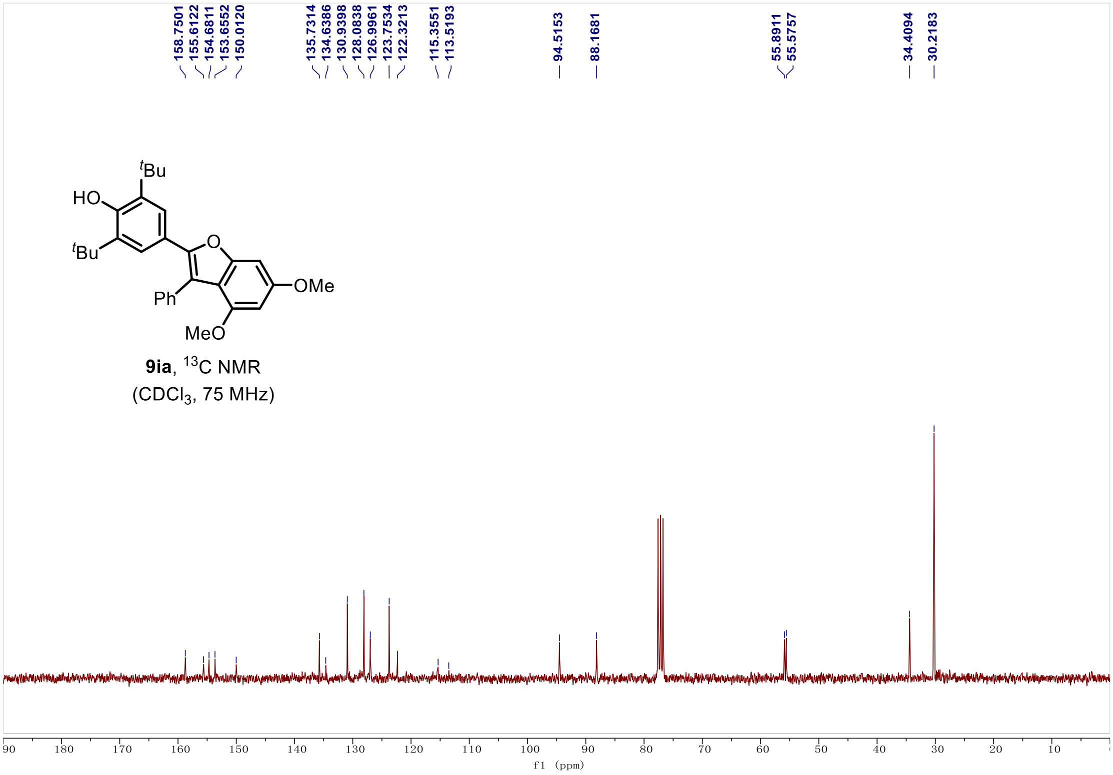
158.7501
155.6122
154.6811
153.6552
150.0120
135.7314
134.6386
130.9398
128.0838
126.9961
123.7534
122.3213
115.3551
113.5193
94.5153
88.1681
55.8911
55.5757
34.4094
30.2183
HO
tBu
tBu
Ph
OMe
MeO
O
9ia, 13C NMR
(CDCl3, 75 MHz)
f1 (ppm)

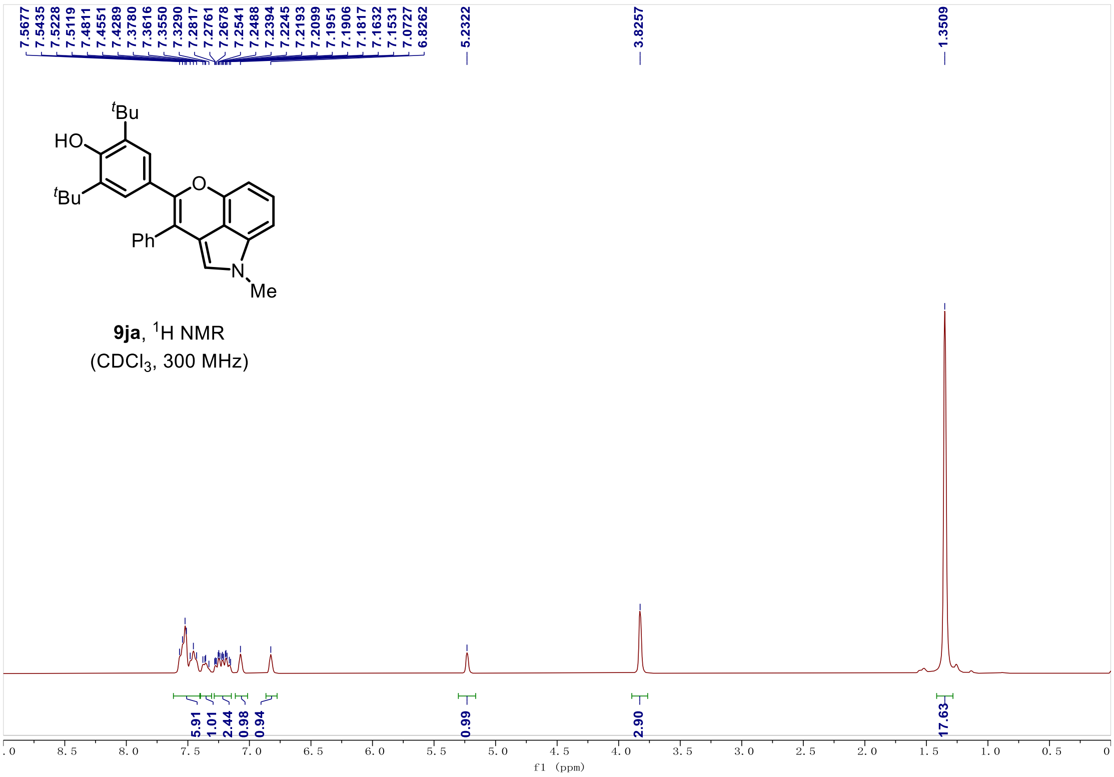
7.5677
7.5435
7.5228
7.5119
7.4811
7.4551
7.4289
7.3780
7.3616
7.3550
7.3290
7.2817
7.2761
7.2678
7.2541
7.2488
7.2394
7.2245
7.2193
7.2099
7.1951
7.1906
7.1817
7.1632
7.1531
7.0727
6.8262
5.2322
3.8257
1.3509
HO
tBu
tBu
O
Ph
N
Me
9ja, 1H NMR
(CDCl3, 300 MHz)
5.91
1.01
2.44
0.98
0.94
0.99
2.90
17.63
f1 (ppm)

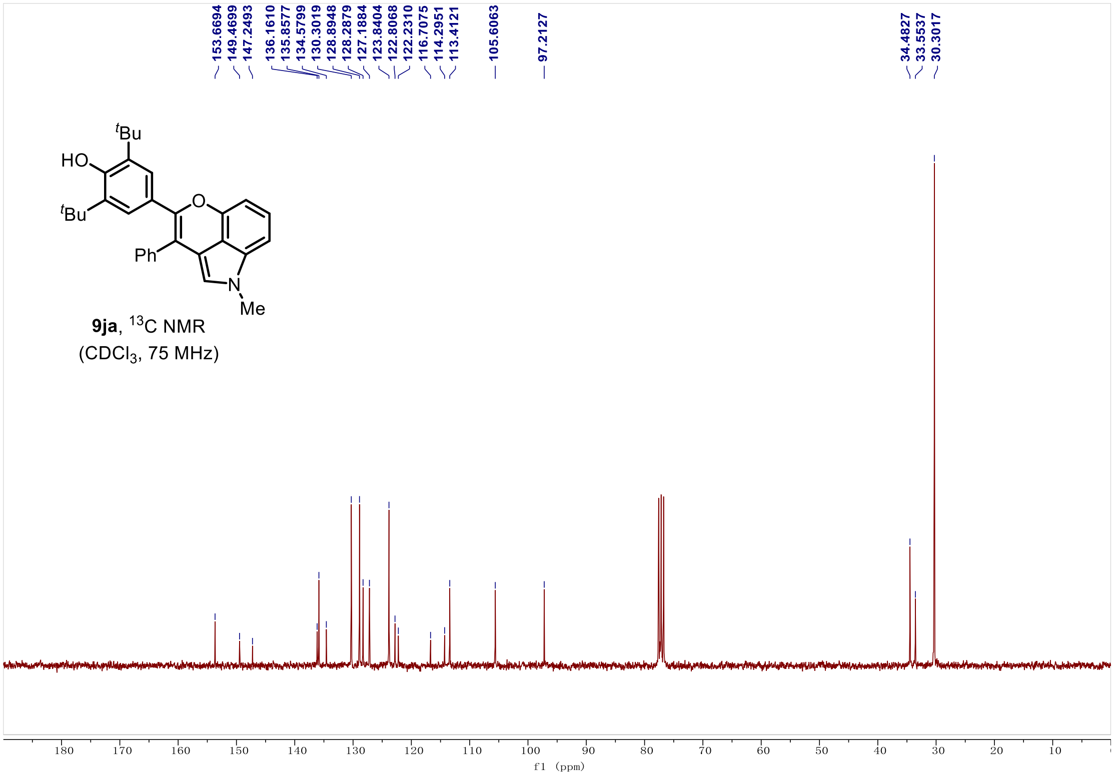

153.6694
149.4699
147.2493
136.1610
135.8577
134.5799
130.3019
128.8948
128.2879
127.1884
123.8404
122.8068
122.2310
116.7075
114.2951
113.4121
105.6063
97.2127
34.4827
33.5537
30.3017
HO
^{t}Bu
O
Ph
N
Me
9ja, ^{13}C NMR
(CDCl$_3$, 75 MHz)
f1 (ppm)

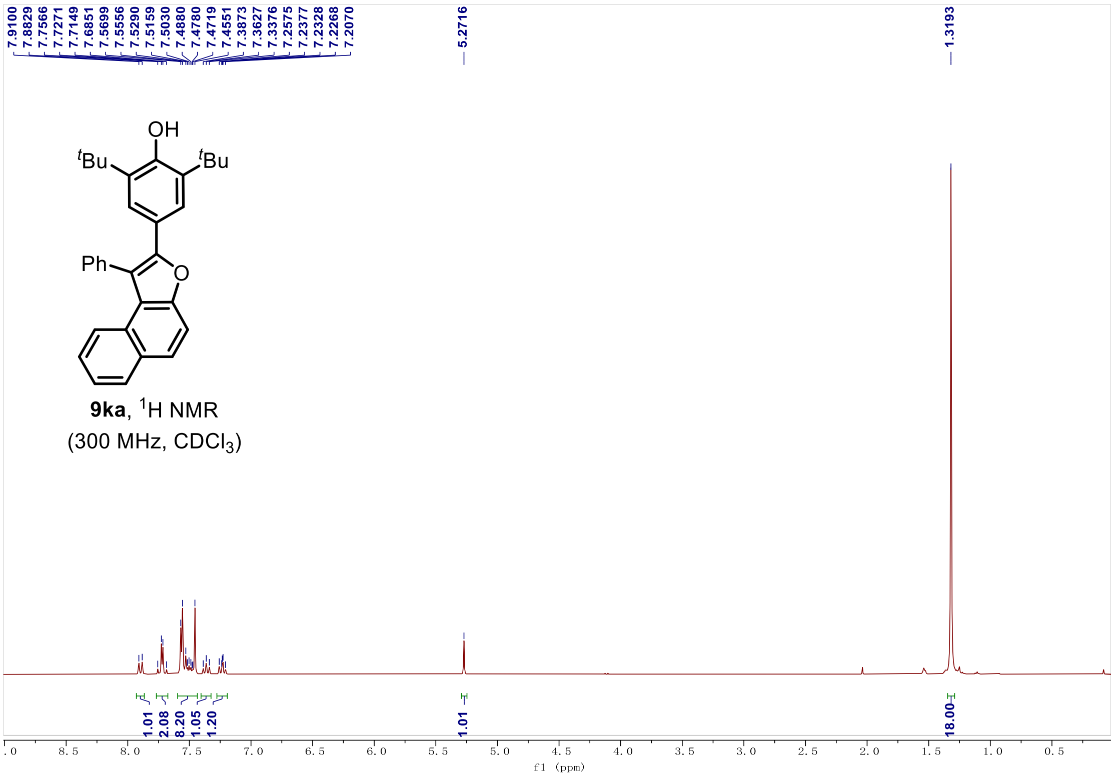

7.9100
7.8829
7.7566
7.7271
7.7149
7.6851
7.5699
7.5556
7.5290
7.5159
7.5030
7.4880
7.4780
7.4719
7.4551
7.3873
7.3627
7.3376
7.2575
7.2377
7.2328
7.2268
7.2070
5.2716
1.3193
OH
^{t}Bu
Ph
O
9ka, ^{1}H NMR
(300 MHz, CDCl$_3$)
1.01
2.08
8.20
1.05
1.20
1.01
18.00
f1 (ppm)

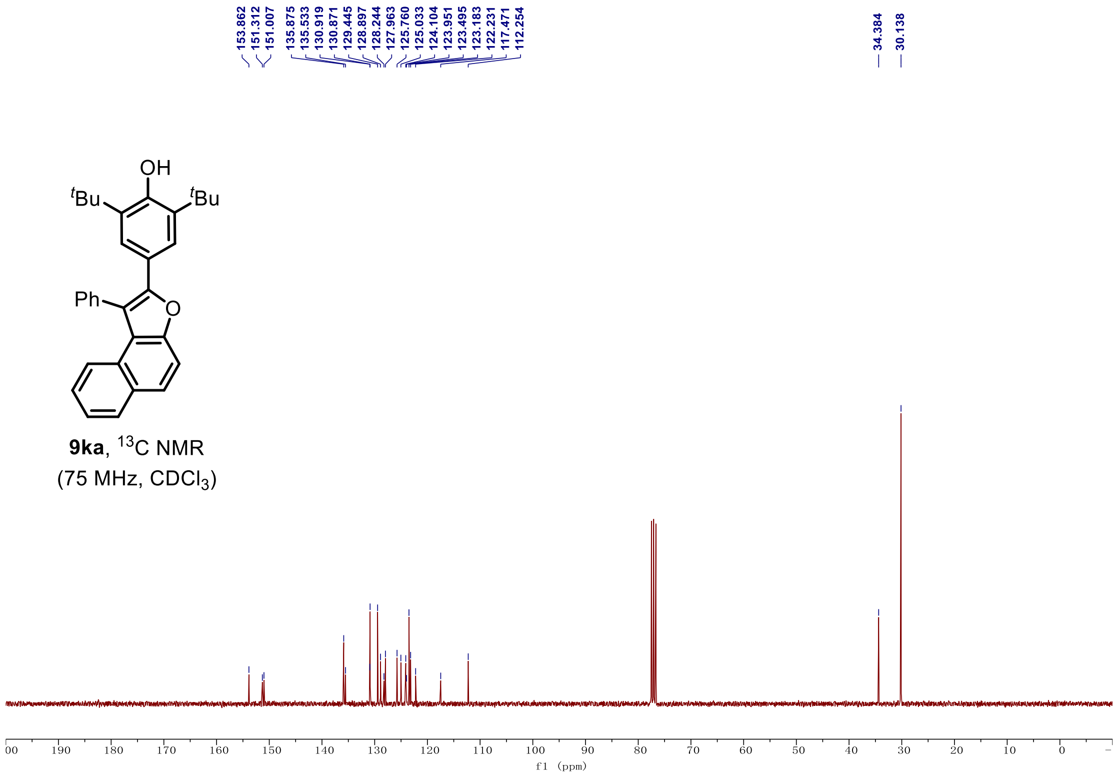
153.862
151.312
151.007
135.875
135.533
130.919
130.871
129.445
128.897
128.244
127.963
125.760
125.033
124.104
123.951
123.495
123.183
122.231
117.471
112.254
34.384
30.138
OH
tBu
tBu
Ph
O
9ka, 13C NMR
(75 MHz, CDCl3)
f1 (ppm)

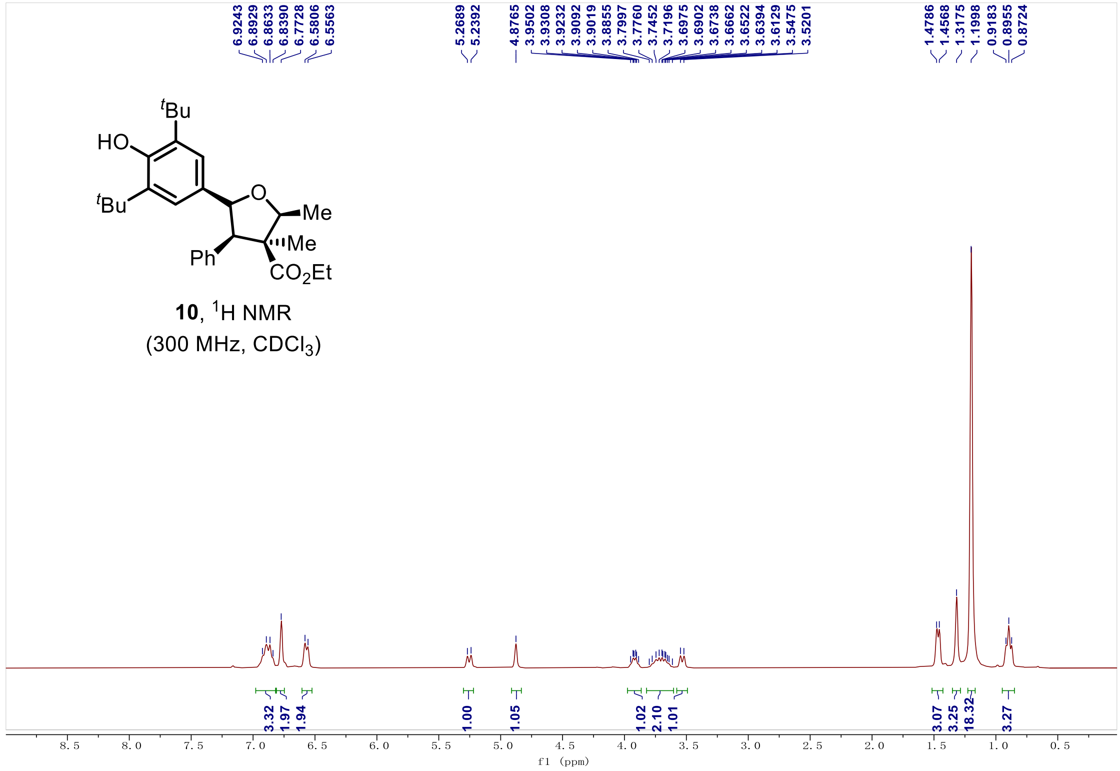
6.9243
6.8929
6.8633
6.8390
6.7728
6.5806
6.5563
5.2689
5.2392
4.8765
3.9502
3.9308
3.9232
3.9092
3.9019
3.8855
3.7997
3.7760
3.7452
3.7196
3.6975
3.6902
3.6738
3.6662
3.6522
3.6394
3.6129
3.5475
3.5201
1.4786
1.4568
1.3175
1.1998
0.9183
0.8955
0.8724
tBu
HO
tBu
O
Me
Me
Ph
CO2Et
10, 1H NMR
(300 MHz, CDCl3)
3.32
1.97
1.94
1.00
1.05
1.02
2.10
1.01
3.07
3.25
18.32
3.27
f1 (ppm)

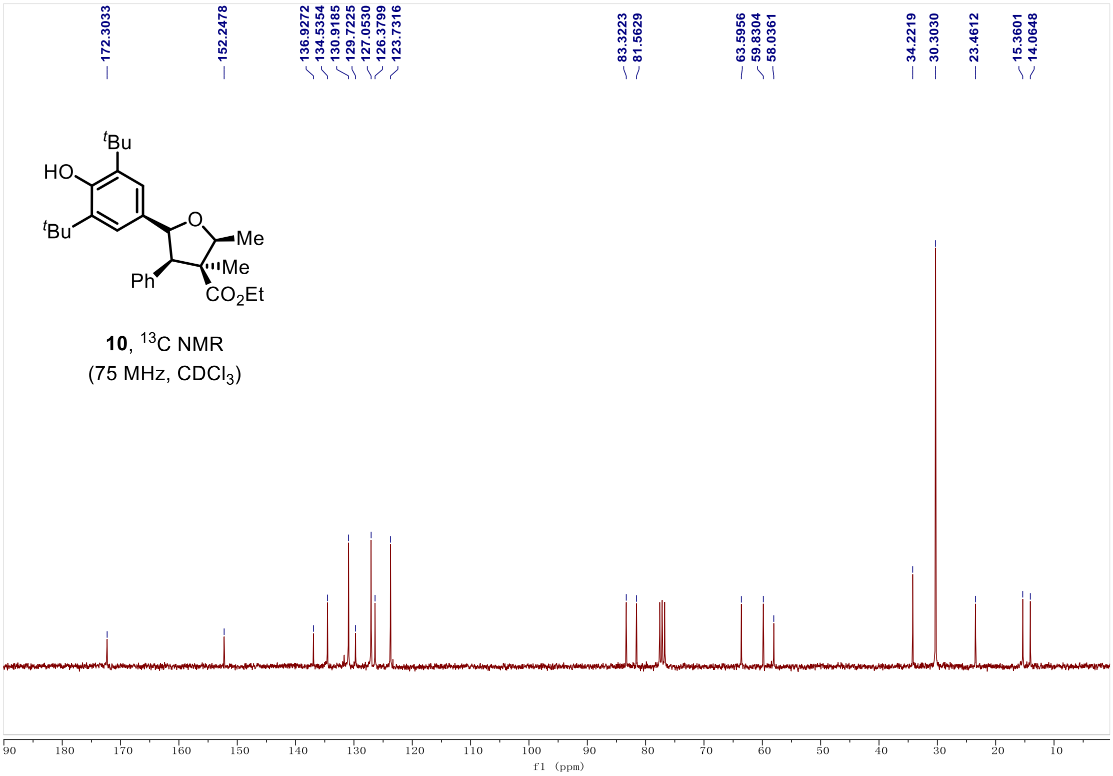
172.3033
152.2478
136.9272
134.5354
130.9185
129.7225
127.0530
126.3799
123.7316
83.3223
81.5629
63.5956
59.8304
58.0361
34.2219
30.3030
23.4612
15.3601
14.0648
HO
tBu
tBu
O
Me
Me
Ph
CO2Et
10, 13C NMR
(75 MHz, CDCl3)
f1 (ppm)

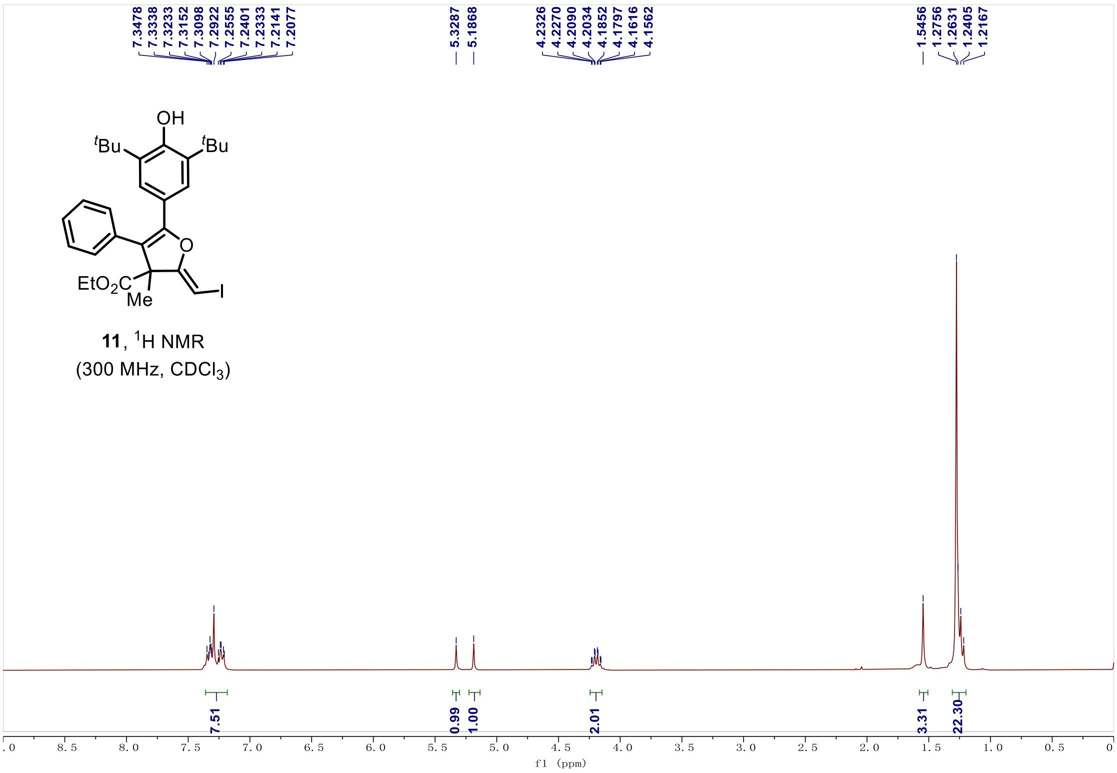
7.3478
7.3338
7.3233
7.3152
7.3098
7.2922
7.2555
7.2401
7.2333
7.2141
7.2077
5.3287
5.1868
4.2326
4.2270
4.2090
4.2034
4.1852
4.1797
4.1616
4.1562
1.5456
1.2756
1.2631
1.2405
1.2167
OH
tBu
tBu
O
EtO2C
Me
I
11, 1H NMR
(300 MHz, CDCl3)
7.51
0.99
1.00
2.01
3.31
22.30
f1 (ppm)

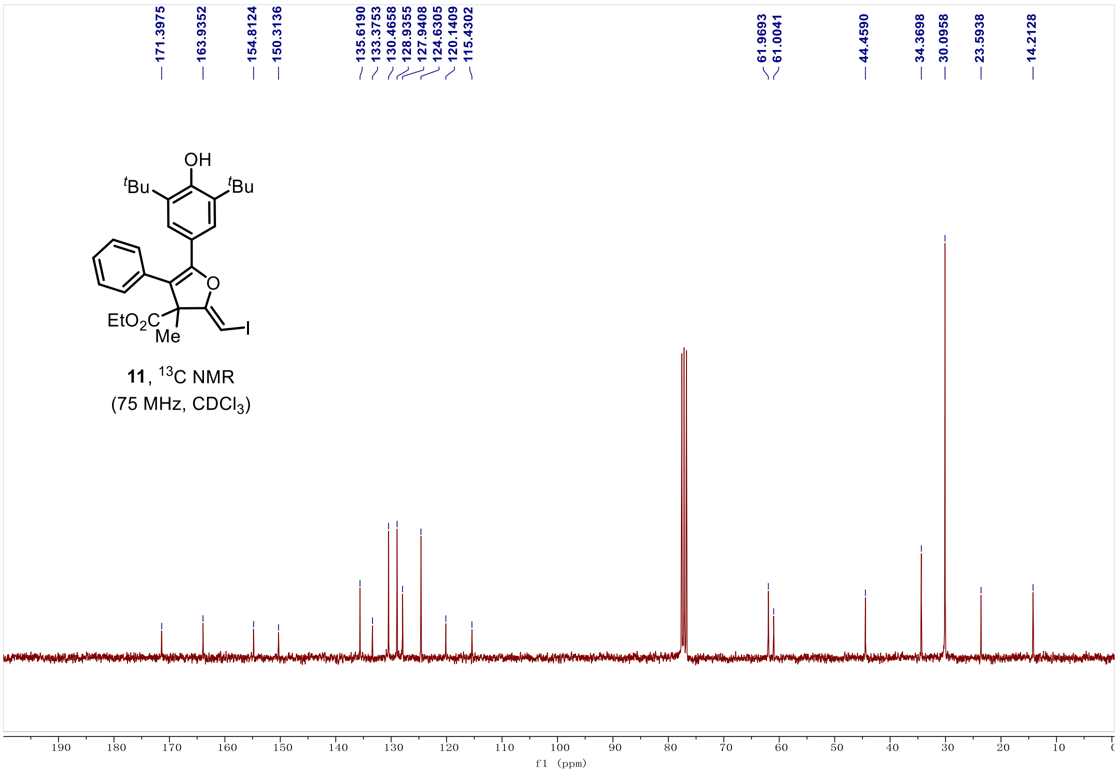
171.3975
163.9352
154.8124
150.3136
135.6190
133.3753
130.4658
128.9355
127.9408
124.6305
120.1409
115.4302
61.9693
61.0041
44.4590
34.3698
30.0958
23.5938
14.2128
OH
^{t}Bu
^{t}Bu
O
EtO_2C
Me
I
11, ^{13}C NMR
(75 MHz, $CDCl_3$)
f1 (ppm)

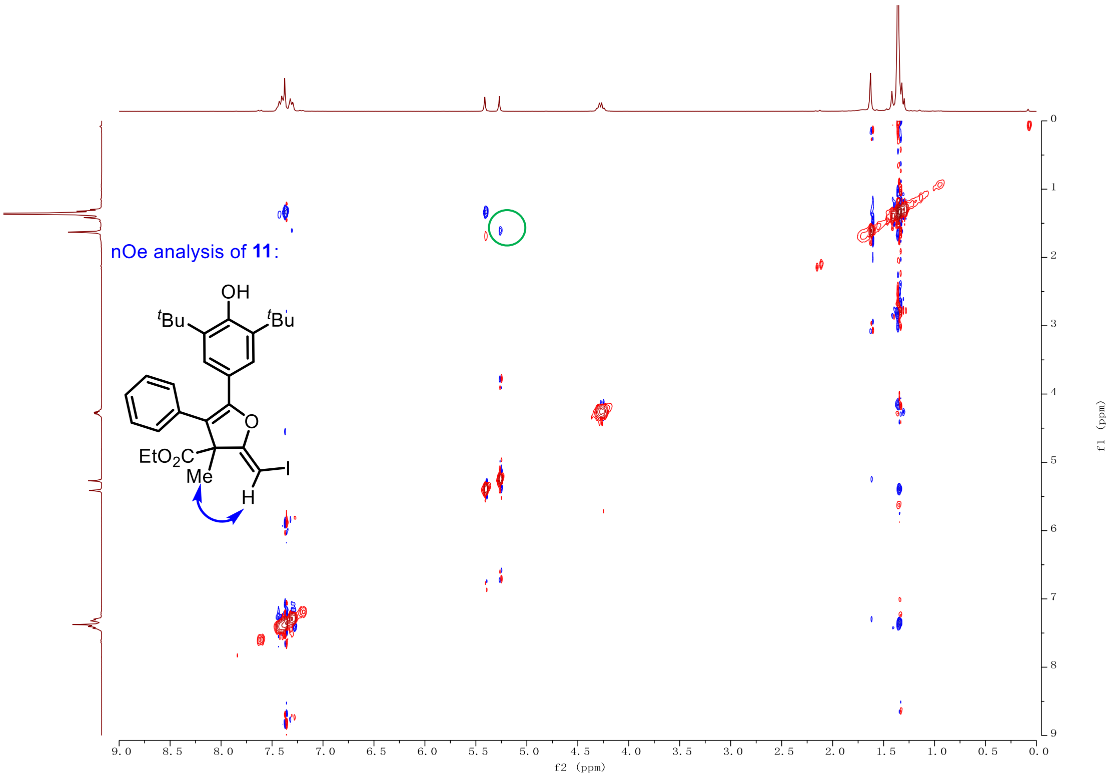
nOe analysis of 11:
OH
^{t}Bu
^{t}Bu
O
EtO_2C
Me
I
H
f2 (ppm)
f1 (ppm)

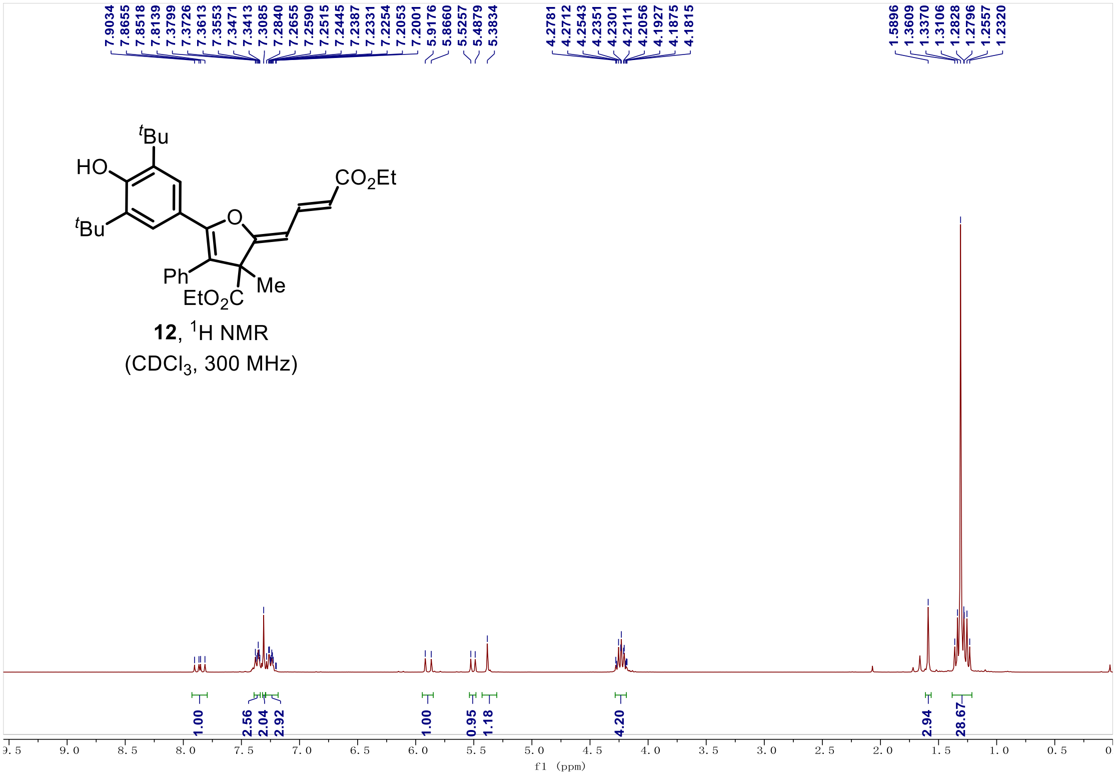
7.9034
7.8655
7.8518
7.8139
7.3799
7.3726
7.3613
7.3553
7.3471
7.3413
7.3085
7.2840
7.2655
7.2590
7.2515
7.2445
7.2387
7.2331
7.2254
7.2053
7.2001
5.9176
5.8660
5.5257
5.4879
5.3834
4.2781
4.2712
4.2543
4.2351
4.2301
4.2111
4.2056
4.1927
4.1875
4.1815
1.5896
1.3609
1.3370
1.3106
1.2828
1.2796
1.2557
1.2320
HO
tBu
tBu
CO2Et
O
Ph
EtO2C
Me
12, 1H NMR
(CDCl3, 300 MHz)
1.00
2.56
2.04
2.92
1.00
0.95
1.18
4.20
2.94
28.67
f1 (ppm)

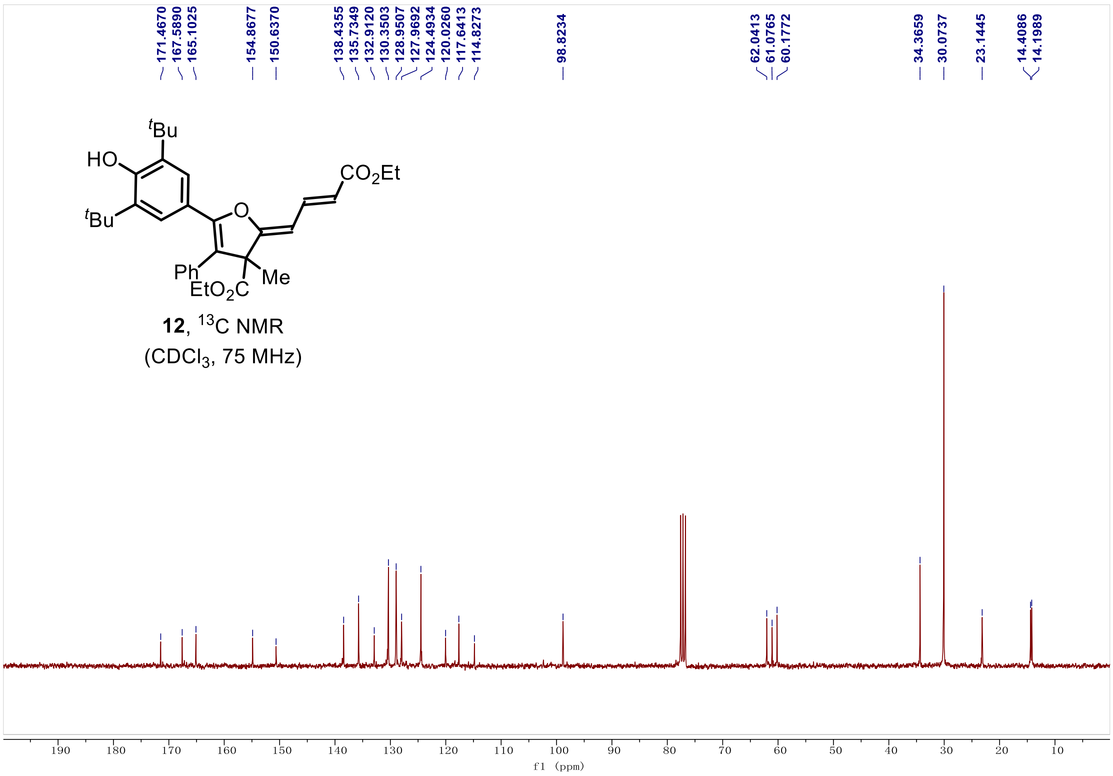
171.4670
167.5890
165.1025
154.8677
150.6370
138.4355
135.7349
132.9120
130.3503
128.9507
127.9692
124.4934
120.0260
117.6413
114.8273
98.8234
62.0413
61.0765
60.1772
34.3659
30.0737
23.1445
14.4086
14.1989
HO
tBu
tBu
CO2Et
O
Ph
EtO2C
Me
12, 13C NMR
(CDCl3, 75 MHz)
f1 (ppm)

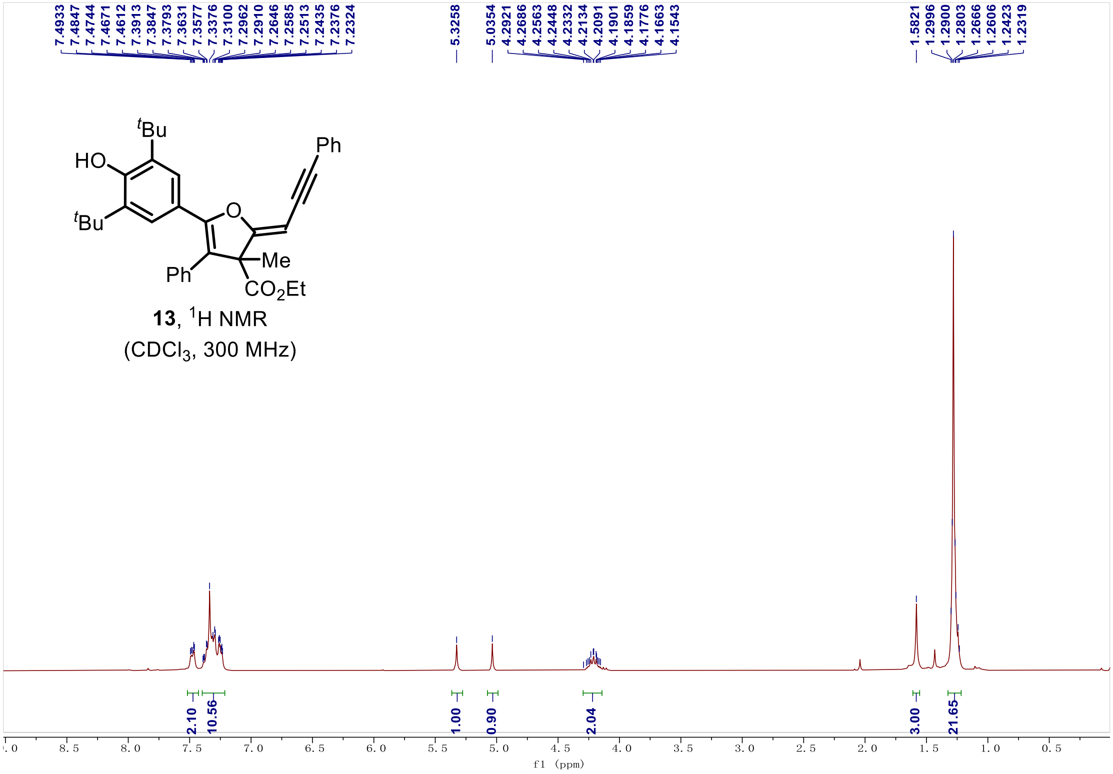

7.4933
7.4847
7.4744
7.4671
7.4612
7.3913
7.3847
7.3793
7.3631
7.3577
7.3376
7.3100
7.2962
7.2910
7.2646
7.2585
7.2513
7.2435
7.2376
7.2324
5.3258
5.0354
4.2921
4.2686
4.2563
4.2448
4.2332
4.2134
4.2091
4.1901
4.1859
4.1776
4.1663
4.1543
1.5821
1.2996
1.2900
1.2803
1.2666
1.2606
1.2423
1.2319
tBu
HO
tBu
Ph
O
Ph
Me
CO2Et
13, 1H NMR
(CDCl3, 300 MHz)
2.10
10.56
1.00
0.90
2.04
3.00
21.65
f1 (ppm)

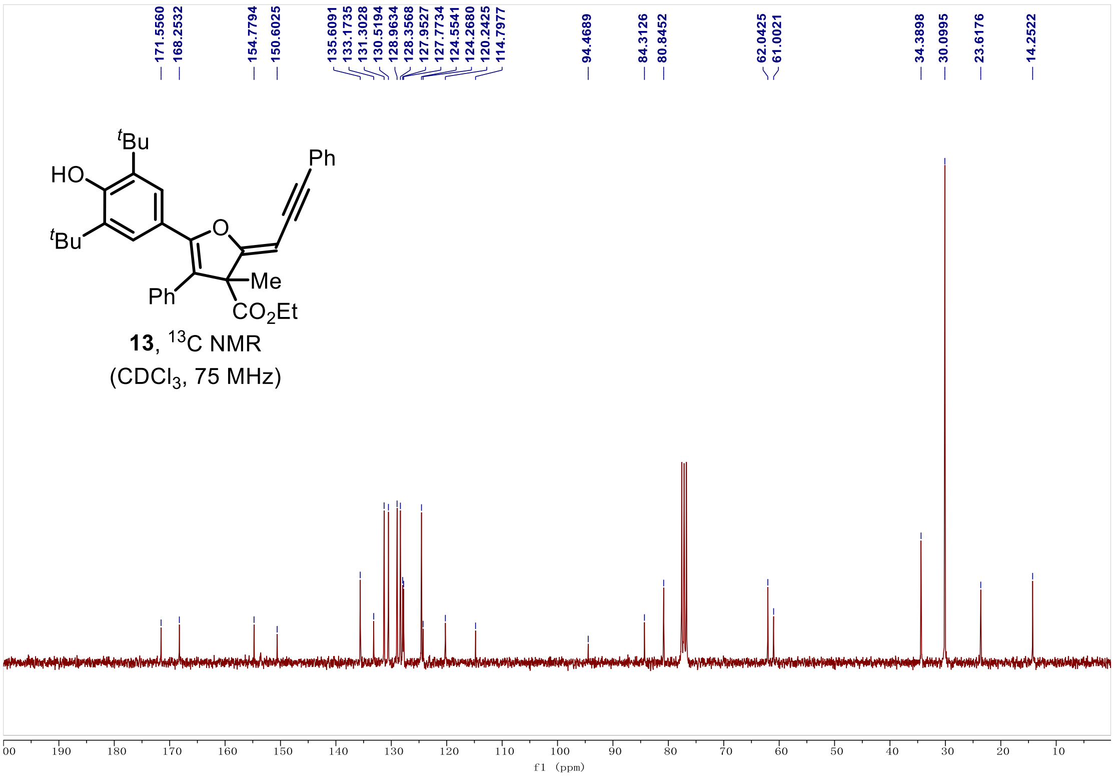

171.5560
168.2532
154.7794
150.6025
135.6091
133.1735
131.3028
130.5194
128.9634
128.3568
127.9527
127.7734
124.5541
124.2680
120.2425
114.7977
94.4689
84.3126
80.8452
62.0425
61.0021
34.3898
30.0995
23.6176
14.2522
tBu
HO
tBu
Ph
O
Ph
Me
CO2Et
13, 13C NMR
(CDCl3, 75 MHz)
f1 (ppm)

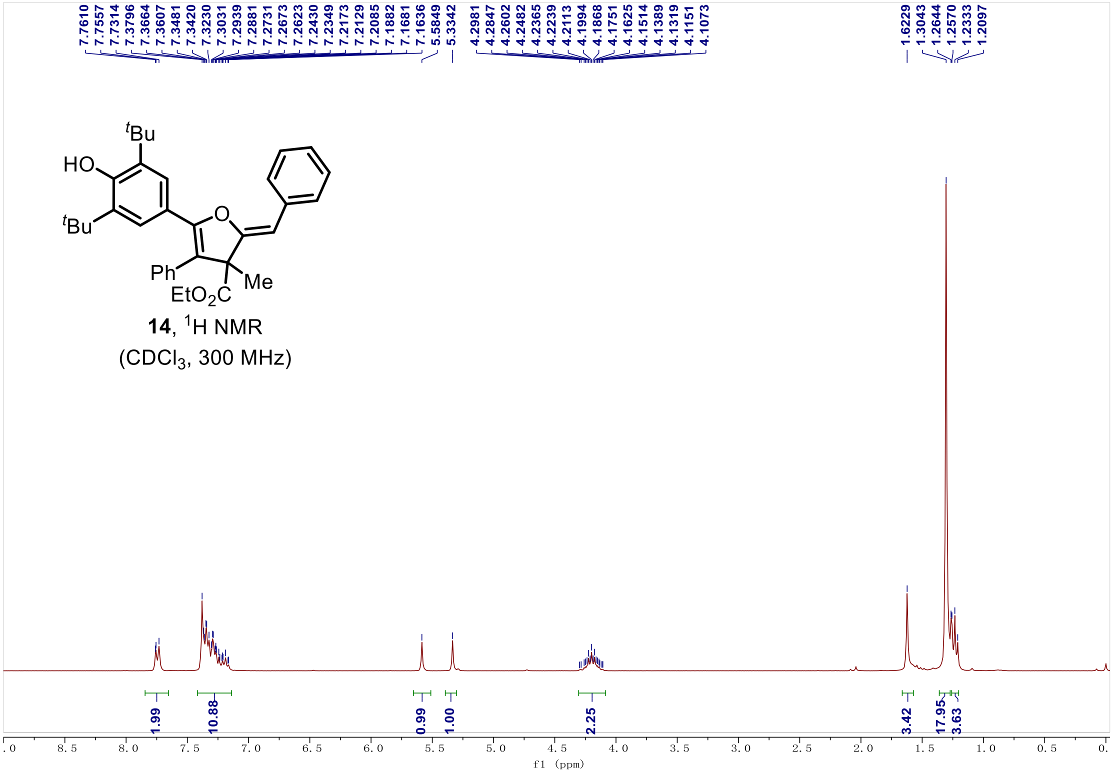
7.7610
7.7557
7.3796
7.3664
7.3607
7.3481
7.3420
7.3230
7.3031
7.2939
7.2881
7.2731
7.2673
7.2623
7.2430
7.2349
7.2173
7.2129
7.2085
7.1882
7.1681
7.1636
5.5849
5.3342
4.2981
4.2847
4.2602
4.2482
4.2365
4.2239
4.2113
4.1994
4.1868
4.1751
4.1625
4.1514
4.1389
4.1319
4.1151
4.1073
1.6229
1.3043
1.2644
1.2570
1.2333
1.2097
tBu
HO
tBu
O
Ph
Me
EtO2C
14, 1H NMR
(CDCl3, 300 MHz)
1.99
10.88
0.99
1.00
2.25
3.42
17.95
3.63
f1 (ppm)

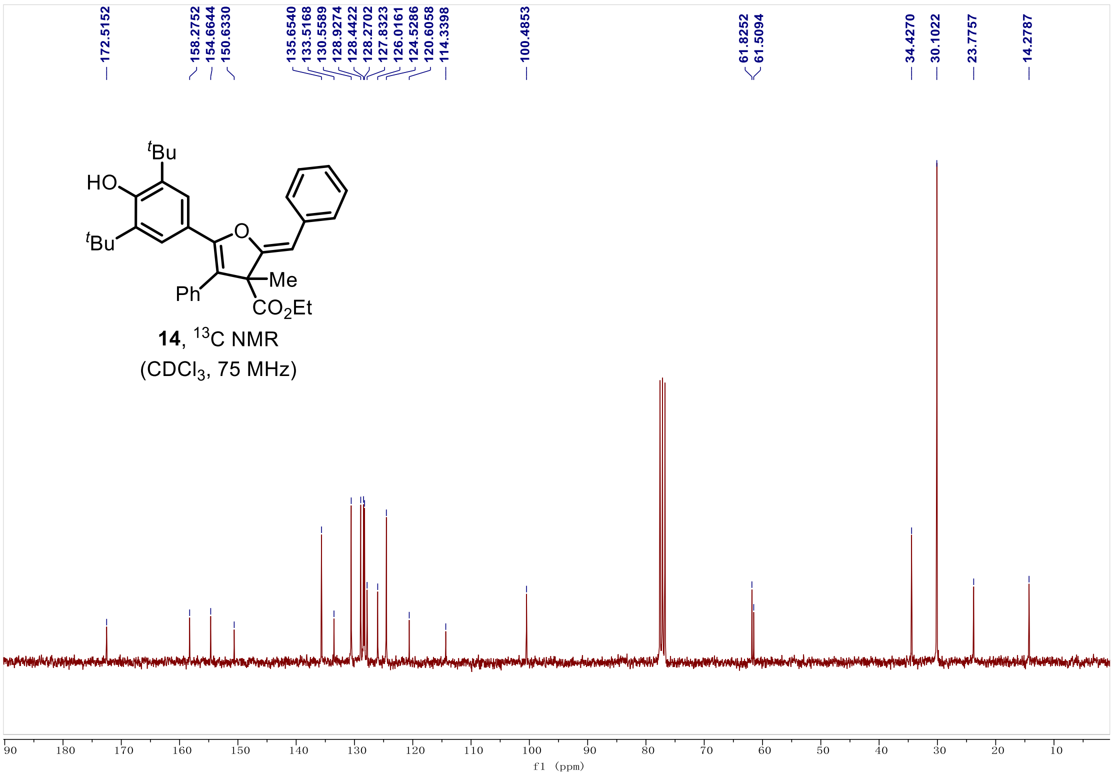
172.5152
158.2752
154.6644
150.6330
135.6540
133.5168
130.5589
128.9274
128.4422
128.2702
127.8323
126.0161
124.5286
120.6058
114.3398
100.4853
61.8252
61.5094
34.4270
30.1022
23.7757
14.2787
tBu
HO
tBu
O
Ph
Me
CO2Et
14, 13C NMR
(CDCl3, 75 MHz)
f1 (ppm)

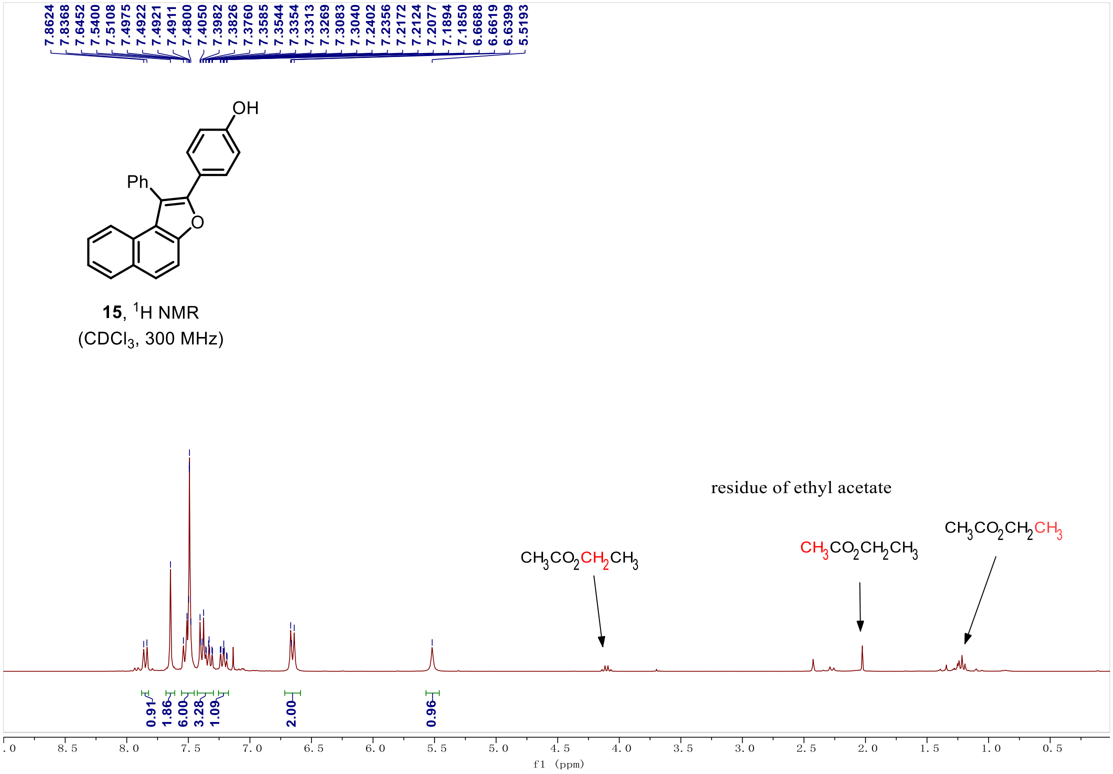

15, ^{1}H NMR
($CDCl_3$, 300 MHz)

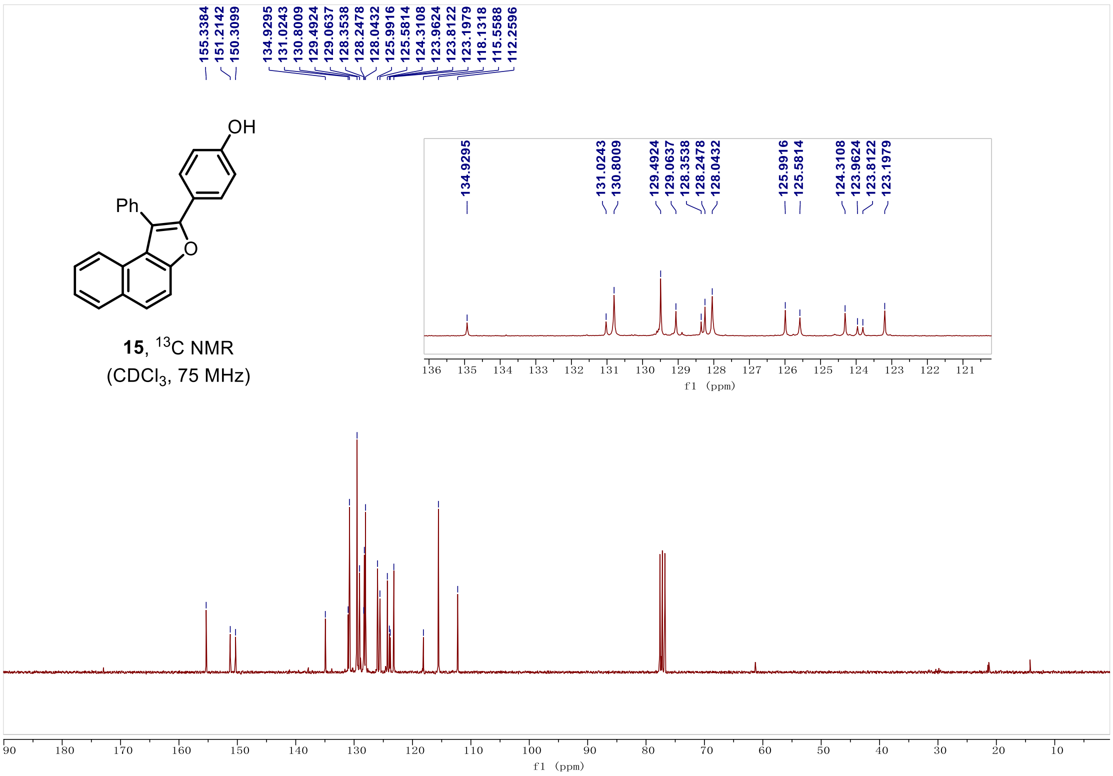

15, ^{13}C NMR
($CDCl_3$, 75 MHz)

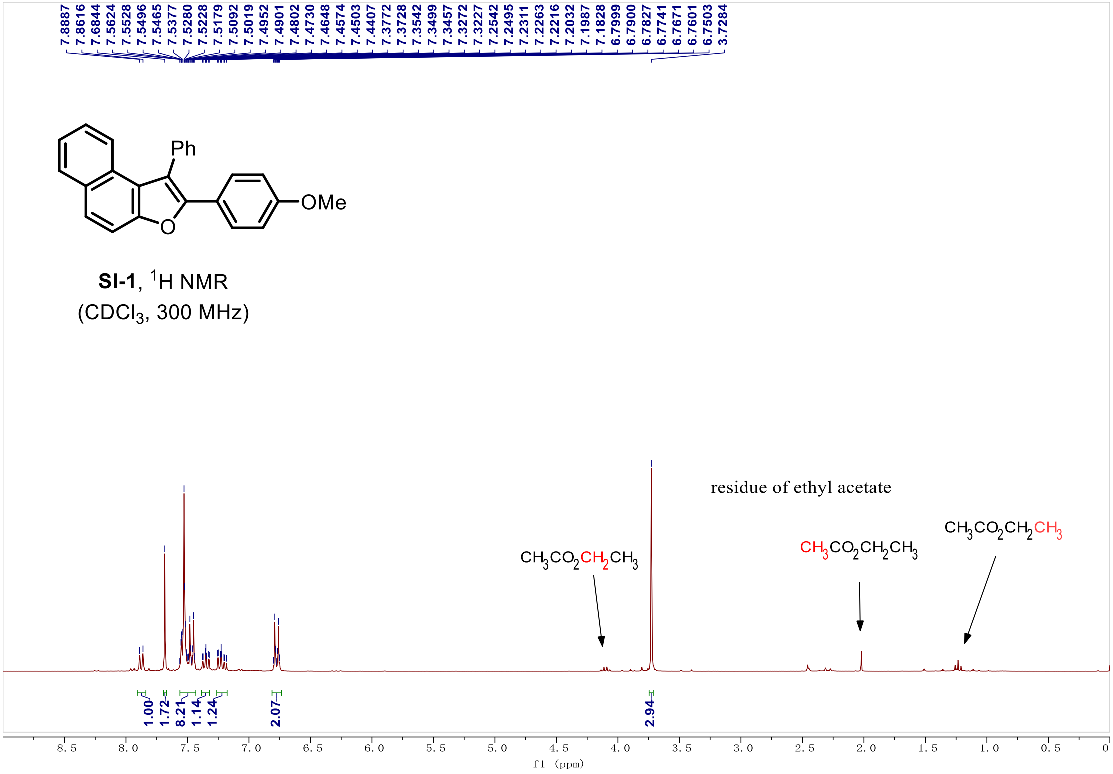
Ph
OMe
O
SI-1, 1H NMR
(CDCl3, 300 MHz)
residue of ethyl acetate
CH3CO2CH2CH3
1.00
1.72
8.21
1.14
1.24
2.07
2.94
f1 (ppm)

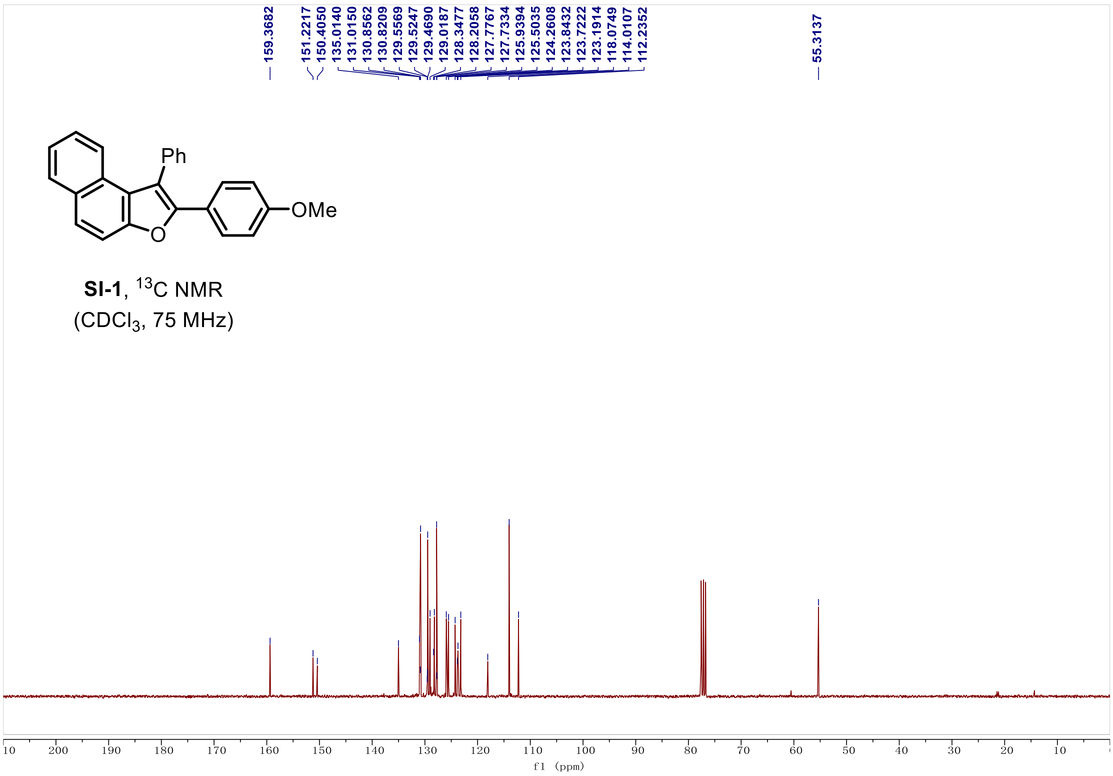
Ph
OMe
O
SI-1, 13C NMR
(CDCl3, 75 MHz)
159.3682
151.2217
150.4050
135.0140
55.3137
f1 (ppm)

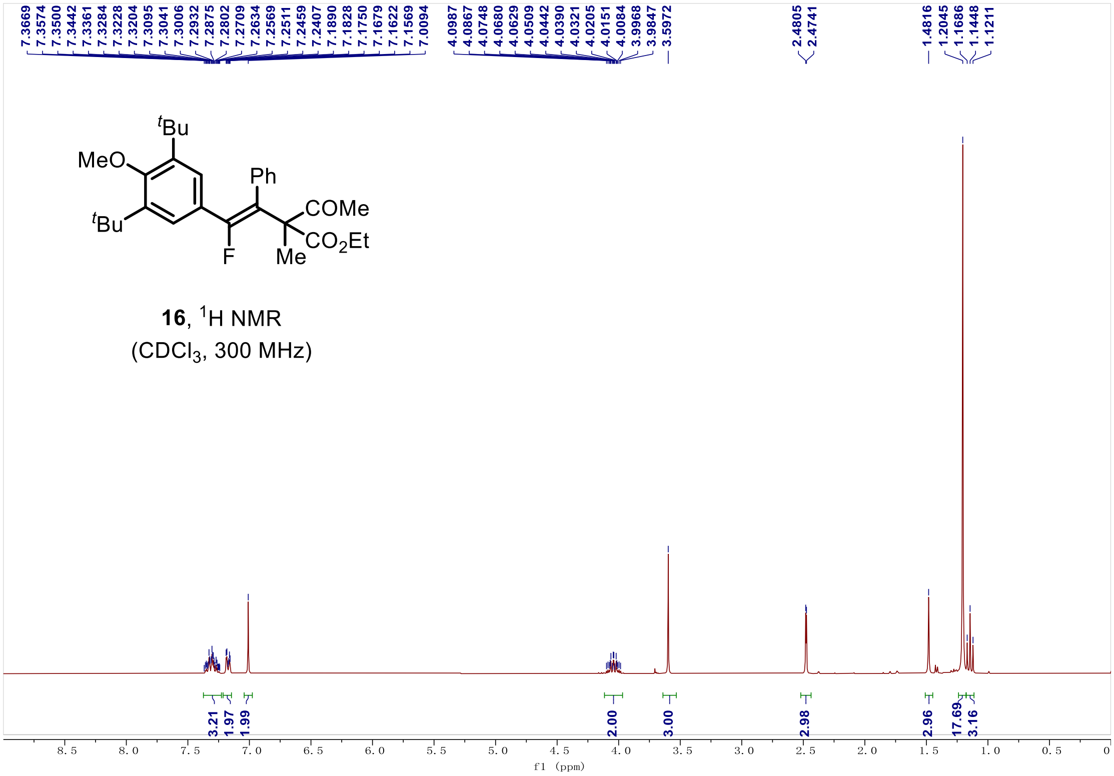
7.3669
7.3574
7.3500
7.3442
7.3361
7.3284
7.3228
7.3204
7.3095
7.3041
7.3006
7.2932
7.2875
7.2802
7.2709
7.2634
7.2569
7.2511
7.2459
7.2407
7.1890
7.1828
7.1750
7.1679
7.1622
7.1569
7.0094
4.0987
4.0867
4.0748
4.0680
4.0629
4.0509
4.0442
4.0390
4.0321
4.0205
4.0151
4.0084
3.9968
3.9847
3.5972
2.4805
2.4741
1.4816
1.2045
1.1686
1.1448
1.1211
tBu
MeO
Ph
COMe
tBu
CO2Et
F
Me
16, 1H NMR
(CDCl3, 300 MHz)
3.21
1.97
1.99
2.00
3.00
2.98
2.96
17.69
3.16
f1 (ppm)

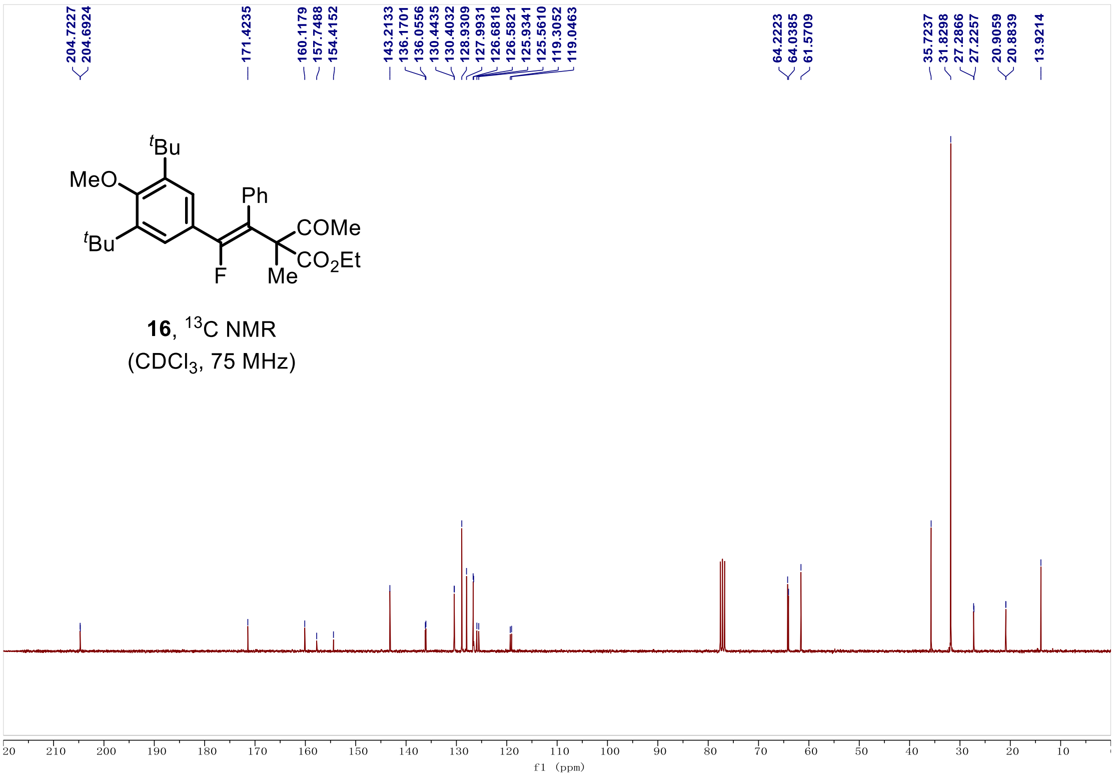
204.7227
204.6924
171.4235
160.1179
157.7488
154.4152
143.2133
136.1701
136.0556
130.4435
130.4032
128.9309
127.9931
126.6818
126.5821
125.9341
125.5610
119.3052
119.0463
64.2223
64.0385
61.5709
35.7237
31.8298
27.2866
27.2257
20.9059
20.8839
13.9214
tBu
MeO
Ph
COMe
tBu
CO2Et
F
Me
16, 13C NMR
(CDCl3, 75 MHz)
f1 (ppm)

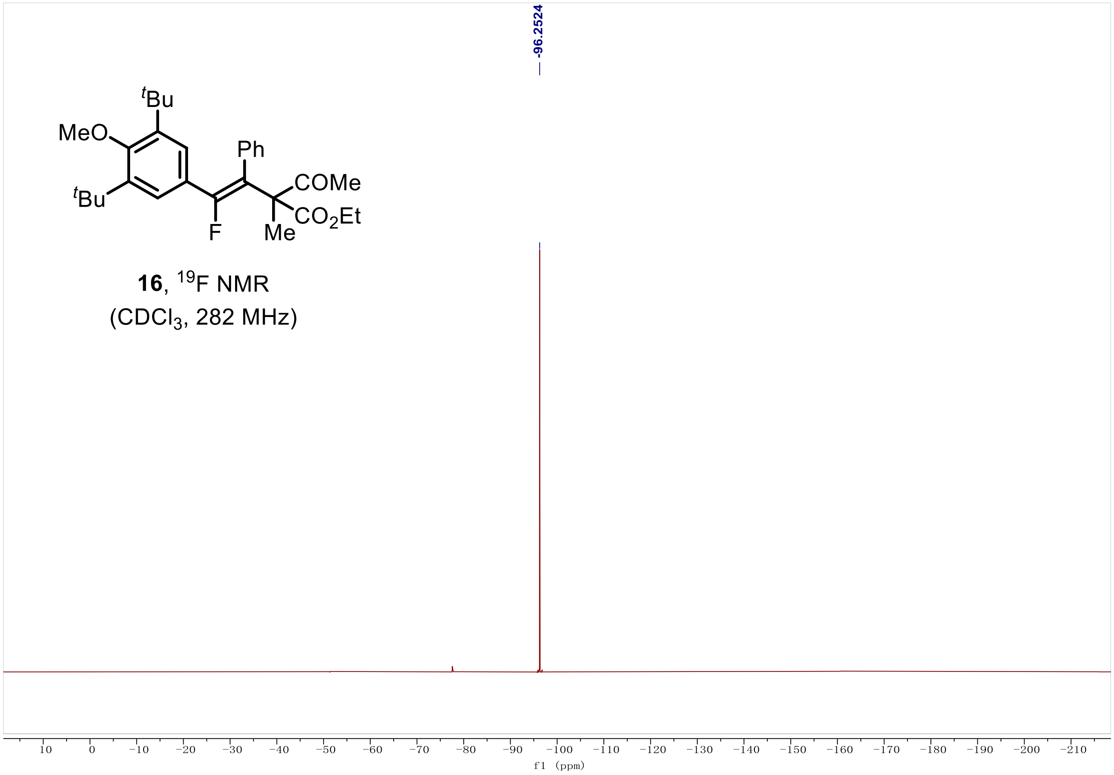
-96.2524
tBu
MeO
tBu
Ph
COMe
CO2Et
F
Me
16, 19F NMR
(CDCl3, 282 MHz)
10 0 −10 −20 −30 −40 −50 −60 −70 −80 −90 −100 −110 −120 −130 −140 −150 −160 −170 −180 −190 −200 −210
f1 (ppm)
